# Integration of Machine Learning Methods to Dissect Genetically Imputed Transcriptomic Profiles in Alzheimer’s Disease

**DOI:** 10.3389/fgene.2019.00726

**Published:** 2019-09-03

**Authors:** Carlo Maj, Tiago Azevedo, Valentina Giansanti, Oleg Borisov, Giovanna Maria Dimitri, Simeon Spasov, Alzheimer’s Disease Neuroimaging Initiative, Pietro Lió, Ivan Merelli

**Affiliations:** ^1^Institute for Genomic Statistics and Bioinformatics, University Hospital Bonn, Bonn, Germany; ^2^Department of Computer Science and Technology, University of Cambridge, Cambridge, United Kingdom; ^3^National Research Council, Institute for Biomedical Technologies, Milan, Italy

**Keywords:** eQTL, gene expression imputation, GTEx, variational autoencoder, support vector machine, deep learning, recurrent neural networks, Alzheimer’s

## Abstract

The genetic component of many common traits is associated with the gene expression and several variants act as expression quantitative loci, regulating the gene expression in a tissue specific manner. In this work, we applied tissue-specific cis-eQTL gene expression prediction models on the genotype of 808 samples including controls, subjects with mild cognitive impairment, and patients with Alzheimer's Disease. We then dissected the imputed transcriptomic profiles by means of different unsupervised and supervised machine learning approaches to identify potential biological associations. Our analysis suggests that unsupervised and supervised methods can provide complementary information, which can be integrated for a better characterization of the underlying biological system. In particular, a variational autoencoder representation of the transcriptomic profiles, followed by a support vector machine classification, has been used for tissue-specific gene prioritizations. Interestingly, the achieved gene prioritizations can be efficiently integrated as a feature selection step for improving the accuracy of deep learning classifier networks. The identified gene-tissue information suggests a potential role for inflammatory and regulatory processes in gut-brain axis related tissues. In line with the expected low heritability that can be apportioned to eQTL variants, we were able to achieve only relatively low prediction capability with deep learning classification models. However, our analysis revealed that the classification power strongly depends on the network structure, with recurrent neural networks being the best performing network class. Interestingly, cross-tissue analysis suggests a potentially greater role of models trained in brain tissues also by considering dementia-related endophenotypes. Overall, the present analysis suggests that the combination of supervised and unsupervised machine learning techniques can be used for the evaluation of high dimensional omics data.

## Introduction

Nowadays researchers can access omics data at different levels, such as genomics (e.g., dbGaP^1^), transcriptomics (e.g., GEO expression^2^) and also at multi-omics levels (e.g., GTEx^3^, Encode^4^). Given the advancement of high-throughput technologies, the increasing availability of omics data can be expected over time. This will allow researchers to better analyze complex systems characterized by many interacting features as the biological systems.

Traditional analytical methods on omics data, such as Genome-wide association study (GWAS) and differential expression analysis, usually rely on univariate approaches with specific statistical modelling (Visscher et al., 2017; McDermaid et al., 2018). These approaches, despite being robust, are limited in detecting potential combinatorial effects in the underlying biological system. Indeed, biological networks can be highly complex with many feedback regulatory loops (Franco and Galloway, 2015). A comprehensive analysis of interaction effects is not feasible with traditional approaches due to the combinatorial explosion of the input factor space (Berger et al., 2013).

On the other hand, machine learning methods have proved to be efficient for the analysis of high dimensional complex systems, although the application of machine learning methods in omics data is still relatively uncommon due to the limited interpretability of the outcome of machine learning frameworks (Li et al., 2016). In this work, we investigate the applicability of different machine learning methods on omics data using, as a case study, matrices of tissue-specific predicted transcriptomic profiles in Alzheimer’s disease (AD). AD is a progressive neurodegenerative disorder, representing the predominant form of dementia (Wang et al., 2017), and is characterized by progressive deterioration of memory and cognitive functions that can be tested with different clinical tests (Kirsebom et al., 2017). The pathophysiology of AD involves the formation of the characteristic extracellular amyloid plaques and intracellular neurofibrillary tangles (Kuznetsov and Kuznetsov, 2018).

A lot of research has been done in order to identify the genetics factor contributing to AD. In cases of specific familiar forms of AD, which are recurrent among family members and are characterized by early onset (i.e., age < 65), disease causing mutations in specific genes have been identified, namely amyloid precursor protein (APP), Presenilin 1 PSEN1 and Presenilin 2 PSEN2 (Piaceri et al., 2013). This is not the case of the most common sporadic AD forms, characterized by late onset (age > 65), representing about 95% of AD cases (Bali et al., 2012), for which the “4 allele of Apolipoprotein E (APOE) is the only strong identified genetic risk factor (Dorszewska et al., 2016).

However, the relatively high heritability also of sporadic AD, estimated to be around 60% to 80% (Van Cauwenberghe et al., 2016), combined with the identification of a number of genetic risk loci from GWAS, suggests the presence of a polygenic component in late onset AD (Escott-Price et al., 2015). Indeed, GWAS hits can be associated with different biological pathways, such as cholesterol and lipid metabolism, immune system, inflammatory response, and endosomal vesicle cycling (Lambert et al., 2013). Moreover, several susceptibility loci are localized in gene-dense regions, but it remains unknown which genes of these regions are responsible for the association (Van Cauwenberghe et al., 2016). In fact, identifying the functional role of variants in intergenic regions is not a trivial process, since the related genes might not be the closest to the loci (e.g., chromatin 3D structure can place in proximity relatively distant region in the primary DNA sequence) (Dekker et al., 2013). Moreover, many complex phenotypes have a polygenic architecture, in which many variants have minor effects over a phenotype, and polygenic risk score modeling is capable of finding significant genetic associations for traits with no monogenic causes, but with relatively high heritability (Chatterjee et al., 2016).

Different works show a co-localization between Expression Quantitative Loci (eQTL) and GWAS hits indicating that the biological effect of non-coding variants can be exerted through the regulation of gene expression (Hormozdiari et al., 2016; Wen et al., 2017), that is a polygenic trait in which many variants may be involved. Indeed, different tools model the combined effect of eQTL signals, considering both strong functional SNP effects and additive effects for modest-strength signals (Gamazon et al., 2015; Gusev et al., 2016). Conducting gene association on the basis of the genetic component of gene expression regulation, also called Transcription Wide Association Study (TWAS), proved to be particularly efficient in finding associations with many traits (Gusev et al., 2016).

There are many advantages in testing the genetic component of gene expression rather than evaluating the nominal variant GWAS association: I) the aggregation of multiple eQTL into one gene can boost the association by including additive effect among variants; II) genes are more interpretable biological unit in comparison with variants; III) the statistical power is increased due to the reduction of multiple-comparison tests from hundreds of thousands/million variants (before/after imputation) to the order of thousands of genes (after filtering for gene expression heritability); IV) eQTL are tissue specific and therefore it is possible to perform gene association analysis in the target tissue for the phenotypes and also in secondary tissues for potential peripheral biomarkers (e.g., blood).

Noteworthy, the evaluation of the solely genetic component of gene expression is less comprehensive than the actual gene expression analysis, but has the advantage to focus only on the genetic/heritable component, avoiding environmental confounding effects (Gamazon et al., 2015). Since polygenic effects can be expected also at gene expression level, given the complexity of biochemical systems, performing multi-gene evaluation can provide greater insights concerning potential biological associations (Marigorta et al., 2017). Therefore, machine learning and deep learning methodologies have proved to be efficient at identifying transcriptomic profiles associated with specific phenotypes, considering different input data, such as measured RNA-seq data (Wang et al., 2018), single cell expression (Hu et al., 2016), and also imputed transcriptomic data (Gottlieb et al., 2017).

In this work, we tested multiple machine learning and deep learning approaches to study multi-tissue imputed transcriptomic profiles in the Alzheimer’s Disease Neuroimaging Initiative (ADNI) cohort (Weiner et al., 2013). Noteworthy, the analysis of imputed transcriptomic profiles on ADNI data has been already performed at single gene level identifying, suggesting potential specific gene-tissue associations with amyloid deposition (Hohman et al., 2017). In the following sections we introduce the supervised and unsupervised methods we exploited in this work, the results achieved combining these approaches, and a discussion of the achieved outcomes.

## Methods

### Machine Learning Methods in Bioinformatics

Machine Learning (ML) algorithms have proved to be particularly useful for the analysis of complex big biological data (Olson et al., 2017). For instance ML has been applied to detect epistasis within the human genome (McKinney et al., 2006) suggesting that ML can reveal non-linear behavior in biological systems. In the same direction, more recent deep learning approaches have been profitably exploited to analyze genotype/phenotype associations (Min et al., 2017) as well as to extract relevant information from many data modalities, including text, images, and sounds (Li et al., 2019).

Deep learning methods follow a data-driven approach and are therefore well-designed to detect nonlinear-behaviors, which are relatively common in natural systems (Tang et al., 2019). Networks can vary depending on the number of layers and type of nodes and not all of them perform equally well on different data typology. Convolutional Neural Networks (CNN) are generally applied to recognize objects in a pattern, Recurrent Neural Networks (RNN) to analyze temporal data, but it is not mandatory to use any kind of network only for a specific task. For instance, CNNs were successfully used to predict the enhancer-promoter interactions with DNA sequences (Zhuang et al., 2019) and for accurate clustering of sequences (Aoki and Sakakibara, 2018). RNNs were used instead for predicting transcription factor binding sites (Shen et al., 2018) and to dissect the regulation of mRNA to protein-coding translation (Hill et al., 2018).

Noteworthy, also variational autoencoders (VAEs) showed good performance in capturing biologically relevant feature in gene expression data analysis (Way and Greene, 2017a). VAEs are part of a large branch of deep learning architectures, the so called generative models (Goodfellow, 2016). These architectures are based on an encoding-decoding approach and, differently from the standard autoencoders, they assume a stochasticity in the modelling of the data. The original input matrices of features are compressed in a lower dimensional space, the so called encoding phase, and are reconstructed back in a second step, called decoding phase. Both phases are composed by neural networks. VAEs have seen increasing success in many different applications in the last few years, among the unsupervised methodologies recently developed, and they are widely used in different types of data such as time series, images or gene expressions (Goodfellow, 2016; Goodfellow et al., 2016; Way and Greene, 2017b).

### Tissue Specific Gene Expression Imputation

Data used for the preparation of this article were obtained from the Alzheimer’s Disease Neuroimaging Initiative (ADNI) database (adni.loni.usc.edu). ADNI was launched in 2003 as a public-private partnership led by Michael W. Weiner, MD. The primary goal of ADNI has been to test whether serial Magnetic Resonance Imaging (MRI), Positron Emission Tomography (PET), other biological markers, clinical and neuropsychological assessment can be combined to measure the progression of mild cognitive impairment (MCI) and early Alzheimer’s disease (AD). In the present work, we analyzed the ADNI1-GWAS dataset including gene array genotyping data for 808 samples available on ADNI portal.

Rigorous quality control has been performed. Namely, samples have been checked for sex, missing genotype rates lower than 0.05 and heterozygosity levels *F* < 0.2, while variants with Hardy–Weinberg *p*-value < 1*e* – 10 have been removed. Then, using the tool by W. Rayner^5^ we checked SNPs for strand consistency, allele names, position, Ref/Alt assignments and minor allele frequency (MAF) in comparison to the reference panel. In order to increase the available genetic information, we imputed our data using Sanger Imputation Server^6^ exploiting Eagle2 for phasing (Loh et al., 2016) and Positional Burrows–Wheeler Transform (Durbin, 2014), considering Haplotype Reference Consortium version 1.1 (McCarthy et al., 2016) as reference panel. As a postimputation quality control, we removed variants with info quality level < 0.6. Genotype calls with posterior probability < 0.9 were set to missing. Post-QC imputed data was used to estimate gene expression regulation across the different samples.

**Figure 1:**
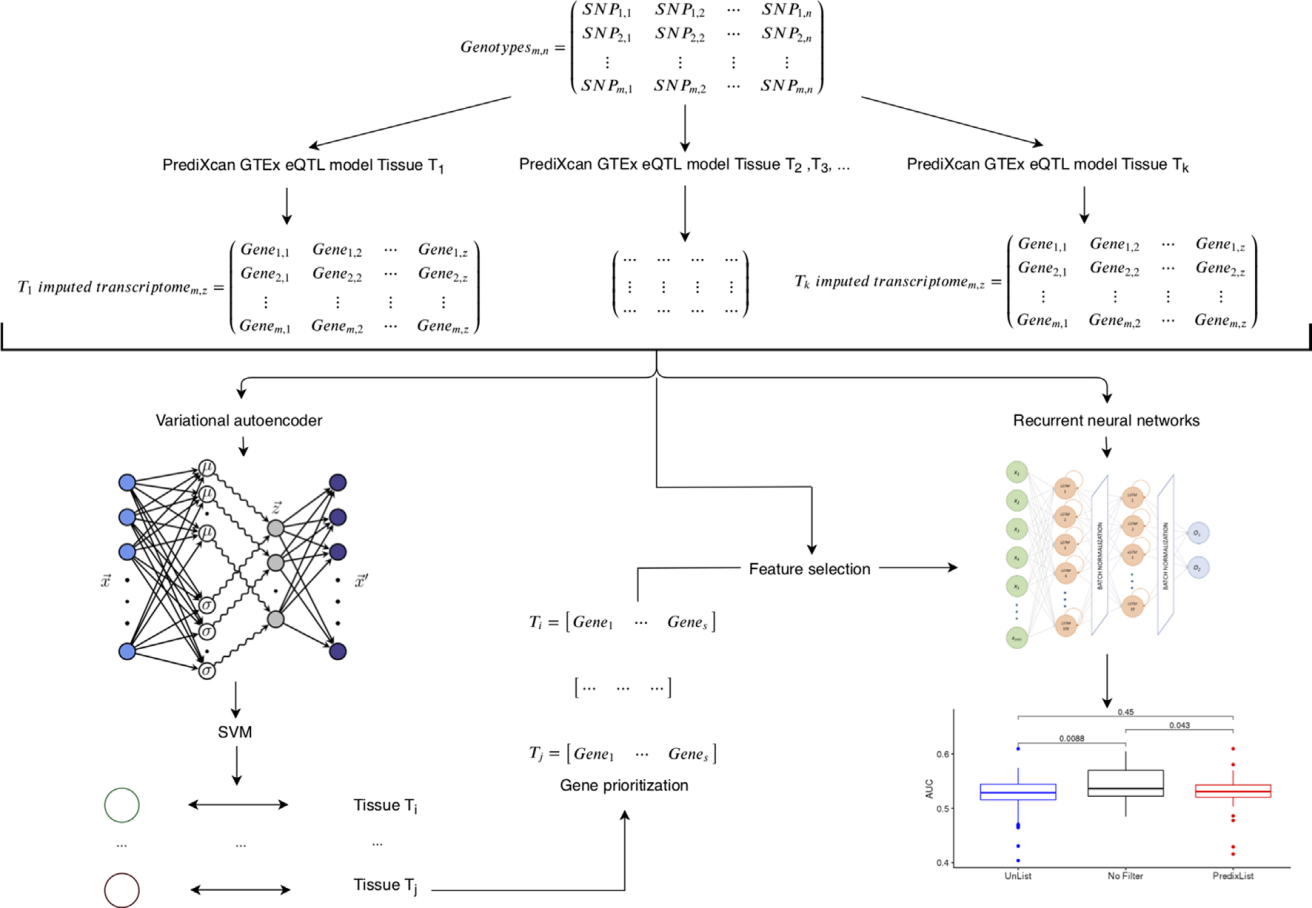
Framework of integrative analysis of multi-tissues expression profiles. Starting from genotyping data (*m* individuals per *n* variants) we imputed tissues specific transcriptomic profiles (for any tissue *T* *_i_*, where *i* = 1‚…‚ *k*) by means of cis-eQTL PrediXcan models trained on GTEx data. Variational autoencoder followed by support vector machine (SVM) latent dimension-tissue match on the imputed gene expression matrices (*m* individuals per *z* genes) is used as a feature selection to identify the most relevant genes per tissue (*T* *_i_* = *gene*_1_‚…‚ *gene* *_s_* where *i* is the *i* *_th_* tissue and *s* in the number of prioritized genes) to provide as input of the recurrent neural network classifier.

In order to predict the genetic component of gene expression, we used PrediXcan that evaluates the aggregate effects of cis-regulatory variants (within 1MB upstream or downstream of genes of interest) on gene expression *via* an elastic net regression method (Gamazon et al., 2015). PediXcan needs a reference dataset in which both genome variation and gene expression levels have been measured to build prediction models for gene expression. We exploited already available models trained on GTEX data^7^ to impute tissues specific transcriptomic profiles in a total of 42 tissues (we excluded sex specific tissues, e.g., prostate, ovary, etc.). The imputed transcriptomic profiles were subsequently analyzed using different machine learning approaches (**Figure 1**). On the one hand, unsupervised machine learning methods were used to analyze data structure, on the other hand, supervised methods were used to test for the presence of “signal” compared to AD related phenotypes.

### Gene Prioritization

Gene prioritization was performed considering as input the predicted transcriptomic matrices from ADNI1-GWAS (excluding sex-specific tissues) resulting in a total of 42 tissues with 808 samples each (42 × 808 = 33, 936 samples overall). We performed an independent analysis involving 528 “cases”, that included people affected by dementia and/or with cognitive dysfunction (AD and MCI) for a total of 528 × 42 = 22, 176 input data, and 280 controls including healthy subjects for a total of 280 × 42 = 11, 760 input data. Each sample was comprised of 24, 203 genes in total.

To identify relevant genes we used variational autoencoders (VAEs) with a single hidden layer with a dimension of 42 units, hence matching the number of tissues. We adapted the code publicly provided by Way and Greene (2017b) to implement our VAE’s architecture. In the encoding phase, the network inputs are the original dataset features representation $\vec{x}$. These are transformed by means of non-linear activation functions in a hidden representation that we denominated $\vec{z}$ and that we assume being characterized by a Gaussian probability density function. In this phase the 2 latent representations of μ and λ of the distribution are learned.

The second part of the architecture that we denoted as the decoder is again built as a neural network. The input this time is the vector $\vec{z}$ i.e. the latent stochastic representation of the input dataset and the output will be the reconstructed representation $\vec{x^{\prime }}$ of the original input vector $\vec{x}$. A representation of the VAE architecture can be seen in **Figure 1**. The loss function of the VAE consists of two parts: the first part being the reconstruction loss (negative log-likelihood) and the second part being the function expressing the Kullback–Leibler (KL) divergence considering the learned hidden distribution and *a priori* Gaussian distribution (Wetzel, 2017).

The first term of the loss function is considered over the encoder distribution of the hidden representation and it “encourages” the decoding phase to correctly reconstruct the input data (Altosaar, 2019). KL divergence is used to enforce the similarity between the distribution of the latent representation and the normal distribution.

We used separate VAEs to encode the gene expression of the cases and healthy classes. Original data include positive (upregulated genes) and negative values (downregulated genes). In order to compute VAE analysis, input data have been scaled between 0 and 1. Noteworthy, different genes can be present in different tissues while VAE pipeline requires an equal number of gene as input, thus NaN (non-existent/Not a Number) values during VAE input preprocessing were set to 0. The input samples were randomly split in training (80%) and test sets (20%) using a stratified approach to maintain the same proportion of samples per tissue. We used the Adam optimizer (Kingma and Ba, 2014) with a learning rate of 0:001 over 75 epochs over the data, rectified linear units during the encoding stage, sigmoid activation during the decoding stage, batch size of 500, and warmup (ĸ) of 1. Hyperparameters were manually selected using a VAE that was not used further in the analysis, to achieve optimal reconstruction performance without overfitting. The entire autoencoding procedure was repeated 75 times separately for the healthy and AD classes in order to study the repeatability of results.

The main goal of the unsupervised analysis was to identify the up or down-regulation of certain genes in specific tissue types in cases and healthy samples. We used a two-step procedure to achieve this association: we identified the tissue(s) encoded in each latent dimension unit of the VAE models, and then we identified the genes most strongly connected to the given latent dimension unit.

In order to identify the tissue(s) encoded in each latent dimension, we used the activations of the hidden layer in the VAEs as an input feature to 42 binary Support Vector Machine (SVM) classifiers, one for each tissue. We trained each SVM classifier to predict whether the input sample to the VAE belonged to a specific tissue relying on the activation value of a single unit from the embedded latent dimension of the VAE. We repeated this tissue-latent unit association procedure for each tissue and each unit in the hidden VAE layer. We performed a 5-fold stratified cross-validation using a linear SVM (*C* = 1 with class weight balance), thus running a total of 5 × 42 × 42 SVM classifiers for each VAE (a 5-fold cross validation procedure, for 42 binary classifiers, for each one of the 42 hidden layer’s unit). We considered a given latent VAE unit to be predictive of a specific tissue type, hence associated with it, if the *F*1 score was greater than 0.8. We found that some hidden units encode more than 1 tissue type.

**Figure 2:**
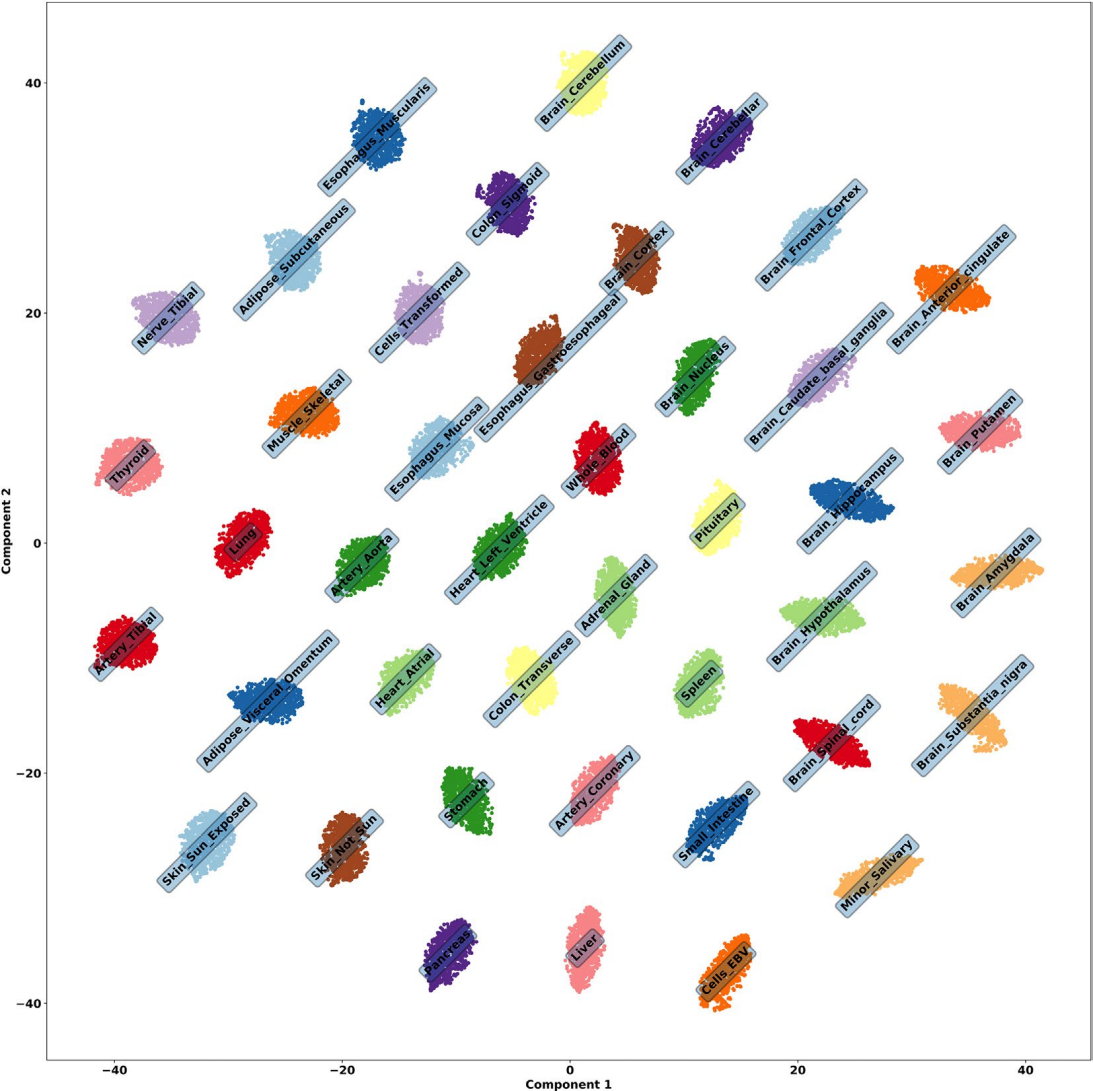
t-SNE embedding of tissue genes, run using the 42 activations on the latent dimension of a VAE to check the embedded structure of all samples. It is obvious that the latent activations are encoding information about each tissue.

It is noteworthy to mention that we tried other unsuccessful approaches. Firstly, we tried to use a single VAE with both cases and controls, trying to find subclusters besides the tissues which cluster very well (see **Figure 2**) in the VAE’s latent dimension as well as in the original data. We also tried to use a single VAE for each tissue in separate. No obvious structures were found when trying to match the results of t-SNE algorithm with all the available phenotypes, including case/control status. Filtering the input for genes within each tissue that show nominal significance for case/control status using standard simple univariate tests did not improve the results. Filtering genes with *R* *^2^* > 0.15 of expression prediction using the same threshold as in Hohman et al.ʹs work (Hohman et al., 2017) did not improve the results as well. In order to understand the features important for classification, we also implemented a saliency map approach. This method is able to detect where the attention of the network (VAEs in our study) is focused (Itti et al., 1998), which can be seen as a sensitivity analysis approach. Saliency maps are generally applied in computer vision but, they can be used in other areas. In our case, the maps were computed on the encoder part of the VAEs and the information extracted is the importance of each gene in the analysis, which is coded as an rgb color code. From this analysis we were not able to identify significative patterns in the input data.

Considering the VAE used in this work, the association of the genes with the latent dimension units can be performed solely relying on the magnitude of the corresponding network weights. Given that each VAE has a single hidden layer, each latent dimension unit is connected directly to every output unit, i.e. reconstructed gene, *via* a linear transformation. Since each reconstructed gene is a summation of the weighted contribution of each latent unit, we could rank the relative importance of the units in the hidden layer relying on the magnitude of the weights. Thus, we selected the 100 most positive and 100 most negative weights for each latent unit encoding a given tissue. This resulted in a set of 100 upregulated and 100 downregulated genes, respectively for each of the trained VAEs. The entire association procedure was performed for the 75 VAEs from healthy and AD samples. We counted the total number of times a given gene was considered up or downregulated by our association procedure and kept it if it appeared more than three times overall. As a result, we produced a list of up or down regulated genes associated with each of the 42 types of tissues. We used this list as an input for pathway enrichment analysis.

In order to perform enrichment analysis, we used Fast Gene Set Enrichment Analysis (FGSEA), a tool developed by Sergushichev et al. (Sergushichev, 2016). The approach implemented by FGSEA deals with quantitative data having inherently directionality like gene expression. The model is based on gene statistic array *S* = *Si*‚…*Sn* where *N* is the number of samples and *Si* > 0 represent over-expression of gene i while *Si* < 0 represent down-expression. The absolute value of *Si* represents a magnitude of the change. The list of gene sets *P* of length m usually contains groups of genes that are commonly regulated in certain biological process. To quantify a co-regulation of genes in a gene set *p* Subramanian et al. (2005) introduced a gene set enrichment score function *sr*(*p*) that uses gene rankings (values of *S*). Given a gene set *p* the more positive is the value of *sr*(*p*) the more enriched the gene set is in positively-regulated genes g with *Sg* > 0, accordingly, negative *sr*(*p*) corresponds to enrichment of negatively regulated genes. To deal with multiple-comparison issues an empirical *p*-value is computed by randomly sampling gene sets of the same size of *p*.

The lists of downregulated and upregulated genes per tissue (referred as *List-unsupervised*) have been considered also as a feature selection step to build prediction models. We also tested other approaches to identify the most relevant genes as considering: I) nominal significantly associated genes from logistic association test between predicted gene expression levels and phenotype status (referred as *List-PrediXcan*), II) nominal associated genes derived by the combination of single tissue-trait association using generalized Berk–Jones test (referred as *List-UTMOST*) obtained with UTMOST tool (Hu et al., 2019).

### Phenotype Prediction Models From Imputed Transcriptomic Matrices

Several supervised analysis techniques were tested in order to understand which one could achieve better results in identifying cases and controls from the transcriptomic profiles: Logistic Regression (LR), Support Vector Machine (SVM), Random Forest (RF) and Deep Learning networks. The latter are known to achieve better results compared to other machine learning methods, especially when the relationships between the observed features is not supposed to be linear (LeCun et al., 2015).

Since we imputed data according to specific tissues, we searched the model that would perform better among them. For this reason, we randomly selected 6 of the 42 tissues (Adipose Subcutaneous, Artery Aorta, Brain Spinal, Colon Transverse, Thyroid, Whole Blood) and trained the models on 600 of the 808 samples from ADNI1-GWAS, considering that the dataset is slightly unbalanced, as it contains more AD samples (528) than controls (280). SVM, RF and LR were not capable of learning how to classify cases and controls, since they assigned the samples only to the majority class. Concerning Deep Learning, the first accomplishment was understanding the appropriate architecture to elaborate transcriptomic data: we tested two Dense Neural Networks (DNN), two CNNs and an RNN.

The first DNN (DNN-1) consisted of 6 layers with respectively 800, 500, 400, 200, 40 and 2 nodes (called neurons). The second DNN (DNN-2) tested consisted of only three layers with 800, 200 and 2 neurons. The first CNN (CNN-1) had 6 layers: a convolutional layer of 10 filters, a convolutional layer of 5 filters after which a dropout regularization was applied, another convolutional layer of 5 filters, a dense layer of 200 neurons with a dropout, and two dense layers of 100 and 2 neurons in the end. The second CNN (CNN-2) was a pure convolutional network of two convolutional layers of 10 and 5 filters, with a dropout regularization applied between them, and a dense layer with 2 neurons as the output layer. The RNN had 3 layers: two Long Short-Term Memory cells (LSTM) with output dimension of 30 and 20 and a final dense layer of 2 neurons.

Looking at the preliminary training results (**Table 3**) we decided to select and optimize the RNN, manually searching the optimal network’s size and then identifying the hyperparameters with the Grid Search algorithm (batch size = 100, epochs = 100). The final architecture consisted of the input and output layers and two hidden LSTM layers of 150 and 10 output dimensions. After every hidden layer a batch normalization was applied to maintain the mean activation close to 0 and the activation standard deviation close to 1. The input layer dimension was equal to the number of genes characterizing the tissue transcriptomic profile, while the output layer was a dense layer of dimension two to make possible the classification of the samples in AD and not-AD.

Considering all the 42 tissues, we had the chance to perform two types of analysis: a tissue-specific analysis and a cross-tissue analysis. In the tissue-specific analysis, we trained models on transcriptomic data specific for each tissue. Therefore, we implemented predictive models that could impute the case/control condition on new transcriptomic data related to the same tissue. The input dimensions of the networks were in the order of thousands, but different for every tissue: the minimum was 2,041 characterizing the Brain Substantia Nigra tissue, and the maximum was reached by the Thyroid tissue with 9,655.

The aim of the cross-tissue analysis was, on the other hand, to observe the similarities between tissues in relationship with the Alzheimer’s disease. Models were trained on each single tissue, taking as input the genes shared by all the 42 tissue transcriptomic profiles (24, 203). The column reporting the information for a gene was filled with zeros if it was not possible to impute the transcriptomic profile of that gene in a specific tissue. Comparing the maximum number of genes imputed for the tissues and the total number of genes identified in all the analysis, it was clear that the new arranged matrices of 24, 203 genes for 808 samples were particularly sparse. The models were then used to impute the case/control condition on tissues different to the one used for the training.

Both in single tissue and cross-tissue analyses all the models were trained on 600 samples from ADNI1-GWAS and the tests were performed on the remaining 208 samples. The network architecture was in all cases the one in **Figure 1**, adjusting the input dimension according to the different analysis. A 10-fold cross validation was applied and models compiled with the Adam optimizer and the binary cross-entropy as the optimization score function. The monitored scores were the accuracy, area under the curve (AUC), precision, recall, and *F*1. The saliency map was applied in the first LSTM layer, therefore we could observe if some samples were more informative than other for the classification purpose. Keras^8^ and Scikit-learn^9^ Python libraries were used, built on top of TensorFlow^10^ to implement the networks.

We then worked on features selection to find groups of genes that were likely to improve the model performance regarding the samples partition in case/control, both in the single-tissue and cross-tissues approaches. The identification of such groups in single-tissue analysis can bring to the determination of tissue specific markers, on the other hand in the cross-tissues section we could focus on the set of genes that explained the relationship between tissues. We used three different filter lists: *List-unsupervised*, *List-PrediXcan* and *List-UTMOST* (see **Supplementary Materials Section 3**). Using these lists the input dimensions for all the tissues decreased: the number of unique genes identified by the List-unsupervised was 2,016, 4,984 with List-PrediXcan. List-UTMOST (649 genes) was used only in the cross-tissue analysis as it doesn’t provide tissue-specific information.

All the steps described above (except the architecture selection and saliency map) were also performed considering Cognitive Decline over time rather than diagnosis at screening. This dataset consisted of 528 samples (some samples did not have this information), 281 controls and 247 cases. Cognitive Decline has been calculated by considering the difference between the Mini-Mental State Examination (MMSE) score 4 years after recruitment and the MMSE score at recruitment. Then, regardless of the original recruitment diagnosis, we classified the samples into two groups: one group showing no cognitive decline (difference equal or greater than 0) and the other showing a cognitive decline (difference minor than 0). The genes imputed for each tissue were therefore the same in ADNI1-GWAS dataset and Cognitive Decline dataset. To consider the effect of AD related variables, we also performed the same analyses by stratifying by sex and early/late onset for dementia and AD [using 65 years of age as a cutoff (Roberts and Petersen, 2014)] as well as for carrier and noncarrier of APOE ∊4 isoform.

## Results

We predicted the genetic component of gene expression across 42 non-sex-specific tissues for all the samples included in ADNI1-GWAS dataset. We exploited tissues specific eQTL models available on precictDB^11^ and used PrediXcan tool^12^ to derive tissue specific matrices representing individual levels of the genetic component of gene expression. The gene levels obtained by these sample matrices represent transcriptomic profiles based on eQTL across tissues in the analyzed dataset.

In the present work the matrices of imputed expression were analyzed using several machine learning strategies to identify potential tissue specific transcriptomic profiles associated with cognitive decline in Alzheimer’s.

### Gene-Based Results Per Tissue

We runned t-SNE (Maaten and Hinton, 2008) using the 42 activations on each latent dimension of a VAE to check the embedded structure of all samples, whose result can be seen in **Figure 2**. Although interpretations of Euclidean distances between points in a t-SNE plot is not straightforward (Wattenberg et al., 2016), it is clear from the clusters that information about tissues are being encoded. Indeed, we were able to identify associations between latent dimensions of VAE and tissue.

The evaluation of the weights associated with the latent dimension (see *Methods*) allow us to rank gene importance per tissue considering case/control status. **Table 1** shows the most upregulated and downregulated genes from Brain Nucleus. Check **Supplementary Table S1** for complete information over all 42 tissues.

**Table 1:** Most upregulated and downregulated genes from the brain nucleus.

	Downregulated		Upregulated	
	AD-MCI	CTR	AD-MCI	CTR
**Brain nucleus**	ENSG00000230850.3	ABHD14A	ENSAP2	AL356475.1
	GMPR2	ATP2B4	KLF1	F2
	C1QC	BDKRB2	EEF1A1P19	NRIP2
	SUN3	C1QC	RP5-1068B5.3	RP11-704J17.5
	RP11-662J14.1	PXN	RP11-321A17.3	RP11-321A17.3

The saliency map implementation returned not useful information. If taken individually, genes don’t have much impact: it is evident also with this result that the AD phenotype is due to a combination of many genes and environmental factors.

In order to investigate the presence of specific gene expression regulation associated with case/control status we considered the lists of tissue-specific up and down regulated genes derived by VAE analysis. Additionally, for each tissue we considered the genes that were differentially regulated in cases but not in controls, that is representing a disease-specific signature. The enrichment analysis have been performed considering Gene ontology^13^, KEGG^14^ and reactome^15^ and pathway databases (Croft et al., 2013; Kanehisa et al., 2016). Complete enrichment analysis results are available as supplementary files (see **Supplementary Materials Section 1**) while significant enrichment tissues specific pathways after FDR correction are shown in **Table 2**.

**Table 2:** Significant tissue-pathways enrichment analysis using Reactome database.

Tissue	Pathway	pval	padj	ES	NES	Genes	
Colon sigmoid	Immune system	3.8E–04	1.2E–02	–5.4E–01	–2.3E+00	CAP1 RASGRP4 RASGRP4 YES1 SIGLEC8 CD47 SELL CALM1	FBXO21 CLEC7A CLEC7A SEC61A1 IL13 HLA-DPB1 KIF11
Brain nucleus	Generic transcription pathway	3.0E–03	1.8E–02	7.2E–01	2.1E+00	ZNF688 ZKSCAN8 ZNF445	RRAGC ZNF697 CASP6
Brain nucleus	RNA polymerase II transcription	3.0E–03	1.8E–02	7.2E–01	2.1E+00	ZNF688 ZKSCAN8 ZNF445	RRAGC ZNF697 CASP6
Brain nucleus	Gene expression (transcription)	3.0E–03	1.8E–02	7.2E–01	2.1E+00	ZNF688 ZKSCAN8 ZNF445	RRAGC ZNF697 CASP6
Colon sigmoid	Adaptive immune system	3.0Ev03	3.3E–02	–6.1E–01	–2.1E+00	FBXO21 SEC61A1 HLA-DPB1 KIF11	YES1 SIGLEC8 SELL CALM1
Colon sigmoid	Innate immune system	2.5E–03	3.3E–02	–6.5E–01	–2.0E+00	CAP1 CLEC7A CD47 CALM1	RASGRP4 YES1 SELL

Interestingly enrichment analysis shows the presence of tissue specific signal in a specific brain tissue (i.e., brain nucleus) concerning pathways involved in gene expression regulation and in immune-related pathways in colon (**Figure S2**). The most significant alterations in brain pathways concern the brain nucleus accumbens (basal ganglia) region. Interestingly, this region has been found to be associated with AD (Nie et al., 2017; Nobili et al., 2017; Li et al., 2018). Instead, the detected downregulation of immune system pathways in cases in comparison to controls could indicate a higher level of inflammation in dementia. This is in line with the association observed between inflammatory bowel diseases and AD (McCaulley and Grush, 2015; Sochocka et al., 2019). Given the pivotal role of APOE (Liu et al., 2013) in AD a specific evaluation was performed to evaluate the effect of APOE related genes.

APOE gene expression is not predicted by gene expression imputation GTEx based models, due to the absence of eQTL explaining a relevant fraction of APOE expression level. However, AD susceptibility due to APOE isoforms (∫2, ∫3 and ∫4), which are well known to confer a different risk for AD depending on the presence of missense coding variants, are associated with APOE gene functionality and can be independent from the genetic component of gene expression regulation. We investigated if other genes directly interacting with APOE, according to string functional database^16^, have a significant association in our analysis (see **Supplementary Materials Section S3**).

One of the 11 genes identified, namely *APOC2* (Shao et al., 2018), is among the top differentially regulated genes from variational autoencoder gene prioritization list in brain putamen, an area of the brain associated with AD (Coupé et al., 2019). Interestingly, the same gene is also the only one (among the 11 APOE interacting genes) significantly associated with AD according to a transcription wide association analysis performed according to a GWAS on AD in UK Biobank dataset (Marioni et al., 2018) and public available on TWAS hub^17^. This suggests a potential role for *APOC2* associated with the gene expression regulation and, interestingly, a recent work showed that the methylation profile in such a gene (which in turn affect gene expression) is associated with AD (Shao et al., 2018).

### Tissue-Specific and Cross-Tissues Classification

To understand which network performs better on different tissues, we tested five models on six sample tissues. In **Table 3**, accuracy and AUC obtained during their preliminary 10 cross-validation training on 600 of 808 samples are reported: although all methods could perform well at least on one tissue during the training, in that phase only the RNN was capable of reaching an accuracy higher than 90% for all of them. Therefore we decided to optimize the RNN and obtained the network structure described in *Phenotype Prediction Models From Imputed Transcriptomic Matrices*, which was then applied for the single-tissue and cross-tissue analysis on ADNI1-GWAS and Cognitive Decline dataset.

**Table 3:** Preliminary networks training performance on six sample tissues: accuracy (Acc) and area under the curve (AUC).

**Network**	Adipose subcutaneous		Artery aorta		Brain spinal		Colon transverse		Thyroid		Whole blood	
	Acc	AUC	Acc	AUC	Acc	AUC	Acc	AUC	Acc	AUC	Acc	AUC
DNN-1	37.50	0.513	37.33	0.503	64.00	0.538	87.67	0.862	64.50	0.503	39.83	0.516
DNN-2	64.50	0.5	64.50	0.5	90.17	0.892	64.50	0.5	64.50	0.5	64.50	0.5
CNN-1	63.00	0.5	76.92	0.721	77.50	0.901	78.50	0.770	64.08	0.5		0.491
CNN-2	95.83	0.948	64.50	0.5	94.83	0.943	64.50	0.5	96.00	0.95	95.67	0.947
RNN	96.17	0.953	95.67	0.951	94.67	0.942	95.33	0.946	95.33	0.946	94.67	0.939

Without the feature selection, we observed a great performance during the training in terms of AUC, accuracy, precision, recall and *F*1 scores (see **Supplementary Materials Section 2**) on both datasets. On test set (composed of 208 samples for tissue for ADNI1-GWAS and 128 for Cognitive Decline) the metrics reached values below expectations, with AUCs near 0:5 especially for ADNI1-GWAS.

**Figure 3:**
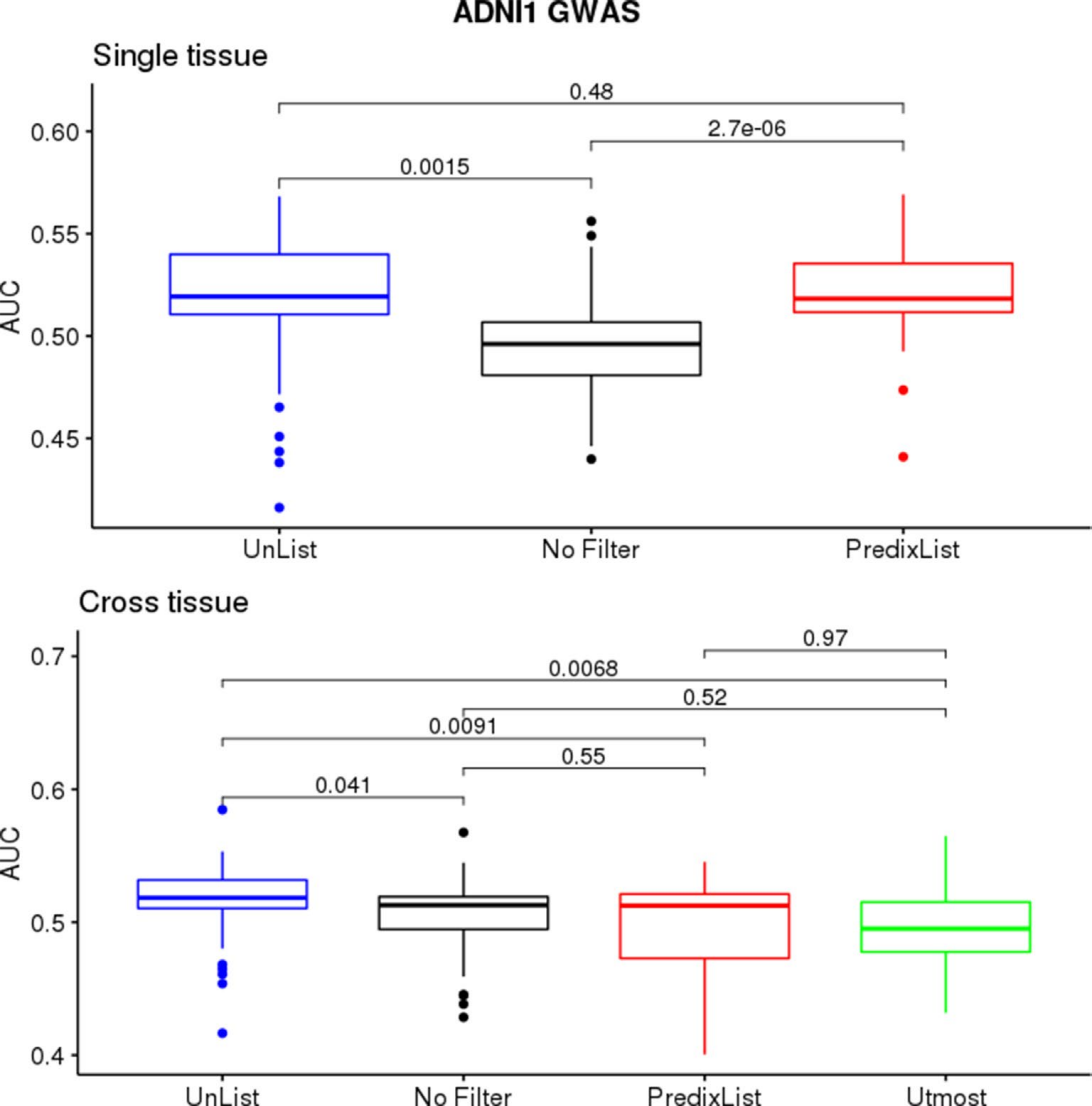
ADNI1-GWAS feature selection evaluation. The single-tissue models (top panel) significantly improved their ability to classify case/control condition thanks to both *List-unsupervised* (blue) and *List-PrediXcan* (red) compared to the no filtering approach (black). On cross-tissue models (bottom panel), where there is also the performance with the *List-UTMOST* (green), the improvement was less evident.

On ADNI1-GWAS (**Figure 3**), models trained for single-tissue analysis improved their AUCs thanks to the *List-unsupervised* and *List-PrediXcan* feature selection: when the AUCs were below 0:5, the filters application returned a score above the threshold for at least one list. We did not observe a major impact of a list in this phase but the *t*-test confirmed a significant improvement compared to the no filter approach (*p*-value = 0.001474 for *List-unsupervised* and *p*-value = 2.693*e* – 06 for *List-PrediXcan*). Models trained for the cross-tissue analysis instead had a less evident improvement with the lists filter: only the List *unsupervised* returned a slightly significant improvement (*p*-value = 0.04084). *List-UTMOST* did not give any improvement and, as we could not use it on single-tissue models, we decided not to further analyze it.

**Figure 4:**
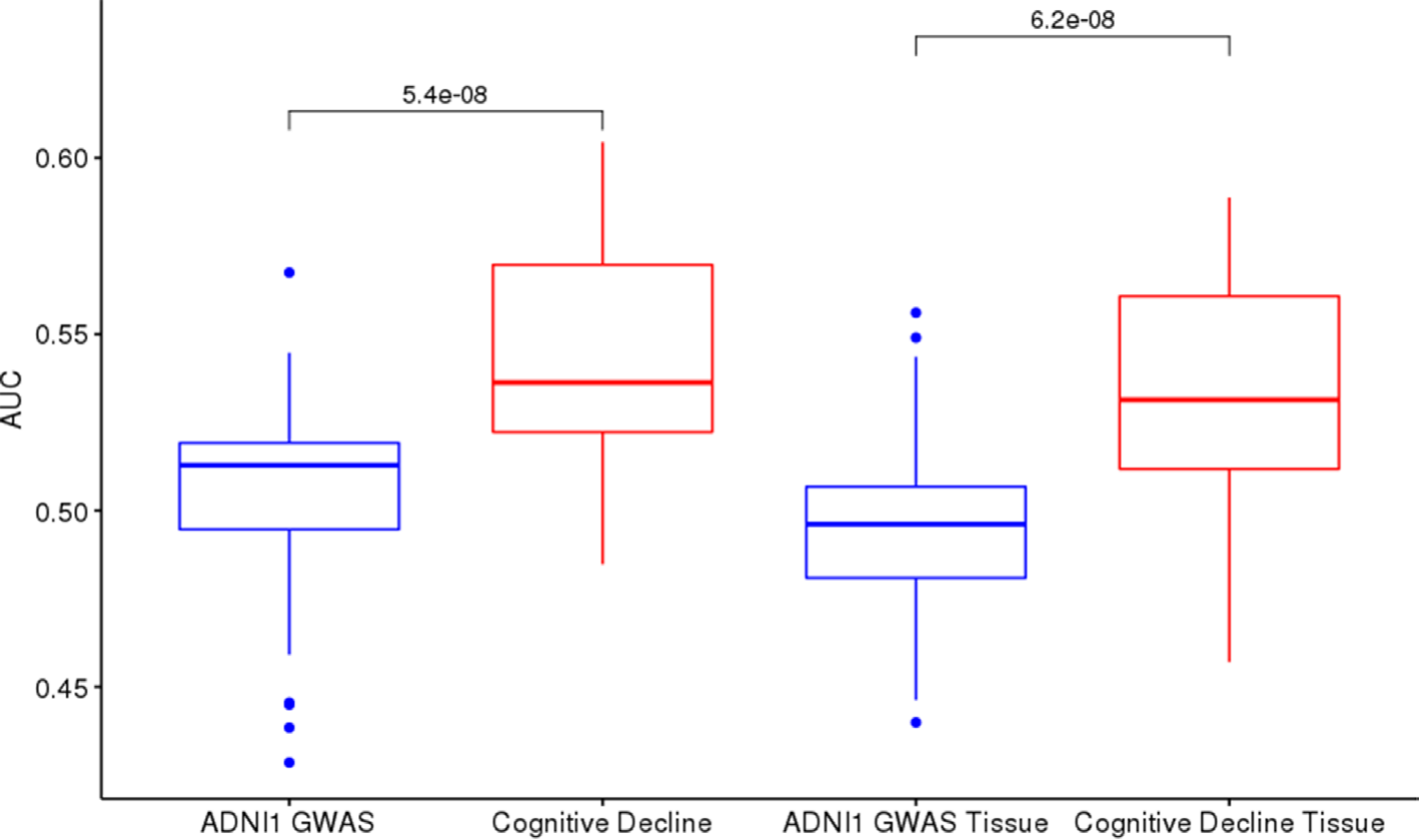
ADNI1-GWAS and Cognitive Decline comparison: Cognitive Decline (red boxes) returns higher AUCs on test sets than ADNI1-GWAS (blue boxes) both in cross-tissue models (left) and in single-tissue models (right).

Cognitive Decline models performed better than ADNI1-GWAS, both in single-tissue and cross-tissue analysis (**Figure 4**). The lists application on Cognitive Decline models also led to an improvement for tissues with borderline or below the threshold performance (**Figure S5**), reaching AUCs between 0:51 and 0:6. On cross-tissue models we obtained a significant *p*-value = 0.008766 for List-unsupervised and *p*-value = 0.04346 for List-PrediXcan.

**Figure 5:**
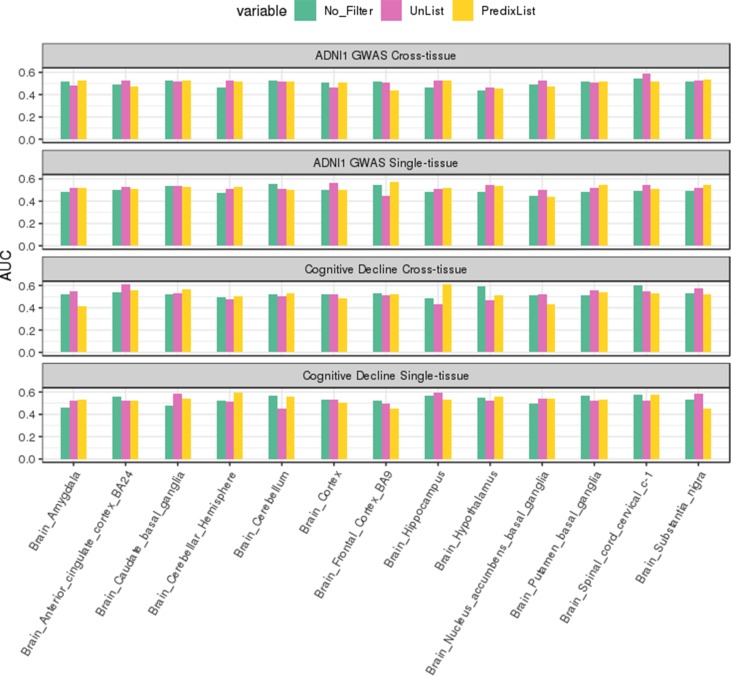
Brain tissues analysis. In green the AUCs on test sets for the no filter application are reported, in red for *List-unsupervised* and in yellow for *List-PrediXcan*. The top two panels report respectively the cross-tissue and single-tissue models performance on ADNI1-GWAS dataset, the third and fourth panels on Cognitive Decline. In both datasets, feature filtering improved the classification in almost all the Brain tissues.

Comparing the two lists on ADNI1-GWAS, List-unsupervised showed the bigger improvement on cross-tissue models: the *t*-test returned a *p*-value of 0:009123, but on single-tissue the difference was not significant. Also on Cognitive Decline we observed a slightly major impact of List-unsupervised both for the single-tissue and cross-tissue models. In **Figure 5**, a focus on the improvement achieved with the filter on the Brain tissue is shown in both datasets, in **Figure S4** the evaluation for all tissues is shown.

**Figure 6:**
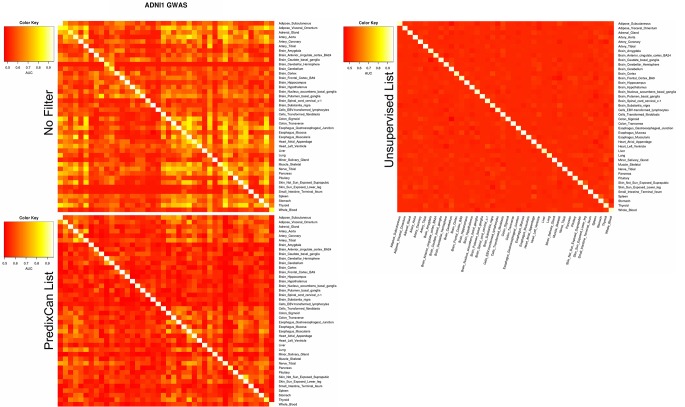
ADNI1-GWAS cross-tissues performance. By column we can observe how much a model trained on a tissue is able to recognize without mistakes (AUC) AD/non-AD subjects from data related to different tissues. On the diagonal for each tissue the AUC obtained for that model during the training is reported. The top panel reports the cross-tissue results without any filter application, the middle panel when using *List-PrediXcan* and the bottom using *List-unsupervised*.

**Figure 6** reports, by columns, the AUC achieved by ADNI1-GWAS cross-tissue models when they were applied on other tissues from the same dataset. The top heatmap describes the relationships between tissue when no filter is applied: we could observe that models trained on Brain tissues, if they were able to correctly identify the AD subjects on a non-Brain tissue, they could do the same on all the other non-Brain tissues. Instead, models trained on non-Brain tissue could identify AD-MCI/CTRL subjects only on a subset of tissues. We performed the same analysis on ADNI1-GWAS models filtered by List-PrediXcan and List-unsupervised, respectively the middle and bottom heatmaps of **Figure 6**: List-unsupervised removed all the information of cross-tissue relationships, when instead List-PrediXcan mitigate them, pointing out the non-Brain models relationships.

We also tested the stratification for sex, age, APOE effect, and AD condition on ADNI1-GWAS dataset for single-tissue and cross-tissue analysis. It returned no considerable variation in the performance. The saliency map application was also not informative: each sample has the same importance. Lastly, we performed the filter analyses on Cognitive Decline, pointing out the same results (**Figure S6**).

## Discussion

In the present work we dissected the tissue specific genetic component of gene expression in association to AD related cognitive decline. Our analysis consisted on the imputation of tissue specific gene expression profiles by using a TWAS-like approach (Mancuso et al., 2017). However, contrary to the standard TWAS analysis, we did not specifically focus on univariate analysis (e.g., gene association based on logistic or linear regression). Instead, we dissected individual transcriptomic levels using different machine learning approaches. We believe that our approach can be of particular interest since is capable of capturing data structure and non-linear behaviour in the system. In fact, it is well known that gene expression levels are not independent, since many genes are actually correlated in terms of regulation (Michalopoulos et al., 2012) and functionality, which means that also epistatic interactions can play a major role in the regulation of biochemical pathways (Sameith et al., 2015).

Interestingly, we observed that a combination of unsupervised and supervised machine learning methods on matrices of predicted expression provided complementary information that can be integrated in order to get new insights in gene expression regulation. On one hand, the VAE combined with enrichment analysis suggests the presence of a specific biochemical pathways alteration in dementia occurring in a specific brain area and in the gut. The identified alteration occur in brain nucleus, a brain region found to be associated with AD by several studies (Cho et al., 2014; Wang et al., 2014; Kuhn et al., 2015; Liu et al., 2015).

This alteration seems to be related to the regulation of gene expression and 436 therefore possibly associated to tissue-specific pathways regulation. Instead, the enriched pathways in gut are related to immune systems and noteworthy, it is well established that immune system dysfunctions can lead to a greater increase of inflammation in AD (Serpente et al., 2014; Heppner et al., 2015; Le Page et al., 2018). These results suggest that our analytical approach can identify relevant biological alterations occurring in AD. Noteworthy, enrichment analysis identified alteration in biological pathway specifically in a brain area and gut, which is in line with the presence of a gut-brain axis dysfunction in AD. Indeed, several researchers pointed out that brain-gut axis can be associated with many neurological disorders (Giau et al., 2018; La Rosa et al., 2018).

In the present work, APOE genotype has not been directly included as covariate in prediction models since our aim was to identify other genetic factors that can explain part of the missing heritability on the established polygenic component in AD (Escott-Price et al., 2017; Tosto et al., 2017). However, APOE is expected to be by far the most influencing risk factor for late onset AD. Though estimation of APOE contribution on the heritability component of AD is still not well defined, ranging from 10% to 28% of the overall genetic heritability (Van Cauwenberghe et al., 2016; Stocker et al., 2018). Moreover, in the present work, gene-expression derived genetic signals neglect not-eQTL effects and therefore we have limited analytical power. This justifies the relatively low AUC values in comparison to other prediction models in AD, including the complete genome-wide polygenic signal and using APOE as a covariate (Escott-Price et al., 2017; Tosto et al., 2017). Our aim was indeed to test whether or not there is a genetic signal associated with AD that could be apportioned to tissue specific gene-expression regulation rather than identify a prediction model. It is also known that genetics is just one of the component involved in AD susceptibility and therefore the use of multimodal data (e.g., imaging data, clinical features, metabolomic, and environmental factors) should be taken into account in order to build a reliable classifier in term of translational application (Sapkota et al., 2018). Despite that, our classification models were still capable of finding a signal between cases and controls (overall AUC > 0:5) suggesting that part of the genetic signal in AD related dementia can be associated with tissue-specific gene expression regulation. Moreover, we observed that feature selection can play a major role in the performance of deep learning networks classification.

We are aware that our work presents some limitations. We performed a genetic association with dementia by considering ADNI data evaluating the solely genetic component of gene expression, which neglects other potential genetics effect not related to gene-expression regulation. Our models are also limited by the current version of GTEx data, which has a relatively small size, therefore it is expected that over time new models will optimize eQTL estimation leading to more precise analyses of the genetic component of gene expression. We also focused on non-sex specific tissues, since we wanted to study general potential alterations not involving sex-specific organs, but this could also be a limitation given the different prevalence of AD in females and males (Mazure and Swendsen, 2016).

## Conclusion

In the present work, we performed an analysis of the predicted genetic component of gene expression in ADNI1-GWAS dataset in association with AD cognitive decline. We dissected the predicted tissue specific gene expression by means of different supervised and unsupervised machine learning approaches. Our results suggest that a framework including unsupervised and supervised methods in data-analysis can provide complementary information and thus leading to better insights into the underlying system.

In particular, variational autoencoder pre-processing of input data proved to be efficient for features selection prior to the implementation of deep learning classification models. However, the limited AUC prediction performance of the developed models suggests that the evaluation of the solely genetic component of gene expression by exploiting up to date available GTEx models is currently under-powered in comparison to genome-wide polygenic risk score modeling.

This is not surprising since we are neglecting the effect of non-eQTL variants. On the other hand, we can disclose tissue specific effects and reveal potential biological mechanisms associated with a given phenotype. In this regard, our analysis showed that brain tissues are more associated with dementia status and that inflammatory processes in brain-gut axis can play a role in AD.

## Author’s Note

Data used in preparing this article were obtained from the Alzheimer’s Disease Neuroimaging Initiative (ADNI) database (adni.loni.usc.edu). As such, many investigators within the ADNI contributed to the design and implementation of ADNI and/or provided data but did not participate in analysis or writing of this report. A complete listing of ADNI investigators can be found at: http://adni.loni.usc.edu/wp-content/uploads/how_to_apply/ADNI_Acknowledgement_List.pdf

## Data Availability

Publicly available datasets were analyzed in this study. This data can be found here: http://adni.loni.usc.edu/. Supplementary data and the code used in this work is available at https://github.com/imerelli/DeepNeuro.

## Author Contributions

CM, TA and VG equally contributed to the work. They conceived the idea and developed the algorithms. OB, GD, and SS contributed to data analysis. PL and IM supervised the whole study. All authors contributed to final revision of the manuscript.

## Conflict of Interest Statement

The authors declare that the research was conducted in the absence of any commercial or financial relationships that could be construed as a potential conflict of interest.

## Footnotes

1
https://www.ncbi.nlm.nih.gov/gap

2
https://www.ncbi.nlm.nih.gov/geo/

3
https://gtexportal.org/home/index.html

4
https://www.encodeproject.org/

5
http://www.well.ox.ac.uk/wrayner/tools

6
https://imputation.sanger.ac.uk/

7
https://gtexportal.org/home

8
https://keras.io/

9
https://scikit-learn.org/stable/

10
https://www.tensorflow.org/

11
http://predictdb.org/

12
https://github.com/hakyimlab/PrediXcan

13
http://geneontology.org/

14
https://www.genome.jp/kegg/pathway.html

15
https://reactome.org/

16
https://string-db.org/cgi/network.pl

17
http://twas-hub.org/
