# Supplementary material for: Integration of Machine Learning Methods to Dissect Genetically Imputed Transcriptomic Profiles in Alzheimer’s Disease
Source: Front Genet. 2019 Sep 3;10:726. doi: 10.3389/fgene.2019.00726 (PMC6735530; doi:10.3389/fgene.2019.00726)
Supplement: Supplementary file 1 [file DataSheet_1.pdf]

Supplementary Material: Integration of machine learning methods to dissect genetically imputed transcriptomic profiles in Alzheimer's Disease

1 MOST UPREGULATED AND DOWNREGULATED GENES

Table S1: Most upregulated and downregulated genes over all 42 tissues, both for patients with Alzheimer's Disease or Mild Cognitive Impairment and healthy people

	Downregulated		Upregulated	
	**AD-MCI**	**CTRL**	**AD-MCI**	**CTRL**
**Adipose Subcutaneous**	ENSG00000230850.3	FAM86A	MAP2K7	DCUN1D5
	VWA3B	C19orf80	STK4-AS1	TMEM200B
	C1QC	KIF11	RP11-163E9.1	SPATA8
	RP11-363E6.4	VWA3B	POC1A	POC1A
	RPL13AP17	CEP135	RP11-138I1.3	DGKZP1
**Adipose Visceral Omentum**	ENSG00000230850.3	ATP2B4	RP11-503N18.5	RP11-279O9.4
	C1QC	RNF115	RP11-546K22.3	RPS27
	CTD-2319I12.2	PTPLAD1	RP11-279O9.4	MIEN1
	CTAGE7P	GTF2IRD1	GOLGA8I	KRBOX1
	COL4A4	GPALPP1	ISY1	DCD
**Adrenal Gland**	ENSG00000230850.3	KRBOX1	CTAGE7P	CTAGE7P
	C1QC	ATP2B4	AC013439.4	ARIH1
	SUN3	LINC00537	SVOP	CTC-756D1.2
	GMPR2	ATP6V1H	RP11-583F2.1	SVOP
	CTD-3224K15.2	ANKH	ARIH1	NEFM
**Artery Aorta**	ENSG00000230850.3	NPAS4	CTD-2291D10.3	SCAF8
	CTD-2319I12.2	RGMA	SPINK13	SPINK13
	VWA3B	ADAM20P1	SCAF8	CTD-2291D10.3
	CCDC149	C14orf159	SENP8	SENP8
	C1QC	RNF115	EEF1A1P22	ZNF777
**Artery Coronary**	ENSG00000230850.3	CABLES1	CEP57L1	CEP57L1
	CTD-3224K15.2	CTBP2	HIST1H3D	ADM
	GMPR2	GPR55	VWA3B	RP11-195B21.3
	TPP1	TDH	ADM	VWA3B
	C1QC	YWHAE	RP11-195B21.3	RP11-775C24.3
**Artery Tibial**	ENSG00000230850.3	VWA3B	RPL13AP17	RP11-165H4.2
	RP11-613F22.6	RNF115	RP11-165H4.2	DYNC1I2P1
	SHISA5	RBFOX3	NSRP1	RPL13AP17
	CTD-2291D10.3	CEP135	CSPG4P13	SEMA3G

	WDR54	GPX2	DYNC1I2P1	SDHAF2
**Brain Amygdala**	ENSG00000230850.3	KRBOX1	SUN3	SUN3
	C1QC	AC018804.7	MKLN1	FBXL8
	CTD-3224K15.2	ZNF469	TMSB4XP1	RCOR2
	CNN2P8	SEMA3C	LHX2	TCF24
	CCDC149	GPALPP1	RP11-253E3.1	CTD-2528L19.3
**Brain Anterior cingulate**	ENSG00000230850.3	ADORA2BP	RP11-662J14.1	RP11-662J14.1
	CTD-2319I12.2	COL4A1	RP11-175B9.2	RP11-175B9.2
	GMPR2	GTF2A1	PNISR	PNISR
	RP4-669L17.10	RASL10A	RB1	PDLIM7
	CTD-2031P19.4	RP11-330M2.4	RP11-446H18.6	MTRNR2L12
**Brain Caudate basal ganglia**	ENSG00000230850.3	TMEM104	RP11-602N24.3	RP11-602N24.3
	RP11-662J14.1	MKRN1	ZHX2	CTD-2319I12.2
	CTD-3224K15.2	SETD3	GTF2A1	PCDHB5
	RP4-669L17.10	ADORA2BP	TCF24	RP11-330M2.4
	GMPR2	ATP6V1H	GRHL3	KIF11
**Brain Cerebellar**	ENSG00000230850.3	ADORA2BP	OR4N3P	OR4N3P
	RP11-662J14.1	AC018804.7	RP11-466F5.10	RP1-90G24.11
	C1QC	PTPLAD1	AC018865.5	AC018865.5
	GMPR2	ZNF192P2	RP11-700N1.1	RP11-466F5.10
	VWA3B	GBGT1	SBDS	SBDS
**Brain Cerebellum**	RP11-662J14.1	UAP1	FKSG63	RP11-255J3.3
	ENSG00000230850.3	C1QC	RP11-255J3.3	FAM104A
	RP11-175B9.2	CCDC149	ATP2B4	SNCAIP
	RP4-669L17.10	GPR55	CORO1B	SETD3
	VWA3B	HCG11	UBE4B	RP11-567C2.1
**Brain Cortex**	RP11-662J14.1	ADORA2BP	U3	CABLES1
	RP11-175B9.2	MYBPC3	PPM1D	PXN
	ENSG00000230850.3	LYSMD2	PXN	U3
	GMPR2	CCDC149	RP11-768G7.1	VWA3B
	C1QC	PTPLAD1	EMBP1	CNNM3
**Brain Frontal Cortex**	RP11-662J14.1	TMEM104	RP4-669L17.10	CDCA5
	GMPR2	S100A2	CTD-2031P19.4	CHRNA1
	ENSG00000230850.3	SETD3	RPL39P40	RP11-651P23.5
	RP11-175B9.2	MYH11	RPS3AP47	RPL39P40
	PNISR	KRTAP5-7	RP11-79E3.2	RPS3AP47
**Brain Hippocampus**	ENSG00000230850.3	GTF2A1	YWHAZP5	YWHAZP5
	C1QC	PXN	RPL24P2	KRT8P13
	RP11-662J14.1	SNAPC2	HRASLS2	RP5-1007H16.1
	GMPR2	PTPLAD1	LINC00488	TLDC2
	CTD-3224K15.2	HIST1H3D	FBXL15	LINC00606
**Brain Hypothalamus**	RP11-662J14.1	ADORA2BP	SLA2	TPRG1-AS1
	ENSG00000230850.3	KRBOX1	CNN2P8	SLA2
	C1QC	GTF2A1	RP1-212P9.2	AC093159.1
	CTD-3224K15.2	FAM27E3	RP11-391M20.1	RP4-580N22.2

	GMPR2	NHLRC1	RP11-168L22.2	RP11-391M20.1
**Brain Nucleus**	ENSG00000230850.3	ABHD14A	ENSAP2	AL356475.1
	GMPR2	ATP2B4	KLF1	F2
	C1QC	BDKRB2	EEF1A1P19	NRIP2
	SUN3	C1QC	RP5-1068B5.3	RP11-704J17.5
	RP11-662J14.1	PXN	RP11-321A17.3	RP11-321A17.3
**Brain Putamen**	ENSG00000230850.3	PXN	CNN2P8	KRBOX1
	GMPR2	C1QC	ST6GALNAC5	RAE1
	RP11-662J14.1	PTPLAD1	RP11-138I1.3	ITGA3
	SUN3	AJAP1	FOXD4L6	RP11-1167A19.6
	TPP1	GTF2A1	CCR10	CCR10
**Brain Spinal cord**	ENSG00000230850.3	COL4A1	GMPR2	ATP2B4
	RP11-662J14.1	KRBOX1	C1QC	GMPR2
	RP11-175B9.2	SPON2	NPM1P6	CEP135
	CTD-3224K15.2	BTG1	CEP135	RP4-580N22.2
	FTL	KDM8	ATP2B4	RP11-458N5.1
**Brain Substantia nigra**	RP11-662J14.1	TMEM104	CTD-3224K15.2	CTD-3224K15.2
	ENSG00000230850.3	DEXI	RP5-857K21.7	EMC3
	RP11-175B9.2	CHMP5	NFATC1	NEK8
	PNISR	SLC7A6	FTL	FTL
	VWA3B	KRBOX1	HNF4G	MBLAC2
**Cells EBV**	C1QC	SETD3	ITM2BP1	F13A1
	CTD-3224K15.2	ENSG00000230850.3	EDC3	RP11-458I7.1
	CTD-2319I12.2	ADORA2BP	CASZ1	RP11-564D11.3
	ENSG00000230850.3	CCDC149	RANP6	EME1
	TPP1	GPALPP1	RP11-856M7.6	PALB2
**Cells Transformed**	C1QC	TMEM104	AC017002.1	AC017002.1
	GMPR2	ATP2B4	WTAPP1	WTAPP1
	CTAGE7P	ADORA2BP	ENSG00000230850.3	COL4A1
	CTD-2319I12.2	HIST1H3D	NSUN3	ENSG00000230850.3
	CTD-3224K15.2	HMGXB4	PRMT10	RP1-124C6.1
**Colon Sigmoid**	ENSG00000230850.3	C14orf159	CTD-2231H16.1	ADORA2BP
	CTD-2319I12.2	PEX13	DVL2	ATP6V1H
	C1QC	RPS27	ABCC6P2	DVL2
	TPP1	AC110619.1	EIF3J-AS1	CENPA
	SUN3	PPP1R3B	RBBP4P1	STMN4
**Colon Transverse**	ENSG00000230850.3	AC018804.7	APOBEC1	CTD-2589M5.5
	RP11-662J14.1	PTPLAD1	HMGB1P24	GPR68
	CTD-2319I12.2	COL4A1	CTD-2589M5.5	GPR88
	C1QC	HIST1H3D	GPR68	RP11-307L3.2
	GTF2A1	LYSMD2	RP11-93K22.13	CTA-246H3.12
**Esophagus Gastroesophageal**	ENSG00000230850.3	TMEM104	CTD-2319I12.2	RP11-533E19.3
	DVL2	VWA3B	KIF11	CTD-2319I12.2
	SUN3	ARGLU1	RP11-438N16.2	PCDHGB7
	C1QC	GTF2A1	GDAP1L1	CTC-559E9.10

	ATP6V1H	PTPLAD1	ADH5P4	KIF11
**Esophagus Mucosa**	C1QC	ADORA2BP	ENSG00000230850.3	ENSG00000230850.3
	ATF4P4	CCDC149	MRPL37P1	MRPL37P1
	VWA3B	HHATL	GAPDHP71	RPL37A
	NEK8	NCF4	RPL37A	RP11-56L13.1
	CTD-2319I12.2	MYBPC3	RP11-498M15.1	ROMO1
**Esophagus Muscularis**	ENSG00000230850.3	MAFK	RP11-613F22.6	RP11-613F22.6
	VWA3B	CALM1	FLJ00273	FLJ00273
	KIF11	TMEM104	Z95704.3	Z95704.3
	CTD-2319I12.2	ZBTB41	ZNF274	C4orf47
	WDR54	KIF11	MON2	MON2
**Heart Atrial**	ENSG00000230850.3	CCDC149	RP11-158H5.7	KLHL18
	CTAGE7P	HCG11	EIF3E	RP11-231P20.2
	C1QC	AC018804.7	SRSF11	RP11-158H5.7
	PTPLAD1	S100A2	ATP6V1H	RP11-296O14.2
	CTD-3224K15.2	TANC2	HOOK3	DNAJC9
**Heart Left Ventricle**	ENSG00000230850.3	AC018804.7	STRAP	LINC00537
	C1QC	CEP135	ANGPTL7	PTPLAD1
	TMEM104	LYSMD2	CTSL3P	ICT1
	CTD-3224K15.2	ZNF192P2	LHX2	UFC1
	RP11-158H5.7	NCF4	DGKD	RP11-423F24.3
**Liver**	C1QC	SETD3	ENSG00000230850.3	ENSG00000230850.3
	CTD-2319I12.2	PTPLAD1	HYAL2	HYAL2
	GMPR2	HIST1H3D	NSUN5	KRBOX1
	RP11-392O18.2	TMEM104	POLR3C	IL6ST
	JAK1	TANC2	BCAS2	RP11-699L21.1
**Lung**	ENSG00000230850.3	RNF115	CYP2A13	CYP2A13
	RP11-142C4.5	CEP135	GS1-531I17.3	RP11-206L10.3
	C1QC	TMEM104	RP1-265C24.8	KIAA0319L
	VWA3B	SHISA5	RP11-112J1.1	RP11-444D3.1
	CTAGE7P	ENSG00000230850.3	ENSG00000237253.1	BMS1P15
**Minor Salivary**	ENSG00000230850.3	KAT2A	CHST4	SCN4A
	CTD-2319I12.2	AC018804.7	RP11-57J16.1	CTD-2287O16.1
	C1QC	PDLIM2	ZNF526	AC011515.2
	KDM8	ADORA2BP	NADK2	CHST4
	GMPR2	AC018865.8	CCDC28B	RGMA
**Muscle Skeletal**	ENSG00000230850.3	ATP2B4	ENSG00000229420.1	TCF24
	CTD-2319I12.2	ANKH	RP11-1018J11.1	ZNF192P2
	C1QC	RNF115	TCF24	BACH1-IT2
	RP11-662J14.1	LINC00537	ZNF192P2	ENSG00000229420.1
	GTF2A1	GALNT12	CCDC149	DGKD
**Nerve Tibial**	ENSG00000230850.3	FAM86A	RP11-363E6.4	NUP210P3
	RPL13AP17	CMTM5	NUP210P3	RP11-363E6.4
	CTD-2291D10.3	MFSD2A	KIF11	PCDHB5
	AC026150.5	ENSG00000230850.3	VWA3B	PDZK1

	CTD-2319I12.2	CTC-297N7.9	RBBP4P4	HPGDS
**Pancreas**	ENSG00000230850.3	ENSG00000230850.3	CEBPD	CEBPD
	CTD-2319I12.2	TMEM163	HMGB3P27	CCDC149
	RP11-662J14.1	CEP135	CCDC149	C4orf48
	GTF2A1	CALM1	C1QC	EOMES
	SUN3	GTF2A1	RBM14	BACH1-IT2
**Pituitary**	ENSG00000230850.3	EIF3E	EBLN1	GTF2A1
	RP11-662J14.1	PTPLAD1	IL13	IL13
	CTD-3224K15.2	CTSV	AC016739.2	AC016739.2
	GMPR2	CCDC149	ST6GALNAC4P1	RP1-144F13.4
	C1QC	MRPS35	CBX3P9	ST6GALNAC4P1
**Skin Not Sun**	ENSG00000230850.3	ENSG00000230850.3	ATF4P4	RP11-7D5.2
	IL17B	DUOXA1	FAM133CP	ATF4P4
	CTD-2319I12.2	LAMP3	RP11-7D5.2	RP5-1097P24.1
	RP11-861A13.4	ADORA2BP	RP5-1097P24.1	GS1-44D20.1
	RNASE3	COG2	HIST1H3J	FAM133CP
**Skin Sun Exposed**	ATF4P4	ENSG00000230850.3	IL17B	IL17B
	RP11-7D5.2	SPATA25	AIRE	ADORA2BP
	FAM133CP	CABLES1	FAM150B	BDH2P1
	RP5-1097P24.1	FAM86A	IL1RAP	FAM150B
	ENSG00000230850.3	C9orf141	RNASE3	AKR1A1
**Small Intestine**	ENSG00000230850.3	PTPLAD1	AKAP8L	HIST1H2BO
	SUN3	TMEM104	RP11-392O18.2	AKAP8L
	CTD-2319I12.2	SPATA25	RP11-223C24.1	LINC00537
	ADORA2BP	ENSG00000230850.3	C17orf77	KLHL18
	CTD-3224K15.2	AC018865.8	CIR1P2	RP6-74O6.6
**Spleen**	ENSG00000230850.3	TMEM104	RP11-142C4.5	RP11-142C4.5
	C1QC	AC018865.8	RP11-712C19.1	FER1L5
	CTAGE7P	LINC00537	AC073410.1	RP11-712C19.1
	GMPR2	CEP135	DEPDC1	NEDD8
	VWA3B	RP11-699L21.1	TPP1	ENSG00000226020.4
**Stomach**	ENSG00000230850.3	RP1-224A6.3	RP11-22L13.1	C19orf80
	VWA3B	KRBOX1	POTEI	HIST1H3D
	CTD-3224K15.2	LINC00537	HIST1H3D	MAMDC4
	CTD-2319I12.2	TMEM104	MAMDC4	IRF8
	C1QC	ATP2B4	PDCD7	PLA2G4A
**Thyroid**	ENSG00000230850.3	RBFOX3	AC026150.5	ZC3H18
	RP11-363E6.4	GPALPP1	ZC3H18	RP11-552D4.1
	RP11-206L10.1	VWA3B	UFM1	ASB7
	RP11-613F22.6	GTF2A1	SP3	ANP32E
	EBLN1	TREML1	ASB7	RP11-973H7.4
**Whole Blood**	ENSG00000230850.3	ENSG00000230850.3	TREML1	GTF2A1
	RP11-142C4.5	CEP135	GTF2A1	AP000322.53
	C1QC	HMGCR	RP11-62H7.3	TREML1
	GMPR2	KRBOX1	RAB1C	RP11-75L1.1

	CTD-3224K15.2	RP11-699L21.1	TREML2	RP11-62H7.3

Table S2. Pathway enrichment for the predicted gene expression using the Reactome database. Only pathways with more than 4 genes and p-value lower than 0.01 are shown

Tissue	Pathway	pval	padj	ES	NES
Colon_Sigmoid	Immune System	3.8E-04	1.2E-02	-5.4E-01	-2.3E+00
Brain_Nucleus	Generic Transcription Pathway	3.0E-03	1.8E-02	7.2E-01	2.1E+00
Brain_Nucleus	RNA Polymerase II Transcription	3.0E-03	1.8E-02	7.2E-01	2.1E+00
Brain_Nucleus	Gene expression (Transcription)	3.0E-03	1.8E-02	7.2E-01	2.1E+00
Colon_Sigmoid	Adaptive Immune System	3.0E-03	3.3E-02	-6.1E-01	-2.1E+00
Colon_Sigmoid	Innate Immune System	2.5E-03	3.3E-02	-6.5E-01	-2.0E+00
Esophagus_Gastroesophageal	Generic Transcription Pathway	4.8E-03	5.3E-02	6.5E-01	2.0E+00
Esophagus_Mucosa	Signaling by Hedgehog	2.1E-03	7.3E-02	8.0E-01	2.1E+00
Esophagus_Mucosa	Hedgehog 'off' state	2.1E-03	7.3E-02	8.0E-01	2.1E+00
Esophagus_Mucosa	Transport of small molecules	3.3E-03	7.7E-02	4.3E-01	2.1E+00
Thyroid	Membrane Trafficking	4.0E-03	8.7E-02	6.1E-01	2.0E+00
Thyroid	Vesicle-mediated transport	4.0E-03	8.7E-02	6.1E-01	2.0E+00
Artery_Coronary	Transcriptional Regulation by TP53	9.5E-03	2.0E-01	6.2E-01	1.9E+00

Table S3. Pathway enrichment for the predicted gene expression using the KEGG database. Only pathways with more than 3 genes and p-value lower than 0.05 are shown

Tissue	Pathway	pval	padj	ES	NES
Esophagus_Mucosa	KEGG GAP JUNCTION	2.0E-03	1.4E-01	8.8E-01	2.1E+00
Esophagus_Mucosa	KEGG DILATED CARDIOMYOPATHY	2.9E-03	1.4E-01	8.4E-01	2.0E+00
Brain_Cerebellar	KEGG HUNTINGTONS DISEASE	2.1E-03	1.4E-01	-8.4E-01	-1.9E+00
Skin_Not_Sun	KEGG FOCAL ADHESION	3.1E-02	2.8E-01	6.4E-01	1.7E+00
Skin_Not_Sun	KEGG NATURAL KILLER CELL MEDIATED CYTOTOXICITY	4.5E-02	2.8E-01	6.7E-01	1.6E+00
Esophagus_Mucosa	KEGG MELANOGENESIS	2.2E-02	3.0E-01	7.2E-01	1.7E+00
Adipose_Subcutaneous	KEGG SYSTEMIC LUPUS ERYTHEMATOSUS	1.6E-02	4.7E-01	-7.6E-01	-1.7E+00
Skin_Sun_Exposed	KEGG ENDOCYTOSIS	2.4E-02	8.7E-01	7.3E-01	1.7E+00

Table S4: Pathway enrichment for the predicted gene expression using the Gene Ontology annotation. Only pathways with more than 4 genes and p-value lower than 0.01 are shown

Tissue	pathway	pval	padj	ES	NES
Adipose_Visceral_Omentum	GO ACTIN BINDING	8.1E-03	9.2E-01	6.7E-01	1.9E+00
Artery_Aorta	GO LEUKOCYTE DIFFERENTIATION	5.9E-03	9.5E-01	7.3E-01	1.9E+00
Artery_Aorta	GO NEUROGENESIS	1.8E-03	9.5E-01	6.1E-01	2.2E+00
Artery_Coronary	GO CHROMOSOME ORGANIZATION	6.0E-03	5.8E-01	5.4E-01	1.9E+00
Artery_Coronary	GO CELLULAR CATABOLIC PROCESS	6.7E-03	5.8E-01	6.2E-01	1.9E+00
Artery_Coronary	GO REGULATION OF HYDROLASE ACTIVITY	4.2E-03	5.8E-01	6.0E-01	2.0E+00
Artery_Coronary	GO NEGATIVE REGULATION OF NITROGEN COMPOUND METABOLIC PROCESS	4.2E-03	5.8E-01	6.0E-01	2.0E+00
Artery_Coronary	GO POSITIVE REGULATION OF HYDROLASE ACTIVITY	1.5E-03	5.8E-01	6.8E-01	2.1E+00

Artery_Coronary	GO PEPTIDYL AMINO ACID MODIFICATION	1.9E-03	5.8E-01	7.3E-01	2.1E+00
Artery_Coronary	GO POSITIVE REGULATION OF MOLECULAR FUNCTION	1.1E-03	5.8E-01	5.5E-01	2.2E+00
Artery_Coronary	GO RESPONSE TO OXYGEN LEVELS	4.5E-03	5.8E-01	7.4E-01	2.0E+00
Artery_Coronary	GO REGULATION OF CELLULAR RESPONSE TO STRESS	3.0E-03	5.8E-01	6.9E-01	2.0E+00
Artery_Coronary	GO CELLULAR RESPONSE TO DNA DAMAGE STIMULUS	2.5E-03	5.8E-01	6.2E-01	2.0E+00
Artery_Coronary	GO CELLULAR MACROMOLECULAR COMPLEX ASSEMBLY	4.0E-03	5.8E-01	6.1E-01	2.0E+00
Artery_Tibial	GO INTRACELLULAR PROTEIN TRANSPORT	9.6E-03	7.7E-01	5.5E-01	1.9E+00
Artery_Tibial	GO CYTOSKELETAL PROTEIN BINDING	4.8E-03	7.7E-01	7.4E-01	2.0E+00
Brain_Anterior_cingulate	GO PROTEIN OLIGOMERIZATION	6.5E-03	4.9E-01	6.7E-01	1.9E+00
Brain_Anterior_cingulate	GO PROTEIN COMPLEX BIOGENESIS	7.6E-04	4.9E-01	6.9E-01	2.4E+00
Brain_Anterior_cingulate	GO POSITIVE REGULATION OF IMMUNE SYSTEM PROCESS	1.7E-03	4.9E-01	7.9E-01	2.1E+00
Brain_Anterior_cingulate	GO MACROMOLECULAR COMPLEX ASSEMBLY	3.6E-03	4.9E-01	5.8E-01	2.1E+00
Brain_Anterior_cingulate	GO REGULATION OF IMMUNE SYSTEM PROCESS	1.5E-03	4.9E-01	7.4E-01	2.2E+00
Brain_Anterior_cingulate	GO PROTEIN COMPLEX SUBUNIT ORGANIZATION	2.7E-04	4.6E-01	6.9E-01	2.7E+00
Brain_Anterior_cingulate	GO INTRINSIC COMPONENT OF PLASMA MEMBRANE	3.0E-03	4.9E-01	5.5E-01	2.2E+00
Brain_Anterior_cingulate	GO ENZYME BINDING	5.8E-03	4.9E-01	5.9E-01	2.0E+00
Brain_Cerebellar	GO CELL PART MORPHOGENESIS	3.6E-03	5.5E-01	7.1E-01	2.0E+00
Brain_Cerebellar	GO CELLULAR RESPONSE TO OXYGEN CONTAINING COMPOUND	2.1E-03	5.5E-01	5.4E-01	2.1E+00
Brain_Cerebellar	GO NUCLEOBASE CONTAINING SMALL MOLECULE METABOLIC PROCESS	5.3E-03	5.5E-01	-7.2E-01	-1.9E+00
Brain_Cerebellar	GO RESPONSE TO ENDOGENOUS STIMULUS	9.1E-03	5.5E-01	3.7E-01	1.9E+00
Brain_Cerebellar	GO RESPONSE TO HORMONE	3.7E-03	5.5E-01	5.5E-01	2.0E+00
Brain_Cerebellar	GO RESPONSE TO OXYGEN CONTAINING COMPOUND	6.5E-03	5.5E-01	3.8E-01	1.9E+00
Brain_Cerebellar	GO GLYCOSYL COMPOUND METABOLIC PROCESS	5.3E-03	5.5E-01	-7.2E-01	-1.9E+00

Brain_Cerebellar	GO NUCLEOSIDE MONOPHOSPHATE METABOLIC PROCESS	5.3E-03	5.5E-01	-7.2E-01	-1.9E+00
Brain_Cerebellar	GO PURINE CONTAINING COMPOUND METABOLIC PROCESS	5.3E-03	5.5E-01	-7.2E-01	-1.9E+00
Brain_Putamen	GO PROTEIN DIMERIZATION ACTIVITY	7.4E-03	8.6E-01	6.3E-01	2.0E+00
Brain_Substantia_nigra	GO ESTABLISHMENT OF LOCALIZATION IN CELL	5.3E-03	8.9E-01	7.0E-01	2.0E+00
Brain_Substantia_nigra	GO ENDOPLASMIC RETICULUM PART	6.1E-03	8.9E-01	7.5E-01	2.0E+00
Cells_Transformed	GO PROTEIN DIMERIZATION ACTIVITY	6.6E-03	7.8E-01	5.3E-01	2.0E+00
Colon_Sigmoid	GO POSITIVE REGULATION OF HOMEOSTATIC PROCESS	8.2E-03	8.0E-01	-7.2E-01	-1.8E+00
Colon_Sigmoid	GO NEGATIVE REGULATION OF MOLECULAR FUNCTION	6.6E-03	8.0E-01	-6.2E-01	-1.9E+00
Colon_Sigmoid	GO NEGATIVE REGULATION OF CATALYTIC ACTIVITY	4.0E-03	8.0E-01	-7.1E-01	-2.0E+00
Colon_Transverse	GO CELL PART MORPHOGENESIS	5.8E-03	8.9E-01	6.1E-01	2.0E+00
Colon_Transverse	GO SINGLE ORGANISM BIOSYNTHETIC PROCESS	2.4E-03	8.9E-01	5.1E-01	2.1E+00
Colon_Transverse	GO MITOCHONDRION ORGANIZATION	4.7E-03	8.9E-01	6.5E-01	2.0E+00
Colon_Transverse	GO NEGATIVE REGULATION OF MULTICELLULAR ORGANISMAL PROCESS	6.1E-03	8.9E-01	-6.1E-01	-1.9E+00
Colon_Transverse	GO NEGATIVE REGULATION OF CELL DIFFERENTIATION	5.0E-03	8.9E-01	-7.5E-01	-1.9E+00
Colon_Transverse	GO TRANSFERASE COMPLEX	7.3E-03	8.9E-01	5.0E-01	2.0E+00
Esophagus_Gastroesophageal	GO NEGATIVE REGULATION OF GENE EXPRESSION	6.9E-04	3.8E-01	8.0E-01	2.3E+00
Esophagus_Gastroesophageal	GO CELL DEATH	4.8E-04	3.8E-01	6.7E-01	2.4E+00
Esophagus_Gastroesophageal	GO NEGATIVE REGULATION OF NITROGEN COMPOUND METABOLIC PROCESS	2.3E-04	3.8E-01	8.0E-01	2.4E+00
Esophagus_Gastroesophageal	GO NEGATIVE REGULATION OF TRANSCRIPTION FROM RNA POLYMERASE II PROMOTER	1.3E-03	5.6E-01	7.9E-01	2.1E+00
Esophagus_Mucosa	GO ION TRANSPORT	6.2E-03	4.0E-01	4.1E-01	2.0E+00
Esophagus_Mucosa	GO INTRACELLULAR PROTEIN TRANSPORT	2.0E-03	4.0E-01	6.1E-01	2.0E+00
Esophagus_Mucosa	GO RESPONSE TO PEPTIDE	1.7E-03	4.0E-01	5.5E-01	2.2E+00
Esophagus_Mucosa	GO ESTABLISHMENT OF PROTEIN LOCALIZATION	5.3E-03	4.0E-01	4.0E-01	2.0E+00
Esophagus_Mucosa	GO CYTOKINE MEDIATED SIGNALING PATHWAY	1.1E-03	4.0E-01	7.0E-01	2.2E+00

Esophagus_Mucosa	GO ESTABLISHMENT OF LOCALIZATION IN CELL	1.0E-03	4.0E-01	4.0E-01	2.4E+00
Esophagus_Mucosa	GO REGULATION OF METAL ION TRANSPORT	8.8E-03	4.0E-01	5.8E-01	1.9E+00
Esophagus_Mucosa	GO G PROTEIN COUPLED RECEPTOR SIGNALING PATHWAY	9.8E-03	4.0E-01	4.5E-01	1.9E+00
Esophagus_Mucosa	GO PROTEIN PHOSPHORYLATION	7.8E-03	4.0E-01	5.0E-01	1.9E+00
Esophagus_Mucosa	GO CELLULAR RESPONSE TO NITROGEN COMPOUND	8.7E-03	4.0E-01	4.8E-01	1.9E+00
Esophagus_Mucosa	GO PROTEASOMAL PROTEIN CATABOLIC PROCESS	2.6E-03	4.0E-01	7.8E-01	2.1E+00
Esophagus_Mucosa	GO CELLULAR RESPONSE TO PEPTIDE	9.4E-03	4.0E-01	5.4E-01	1.9E+00
Esophagus_Mucosa	GO PHOSPHORYLATION	7.8E-03	4.0E-01	5.0E-01	1.9E+00
Esophagus_Mucosa	GO AXON	6.4E-03	4.0E-01	5.6E-01	2.0E+00
Esophagus_Mucosa	GO ENDOPLASMIC RETICULUM PART	3.3E-03	4.0E-01	4.4E-01	2.2E+00
Esophagus_Mucosa	GO NUCLEAR OUTER MEMBRANE ENDOPLASMIC RETICULUM MEMBRANE NETWORK	4.1E-03	4.0E-01	4.3E-01	2.1E+00
Esophagus_Mucosa	GO T TUBULE	6.9E-03	4.0E-01	7.2E-01	1.9E+00
Esophagus_Mucosa	GO SARCOLEMMA	6.0E-03	4.0E-01	6.2E-01	1.9E+00
Esophagus_Mucosa	GO TRANSPORT VESICLE	3.2E-03	4.0E-01	5.6E-01	2.1E+00
Esophagus_Mucosa	GO CELL LEADING EDGE	9.8E-03	4.0E-01	5.1E-01	1.9E+00
Esophagus_Muscularis	GO HYDROLASE ACTIVITY ACTING ON ESTER BONDS	5.9E-03	6.0E-01	6.8E-01	2.0E+00
Heart_Atrial	GO RESPONSE TO INORGANIC SUBSTANCE	3.8E-03	2.8E-01	7.5E-01	2.0E+00
Heart_Atrial	GO VESICLE MEMBRANE	3.8E-03	2.8E-01	5.3E-01	2.1E+00
Heart_Left_Ventricle	GO PROTEIN HOMODIMERIZATION ACTIVITY	8.8E-03	9.7E-01	6.3E-01	1.9E+00
Liver	GO POSITIVE REGULATION OF CELL COMMUNICATION	8.0E-03	9.9E-01	4.9E-01	2.0E+00
Liver	GO CYTOSKELETAL PROTEIN BINDING	4.5E-03	9.9E-01	6.5E-01	2.0E+00
Liver	GO MOLECULAR FUNCTION REGULATOR	1.0E-02	9.9E-01	6.5E-01	1.9E+00
Lung	GO G PROTEIN COUPLED RECEPTOR SIGNALING PATHWAY	3.3E-03	9.1E-01	5.6E-01	2.0E+00
Lung	GO MITOCHONDRION ORGANIZATION	7.1E-03	9.1E-01	6.3E-01	1.9E+00
Lung	GO REGULATION OF CELL DEVELOPMENT	9.5E-03	9.1E-01	6.5E-01	1.9E+00

Lung	GO NEGATIVE REGULATION OF CELLULAR COMPONENT ORGANIZATION	1.9E-03	9.1E-01	5.5E-01	2.1E+00
Lung	GO ADENYL NUCLEOTIDE BINDING	4.9E-03	9.1E-01	4.4E-01	2.0E+00
Minor_Salivary	GO PHOSPHORYLATION	8.3E-03	9.1E-01	5.8E-01	1.9E+00
Minor_Salivary	GO RIBONUCLEOTIDE BINDING	1.8E-03	9.1E-01	6.4E-01	2.2E+00
Minor_Salivary	GO ADENYL NUCLEOTIDE BINDING	2.0E-03	9.1E-01	6.3E-01	2.1E+00
Muscle_Skeletal	GO CELLULAR CATABOLIC PROCESS	8.4E-03	9.3E-01	3.6E-01	1.9E+00
Muscle_Skeletal	GO POSITIVE REGULATION OF CELLULAR PROTEIN LOCALIZATION	7.4E-03	9.3E-01	-7.5E-01	-1.8E+00
Muscle_Skeletal	GO CELLULAR PROTEIN COMPLEX ASSEMBLY	4.7E-03	9.3E-01	7.4E-01	2.0E+00
Muscle_Skeletal	GO CELLULAR LIPID METABOLIC PROCESS	1.7E-03	8.2E-01	5.3E-01	2.2E+00
Muscle_Skeletal	GO NEGATIVE REGULATION OF HYDROLASE ACTIVITY	9.4E-03	9.3E-01	7.0E-01	1.9E+00
Muscle_Skeletal	GO CELLULAR RESPONSE TO STRESS	4.4E-04	8.2E-01	4.7E-01	2.6E+00
Muscle_Skeletal	GO PROTEOLYSIS	3.8E-03	9.3E-01	4.4E-01	2.1E+00
Nerve_Tibial	GO TISSUE MORPHOGENESIS	8.7E-03	9.7E-01	6.2E-01	1.9E+00
Nerve_Tibial	GO CELL PROLIFERATION	4.8E-03	9.7E-01	5.5E-01	2.0E+00
Nerve_Tibial	GO REGULATION OF CELL DIFFERENTIATION	4.2E-03	9.7E-01	4.2E-01	2.1E+00
Nerve_Tibial	GO TISSUE DEVELOPMENT	2.2E-03	9.7E-01	4.8E-01	2.2E+00
Pancreas	GO POSITIVE REGULATION OF PROTEIN MODIFICATION PROCESS	3.4E-03	6.1E-01	5.3E-01	2.0E+00
Pancreas	GO MITOCHONDRIAL ENVELOPE	7.5E-03	6.1E-01	7.1E-01	1.9E+00
Pituitary	GO CELL DIVISION	2.6E-03	7.0E-01	7.7E-01	2.0E+00
Pituitary	GO MITOCHONDRION	5.2E-03	7.0E-01	5.6E-01	2.0E+00
Pituitary	GO CYTOSKELETAL PROTEIN BINDING	3.5E-03	7.0E-01	6.0E-01	2.1E+00
Skin_Sun_Exposed	GO REGULATION OF HYDROLASE ACTIVITY	5.4E-03	9.8E-01	3.8E-01	2.0E+00
Skin_Sun_Exposed	GO REGULATION OF CELL DIFFERENTIATION	1.8E-03	9.8E-01	4.3E-01	2.1E+00
Skin_Sun_Exposed	GO REGULATION OF CYTOSKELETON ORGANIZATION	8.9E-03	9.8E-01	6.4E-01	2.0E+00
Skin_Sun_Exposed	GO CELLULAR COMPONENT MORPHOGENESIS	4.2E-03	9.8E-01	4.7E-01	2.0E+00
Skin_Sun_Exposed	GO CELL PROJECTION ORGANIZATION	4.2E-03	9.8E-01	4.7E-01	2.0E+00

Skin_Sun_Exposed	GO POSITIVE REGULATION OF CELL DIFFERENTIATION	3.0E-03	9.8E-01	5.0E-01	2.1E+00
Skin_Sun_Exposed	GO REPRODUCTION	4.9E-03	9.8E-01	4.6E-01	2.0E+00
Skin_Sun_Exposed	GO CELL DEVELOPMENT	4.7E-04	9.8E-01	5.0E-01	2.5E+00
Skin_Sun_Exposed	GO CELL CORTEX	8.5E-03	9.8E-01	6.8E-01	1.9E+00
Skin_Sun_Exposed	GO CELL PROJECTION PART	5.3E-03	9.8E-01	4.4E-01	2.0E+00
Spleen	GO REGULATION OF CELL DEVELOPMENT	1.3E-03	6.6E-01	5.4E-01	2.1E+00
Spleen	GO CYTOPLASMIC VESICLE PART	8.8E-03	6.6E-01	6.0E-01	1.9E+00
Stomach	GO NEUROGENESIS	4.0E-03	9.5E-01	5.4E-01	2.1E+00
Stomach	GO RECEPTOR MEDIATED ENDOCYTOSIS	1.6E-03	7.6E-01	7.9E-01	2.1E+00
Stomach	GO CELL DEVELOPMENT	5.2E-04	6.4E-01	6.8E-01	2.5E+00
Thyroid	GO REGULATION OF HYDROLASE ACTIVITY	4.4E-03	6.9E-01	4.5E-01	2.1E+00
Thyroid	GO REGULATION OF CELLULAR COMPONENT BIOGENESIS	1.6E-03	6.9E-01	6.4E-01	2.1E+00
Thyroid	GO POSITIVE REGULATION OF HYDROLASE ACTIVITY	6.2E-03	6.9E-01	4.7E-01	1.9E+00
Thyroid	GO POSITIVE REGULATION OF CATALYTIC ACTIVITY	3.8E-03	6.9E-01	4.7E-01	2.1E+00
Thyroid	GO REGULATION OF CELL PROJECTION ORGANIZATION	6.1E-04	5.3E-01	7.3E-01	2.2E+00
Thyroid	GO POSITIVE REGULATION OF MOLECULAR FUNCTION	2.3E-03	6.9E-01	4.6E-01	2.2E+00
Thyroid	GO REGULATION OF NEURON DIFFERENTIATION	6.4E-03	6.9E-01	5.9E-01	1.9E+00
Thyroid	GO BLOOD VESSEL MORPHOGENESIS	2.0E-03	6.9E-01	7.1E-01	2.0E+00
Thyroid	GO VASCULATURE DEVELOPMENT	6.1E-04	5.3E-01	7.3E-01	2.3E+00
Thyroid	GO REGULATION OF SYSTEM PROCESS	9.4E-03	6.9E-01	6.0E-01	1.9E+00
Thyroid	GO REGULATION OF MUSCLE SYSTEM PROCESS	4.7E-03	6.9E-01	7.5E-01	2.0E+00
Thyroid	GO ANGIOGENESIS	9.1E-03	6.9E-01	7.0E-01	1.8E+00
Thyroid	GO CIRCULATORY SYSTEM DEVELOPMENT	3.4E-04	5.3E-01	7.1E-01	2.4E+00
Whole_Blood	GO REGULATION OF CELLULAR COMPONENT SIZE	1.3E-03	9.7E-01	6.7E-01	2.2E+00
Whole_Blood	GO REGULATION OF HORMONE LEVELS	6.0E-03	9.7E-01	6.3E-01	1.9E+00
Whole_Blood	GO REGULATION OF ANATOMICAL STRUCTURE SIZE	6.2E-03	9.7E-01	5.5E-01	2.0E+00
Whole_Blood	GO CHROMATIN MODIFICATION	6.0E-03	9.7E-01	6.3E-01	1.9E+00

Whole_Blood	GO ORGANIZATION	CHROMATIN	6.0E-03	9.7E-01	6.3E-01	1.9E+00

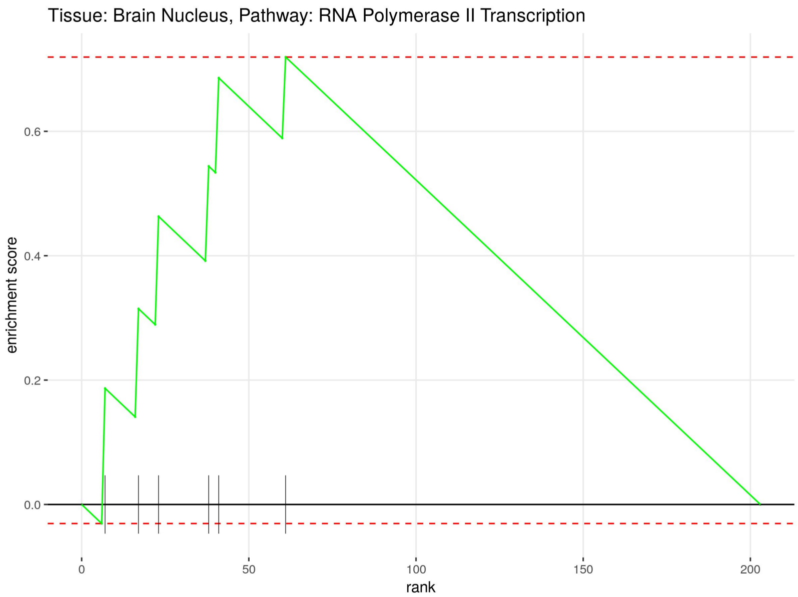

Figure S1: Positive enrichment plot for the “RNA Polymerase II Transcription” pathway in the Brain Nucleus tissue.

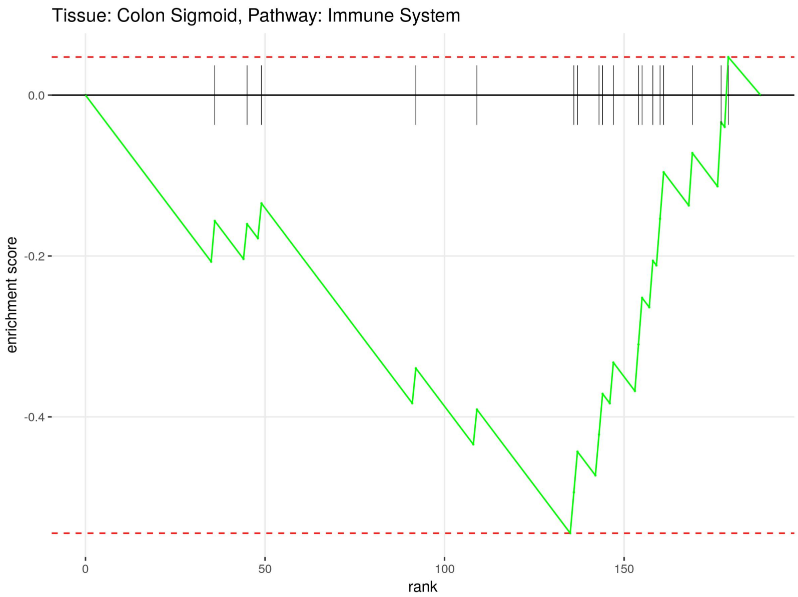

Figure S2: Negative enrichment plot for the “Immune System” pathway in the Colon Sigmoid tissue.

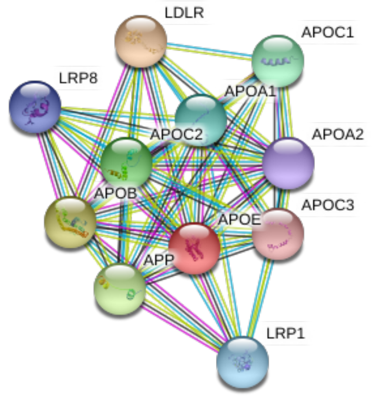

Figure S3: Representative interactions involving *APOE* gene. Lines represent predicted functional edges of interaction between nodes, and they are represented with eight different colors according to the type of evidence and the predictive method used (neighborhood, gene fusion, co-occurrence, co-expression, experiments, databases, and text-mining). For details refer to http://string-db.org.

2 SUPERVISED MODELS EVALUATION

2.1 ADNI1-GWAS cross-tissue models training scores

Tissue	ADNI1-GWAS 10CV Mean Scores				
	AUC	Accuracy	Precision	Recall	F1
Adipose Subcutaneus	0.95	95.50	0.96	0.99	0.97
Adipose Visceral Omentum	0.84	82.83	0.91	0.84	0.83
Adrenal Gland	0.87	87.00	0.93	0.87	0.88
Artery Aorta	0.92	93.00	0.93	0.99	0.95
Artery Coronary	0.94	95.00	0.95	0.98	0.96
Artery Tibial	0.94	94.67	0.96	0.97	0.96
Brain Amygdala	0.95	95.33	0.95	0.99	0.97
Brain Anterior cingulate cortex BA24	0.94	95.00	0.95	0.98	0.96
Brain Caudate basal ganglia	0.92	93.17	0.93	0.97	0.95
Brain Cerebellar Hemisphere	0.88	89.67	0.89	0.97	0.92
Brain Cerebellum	0.87	85.83	0.93	0.88	0.86
Brain Cortex	0.92	93.33	0.93	0.98	0.95
Brain Frontal Cortex BA9	0.95	96.00	0.96	0.98	0.97
Brain Hippocampus	0.94	95.17	0.95	0.98	0.96
Brain Hypothalamus	0.93	94.00	0.94	0.98	0.96
Brain Nucleus accumbens basal ganglia	0.91	91.67	0.94	0.94	0.93
Brain Putamen basal ganglia	0.94	94.33	0.95	0.97	0.96
Brain Spinal cord cervical c-1	0.92	93.00	0.93	0.97	0.95
Brain Substantia nigra	0.95	95.50	0.95	0.98	0.97
Cells EBV-transformed lymphocytes	0.94	95.33	0.96	0.99	0.97
Cells Transformed fibroblasts	0.93	94.33	0.94	0.98	0.96
Colon Sigmoid	0.94	94.33	0.94	0.97	0.96
Colon Transverse	0.95	95.67	0.95	0.99	0.97
Esophagus Gastroesophageal Junction	0.93	93.00	0.95	0.95	0.95
Esophagus Mucosa	0.91	92.67	0.93	0.98	0.95
Esophagus Muscularis	0.94	94.50	0.96	0.97	0.96
Heart Atrial Appendage	0.90	90.33	0.95	0.92	0.91
Heart Left Ventricle	0.93	93.33	0.95	0.94	0.94
Liver	094	94.83	0.94	0.99	0.96
Lung	0.95	93.50	0.92	0.99	0.95
Minor Salivary Gland	0.95	95.83	0.96	0.98	0.97
Muscle Skeletal	0.90	90.67	0.96	0.91	0.92
Nerve Tibial	0.87	88.00	0.95	0.87	0.88
Pancreas	0.89	88.00	0.94	0.89	0.89
Pituitary	0.95	95.67	0.96	0.99	0.97
Skin Not Sun Exposed Suprapubic	0.94	94.67	0.95	0.97	0.96
Skin Sun Exposed Lower leg	0.94	95.50	0.96	0.99	0.97
Small Intestine Terminal Ileum	0.94	95.17	0.95	0.98	0.96
Spleen	0.93	94.33	0.94	0.98	0.96
Stomach	0.95	95.50	0.95	0.98	0.97
Thyroid	0.93	93.33	0.96	0.95	0.94
Whole Blood	0.85	85.17	0.92	0.89	0.87

Tissue	ADNI1-GWAS - UnList 10CV Mean Scores				
	AUC	Accuracy	Precision	Recall	F1
Adipose Subcutaneus	0.93	93.00	0.93	0.95	0.94
Adipose Visceral Omentum	0.94	94.67	0.95	0.96	0.96
Adrenal Gland	0.94	94.17	0.95	0.95	0.95
Artery Aorta	0.95	95.17	0.95	0.97	0.96
Artery Coronary	0.94	93.83	0.95	0.93	0.94
Artery Tibial	0.95	95.67	0.96	0.98	0.97
Brain Amygdala	0.92	92.50	0.95	0.92	0.93
Brain Anterior cingulate cortex BA24	0.92	92.17	0.95	0.93	0.94
Brain Caudate basal ganglia	0.94	94.17	0.94	0.97	0.95
Brain Cerebellar Hemisphere	0.94	94.17	0.95	0.95	0.95
Brain Cerebellum	0.95	9533	0.96	0.97	0.96
Brain Cortex	0.92	92.00	0.94	0.92	0.93
Brain Frontal Cortex BA9	0.92	92.67	0.93	0.94	0.93
Brain Hippocampus	0.95	95.00	0.96	0.96	0.96
Brain Hypothalamus	0.94	95.17	0.95	0.98	0.96
Brain Nucleus accumbens basal ganglia	0.93	92.50	0.94	0.91	0.93
Brain Putamen basal ganglia	0.94	95.00	0.96	0.96	0.96
Brain Spinal cord cervical c-1	0.93	93.83	0.94	0.97	0.95
Brain Substantia nigra	0.93	93.00	0.94	0.94	0.94
Cells EBV-transformed lymphocytes	0.93	93.17	0.96	0.93	0.94
Cells Transformed fibroblasts	0.95	95.50	0.95	0.99	0.97
Colon Sigmoid	0.94	94.50	0.95	0.95	0.95
Colon Transverse	0.94	94.17	0.96	0.93	0.94
Esophagus Gastroesophageal Junction	0.94	94.83	0.96	0.96	0.96
Esophagus Mucosa	0.95	95.33	0.95	0.98	0.96
Esophagus Muscularis	0.94	93.67	0.95	0.93	0.94
Heart Atrial Appendage	0.96	96.33	0.97	0.98	0.97
Heart Left Ventricle	0.93	93.67	0.94	0.96	0.95
Liver	0.91	91.83	0.93	0.93	0.93
Lung	0.92	92.00	0.93	0.93	0.93
Minor Salivary Gland	0.95	95.50	0.97	0.95	0.96
Muscle Skeletal	0.95	95.50	0.96	0.97	0.96
Nerve Tibial	0.95	95.17	0.96	0.96	0.96
Pancreas	0.94	94.67	0.95	0.96	0.96
Pituitary	0.94	94.50	0.95	0.96	0.96
Skin Not Sun Exposed Suprapubic	0.93	93.50	0.94	0.95	0.94
Skin Sun Exposed Lower leg	0.94	94.83	0.95	0.97	0.96
Small Intestine Terminal Ileum	0.95	95.17	0.96	0.97	0.94
Spleen	0.95	95.00	0.96	0.95	0.96
Stomach	0.94	94.67	0.95	0.97	0.96
Thyroid	0.93	93.67	0.94	0.95	0.95
Whole Blood	0.93	93.33	0.96	0.93	0.94

Tissue	ADNI1-GWAS - PredixList 10CV Mean Scores				
	AUC	Accuracy	Precision	Recall	F1
Adipose Subcutaneus	0.95	95.83	0.96	0.98	0.97
Adipose Visceral Omentum	0.91	92.17	0.91	0.98	0.94
Adrenal Gland	0.95	95.50	0.96	0.98	0.97
Artery Aorta	0.95	95.67	0.96	0.98	0.97
Artery Coronary	0.90	91.00	0.91	0.96	0.93
Artery Tibial	0.94	94.17	0.94	0.97	0.95
Brain Amygdala	0.95	95.00	0.95	0.96	0.96
Brain Anterior cingulate cortex BA24	0.93	93.83	0.95	0.95	0.95
Brain Caudate basal ganglia	0.94	95.00	0.95	0.98	0.96
Brain Cerebellar Hemisphere	0.95	95.50	0.96	0.97	0.96
Brain Cerebellum	0.94	94.17	0.95	0.95	0.95
Brain Cortex	0.95	95.17	0.96	0.97	0.96
Brain Frontal Cortex BA9	0.95	95.83	0.96	0.97	0.97
Brain Hippocampus	0.95	95.33	0.96	0.96	0.96
Brain Hypothalamus	0.95	95.33	0.96	0.96	0.96
Brain Nucleus accumbens basal ganglia	0.94	94.00	0.95	0.94	0.95
Brain Putamen basal ganglia	0.96	95.83	0.96	0.96	0.96
Brain Spinal cord cervical c-1	0.94	95.00	0.95	0.97	0.96
Brain Substantia nigra	0.96	96.17	0.96	0.97	0.97
Cells EBV-transformed lymphocytes	0.94	94.17	0.95	0.95	0.95
Cells Transformed fibroblasts	0.95	95.50	0.96	0.97	0.97
Colon Sigmoid	0.95	95.50	0.95	0.98	0.97
Colon Transverse	0.95	95.67	0.96	0.97	0.96
Esophagus Gastroesophageal Junction	0.94	94.67	0.95	0.97	0.95
Esophagus Mucosa	0.95	95.67	0.96	0.99	0.97
Esophagus Muscularis	0.96	95.50	0.96	0.98	0.97
Heart Atrial Appendage	0.94	94.33	0.94	0.98	0.96
Heart Left Ventricle	0.96	96.00	0.96	0.97	0.97
Liver	0.94	95.00	0.96	0.97	0.96
Lung	0.94	94.33	0.94	0.97	0.96
Minor Salivary Gland	0.94	93.83	0.94	0.95	0.94
Muscle Skeletal	0.94	94.83	0.94	0.98	0.96
Nerve Tibial	0.93	94.17	0.94	0.98	0.96
Pancreas	0.89	90.83	0.91	0.97	0.93
Pituitary	0.95	95.50	0.95	0.98	0.97
Skin Not Sun Exposed Suprapubic	0.95	95.33	0.96	0.98	0.97
Skin Sun Exposed Lower leg	0.94	94.83	0.95	0.96	0.96
Small Intestine Terminal Ileum	0.95	95.17	0.95	0.97	0.96
Spleen	0.95	95.17	0.95	0.97	0.96
Stomach	0.95	95.17	0.95	0.97	0.96
Thyroid	0.88	89.83	0.90	0.97	0.93
Whole Blood	0.95	95.33	0.95	0.98	0.97

2.2 Cognitive Decline cross-tissue models training scores

Tissue	Cognitive Decline 10CV Mean Scores				
	AUC	Accuracy	Precision	Recall	F1
Adipose Subcutaneus	0.95	94.75	0.94	0.96	0.95
Adipose Visceral Omentum	0.95	95.00	0.94	0.92	0.93
Adrenal Gland	0.95	95.00	0.94	0.96	0.95
Artery Aorta	0.95	95.75	0.97	0.93	0.94
Artery Coronary	0.95	95.00	0.95	0.95	0.95
Artery Tibial	0.95	94.75	0.94	0.94	0.94
Brain Amygdala	0.94	93.75	0.93	0.95	0.94
Brain Anterior cingulate cortex BA24	0.96	96.00	0.96	0.93	0.94
Brain Caudate basal ganglia	0.95	95.50	0.95	0.96	0.95
Brain Cerebellar Hemisphere	0.96	96.25	0.96	0.94	0.95
Brain Cerebellum	0.95	95.50	0.95	0.93	0.94
Brain Cortex	0.95	95.50	0.95	0.94	0.94
Brain Frontal Cortex BA9	0.96	96.00	0.95	0.95	0.95
Brain Hippocampus	0.95	95.25	0.95	0.95	0.95
Brain Hypothalamus	0.95	94.75	0.94	0.95	0.95
Brain Nucleus accumbens basal ganglia	0.95	95.25	0.95	0.95	0.95
Brain Putamen basal ganglia	0.93	93.50	0.95	0.88	0.91
Brain Spinal cord cervical c-1	0.94	94.00	0.94	0.97	0.95
Brain Substantia nigra	0.95	95.75	0.95	0.94	0.94
Cells EBV-transformed lymphocytes	0.94	94.50	0.94	0.92	0.93
Cells Transformed fibroblasts	0.95	95.00	0.95	0.96	0.95
Colon Sigmoid	0.94	94.25	0.94	0.93	0.94
Colon Transverse	0.95	95.25	0.95	0.94	0.94
Esophagus Gastroesophageal Junction	0.96	96.00	0.95	0.95	0.95
Esophagus Mucosa	0.95	94.75	0.94	0.93	0.94
Esophagus Muscularis	0.95	95.00	0.94	0.93	0.93
Heart Atrial Appendage	0.96	95.75	0.95	0.95	0.95
Heart Left Ventricle	0.94	94.50	0.94	0.93	0.94
Liver	0.95	95.25	0.95	0.94	0.94
Lung	0.95	95.25	0.95	0.94	0.94
Minor Salivary Gland	0.94	94.50	0.94	0.92	0.93
Muscle Skeletal	0.91	90.50	0.94	0.87	0.88
Nerve Tibial	0.96	95.75	0.95	0.94	0.95
Pancreas	0.95	95.00	0.94	0.94	0.94
Pituitary	0.96	96.25	0.96	0.95	0.96
Skin Not Sun Exposed Suprapubic	0.95	95.00	0.94	0.95	0.94
Skin Sun Exposed Lower leg	0.95	95.75	0.95	0.94	0.94
Small Intestine Terminal Ileum	0.95	95.25	0.94	0.92	0.93
Spleen	0.95	95.75	0.95	0.94	0.94
Stomach	0.95	95.50	0.95	0.94	0.94
Thyroid	0.95	95.75	0.96	0.92	0.94
Whole Blood	0.94	93.50	0.94	0.95	0.94

Tissue	Cognitive Decline - UnList 10CV Mean Scores				
	AUC	Accuracy	Precision	Recall	F1
Adipose Subcutaneus	0.93	93.25	0.94	0.93	0.93
Adipose Visceral Omentum	0.95	94.75	0.94	0.96	0.95
Adrenal Gland	0.94	94.50	0.94	0.92	0.93
Artery Aorta	0.95	95.00	0.94	0.95	0.95
Artery Coronary	0.95	95.00	0.96	0.94	0.94
Artery Tibial	0.95	95.00	0.94	0.94	0.94
Brain Amygdala	0.93	93.00	0.92	0.95	0.93
Brain Anterior cingulate cortex BA24	0.94	93.50	0.94	0.93	0.94
Brain Caudate basal ganglia	0.95	94.75	0.96	0.93	0.94
Brain Cerebellar Hemisphere	0.95	95.25	0.96	0.92	0.93
Brain Cerebellum	0.92	92.25	0.94	0.92	0.92
Brain Cortex	0.94	94.00	0.93	0.98	0.95
Brain Frontal Cortex BA9	0.95	94.75	0.94	0.95	0.94
Brain Hippocampus	0.94	94.25	0.94	0.93	0.94
Brain Hypothalamus	0.95	95.50	0.95	0.94	0.94
Brain Nucleus accumbens basal ganglia	0.95	95.25	0.95	0.94	0.95
Brain Putamen basal ganglia	0.93	92.75	0.96	0.87	0.90
Brain Spinal cord cervical c-1	0.93	93.75	0.93	0.93	0.93
Brain Substantia nigra	0.94	94.00	0.94	0.96	0.95
Cells EBV-transformed lymphocytes	0.94	94.25	0.93	0.94	0.94
Cells Transformed fibroblasts	0.95	94.75	0.94	0.94	0.94
Colon Sigmoid	0.95	94.75	0.95	0.94	0.95
Colon Transverse	0.95	95.25	0.95	0.95	0.95
Esophagus Gastroesophageal Junction	0.96	96.00	0.96	0.96	0.96
Esophagus Mucosa	0.93	93.50	0.92	0.92	0.92
Esophagus Muscularis	0.95	95.50	0.95	0.96	0.96
Heart Atrial Appendage	0.94	94.75	0.95	0.91	0.93
Heart Left Ventricle	0.92	92.75	0.95	0.88	0.91
Liver	0.94	94.50	0.94	0.94	0.94
Lung	0.96	96.25	0.96	0.95	0.95
Minor Salivary Gland	0.93	93.25	0.91	0.94	0.92
Muscle Skeletal	0.93	93.50	0.93	0.94	0.93
Nerve Tibial	0.95	95.25	0.95	0.95	0.95
Pancreas	0.96	95.75	0.95	0.95	0.95
Pituitary	0.93	93.50	0.93	0.93	0.93
Skin Not Sun Exposed Suprapubic	0.94	94.25	0.94	0.93	0.93
Skin Sun Exposed Lower leg	0.95	95.00	0.94	0.94	0.94
Small Intestine Terminal Ileum	0.95	94.50	0.93	0.96	0.95
Spleen	0.95	95.50	0.94	0.97	0.95
Stomach	0.94	94.50	0.94	0.95	0.94
Thyroid	0.94	94.25	0.93	0.93	0.93
Whole Blood	0.94	93.75	0.93	0.96	0.94

Tissue	Cognitive Decline - PredixList 10CV Mean Scores				
	AUC	Accuracy	Precision	Recall	F1
Adipose Subcutaneus	0.93	93.50	0.94	0.91	0.93
Adipose Visceral Omentum	0.95	95.00	0.94	0.94	0.94
Adrenal Gland	0.95	95.25	0.95	0.94	0.94
Artery Aorta	0.95	95.00	0.94	0.94	0.94
Artery Coronary	0.94	94.00	0.94	0.95	0.94
Artery Tibial	0.94	94.50	0.94	0.94	0.94
Brain Amygdala	0.95	94.75	0.94	0.93	0.94
Brain Anterior cingulate cortex BA24	0.95	95.00	0.94	0.96	0.95
Brain Caudate basal ganglia	0.95	95.25	0.95	0.95	0.95
Brain Cerebellar Hemisphere	0.95	95.50	0.95	0.95	0.95
Brain Cerebellum	0.95	95.25	0.95	0.95	0.95
Brain Cortex	0.95	95.00	0.94	0.93	0.94
Brain Frontal Cortex BA9	0.94	94.50	0.94	0.93	0.94
Brain Hippocampus	0.94	94.50	0.94	0.93	0.93
Brain Hypothalamus	0.94	94.25	0.94	0.94	0.94
Brain Nucleus accumbens basal ganglia	0.93	93.25	0.92	0.92	0.92
Brain Putamen basal ganglia	0.94	94.25	0.92	0.91	0.91
Brain Spinal cord cervical c-1	0.96	96.25	0.96	0.96	0.96
Brain Substantia nigra	0.95	94.75	0.95	0.96	0.95
Cells EBV-transformed lymphocytes	0.94	94.25	0.94	0.94	0.94
Cells Transformed fibroblasts	0.95	95.25	0.95	0.95	0.95
Colon Sigmoid	0.95	95.00	0.94	0.95	0.95
Colon Transverse	0.94	94.50	0.94	0.95	0.94
Esophagus Gastroesophageal Junction	0.96	95.75	0.95	0.95	0.95
Esophagus Mucosa	0.95	95.25	0.95	0.97	0.95
Esophagus Muscularis	0.94	94.00	0.93	0.93	0.93
Heart Atrial Appendage	0.95	95.00	0.94	0.93	0.93
Heart Left Ventricle	0.94	94.00	0.93	0.94	0.93
Liver	0.94	93.75	0.93	0.92	0.93
Lung	0.95	94.75	0.95	0.95	0.95
Minor Salivary Gland	0.94	94.25	0.94	0.93	0.94
Muscle Skeletal	0.95	95.50	0.95	0.92	0.92
Nerve Tibial	0.95	94.75	0.94	0.93	0.93
Pancreas	0.95	95.00	0.94	0.96	0.95
Pituitary	0.93	93.50	0.93	0.91	0.92
Skin Not Sun Exposed Suprapubic	0.91	91.50	0.92	0.90	0.91
Skin Sun Exposed Lower leg	0.95	95.75	0.95	0.94	0.94
Small Intestine Terminal Ileum	0.94	94.50	0.94	0.95	0.94
Spleen	0.93	93.25	0.93	0.95	0.94
Stomach	0.95	95.00	0.95	0.95	0.95
Thyroid	0.95	95.25	0.95	0.93	0.94
Whole Blood	0.94	94.50	0.94	0.95	0.94

2.3 ADNI1 GWAS single-tissue models training scores

Tissue	ADNI1-GWAS - tissue 10CV Mean Scores				
	AUC	Accuracy	Precision	Recall	F1
Adipose Subcutaneus	0.95	96.17	0.96	0.99	0.97
Adipose Visceral Omentum	0.91	91.83	0.91	0.97	0.94
Adrenal Gland	0.95	95.50	0.96	0.99	0.97
Artery Aorta	0.90	91.00	0.91	0.97	0.94
Artery Coronary	0.84	87.00	0.87	0.96	0.91
Artery Tibial	0.85	87.17	0.86	0.99	0.91
Brain Amygdala	0.91	91.00	0.93	0.93	0.93
Brain Anterior cingulate cortex BA24	0.83	81.50	0.94	0.78	0.79
Brain Caudate basal ganglia	0.90	90.83	0.93	0.95	0.93
Brain Cerebellar Hemisphere	0.83	82.50	0.81	0.84	0.81
Brain Cerebellum	0.88	87.50	0.93	0.89	0.89
Brain Cortex	0.94	94.33	0.94	0.97	0.96
Brain Frontal Cortex BA9	0.94	94.67	0.95	0.98	0.96
Brain Hippocampus	0.90	90.67	0.93	0.93	0.93
Brain Hypothalamus	0.90	88.33	0.91	0.92	0.90
Brain Nucleus accumbens basal ganglia	0.94	94.17	0.95	0.98	0.96
Brain Putamen basal ganglia	0.95	95.17	0.96	0.96	0.96
Brain Spinal cord cervical c-1	0.94	94.33	0.95	0.96	0.95
Brain Substantia nigra	0.94	95.00	0.95	0.98	0.96
Cells EBV-transformed lymphocytes	0.94	94.50	0.94	0.97	0.96
Cells Transformed fibroblasts	0.92	93.00	0.94	0.97	0.95
Colon Sigmoid	0.94	94.83	0.95	0.98	0.96
Colon Transverse	0.95	95.50	0.96	0.99	0.97
Esophagus Gastroesophageal Junction	0.92	92.50	0.96	0.93	0.93
Esophagus Mucosa	0.93	94.00	0.93	0.99	0.96
Esophagus Muscularis	0.94	95.17	0.95	0.99	0.97
Heart Atrial Appendage	0.93	94.00	0.95	0.97	0.96
Heart Left Ventricle	0.93	94.00	0.94	0.98	0.96
Liver	0.95	95.83	0.96	0.99	0.97
Lung	0.94	95.00	0.95	0.98	0.96
Minor Salivary Gland	0.91	91.83	0.92	0.98	0.94
Muscle Skeletal	0.93	93.83	0.93	0.99	0.96
Nerve Tibial	0.94	94.50	0.94	0.98	0.96
Pancreas	0.95	96.00	0.96	0.99	0.97
Pituitary	0.88	89.83	0.89	0.97	0.92
Skin Not Sun Exposed Suprapubic	0.92	92.67	0.92	0.98	0.95
Skin Sun Exposed Lower leg	0.92	93.17	0.93	0.98	0.95
Small Intestine Terminal Ileum	0.95	95.33	0.96	0.98	0.96
Spleen	0.93	94.50	0.94	0.99	0.96
Stomach	0.92	93.17	0.93	0.98	0.95
Thyroid	0.94	95.00	0.96	0.98	0.97
Whole Blood	0.95	95.33	0.96	0.97	0.96

Tissue	ADNI1-GWAS - tissue UnList 10CV Mean Scores				
	AUC	Accuracy	Precision	Recall	F1
Adipose Subcutaneus	0.90	89.83	0.94	0.90	0.91
Adipose Visceral Omentum	0.93	93.67	0.95	0.94	0.94
Adrenal Gland	0.91	91.67	0.92	0.94	0.93
Artery Aorta	0.94	94.83	0.95	0.97	0.96
Artery Coronary	0.93	93.33	0.94	0.95	0.94
Artery Tibial	0.96	96.00	0.96	0.97	0.97
Brain Amygdala	0.89	89.50	0.91	0.93	0.91
Brain Anterior cingulate cortex BA24	0.92	91.83	0.93	0.93	0.93
Brain Caudate basal ganglia	0.92	93.17	0.93	0.96	0.95
Brain Cerebellar Hemisphere	0.94	94.83	0.95	0.96	0.96
Brain Cerebellum	0.94	94.17	0.95	0.94	0.95
Brain Cortex	0.93	93.17	0.94	0.95	0.95
Brain Frontal Cortex BA9	0.93	94.00	0.95	0.96	0.95
Brain Hippocampus	0.90	90.83	0.92	0.92	0.92
Brain Hypothalamus	0.93	93.00	0.94	0.94	0.94
Brain Nucleus accumbens basal ganglia	0.93	93.83	0.95	0.96	0.93
Brain Putamen basal ganglia	0.92	92.17	0.93	0.94	0.93
Brain Spinal cord cervical c-1	0.92	92.67	0.95	0.93	0.94
Brain Substantia nigra	0.91	91.00	0.93	0.93	0.93
Cells EBV-transformed lymphocytes	0.94	94.50	0.95	0.95	0.95
Cells Transformed fibroblasts	0.92	92.67	0.94	0.93	0.94
Colon Sigmoid	0.91	91.83	0.93	0.94	0.93
Colon Transverse	0.94	94.00	0.95	0.94	0.95
Esophagus Gastroesophageal Junction	0.93	94.00	0.94	0.97	0.95
Esophagus Mucosa	0.94	94.67	0.95	0.97	0.96
Esophagus Muscularis	0.93	93.67	0.94	0.94	0.94
Heart Atrial Appendage	0.94	94.67	0.95	0.97	0.96
Heart Left Ventricle	0.94	94.83	0.95	0.97	0.96
Liver	0.92	92.33	0.93	0.95	0.94
Lung	0.94	94.33	0.95	0.96	0.95
Minor Salivary Gland	0.82	84.83	0.85	0.94	0.89
Muscle Skeletal	0.93	94.00	0.95	0.95	0.95
Nerve Tibial	0.94	94.50	0.96	0.95	0.95
Pancreas	0.94	95.00	0.95	0.97	0.96
Pituitary	0.94	94.50	0.95	0.96	0.95
Skin Not Sun Exposed Suprapubic	0.93	93.33	0.95	0.93	0.94
Skin Sun Exposed Lower leg	0.95	95.33	0.95	0.99	0.97
Small Intestine Terminal Ileum	0.94	94.33	0.95	0.96	0.95
Spleen	0.93	93.83	0.94	0.95	0.95
Stomach	0.91	92.00	0.92	0.94	0.93
Thyroid	0.93	93.33	0.95	0.94	0.94
Whole Blood	0.94	93.83	0.96	0.93	0.94

Tissue	ADNI1-GWAS - tissue PredixList 10CV Mean Scores				
	AUC	Accuracy	Precision	Recall	F1
Adipose Subcutaneus	0.95	95.17	0.96	0.96	0.96
Adipose Visceral Omentum	0.94	94.33	0.95	0.95	0.95
Adrenal Gland	0.96	95.83	0.97	0.96	0.96
Artery Aorta	0.95	95.33	0.96	0.97	0.96
Artery Coronary	0.95	95.17	0.95	0.96	0.96
Artery Tibial	0.95	95.00	0.95	0.96	0.96
Brain Amygdala	0.93	93.50	0.94	0.95	0.94
Brain Anterior cingulate cortex BA24	0.94	94.83	0.95	0.97	0.96
Brain Caudate basal ganglia	0.96	96.17	0.97	0.97	0.97
Brain Cerebellar Hemisphere	0.94	94.67	0.96	0.95	0.95
Brain Cerebellum	0.94	94.17	0.95	0.95	0.95
Brain Cortex	0.93	93.83	0.94	0.96	0.95
Brain Frontal Cortex BA9	0.94	94.67	0.96	0.95	0.95
Brain Hippocampus	0.95	94.83	0.95	0.96	0.95
Brain Hypothalamus	0.93	93.83	0.95	0.95	0.95
Brain Nucleus accumbens basal ganglia	0.95	95.17	0.96	0.96	0.96
Brain Putamen basal ganglia	0.96	96.50	0.97	0.98	0.97
Brain Spinal cord cervical c-1	0.93	94.00	0.94	0.96	0.95
Brain Substantia nigra	0.94	94.33	0.95	0.96	0.95
Cells EBV-transformed lymphocytes	0.95	95.33	0.96	0.97	0.96
Cells Transformed fibroblasts	0.95	95.17	0.96	0.96	0.96
Colon Sigmoid	0.94	94.17	0.95	0.95	0.95
Colon Transverse	0.96	95.83	0.97	0.96	0.96
Esophagus Gastroesophageal Junction	0.94	94.50	0.96	0.95	0.95
Esophagus Mucosa	0.93	93.83	0.95	0.95	0.95
Esophagus Muscularis	0.95	95.83	0.96	0.97	0.97
Heart Atrial Appendage	0.95	95.50	0.96	0.96	0.96
Heart Left Ventricle	0.95	95.33	0.96	0.96	0.96
Liver	0.94	94.17	0.95	0.95	0.95
Lung	0.95	95.50	0.96	0.96	0.96
Minor Salivary Gland	0.95	94.83	0.96	0.95	0.95
Muscle Skeletal	0.95	95.17	0.96	0.97	0.96
Nerve Tibial	0.96	96.00	0.96	0.97	0.97
Pancreas	0.94	94.00	0.95	0.95	0.95
Pituitary	0.95	95.00	0.97	0.95	0.96
Skin Not Sun Exposed Suprapubic	0.95	95.67	0.96	0.97	0.96
Skin Sun Exposed Lower leg	0.95	95.50	0.96	0.96	0.96
Small Intestine Terminal Ileum	0.95	95.00	0.96	0.96	0.96
Spleen	0.95	95.50	0.96	0.97	0.96
Stomach	0.95	95.50	0.96	0.96	0.96
Thyroid	0.94	93.83	0.95	0.93	0.94
Whole Blood	0.95	95.00	0.96	0.95	0.96

2.4 Cognitive Decline single-tissue models training scores

Tissue	Cognitive Decline-tissue 10CV Mean Scores				
	AUC	Accuracy	Precision	Recall	F1
Adipose Subcutaneus	0.95	94.50	0.94	0.99	0.96
Adipose Visceral Omentum	0.95	95.25	0.95	0.95	0.95
Adrenal Gland	0.95	95.00	0.95	0.95	0.95
Artery Aorta	0.95	95.75	0.96	0.92	0.93
Artery Coronary	0.93	92.50	0.94	0.91	0.92
Artery Tibial	0.95	95.50	0.95	0.95	0.95
Brain Amygdala	0.92	91.75	0.91	0.92	0.92
Brain Anterior cingulate cortex BA24	0.94	93.75	0.93	0.94	0.93
Brain Caudate basal ganglia	0.94	94.25	0.95	0.91	0.93
Brain Cerebellar Hemisphere	0.95	95.00	0.95	0.94	0.95
Brain Cerebellum	0.96	96.00	0.96	0.94	0.95
Brain Cortex	0.95	95.50	0.95	0.94	0.95
Brain Frontal Cortex BA9	0.94	94.00	0.96	0.91	0.93
Brain Hippocampus	0.93	93.50	0.92	0.92	0.92
Brain Hypothalamus	0.95	94.75	0.94	0.95	0.95
Brain Nucleus accumbens basal ganglia	0.93	93.50	0.95	0.90	0.92
Brain Putamen basal ganglia	0.96	95.75	0.95	0.95	0.95
Brain Spinal cord cervical c-1	0.92	91.75	0.90	0.95	0.92
Brain Substantia nigra	0.95	95.25	0.94	0.94	0.94
Cells EBV-transformed lymphocytes	0.94	94.25	0.94	0.95	0.94
Cells Transformed fibroblasts	0.95	95.00	0.95	0.94	0.95
Colon Sigmoid	0.96	96.25	0.97	0.93	0.95
Colon Transverse	0.93	93.50	0.91	0.93	0.91
Esophagus Gastroesophageal Junction	0.95	95.25	0.95	0.97	0.96
Esophagus Mucosa	0.94	94.75	0.93	0.92	0.92
Esophagus Muscularis	0.95	95.50	0.95	0.95	0.95
Heart Atrial Appendage	0.95	95.00	0.94	0.94	0.94
Heart Left Ventricle	0.94	94.50	0.94	0.92	0.93
Liver	0.94	93.75	0.94	0.94	0.94
Lung	0.94	94.75	0.94	0.92	0.93
Minor Salivary Gland	0.95	95.50	0.95	0.92	0.93
Muscle Skeletal	0.94	94.00	0.94	0.94	0.94
Nerve Tibial	0.93	93.50	0.93	0.95	0.94
Pancreas	0.95	95.00	0.94	0.95	0.95
Pituitary	0.94	94.00	0.95	0.94	0.94
Skin Not Sun Exposed Suprapubic	0.95	95.25	0.95	0.95	0.95
Skin Sun Exposed Lower leg	0.94	94.50	0.94	0.91	0.93
Small Intestine Terminal Ileum	0.92	91.50	0.89	0.93	0.91
Spleen	0.95	95.50	0.95	0.93	0.94
Stomach	0.96	96.75	0.97	0.95	0.96
Thyroid	0.95	95.25	0.95	0.94	0.94
Whole Blood	0.95	95.50	0.95	0.93	0.94

Tissue	Cognitive Decline-tissue UnList 10CV Mean Scores				
	AUC	Accuracy	Precision	Recall	F1
Adipose Subcutaneus	0.95	94.75	0.94	0.95	0.94
Adipose Visceral Omentum	0.92	92.50	0.91	0.93	0.92
Adrenal Gland	0.93	93.25	0.93	0.93	0.93
Artery Aorta	0.95	95.50	0.95	0.94	0.95
Artery Coronary	0.92	92.25	0.93	0.92	0.92
Artery Tibial	0.95	95.50	0.95	0.94	0.95
Brain Amygdala	0.91	90.50	0.89	0.92	0.90
Brain Anterior cingulate cortex BA24	0.93	93.50	0.92	0.93	0.93
Brain Caudate basal ganglia	0.93	93.50	0.93	0.95	0.94
Brain Cerebellar Hemisphere	0.94	94.00	0.93	0.92	0.92
Brain Cerebellum	0.93	93.25	0.95	0.90	0.92
Brain Cortex	0.94	94.50	0.93	0.94	0.94
Brain Frontal Cortex BA9	0.94	94.25	0.93	0.94	0.94
Brain Hippocampus	0.89	89.25	0.89	0.89	0.88
Brain Hypothalamus	0.92	92.75	0.95	0.88	0.91
Brain Nucleus accumbens basal ganglia	0.94	94.50	0.95	0.94	0.94
Brain Putamen basal ganglia	0.92	92.50	0.93	0.89	0.90
Brain Spinal cord cervical c-1	0.94	93.75	0.93	0.94	0.94
Brain Substantia nigra	0.92	92.25	0.92	0.90	0.91
Cells EBV-transformed lymphocytes	0.94	94.50	0.94	0.95	0.94
Cells Transformed fibroblasts	0.94	93.75	0.94	0.93	0.93
Colon Sigmoid	0.92	92.00	0.91	0.91	0.91
Colon Transverse	0.93	93.75	0.93	0.91	0.92
Esophagus Gastroesophageal Junction	0.92	92.50	0.91	0.89	0.90
Esophagus Mucosa	0.95	94.75	0.94	0.97	0.95
Esophagus Muscularis	0.94	94.25	0.94	0.93	0.94
Heart Atrial Appendage	0.96	96.25	0.96	0.96	0.96
Heart Left Ventricle	0.93	93.25	0.93	0.89	0.91
Liver	0.93	93.25	0.93	0.91	0.92
Lung	0.95	95.50	0.96	0.94	0.95
Minor Salivary Gland	0.88	88.00	0.86	0.89	0.87
Muscle Skeletal	0.95	95.00	0.94	0.95	0.95
Nerve Tibial	0.94	93.75	0.94	0.95	0.94
Pancreas	0.94	93.75	0.94	0.94	0.94
Pituitary	0.95	94.75	0.95	0.94	0.94
Skin Not Sun Exposed Suprapubic	0.95	95.00	0.95	0.95	0.95
Skin Sun Exposed Lower leg	0.97	96.75	0.96	0.97	0.97
Small Intestine Terminal Ileum	0.95	95.50	0.95	0.96	0.95
Spleen	0.94	93.75	0.93	0.94	0.93
Stomach	0.94	93.75	0.94	0.95	0.94
Thyroid	0.94	94.50	0.94	0.95	0.94
Whole Blood	0.95	95.25	0.95	0.94	0.94

Tissue	Cognitive Decline-tissue PredixList 10CV Mean Scores				
	AUC	Accuracy	Precision	Recall	F1
Adipose Subcutaneus	0.94	94.75	0.94	0.93	0.93
Adipose Visceral Omentum	0.96	96.00	0.96	0.95	0.95
Adrenal Gland	0.95	95.50	0.95	0.96	0.95
Artery Aorta	0.95	94.75	0.94	0.93	0.94
Artery Coronary	0.94	94.75	0.94	0.92	0.93
Artery Tibial	0.93	93.25	0.92	0.94	0.93
Brain Amygdala	0.94	93.75	0.94	0.94	0.94
Brain Anterior cingulate cortex BA24	0.96	96.25	0.96	0.95	0.95
Brain Caudate basal ganglia	0.94	94.50	0.94	0.93	0.94
Brain Cerebellar Hemisphere	0.96	96.00	0.95	0.96	0.96
Brain Cerebellum	0.93	93.25	0.93	0.92	0.92
Brain Cortex	0.95	94.75	0.94	0.94	0.94
Brain Frontal Cortex BA9	0.96	95.75	0.95	0.95	0.95
Brain Hippocampus	0.93	93.25	0.93	0.92	0.93
Brain Hypothalamus	0.95	95.25	0.95	0.95	0.95
Brain Nucleus accumbens basal ganglia	0.95	94.75	0.94	0.94	0.94
Brain Putamen basal ganglia	0.94	94.00	0.93	0.93	0.93
Brain Spinal cord cervical c-1	0.95	95.25	0.95	0.95	0.95
Brain Substantia nigra	0.95	95.25	0.95	0.95	0.95
Cells EBV-transformed lymphocytes	0.94	94.50	0.94	0.94	0.94
Cells Transformed fibroblasts	0.95	95.25	0.94	0.94	0.94
Colon Sigmoid	0.95	94.75	0.94	0.95	0.95
Colon Transverse	0.94	93.75	0.94	0.95	0.94
Esophagus Gastroesophageal Junction	0.94	93.75	0.95	0.92	0.93
Esophagus Mucosa	0.95	95.25	0.94	0.94	0.94
Esophagus Muscularis	0.94	94.00	0.93	0.95	0.94
Heart Atrial Appendage	0.95	95.50	0.95	0.96	0.95
Heart Left Ventricle	0.93	93.25	0.92	0.92	0.92
Liver	0.94	94.75	0.95	0.92	0.93
Lung	0.96	95.75	0.95	0.94	0.95
Minor Salivary Gland	0.94	94.25	0.94	0.93	0.94
Muscle Skeletal	0.94	94.25	0.94	0.95	0.94
Nerve Tibial	0.94	94.25	0.93	0.94	0.94
Pancreas	0.95	95.00	0.94	0.93	0.94
Pituitary	0.94	94.00	0.94	0.93	0.93
Skin Not Sun Exposed Suprapubic	0.95	95.50	0.95	0.95	0.95
Skin Sun Exposed Lower leg	0.93	93.25	0.92	0.91	0.92
Small Intestine Terminal Ileum	0.95	94.75	0.95	0.93	0.94
Spleen	0.94	94.50	0.94	0.94	0.94
Stomach	0.96	95.75	0.95	0.94	0.95
Thyroid	0.96	95.75	0.95	0.97	0.96
Whole Blood	0.94	93.75	0.93	0.93	0.93

2.5 ROC Curve: ADNI1-GWAS

Here are reported the ROC curve obtained for each ADNI1-GWAS Brain tissue model during the training and test, for cross-tissue (first and second row) and single-tissue analysis (third and fourth row). By column are reported the models without feature selection (left column), with List-unsupervised (middle column) and with List-PrediXcan selection (right column).

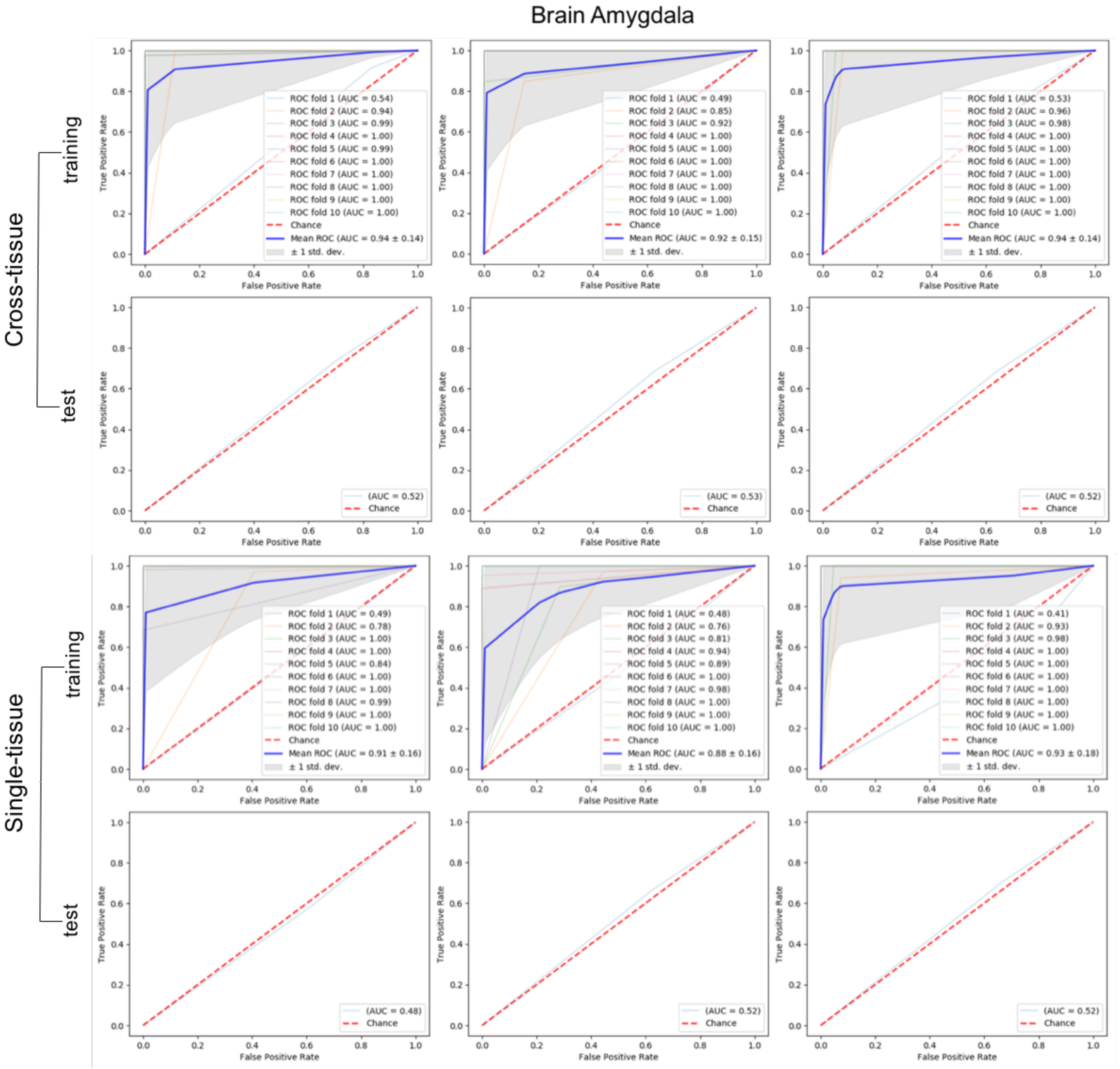

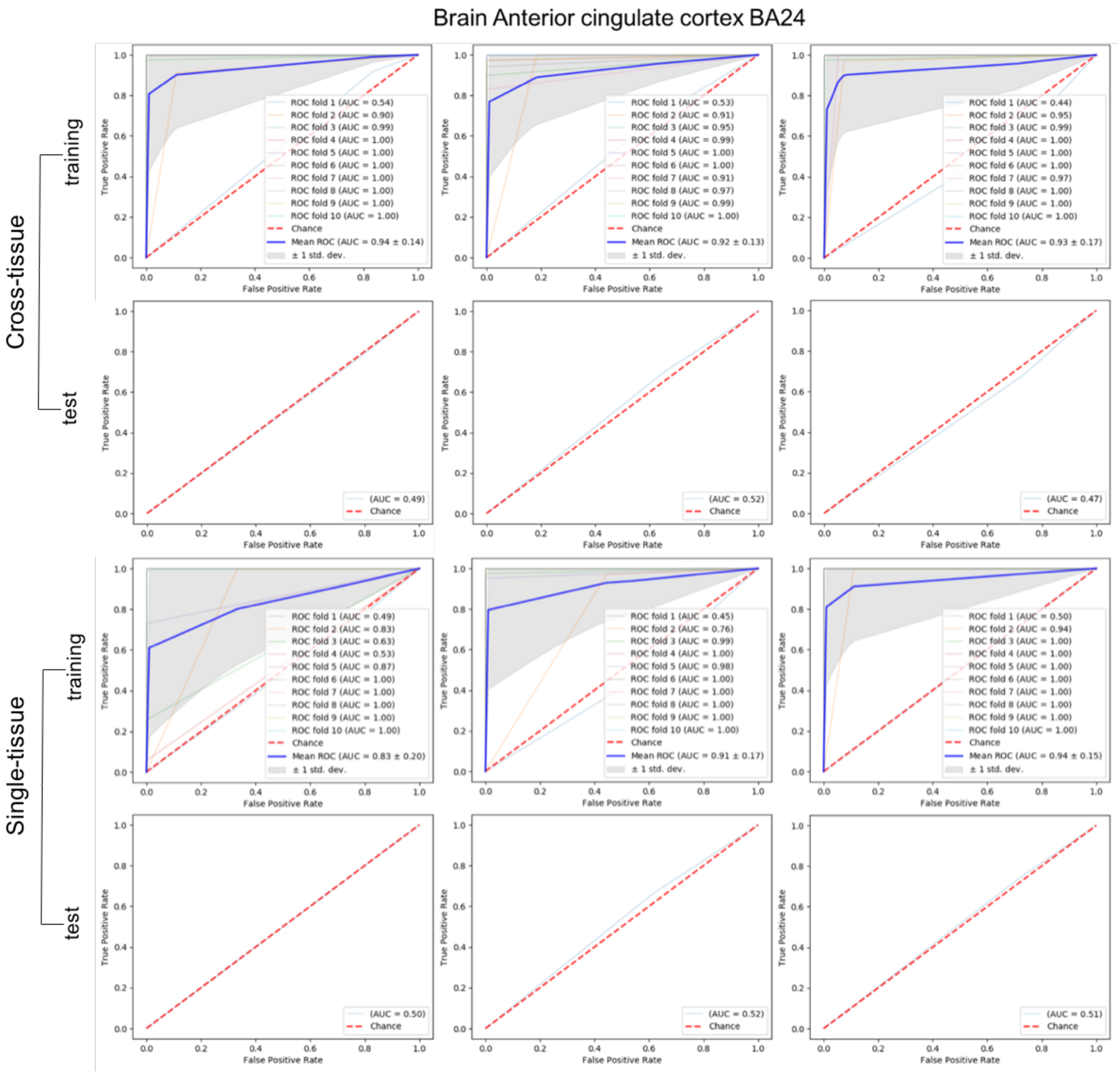

Brain Anterior cingulate cortex BA24
Cross-tissue
training
test
Single-tissue
training
test
True Positive Rate
False Positive Rate
ROC fold 1 (AUC = 0.54)
ROC fold 2 (AUC = 0.90)
ROC fold 3 (AUC = 0.99)
ROC fold 4 (AUC = 1.00)
ROC fold 5 (AUC = 1.00)
ROC fold 6 (AUC = 1.00)
ROC fold 7 (AUC = 1.00)
ROC fold 8 (AUC = 1.00)
ROC fold 9 (AUC = 1.00)
ROC fold 10 (AUC = 1.00)
Chance
Mean ROC (AUC = 0.94 ± 0.14)
± 1 std. dev.
ROC fold 1 (AUC = 0.53)
ROC fold 2 (AUC = 0.91)
ROC fold 3 (AUC = 0.95)
ROC fold 4 (AUC = 0.99)
ROC fold 5 (AUC = 1.00)
ROC fold 6 (AUC = 1.00)
ROC fold 7 (AUC = 0.91)
ROC fold 8 (AUC = 0.97)
ROC fold 9 (AUC = 0.99)
ROC fold 10 (AUC = 1.00)
Chance
Mean ROC (AUC = 0.92 ± 0.13)
± 1 std. dev.
ROC fold 1 (AUC = 0.44)
ROC fold 2 (AUC = 0.95)
ROC fold 3 (AUC = 0.99)
ROC fold 4 (AUC = 1.00)
ROC fold 5 (AUC = 1.00)
ROC fold 6 (AUC = 1.00)
ROC fold 7 (AUC = 0.97)
ROC fold 8 (AUC = 1.00)
ROC fold 9 (AUC = 1.00)
ROC fold 10 (AUC = 1.00)
Chance
Mean ROC (AUC = 0.93 ± 0.17)
± 1 std. dev.
(AUC = 0.49)
Chance
(AUC = 0.52)
Chance
(AUC = 0.47)
Chance
ROC fold 1 (AUC = 0.49)
ROC fold 2 (AUC = 0.83)
ROC fold 3 (AUC = 0.63)
ROC fold 4 (AUC = 0.53)
ROC fold 5 (AUC = 0.87)
ROC fold 6 (AUC = 1.00)
ROC fold 7 (AUC = 1.00)
ROC fold 8 (AUC = 1.00)
ROC fold 9 (AUC = 1.00)
ROC fold 10 (AUC = 1.00)
Chance
Mean ROC (AUC = 0.83 ± 0.20)
± 1 std. dev.
ROC fold 1 (AUC = 0.45)
ROC fold 2 (AUC = 0.76)
ROC fold 3 (AUC = 0.99)
ROC fold 4 (AUC = 1.00)
ROC fold 5 (AUC = 0.98)
ROC fold 6 (AUC = 1.00)
ROC fold 7 (AUC = 1.00)
ROC fold 8 (AUC = 1.00)
ROC fold 9 (AUC = 1.00)
ROC fold 10 (AUC = 1.00)
Chance
Mean ROC (AUC = 0.91 ± 0.17)
± 1 std. dev.
ROC fold 1 (AUC = 0.50)
ROC fold 2 (AUC = 0.94)
ROC fold 3 (AUC = 1.00)
ROC fold 4 (AUC = 1.00)
ROC fold 5 (AUC = 1.00)
ROC fold 6 (AUC = 1.00)
ROC fold 7 (AUC = 1.00)
ROC fold 8 (AUC = 1.00)
ROC fold 9 (AUC = 1.00)
ROC fold 10 (AUC = 1.00)
Chance
Mean ROC (AUC = 0.94 ± 0.15)
± 1 std. dev.
(AUC = 0.50)
Chance
(AUC = 0.52)
Chance
(AUC = 0.51)
Chance

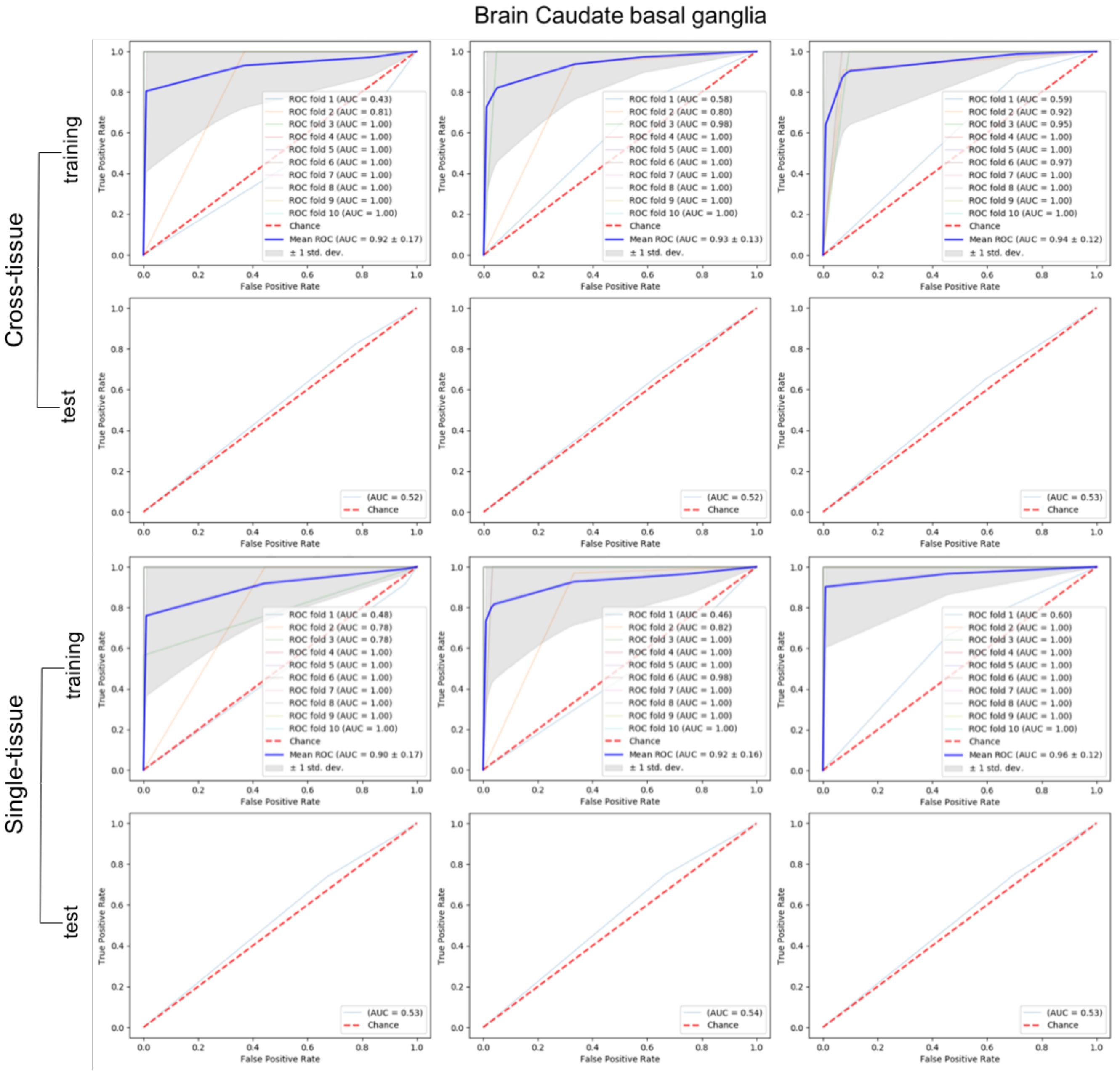
Brain Caudate basal ganglia
Cross-tissue
training
test
Single-tissue
training
test
True Positive Rate
False Positive Rate
ROC fold 1 (AUC = 0.43)
ROC fold 2 (AUC = 0.81)
ROC fold 3 (AUC = 1.00)
ROC fold 4 (AUC = 1.00)
ROC fold 5 (AUC = 1.00)
ROC fold 6 (AUC = 1.00)
ROC fold 7 (AUC = 1.00)
ROC fold 8 (AUC = 1.00)
ROC fold 9 (AUC = 1.00)
ROC fold 10 (AUC = 1.00)
Chance
Mean ROC (AUC = 0.92 ± 0.17)
± 1 std. dev.
ROC fold 1 (AUC = 0.58)
ROC fold 2 (AUC = 0.80)
ROC fold 3 (AUC = 0.98)
ROC fold 4 (AUC = 1.00)
ROC fold 5 (AUC = 1.00)
ROC fold 6 (AUC = 1.00)
ROC fold 7 (AUC = 1.00)
ROC fold 8 (AUC = 1.00)
ROC fold 9 (AUC = 1.00)
ROC fold 10 (AUC = 1.00)
Chance
Mean ROC (AUC = 0.93 ± 0.13)
± 1 std. dev.
ROC fold 1 (AUC = 0.59)
ROC fold 2 (AUC = 0.92)
ROC fold 3 (AUC = 0.95)
ROC fold 4 (AUC = 1.00)
ROC fold 5 (AUC = 1.00)
ROC fold 6 (AUC = 0.97)
ROC fold 7 (AUC = 1.00)
ROC fold 8 (AUC = 1.00)
ROC fold 9 (AUC = 1.00)
ROC fold 10 (AUC = 1.00)
Chance
Mean ROC (AUC = 0.94 ± 0.12)
± 1 std. dev.
(AUC = 0.52)
Chance
(AUC = 0.52)
Chance
(AUC = 0.53)
Chance
ROC fold 1 (AUC = 0.48)
ROC fold 2 (AUC = 0.78)
ROC fold 3 (AUC = 0.78)
ROC fold 4 (AUC = 1.00)
ROC fold 5 (AUC = 1.00)
ROC fold 6 (AUC = 1.00)
ROC fold 7 (AUC = 1.00)
ROC fold 8 (AUC = 1.00)
ROC fold 9 (AUC = 1.00)
ROC fold 10 (AUC = 1.00)
Chance
Mean ROC (AUC = 0.90 ± 0.17)
± 1 std. dev.
ROC fold 1 (AUC = 0.46)
ROC fold 2 (AUC = 0.82)
ROC fold 3 (AUC = 1.00)
ROC fold 4 (AUC = 1.00)
ROC fold 5 (AUC = 1.00)
ROC fold 6 (AUC = 0.98)
ROC fold 7 (AUC = 1.00)
ROC fold 8 (AUC = 1.00)
ROC fold 9 (AUC = 1.00)
ROC fold 10 (AUC = 1.00)
Chance
Mean ROC (AUC = 0.92 ± 0.16)
± 1 std. dev.
ROC fold 1 (AUC = 0.60)
ROC fold 2 (AUC = 1.00)
ROC fold 3 (AUC = 1.00)
ROC fold 4 (AUC = 1.00)
ROC fold 5 (AUC = 1.00)
ROC fold 6 (AUC = 1.00)
ROC fold 7 (AUC = 1.00)
ROC fold 8 (AUC = 1.00)
ROC fold 9 (AUC = 1.00)
ROC fold 10 (AUC = 1.00)
Chance
Mean ROC (AUC = 0.96 ± 0.12)
± 1 std. dev.
(AUC = 0.53)
Chance
(AUC = 0.54)
Chance
(AUC = 0.53)
Chance

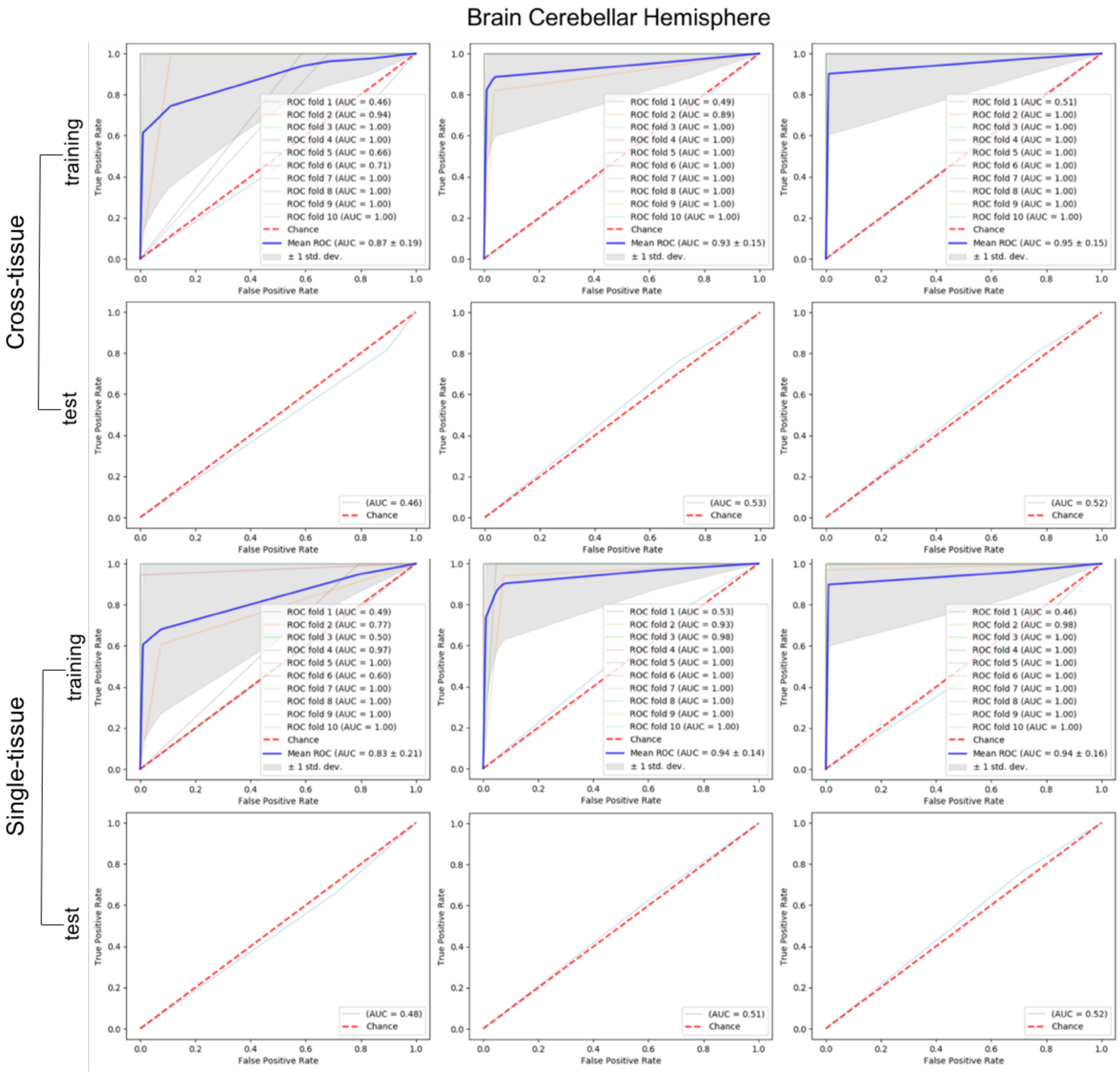
Brain Cerebellar Hemisphere
Cross-tissue
training
test
Single-tissue
training
test
True Positive Rate
False Positive Rate
ROC fold 1 (AUC = 0.46)
ROC fold 2 (AUC = 0.94)
ROC fold 3 (AUC = 1.00)
ROC fold 4 (AUC = 1.00)
ROC fold 5 (AUC = 0.66)
ROC fold 6 (AUC = 0.71)
ROC fold 7 (AUC = 1.00)
ROC fold 8 (AUC = 1.00)
ROC fold 9 (AUC = 1.00)
ROC fold 10 (AUC = 1.00)
Chance
Mean ROC (AUC = 0.87 ± 0.19)
± 1 std. dev.
ROC fold 1 (AUC = 0.49)
ROC fold 2 (AUC = 0.89)
ROC fold 3 (AUC = 1.00)
ROC fold 4 (AUC = 1.00)
ROC fold 5 (AUC = 1.00)
ROC fold 6 (AUC = 1.00)
ROC fold 7 (AUC = 1.00)
ROC fold 8 (AUC = 1.00)
ROC fold 9 (AUC = 1.00)
ROC fold 10 (AUC = 1.00)
Chance
Mean ROC (AUC = 0.93 ± 0.15)
± 1 std. dev.
ROC fold 1 (AUC = 0.51)
ROC fold 2 (AUC = 1.00)
ROC fold 3 (AUC = 1.00)
ROC fold 4 (AUC = 1.00)
ROC fold 5 (AUC = 1.00)
ROC fold 6 (AUC = 1.00)
ROC fold 7 (AUC = 1.00)
ROC fold 8 (AUC = 1.00)
ROC fold 9 (AUC = 1.00)
ROC fold 10 (AUC = 1.00)
Chance
Mean ROC (AUC = 0.95 ± 0.15)
± 1 std. dev.
(AUC = 0.46)
Chance
(AUC = 0.53)
Chance
(AUC = 0.52)
Chance
ROC fold 1 (AUC = 0.49)
ROC fold 2 (AUC = 0.77)
ROC fold 3 (AUC = 0.50)
ROC fold 4 (AUC = 0.97)
ROC fold 5 (AUC = 1.00)
ROC fold 6 (AUC = 0.60)
ROC fold 7 (AUC = 1.00)
ROC fold 8 (AUC = 1.00)
ROC fold 9 (AUC = 1.00)
ROC fold 10 (AUC = 1.00)
Chance
Mean ROC (AUC = 0.83 ± 0.21)
± 1 std. dev.
ROC fold 1 (AUC = 0.53)
ROC fold 2 (AUC = 0.93)
ROC fold 3 (AUC = 0.98)
ROC fold 4 (AUC = 1.00)
ROC fold 5 (AUC = 1.00)
ROC fold 6 (AUC = 1.00)
ROC fold 7 (AUC = 1.00)
ROC fold 8 (AUC = 1.00)
ROC fold 9 (AUC = 1.00)
ROC fold 10 (AUC = 1.00)
Chance
Mean ROC (AUC = 0.94 ± 0.14)
± 1 std. dev.
ROC fold 1 (AUC = 0.46)
ROC fold 2 (AUC = 0.98)
ROC fold 3 (AUC = 1.00)
ROC fold 4 (AUC = 1.00)
ROC fold 5 (AUC = 1.00)
ROC fold 6 (AUC = 1.00)
ROC fold 7 (AUC = 1.00)
ROC fold 8 (AUC = 1.00)
ROC fold 9 (AUC = 1.00)
ROC fold 10 (AUC = 1.00)
Chance
Mean ROC (AUC = 0.94 ± 0.16)
± 1 std. dev.
(AUC = 0.48)
Chance
(AUC = 0.51)
Chance
(AUC = 0.52)
Chance

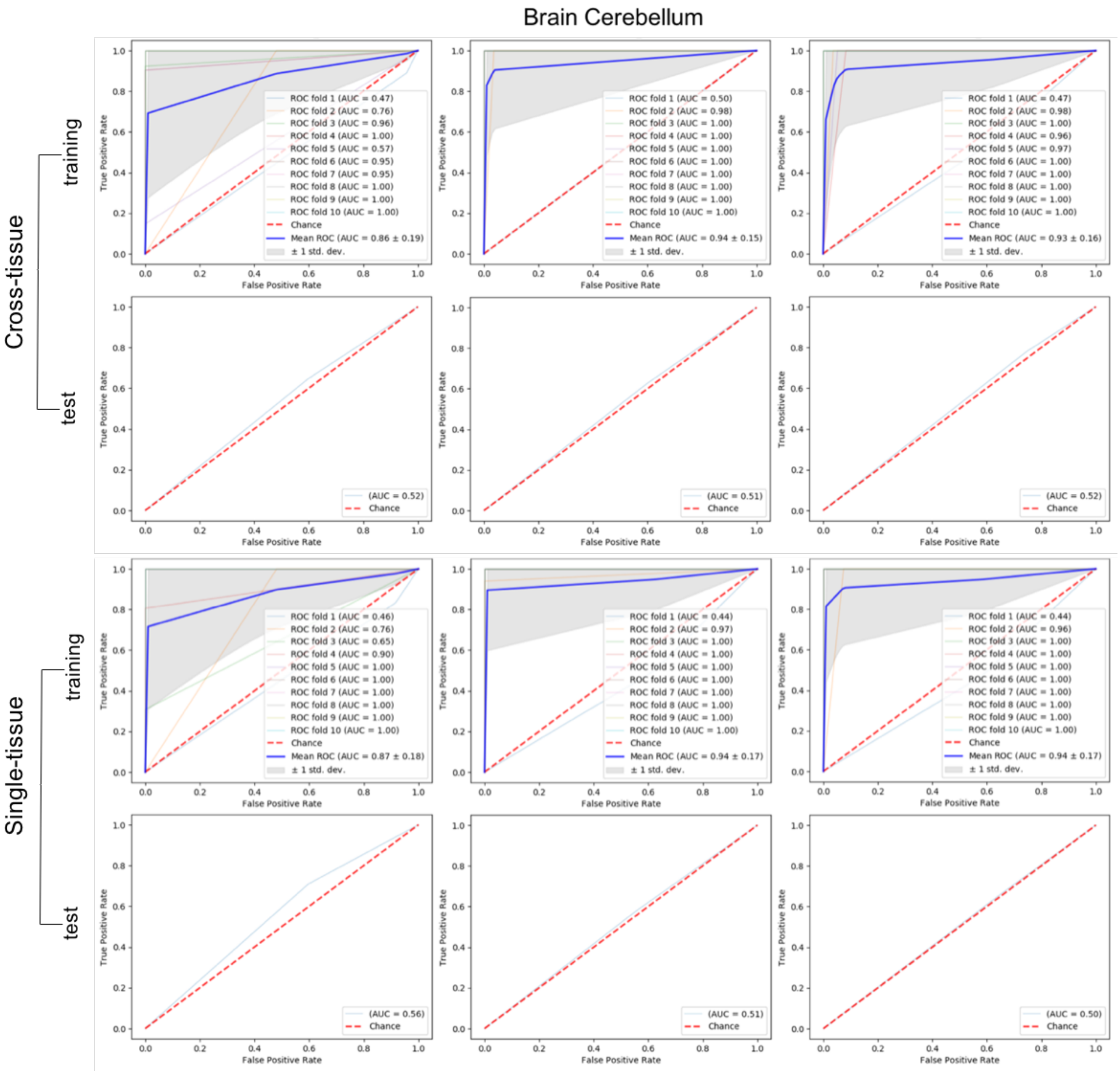
Brain Cerebellum
Cross-tissue
training
test
Single-tissue
training
test

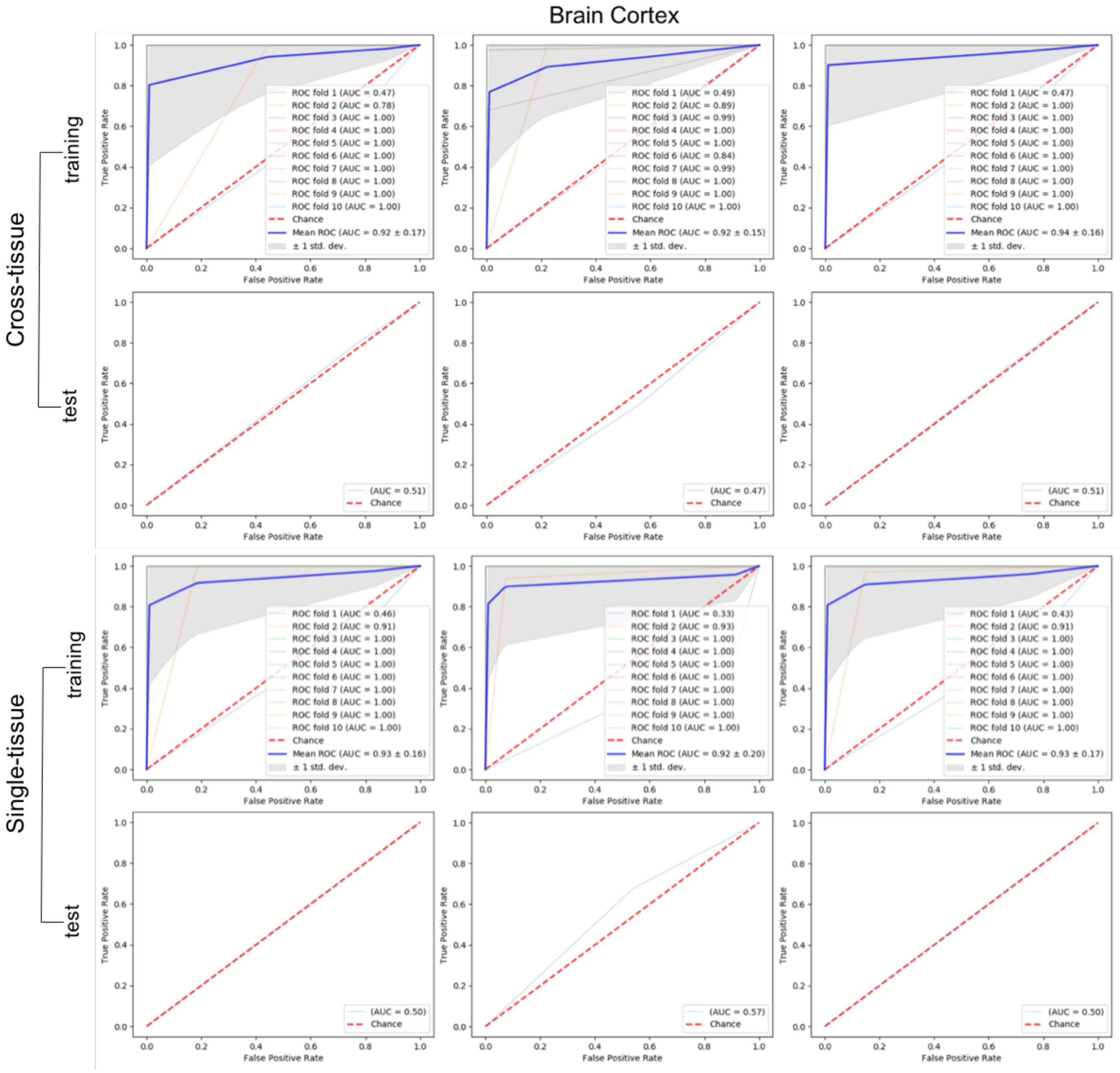
Brain Cortex
Cross-tissue
training
test
Single-tissue
training
test
True Positive Rate
False Positive Rate
ROC fold 1 (AUC = 0.47)
ROC fold 2 (AUC = 0.78)
Mean ROC (AUC = 0.92 ± 0.17)
± 1 std. dev.
Chance
ROC fold 1 (AUC = 0.49)
ROC fold 2 (AUC = 0.89)
ROC fold 3 (AUC = 0.99)
ROC fold 6 (AUC = 0.84)
ROC fold 7 (AUC = 0.99)
Mean ROC (AUC = 0.92 ± 0.15)
Mean ROC (AUC = 0.94 ± 0.16)
(AUC = 0.51)
(AUC = 0.47)
ROC fold 1 (AUC = 0.46)
ROC fold 2 (AUC = 0.91)
Mean ROC (AUC = 0.93 ± 0.16)
ROC fold 1 (AUC = 0.33)
ROC fold 2 (AUC = 0.93)
Mean ROC (AUC = 0.92 ± 0.20)
ROC fold 1 (AUC = 0.43)
Mean ROC (AUC = 0.93 ± 0.17)
(AUC = 0.50)
(AUC = 0.57)

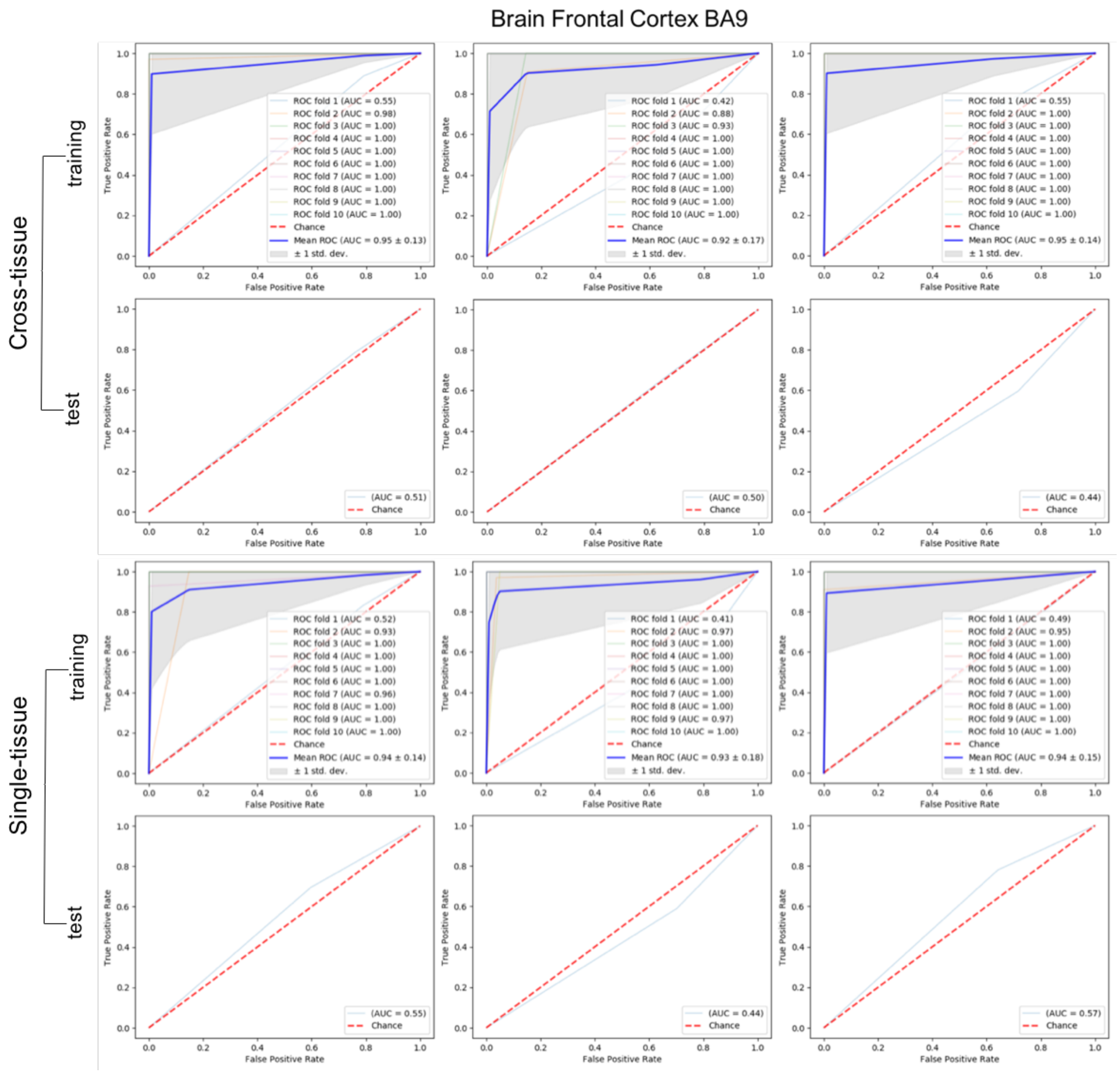
Brain Frontal Cortex BA9
Cross-tissue
training
test
Single-tissue
training
test
True Positive Rate
False Positive Rate
ROC fold 1 (AUC = 0.55)
ROC fold 2 (AUC = 0.98)
ROC fold 3 (AUC = 1.00)
ROC fold 4 (AUC = 1.00)
ROC fold 5 (AUC = 1.00)
ROC fold 6 (AUC = 1.00)
ROC fold 7 (AUC = 1.00)
ROC fold 8 (AUC = 1.00)
ROC fold 9 (AUC = 1.00)
ROC fold 10 (AUC = 1.00)
Chance
Mean ROC (AUC = 0.95 ± 0.13)
± 1 std. dev.
ROC fold 1 (AUC = 0.42)
ROC fold 2 (AUC = 0.88)
ROC fold 3 (AUC = 0.93)
ROC fold 4 (AUC = 1.00)
ROC fold 5 (AUC = 1.00)
ROC fold 6 (AUC = 1.00)
ROC fold 7 (AUC = 1.00)
ROC fold 8 (AUC = 1.00)
ROC fold 9 (AUC = 1.00)
ROC fold 10 (AUC = 1.00)
Chance
Mean ROC (AUC = 0.92 ± 0.17)
± 1 std. dev.
ROC fold 1 (AUC = 0.55)
ROC fold 2 (AUC = 1.00)
ROC fold 3 (AUC = 1.00)
ROC fold 4 (AUC = 1.00)
ROC fold 5 (AUC = 1.00)
ROC fold 6 (AUC = 1.00)
ROC fold 7 (AUC = 1.00)
ROC fold 8 (AUC = 1.00)
ROC fold 9 (AUC = 1.00)
ROC fold 10 (AUC = 1.00)
Chance
Mean ROC (AUC = 0.95 ± 0.14)
± 1 std. dev.
(AUC = 0.51)
Chance
(AUC = 0.50)
Chance
(AUC = 0.44)
Chance
ROC fold 1 (AUC = 0.52)
ROC fold 2 (AUC = 0.93)
ROC fold 3 (AUC = 1.00)
ROC fold 4 (AUC = 1.00)
ROC fold 5 (AUC = 1.00)
ROC fold 6 (AUC = 1.00)
ROC fold 7 (AUC = 0.96)
ROC fold 8 (AUC = 1.00)
ROC fold 9 (AUC = 1.00)
ROC fold 10 (AUC = 1.00)
Chance
Mean ROC (AUC = 0.94 ± 0.14)
± 1 std. dev.
ROC fold 1 (AUC = 0.41)
ROC fold 2 (AUC = 0.97)
ROC fold 3 (AUC = 1.00)
ROC fold 4 (AUC = 1.00)
ROC fold 5 (AUC = 1.00)
ROC fold 6 (AUC = 1.00)
ROC fold 7 (AUC = 1.00)
ROC fold 8 (AUC = 1.00)
ROC fold 9 (AUC = 0.97)
ROC fold 10 (AUC = 1.00)
Chance
Mean ROC (AUC = 0.93 ± 0.18)
± 1 std. dev.
ROC fold 1 (AUC = 0.49)
ROC fold 2 (AUC = 0.95)
ROC fold 3 (AUC = 1.00)
ROC fold 4 (AUC = 1.00)
ROC fold 5 (AUC = 1.00)
ROC fold 6 (AUC = 1.00)
ROC fold 7 (AUC = 1.00)
ROC fold 8 (AUC = 1.00)
ROC fold 9 (AUC = 1.00)
ROC fold 10 (AUC = 1.00)
Chance
Mean ROC (AUC = 0.94 ± 0.15)
± 1 std. dev.
(AUC = 0.55)
Chance
(AUC = 0.44)
Chance
(AUC = 0.57)
Chance

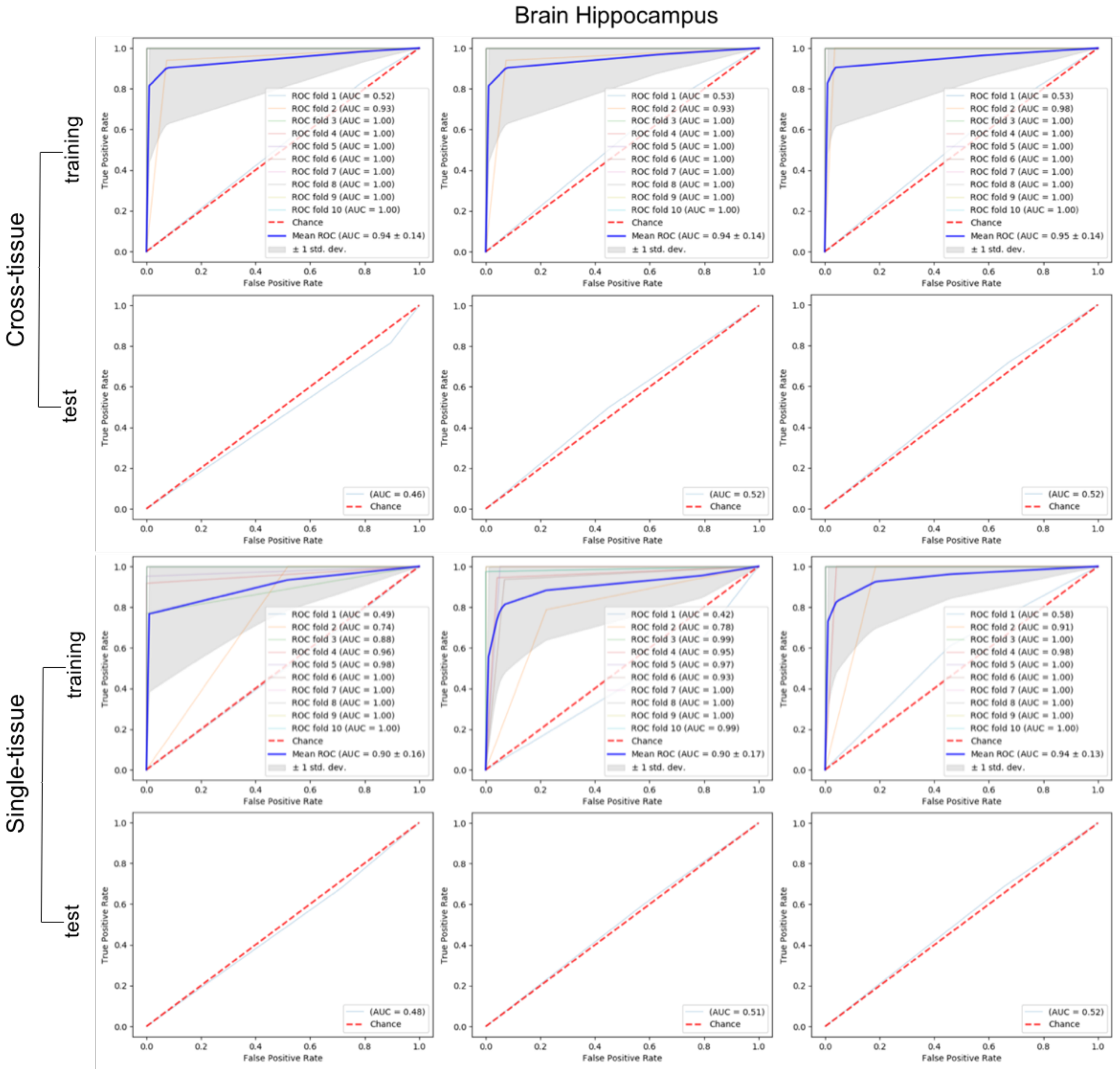
Brain Hippocampus
Cross-tissue
training
test
Single-tissue
training
test
True Positive Rate
False Positive Rate
ROC fold 1 (AUC = 0.52)
ROC fold 2 (AUC = 0.93)
Chance
Mean ROC (AUC = 0.94 ± 0.14)
± 1 std. dev.
Mean ROC (AUC = 0.95 ± 0.14)
(AUC = 0.46)
(AUC = 0.52)
Mean ROC (AUC = 0.90 ± 0.16)
Mean ROC (AUC = 0.90 ± 0.17)
Mean ROC (AUC = 0.94 ± 0.13)
(AUC = 0.48)
(AUC = 0.51)

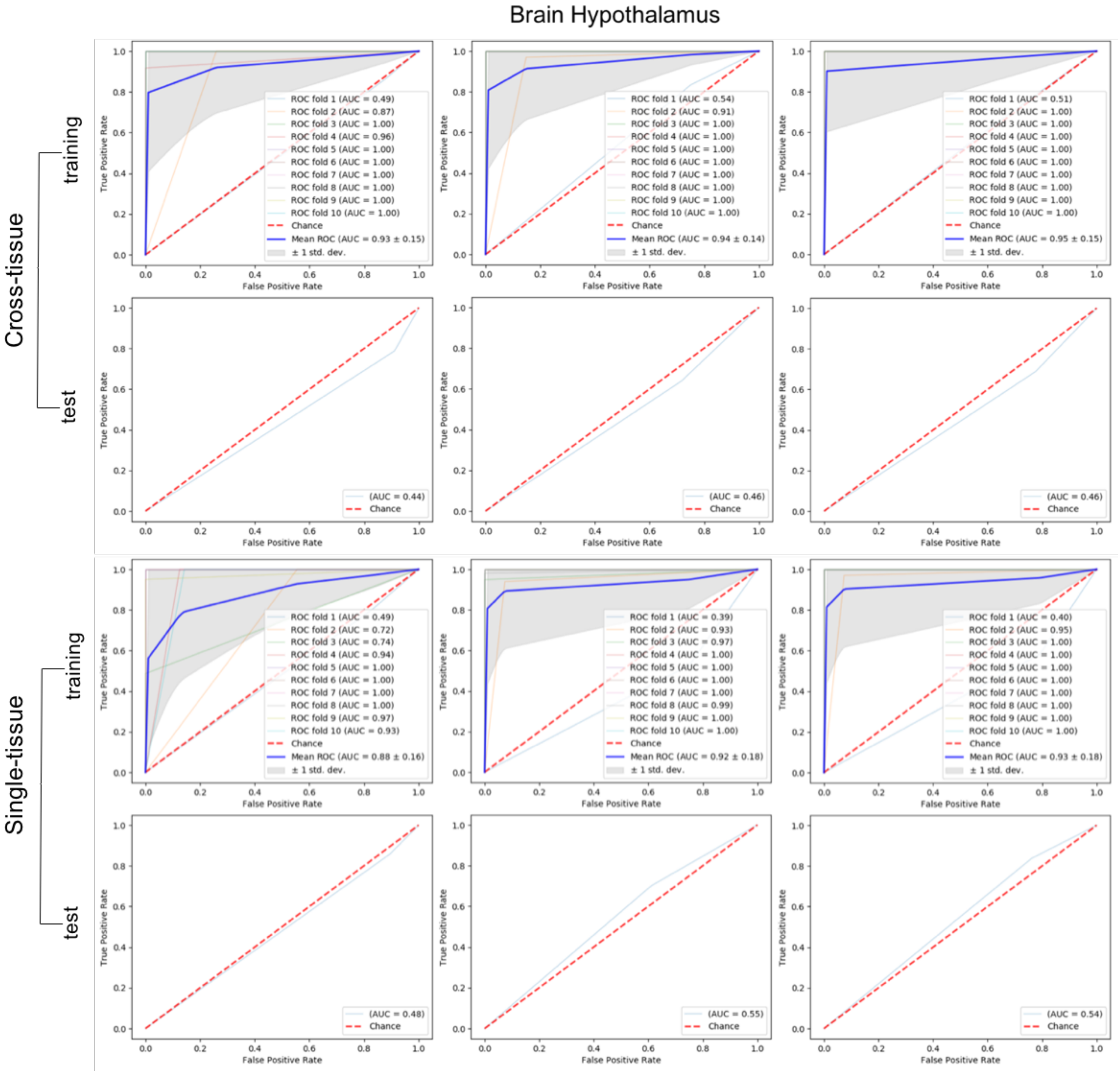
Brain Hypothalamus
Cross-tissue
training
test
Single-tissue
training
test
True Positive Rate
False Positive Rate
ROC fold 1 (AUC = 0.49)
ROC fold 2 (AUC = 0.87)
ROC fold 3 (AUC = 1.00)
ROC fold 4 (AUC = 0.96)
ROC fold 5 (AUC = 1.00)
ROC fold 6 (AUC = 1.00)
ROC fold 7 (AUC = 1.00)
ROC fold 8 (AUC = 1.00)
ROC fold 9 (AUC = 1.00)
ROC fold 10 (AUC = 1.00)
Chance
Mean ROC (AUC = 0.93 ± 0.15)
± 1 std. dev.
ROC fold 1 (AUC = 0.54)
ROC fold 2 (AUC = 0.91)
ROC fold 3 (AUC = 1.00)
ROC fold 4 (AUC = 1.00)
ROC fold 5 (AUC = 1.00)
ROC fold 6 (AUC = 1.00)
ROC fold 7 (AUC = 1.00)
ROC fold 8 (AUC = 1.00)
ROC fold 9 (AUC = 1.00)
ROC fold 10 (AUC = 1.00)
Chance
Mean ROC (AUC = 0.94 ± 0.14)
± 1 std. dev.
ROC fold 1 (AUC = 0.51)
ROC fold 2 (AUC = 1.00)
ROC fold 3 (AUC = 1.00)
ROC fold 4 (AUC = 1.00)
ROC fold 5 (AUC = 1.00)
ROC fold 6 (AUC = 1.00)
ROC fold 7 (AUC = 1.00)
ROC fold 8 (AUC = 1.00)
ROC fold 9 (AUC = 1.00)
ROC fold 10 (AUC = 1.00)
Chance
Mean ROC (AUC = 0.95 ± 0.15)
± 1 std. dev.
(AUC = 0.44)
Chance
(AUC = 0.46)
Chance
(AUC = 0.46)
Chance
ROC fold 1 (AUC = 0.49)
ROC fold 2 (AUC = 0.72)
ROC fold 3 (AUC = 0.74)
ROC fold 4 (AUC = 0.94)
ROC fold 5 (AUC = 1.00)
ROC fold 6 (AUC = 1.00)
ROC fold 7 (AUC = 1.00)
ROC fold 8 (AUC = 1.00)
ROC fold 9 (AUC = 0.97)
ROC fold 10 (AUC = 0.93)
Chance
Mean ROC (AUC = 0.88 ± 0.16)
± 1 std. dev.
ROC fold 1 (AUC = 0.39)
ROC fold 2 (AUC = 0.93)
ROC fold 3 (AUC = 0.97)
ROC fold 4 (AUC = 1.00)
ROC fold 5 (AUC = 1.00)
ROC fold 6 (AUC = 1.00)
ROC fold 7 (AUC = 1.00)
ROC fold 8 (AUC = 0.99)
ROC fold 9 (AUC = 1.00)
ROC fold 10 (AUC = 1.00)
Chance
Mean ROC (AUC = 0.92 ± 0.18)
± 1 std. dev.
ROC fold 1 (AUC = 0.40)
ROC fold 2 (AUC = 0.95)
ROC fold 3 (AUC = 1.00)
ROC fold 4 (AUC = 1.00)
ROC fold 5 (AUC = 1.00)
ROC fold 6 (AUC = 1.00)
ROC fold 7 (AUC = 1.00)
ROC fold 8 (AUC = 1.00)
ROC fold 9 (AUC = 1.00)
ROC fold 10 (AUC = 1.00)
Chance
Mean ROC (AUC = 0.93 ± 0.18)
± 1 std. dev.
(AUC = 0.48)
Chance
(AUC = 0.55)
Chance
(AUC = 0.54)
Chance

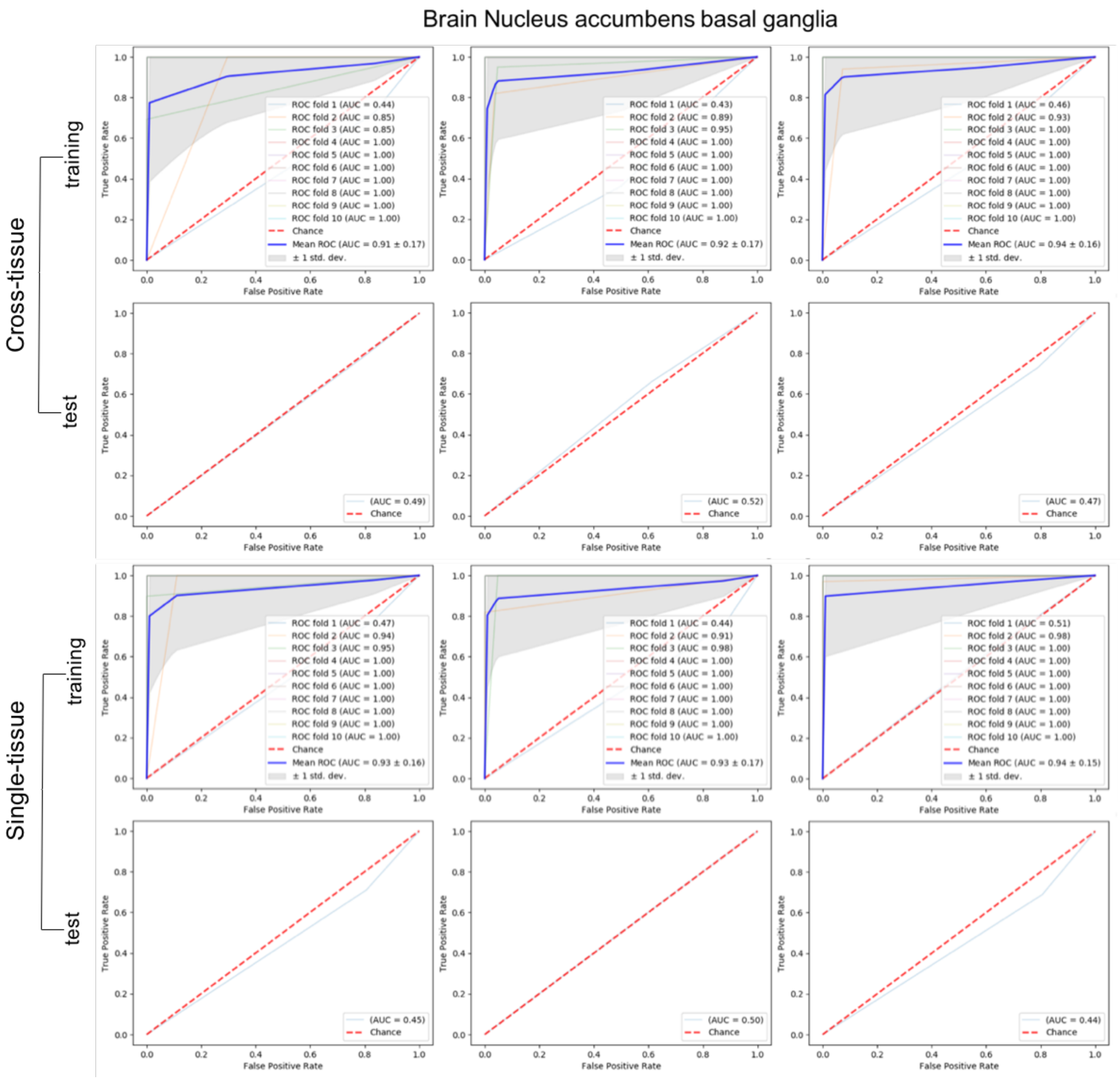
Brain Nucleus accumbens basal ganglia
Cross-tissue
training
test
Single-tissue
training
test
True Positive Rate
False Positive Rate
ROC fold 1 (AUC = 0.44)
ROC fold 2 (AUC = 0.85)
ROC fold 3 (AUC = 0.85)
ROC fold 4 (AUC = 1.00)
ROC fold 5 (AUC = 1.00)
ROC fold 6 (AUC = 1.00)
ROC fold 7 (AUC = 1.00)
ROC fold 8 (AUC = 1.00)
ROC fold 9 (AUC = 1.00)
ROC fold 10 (AUC = 1.00)
Chance
Mean ROC (AUC = 0.91 ± 0.17)
± 1 std. dev.
ROC fold 1 (AUC = 0.43)
ROC fold 2 (AUC = 0.89)
ROC fold 3 (AUC = 0.95)
ROC fold 4 (AUC = 1.00)
ROC fold 5 (AUC = 1.00)
ROC fold 6 (AUC = 1.00)
ROC fold 7 (AUC = 1.00)
ROC fold 8 (AUC = 1.00)
ROC fold 9 (AUC = 1.00)
ROC fold 10 (AUC = 1.00)
Chance
Mean ROC (AUC = 0.92 ± 0.17)
± 1 std. dev.
ROC fold 1 (AUC = 0.46)
ROC fold 2 (AUC = 0.93)
ROC fold 3 (AUC = 1.00)
ROC fold 4 (AUC = 1.00)
ROC fold 5 (AUC = 1.00)
ROC fold 6 (AUC = 1.00)
ROC fold 7 (AUC = 1.00)
ROC fold 8 (AUC = 1.00)
ROC fold 9 (AUC = 1.00)
ROC fold 10 (AUC = 1.00)
Chance
Mean ROC (AUC = 0.94 ± 0.16)
± 1 std. dev.
(AUC = 0.49)
Chance
(AUC = 0.52)
Chance
(AUC = 0.47)
Chance
ROC fold 1 (AUC = 0.47)
ROC fold 2 (AUC = 0.94)
ROC fold 3 (AUC = 0.95)
ROC fold 4 (AUC = 1.00)
ROC fold 5 (AUC = 1.00)
ROC fold 6 (AUC = 1.00)
ROC fold 7 (AUC = 1.00)
ROC fold 8 (AUC = 1.00)
ROC fold 9 (AUC = 1.00)
ROC fold 10 (AUC = 1.00)
Chance
Mean ROC (AUC = 0.93 ± 0.16)
± 1 std. dev.
ROC fold 1 (AUC = 0.44)
ROC fold 2 (AUC = 0.91)
ROC fold 3 (AUC = 0.98)
ROC fold 4 (AUC = 1.00)
ROC fold 5 (AUC = 1.00)
ROC fold 6 (AUC = 1.00)
ROC fold 7 (AUC = 1.00)
ROC fold 8 (AUC = 1.00)
ROC fold 9 (AUC = 1.00)
ROC fold 10 (AUC = 1.00)
Chance
Mean ROC (AUC = 0.93 ± 0.17)
± 1 std. dev.
ROC fold 1 (AUC = 0.51)
ROC fold 2 (AUC = 0.98)
ROC fold 3 (AUC = 1.00)
ROC fold 4 (AUC = 1.00)
ROC fold 5 (AUC = 1.00)
ROC fold 6 (AUC = 1.00)
ROC fold 7 (AUC = 1.00)
ROC fold 8 (AUC = 1.00)
ROC fold 9 (AUC = 1.00)
ROC fold 10 (AUC = 1.00)
Chance
Mean ROC (AUC = 0.94 ± 0.15)
± 1 std. dev.
(AUC = 0.45)
Chance
(AUC = 0.50)
Chance
(AUC = 0.44)
Chance

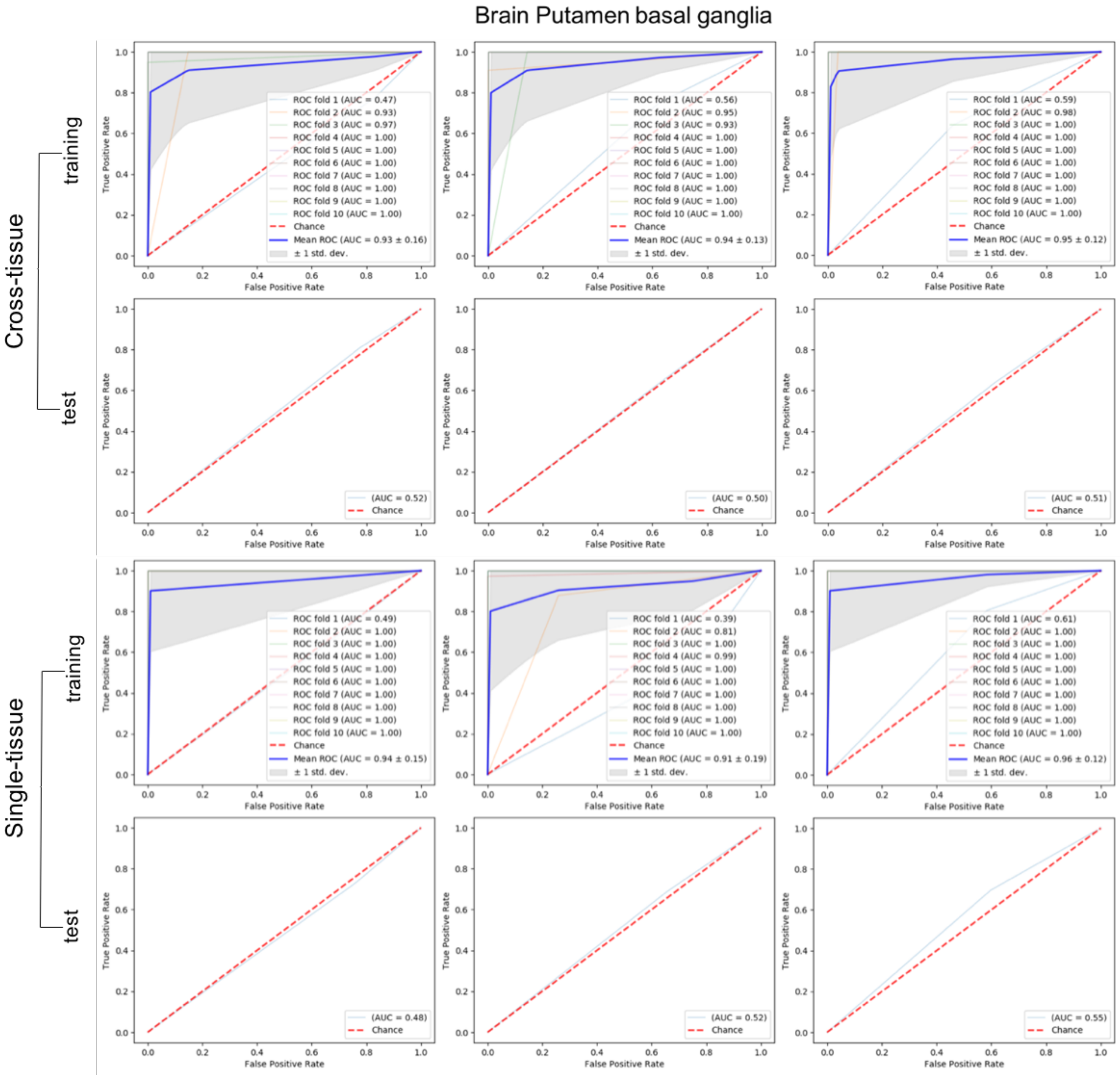
Brain Putamen basal ganglia
Cross-tissue
training
test
Single-tissue
training
test
True Positive Rate
False Positive Rate
ROC fold 1 (AUC = 0.47)
ROC fold 2 (AUC = 0.93)
ROC fold 3 (AUC = 0.97)
ROC fold 4 (AUC = 1.00)
ROC fold 5 (AUC = 1.00)
ROC fold 6 (AUC = 1.00)
ROC fold 7 (AUC = 1.00)
ROC fold 8 (AUC = 1.00)
ROC fold 9 (AUC = 1.00)
ROC fold 10 (AUC = 1.00)
Chance
Mean ROC (AUC = 0.93 ± 0.16)
± 1 std. dev.
ROC fold 1 (AUC = 0.56)
ROC fold 2 (AUC = 0.95)
ROC fold 3 (AUC = 0.93)
Mean ROC (AUC = 0.94 ± 0.13)
ROC fold 1 (AUC = 0.59)
ROC fold 2 (AUC = 0.98)
Mean ROC (AUC = 0.95 ± 0.12)
(AUC = 0.52)
(AUC = 0.50)
(AUC = 0.51)
ROC fold 1 (AUC = 0.49)
Mean ROC (AUC = 0.94 ± 0.15)
ROC fold 1 (AUC = 0.39)
ROC fold 2 (AUC = 0.81)
ROC fold 4 (AUC = 0.99)
Mean ROC (AUC = 0.91 ± 0.19)
ROC fold 1 (AUC = 0.61)
Mean ROC (AUC = 0.96 ± 0.12)
(AUC = 0.48)
(AUC = 0.52)
(AUC = 0.55)

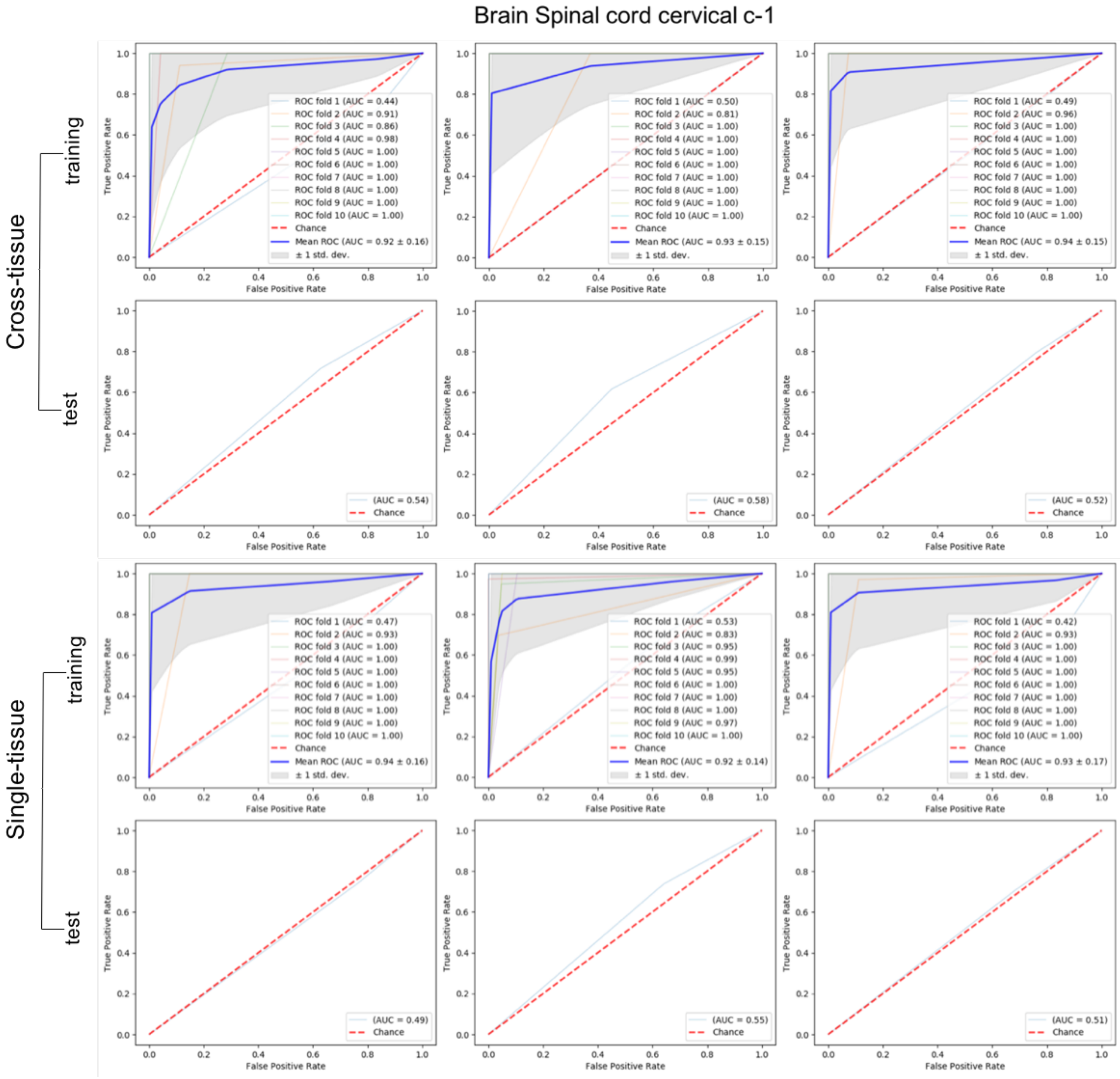
Brain Spinal cord cervical c-1
Cross-tissue
training
test
Single-tissue
training
test
True Positive Rate
False Positive Rate
ROC fold 1 (AUC = 0.44)
ROC fold 2 (AUC = 0.91)
ROC fold 3 (AUC = 0.86)
ROC fold 4 (AUC = 0.98)
ROC fold 5 (AUC = 1.00)
ROC fold 6 (AUC = 1.00)
ROC fold 7 (AUC = 1.00)
ROC fold 8 (AUC = 1.00)
ROC fold 9 (AUC = 1.00)
ROC fold 10 (AUC = 1.00)
Chance
Mean ROC (AUC = 0.92 ± 0.16)
± 1 std. dev.
ROC fold 1 (AUC = 0.50)
ROC fold 2 (AUC = 0.81)
ROC fold 3 (AUC = 1.00)
ROC fold 4 (AUC = 1.00)
ROC fold 5 (AUC = 1.00)
ROC fold 6 (AUC = 1.00)
ROC fold 7 (AUC = 1.00)
ROC fold 8 (AUC = 1.00)
ROC fold 9 (AUC = 1.00)
ROC fold 10 (AUC = 1.00)
Chance
Mean ROC (AUC = 0.93 ± 0.15)
± 1 std. dev.
ROC fold 1 (AUC = 0.49)
ROC fold 2 (AUC = 0.96)
ROC fold 3 (AUC = 1.00)
ROC fold 4 (AUC = 1.00)
ROC fold 5 (AUC = 1.00)
ROC fold 6 (AUC = 1.00)
ROC fold 7 (AUC = 1.00)
ROC fold 8 (AUC = 1.00)
ROC fold 9 (AUC = 1.00)
ROC fold 10 (AUC = 1.00)
Chance
Mean ROC (AUC = 0.94 ± 0.15)
± 1 std. dev.
(AUC = 0.54)
Chance
(AUC = 0.58)
Chance
(AUC = 0.52)
Chance
ROC fold 1 (AUC = 0.47)
ROC fold 2 (AUC = 0.93)
ROC fold 3 (AUC = 1.00)
ROC fold 4 (AUC = 1.00)
ROC fold 5 (AUC = 1.00)
ROC fold 6 (AUC = 1.00)
ROC fold 7 (AUC = 1.00)
ROC fold 8 (AUC = 1.00)
ROC fold 9 (AUC = 1.00)
ROC fold 10 (AUC = 1.00)
Chance
Mean ROC (AUC = 0.94 ± 0.16)
± 1 std. dev.
ROC fold 1 (AUC = 0.53)
ROC fold 2 (AUC = 0.83)
ROC fold 3 (AUC = 0.95)
ROC fold 4 (AUC = 0.99)
ROC fold 5 (AUC = 0.95)
ROC fold 6 (AUC = 1.00)
ROC fold 7 (AUC = 1.00)
ROC fold 8 (AUC = 1.00)
ROC fold 9 (AUC = 0.97)
ROC fold 10 (AUC = 1.00)
Chance
Mean ROC (AUC = 0.92 ± 0.14)
± 1 std. dev.
ROC fold 1 (AUC = 0.42)
ROC fold 2 (AUC = 0.93)
ROC fold 3 (AUC = 1.00)
ROC fold 4 (AUC = 1.00)
ROC fold 5 (AUC = 1.00)
ROC fold 6 (AUC = 1.00)
ROC fold 7 (AUC = 1.00)
ROC fold 8 (AUC = 1.00)
ROC fold 9 (AUC = 1.00)
ROC fold 10 (AUC = 1.00)
Chance
Mean ROC (AUC = 0.93 ± 0.17)
± 1 std. dev.
(AUC = 0.49)
Chance
(AUC = 0.55)
Chance
(AUC = 0.51)
Chance

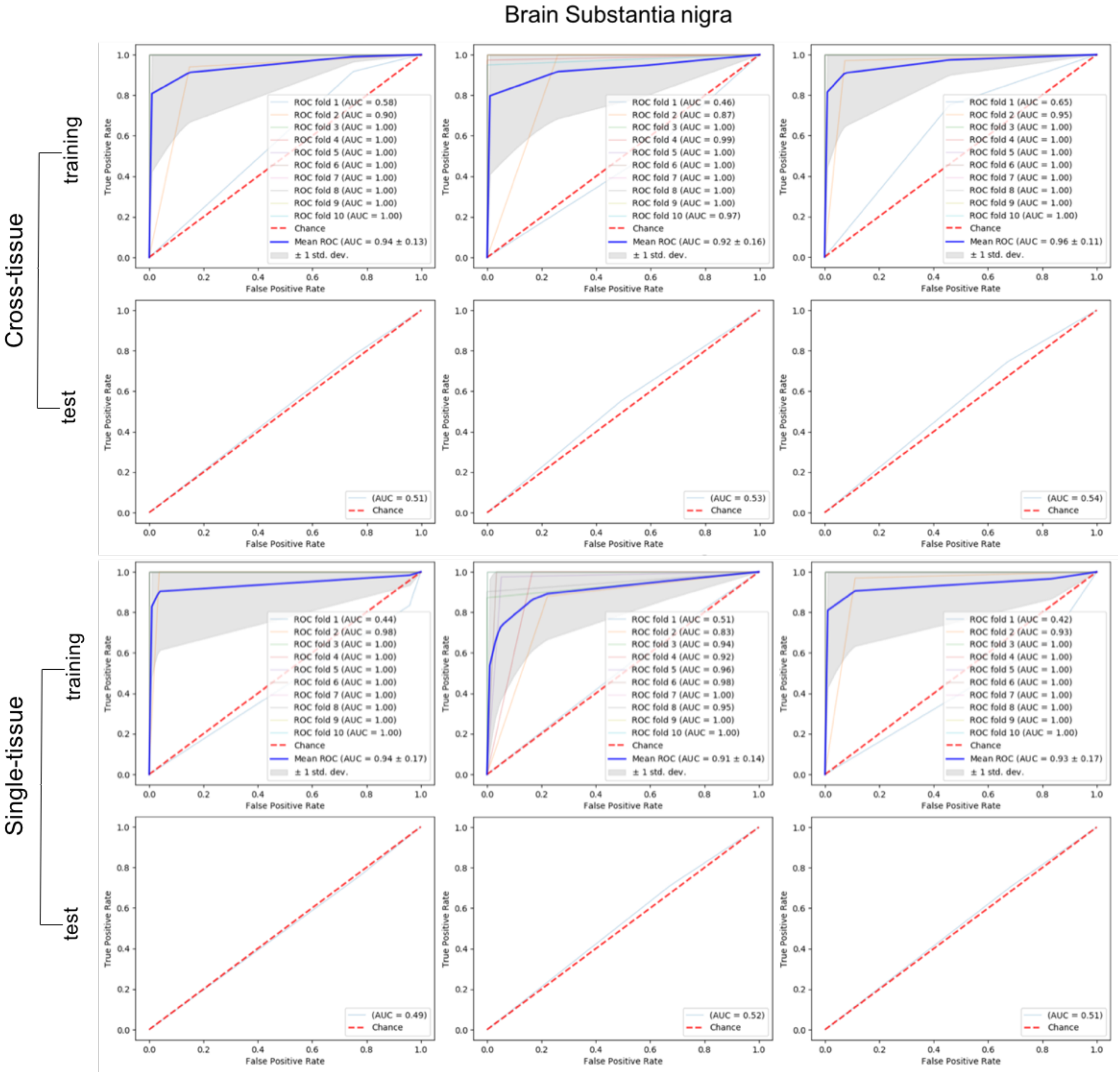

2.6 ROC Curve: Cognitive Decline

Here are reported the ROC curve obtained for each Cognitive Decline Brain tissue model during the training and test, for cross-tissue (first and second row) and single-tissue analysis (third and fourth row). By column are reported the models without feature selection (left column), with List-unsupervised (middle column) and with List-PrediXcan selection (right column).

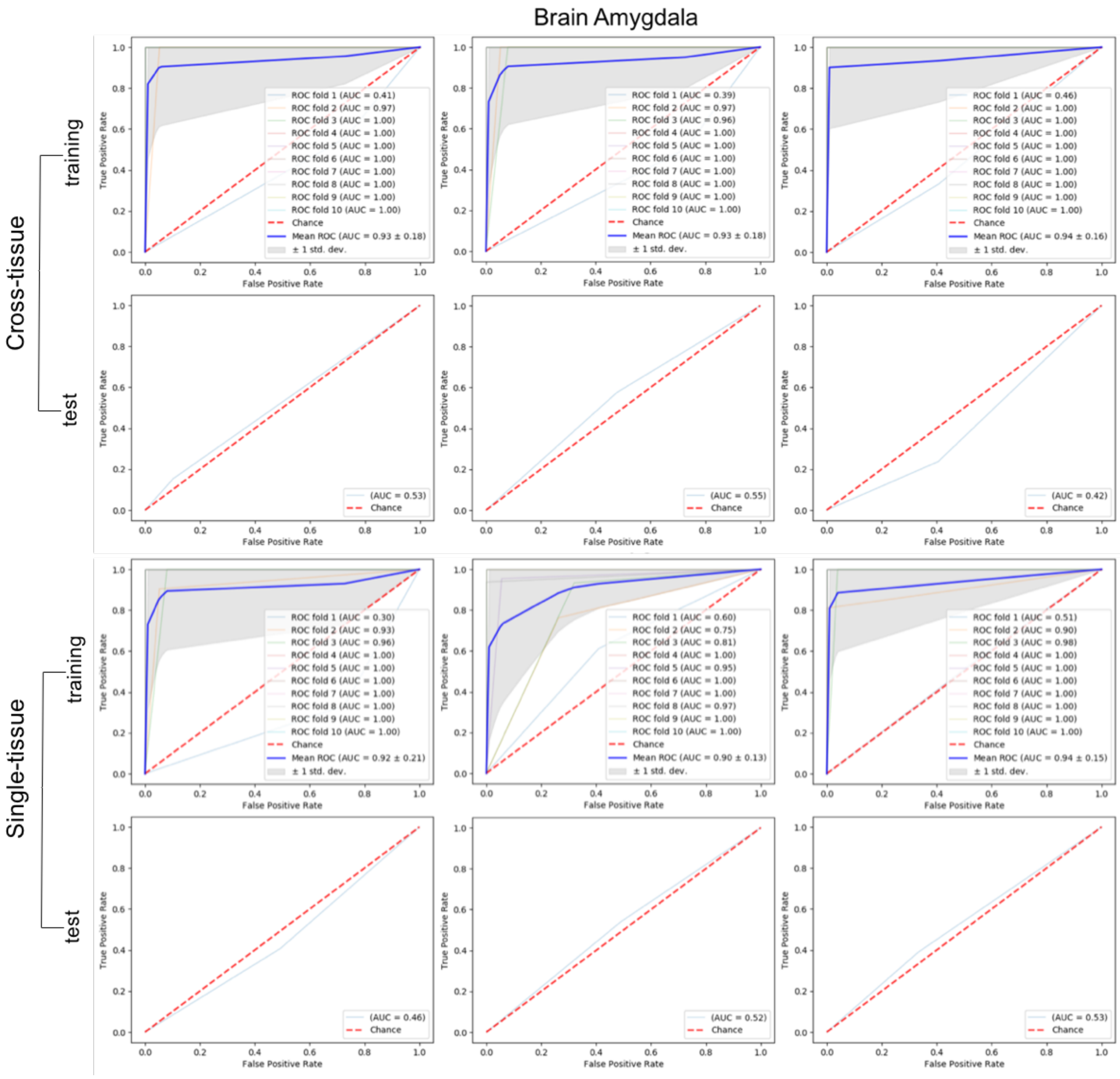
Brain Amygdala
Cross-tissue
training
test
Single-tissue
training
test
True Positive Rate
False Positive Rate
ROC fold 1 (AUC = 0.41)
ROC fold 2 (AUC = 0.97)
ROC fold 3 (AUC = 1.00)
ROC fold 4 (AUC = 1.00)
ROC fold 5 (AUC = 1.00)
ROC fold 6 (AUC = 1.00)
ROC fold 7 (AUC = 1.00)
ROC fold 8 (AUC = 1.00)
ROC fold 9 (AUC = 1.00)
ROC fold 10 (AUC = 1.00)
Chance
Mean ROC (AUC = 0.93 ± 0.18)
± 1 std. dev.
ROC fold 1 (AUC = 0.39)
ROC fold 2 (AUC = 0.97)
ROC fold 3 (AUC = 0.96)
ROC fold 4 (AUC = 1.00)
ROC fold 5 (AUC = 1.00)
ROC fold 6 (AUC = 1.00)
ROC fold 7 (AUC = 1.00)
ROC fold 8 (AUC = 1.00)
ROC fold 9 (AUC = 1.00)
ROC fold 10 (AUC = 1.00)
Chance
Mean ROC (AUC = 0.93 ± 0.18)
± 1 std. dev.
ROC fold 1 (AUC = 0.46)
ROC fold 2 (AUC = 1.00)
ROC fold 3 (AUC = 1.00)
ROC fold 4 (AUC = 1.00)
ROC fold 5 (AUC = 1.00)
ROC fold 6 (AUC = 1.00)
ROC fold 7 (AUC = 1.00)
ROC fold 8 (AUC = 1.00)
ROC fold 9 (AUC = 1.00)
ROC fold 10 (AUC = 1.00)
Chance
Mean ROC (AUC = 0.94 ± 0.16)
± 1 std. dev.
(AUC = 0.53)
Chance
(AUC = 0.55)
Chance
(AUC = 0.42)
Chance
ROC fold 1 (AUC = 0.30)
ROC fold 2 (AUC = 0.93)
ROC fold 3 (AUC = 0.96)
ROC fold 4 (AUC = 1.00)
ROC fold 5 (AUC = 1.00)
ROC fold 6 (AUC = 1.00)
ROC fold 7 (AUC = 1.00)
ROC fold 8 (AUC = 1.00)
ROC fold 9 (AUC = 1.00)
ROC fold 10 (AUC = 1.00)
Chance
Mean ROC (AUC = 0.92 ± 0.21)
± 1 std. dev.
ROC fold 1 (AUC = 0.60)
ROC fold 2 (AUC = 0.75)
ROC fold 3 (AUC = 0.81)
ROC fold 4 (AUC = 1.00)
ROC fold 5 (AUC = 0.95)
ROC fold 6 (AUC = 1.00)
ROC fold 7 (AUC = 1.00)
ROC fold 8 (AUC = 0.97)
ROC fold 9 (AUC = 1.00)
ROC fold 10 (AUC = 1.00)
Chance
Mean ROC (AUC = 0.90 ± 0.13)
± 1 std. dev.
ROC fold 1 (AUC = 0.51)
ROC fold 2 (AUC = 0.90)
ROC fold 3 (AUC = 0.98)
ROC fold 4 (AUC = 1.00)
ROC fold 5 (AUC = 1.00)
ROC fold 6 (AUC = 1.00)
ROC fold 7 (AUC = 1.00)
ROC fold 8 (AUC = 1.00)
ROC fold 9 (AUC = 1.00)
ROC fold 10 (AUC = 1.00)
Chance
Mean ROC (AUC = 0.94 ± 0.15)
± 1 std. dev.
(AUC = 0.46)
Chance
(AUC = 0.52)
Chance
(AUC = 0.53)
Chance

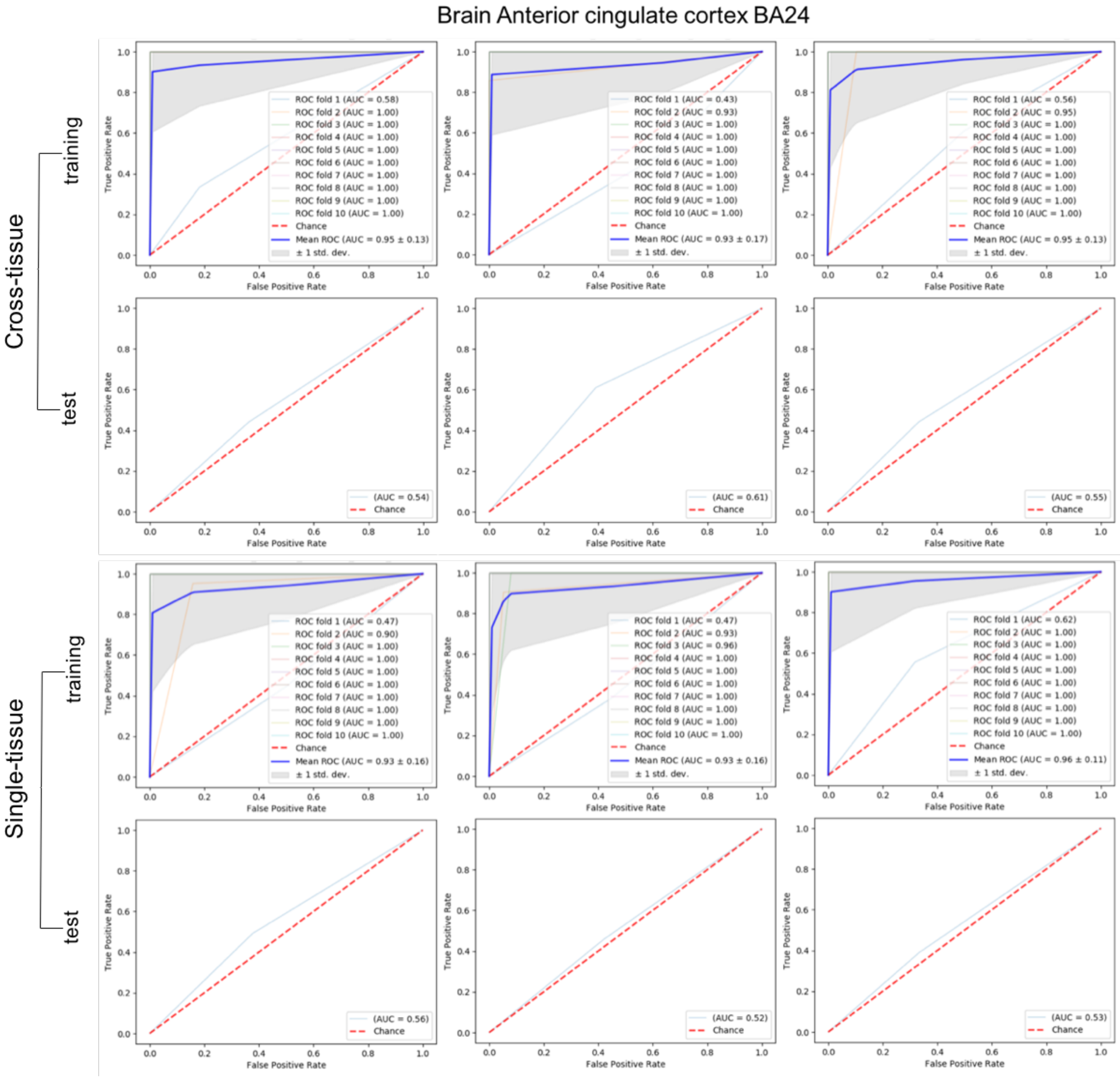
Brain Anterior cingulate cortex BA24
Cross-tissue
Single-tissue
training
test
True Positive Rate
False Positive Rate
ROC fold 1 (AUC = 0.58)
ROC fold 2 (AUC = 1.00)
ROC fold 3 (AUC = 1.00)
ROC fold 4 (AUC = 1.00)
ROC fold 5 (AUC = 1.00)
ROC fold 6 (AUC = 1.00)
ROC fold 7 (AUC = 1.00)
ROC fold 8 (AUC = 1.00)
ROC fold 9 (AUC = 1.00)
ROC fold 10 (AUC = 1.00)
Chance
Mean ROC (AUC = 0.95 ± 0.13)
± 1 std. dev.
ROC fold 1 (AUC = 0.43)
ROC fold 2 (AUC = 0.93)
ROC fold 3 (AUC = 1.00)
ROC fold 4 (AUC = 1.00)
ROC fold 5 (AUC = 1.00)
ROC fold 6 (AUC = 1.00)
ROC fold 7 (AUC = 1.00)
ROC fold 8 (AUC = 1.00)
ROC fold 9 (AUC = 1.00)
ROC fold 10 (AUC = 1.00)
Chance
Mean ROC (AUC = 0.93 ± 0.17)
± 1 std. dev.
ROC fold 1 (AUC = 0.56)
ROC fold 2 (AUC = 0.95)
ROC fold 3 (AUC = 1.00)
ROC fold 4 (AUC = 1.00)
ROC fold 5 (AUC = 1.00)
ROC fold 6 (AUC = 1.00)
ROC fold 7 (AUC = 1.00)
ROC fold 8 (AUC = 1.00)
ROC fold 9 (AUC = 1.00)
ROC fold 10 (AUC = 1.00)
Chance
Mean ROC (AUC = 0.95 ± 0.13)
± 1 std. dev.
(AUC = 0.54)
Chance
(AUC = 0.61)
Chance
(AUC = 0.55)
Chance
ROC fold 1 (AUC = 0.47)
ROC fold 2 (AUC = 0.90)
ROC fold 3 (AUC = 1.00)
ROC fold 4 (AUC = 1.00)
ROC fold 5 (AUC = 1.00)
ROC fold 6 (AUC = 1.00)
ROC fold 7 (AUC = 1.00)
ROC fold 8 (AUC = 1.00)
ROC fold 9 (AUC = 1.00)
ROC fold 10 (AUC = 1.00)
Chance
Mean ROC (AUC = 0.93 ± 0.16)
± 1 std. dev.
ROC fold 1 (AUC = 0.47)
ROC fold 2 (AUC = 0.93)
ROC fold 3 (AUC = 0.96)
ROC fold 4 (AUC = 1.00)
ROC fold 5 (AUC = 1.00)
ROC fold 6 (AUC = 1.00)
ROC fold 7 (AUC = 1.00)
ROC fold 8 (AUC = 1.00)
ROC fold 9 (AUC = 1.00)
ROC fold 10 (AUC = 1.00)
Chance
Mean ROC (AUC = 0.93 ± 0.16)
± 1 std. dev.
ROC fold 1 (AUC = 0.62)
ROC fold 2 (AUC = 1.00)
ROC fold 3 (AUC = 1.00)
ROC fold 4 (AUC = 1.00)
ROC fold 5 (AUC = 1.00)
ROC fold 6 (AUC = 1.00)
ROC fold 7 (AUC = 1.00)
ROC fold 8 (AUC = 1.00)
ROC fold 9 (AUC = 1.00)
ROC fold 10 (AUC = 1.00)
Chance
Mean ROC (AUC = 0.96 ± 0.11)
± 1 std. dev.
(AUC = 0.56)
Chance
(AUC = 0.52)
Chance
(AUC = 0.53)
Chance

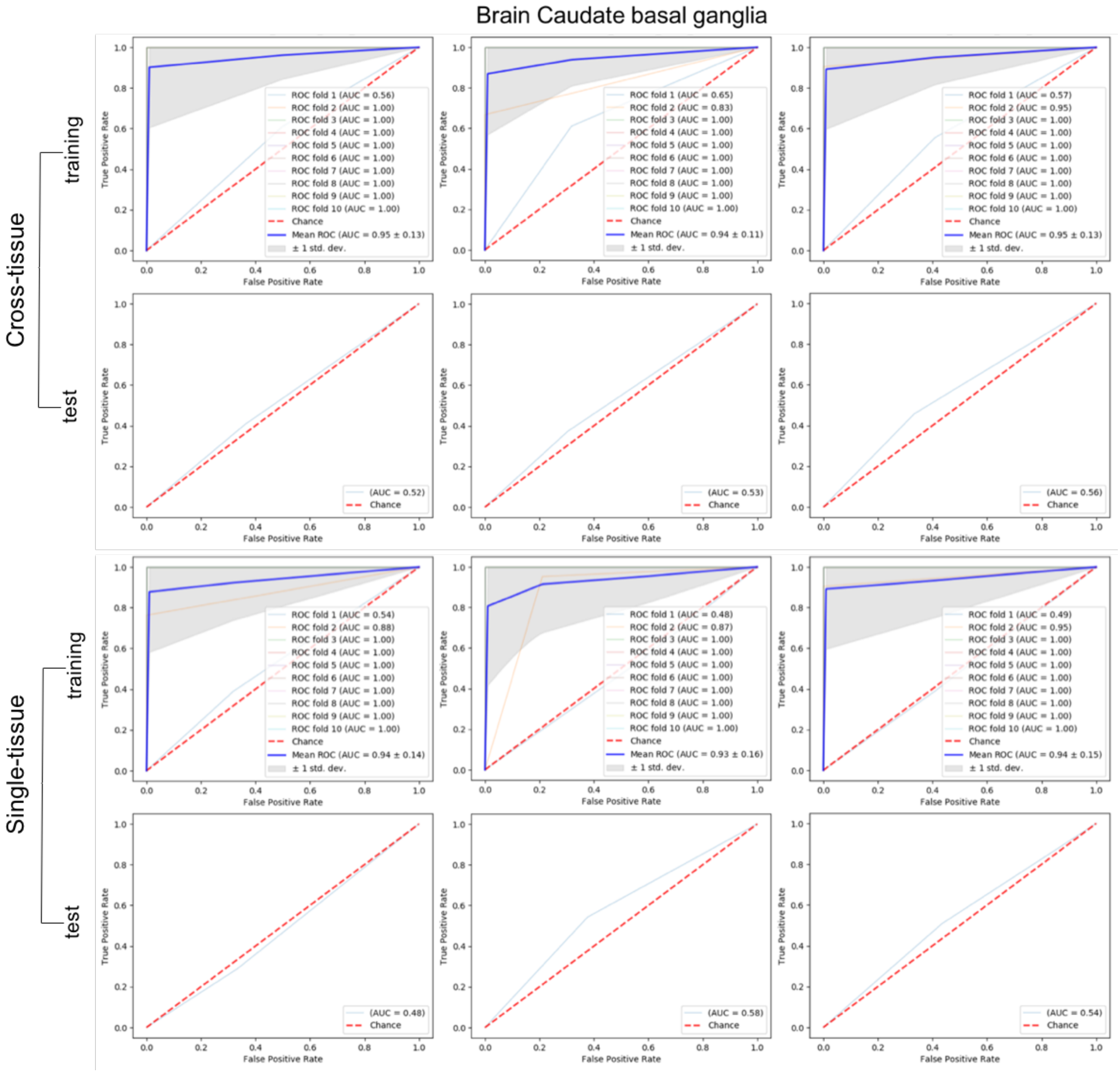
Brain Caudate basal ganglia
Cross-tissue
Single-tissue
training
test
True Positive Rate
False Positive Rate
ROC fold 1 (AUC = 0.56)
Chance
Mean ROC (AUC = 0.95 ± 0.13)
± 1 std. dev.
Mean ROC (AUC = 0.94 ± 0.11)
Mean ROC (AUC = 0.95 ± 0.13)
(AUC = 0.52)
(AUC = 0.53)
(AUC = 0.56)
Mean ROC (AUC = 0.94 ± 0.14)
Mean ROC (AUC = 0.93 ± 0.16)
Mean ROC (AUC = 0.94 ± 0.15)
(AUC = 0.48)
(AUC = 0.58)
(AUC = 0.54)

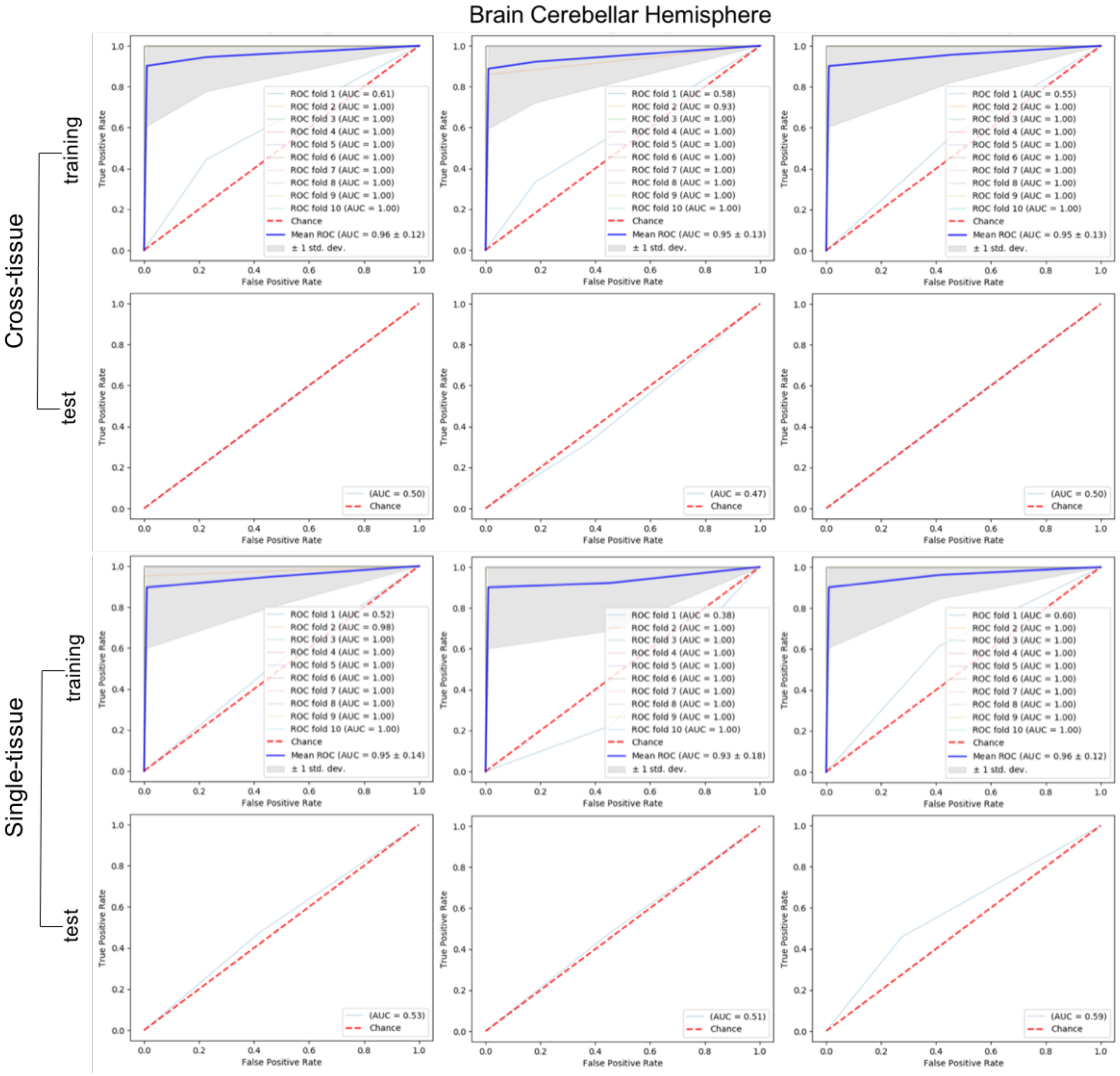
Brain Cerebellar Hemisphere
Cross-tissue
training
test
Single-tissue
training
test
True Positive Rate
False Positive Rate
ROC fold 1 (AUC = 0.61)
Mean ROC (AUC = 0.96 ± 0.12)
ROC fold 1 (AUC = 0.58)
ROC fold 2 (AUC = 0.93)
Mean ROC (AUC = 0.95 ± 0.13)
ROC fold 1 (AUC = 0.55)
Mean ROC (AUC = 0.95 ± 0.13)
(AUC = 0.50)
(AUC = 0.47)
(AUC = 0.50)
ROC fold 1 (AUC = 0.52)
ROC fold 2 (AUC = 0.98)
Mean ROC (AUC = 0.95 ± 0.14)
ROC fold 1 (AUC = 0.38)
Mean ROC (AUC = 0.93 ± 0.18)
ROC fold 1 (AUC = 0.60)
Mean ROC (AUC = 0.96 ± 0.12)
(AUC = 0.53)
(AUC = 0.51)
(AUC = 0.59)
Chance
± 1 std. dev.

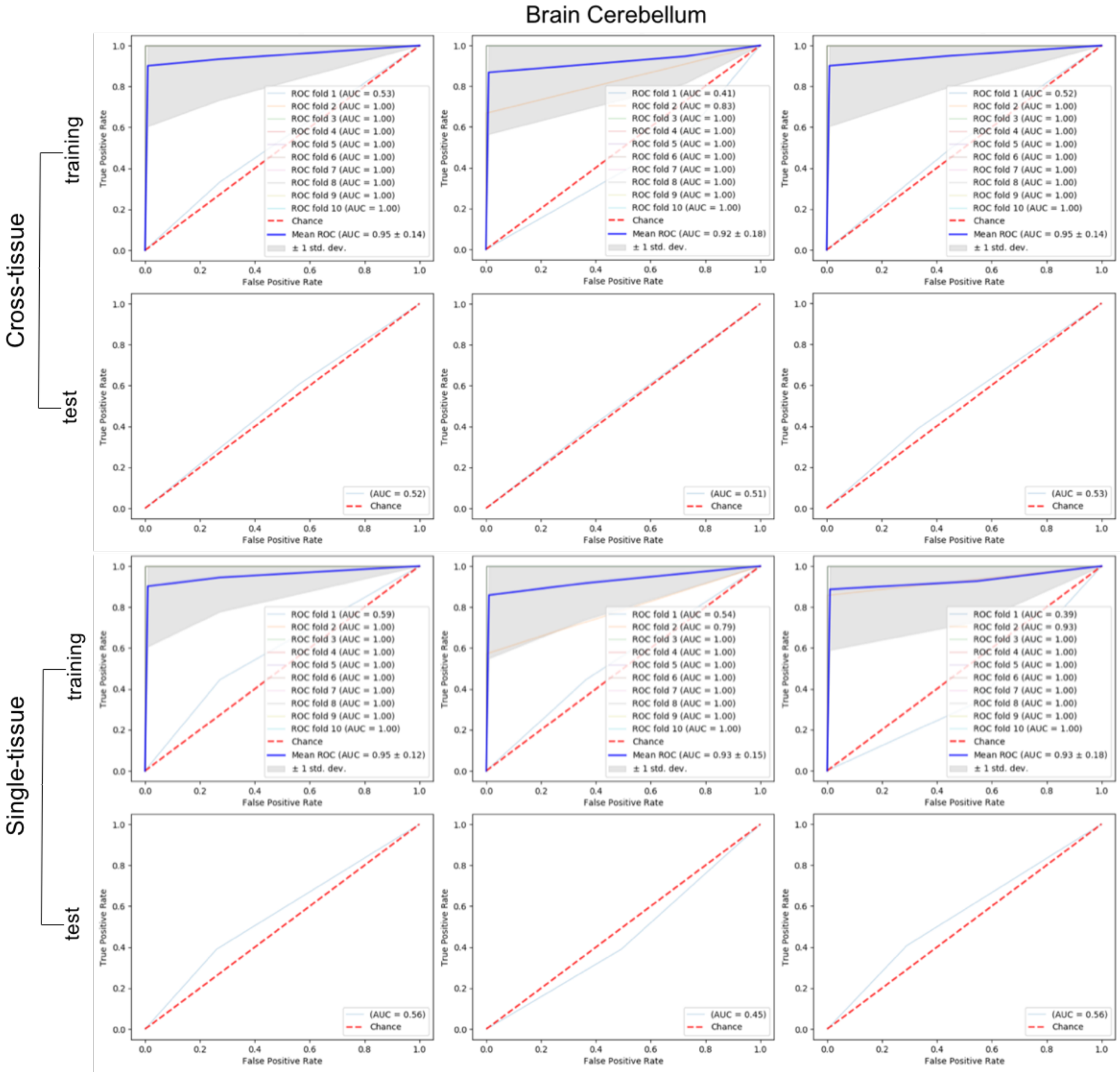
Brain Cerebellum
Cross-tissue
training
test
Single-tissue
training
test
True Positive Rate
False Positive Rate
ROC fold 1 (AUC = 0.53)
ROC fold 2 (AUC = 1.00)
ROC fold 3 (AUC = 1.00)
ROC fold 4 (AUC = 1.00)
ROC fold 5 (AUC = 1.00)
ROC fold 6 (AUC = 1.00)
ROC fold 7 (AUC = 1.00)
ROC fold 8 (AUC = 1.00)
ROC fold 9 (AUC = 1.00)
ROC fold 10 (AUC = 1.00)
Chance
Mean ROC (AUC = 0.95 ± 0.14)
± 1 std. dev.
ROC fold 1 (AUC = 0.41)
ROC fold 2 (AUC = 0.83)
ROC fold 3 (AUC = 1.00)
ROC fold 4 (AUC = 1.00)
ROC fold 5 (AUC = 1.00)
ROC fold 6 (AUC = 1.00)
ROC fold 7 (AUC = 1.00)
ROC fold 8 (AUC = 1.00)
ROC fold 9 (AUC = 1.00)
ROC fold 10 (AUC = 1.00)
Chance
Mean ROC (AUC = 0.92 ± 0.18)
± 1 std. dev.
ROC fold 1 (AUC = 0.52)
ROC fold 2 (AUC = 1.00)
ROC fold 3 (AUC = 1.00)
ROC fold 4 (AUC = 1.00)
ROC fold 5 (AUC = 1.00)
ROC fold 6 (AUC = 1.00)
ROC fold 7 (AUC = 1.00)
ROC fold 8 (AUC = 1.00)
ROC fold 9 (AUC = 1.00)
ROC fold 10 (AUC = 1.00)
Chance
Mean ROC (AUC = 0.95 ± 0.14)
± 1 std. dev.
(AUC = 0.52)
Chance
(AUC = 0.51)
Chance
(AUC = 0.53)
Chance
ROC fold 1 (AUC = 0.59)
ROC fold 2 (AUC = 1.00)
ROC fold 3 (AUC = 1.00)
ROC fold 4 (AUC = 1.00)
ROC fold 5 (AUC = 1.00)
ROC fold 6 (AUC = 1.00)
ROC fold 7 (AUC = 1.00)
ROC fold 8 (AUC = 1.00)
ROC fold 9 (AUC = 1.00)
ROC fold 10 (AUC = 1.00)
Chance
Mean ROC (AUC = 0.95 ± 0.12)
± 1 std. dev.
ROC fold 1 (AUC = 0.54)
ROC fold 2 (AUC = 0.79)
ROC fold 3 (AUC = 1.00)
ROC fold 4 (AUC = 1.00)
ROC fold 5 (AUC = 1.00)
ROC fold 6 (AUC = 1.00)
ROC fold 7 (AUC = 1.00)
ROC fold 8 (AUC = 1.00)
ROC fold 9 (AUC = 1.00)
ROC fold 10 (AUC = 1.00)
Chance
Mean ROC (AUC = 0.93 ± 0.15)
± 1 std. dev.
ROC fold 1 (AUC = 0.39)
ROC fold 2 (AUC = 0.93)
ROC fold 3 (AUC = 1.00)
ROC fold 4 (AUC = 1.00)
ROC fold 5 (AUC = 1.00)
ROC fold 6 (AUC = 1.00)
ROC fold 7 (AUC = 1.00)
ROC fold 8 (AUC = 1.00)
ROC fold 9 (AUC = 1.00)
ROC fold 10 (AUC = 1.00)
Chance
Mean ROC (AUC = 0.93 ± 0.18)
± 1 std. dev.
(AUC = 0.56)
Chance
(AUC = 0.45)
Chance
(AUC = 0.56)
Chance

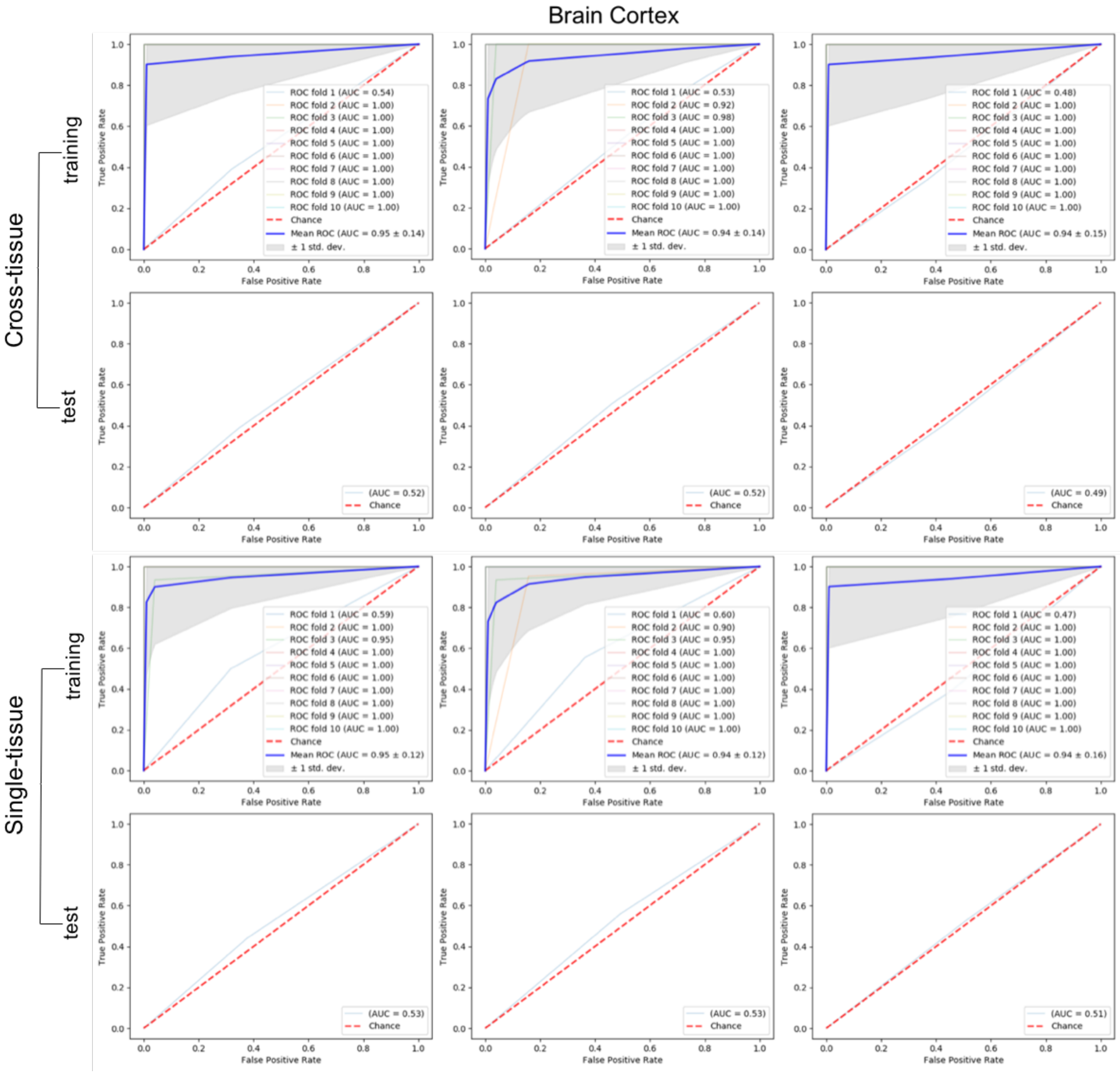
Brain Cortex
Cross-tissue
Single-tissue
training
test
True Positive Rate
False Positive Rate
ROC fold 1 (AUC = 0.54)
ROC fold 2 (AUC = 1.00)
ROC fold 3 (AUC = 1.00)
ROC fold 4 (AUC = 1.00)
ROC fold 5 (AUC = 1.00)
ROC fold 6 (AUC = 1.00)
ROC fold 7 (AUC = 1.00)
ROC fold 8 (AUC = 1.00)
ROC fold 9 (AUC = 1.00)
ROC fold 10 (AUC = 1.00)
Chance
Mean ROC (AUC = 0.95 ± 0.14)
± 1 std. dev.
ROC fold 1 (AUC = 0.53)
ROC fold 2 (AUC = 0.92)
ROC fold 3 (AUC = 0.98)
ROC fold 4 (AUC = 1.00)
ROC fold 5 (AUC = 1.00)
ROC fold 6 (AUC = 1.00)
ROC fold 7 (AUC = 1.00)
ROC fold 8 (AUC = 1.00)
ROC fold 9 (AUC = 1.00)
ROC fold 10 (AUC = 1.00)
Chance
Mean ROC (AUC = 0.94 ± 0.14)
± 1 std. dev.
ROC fold 1 (AUC = 0.48)
ROC fold 2 (AUC = 1.00)
ROC fold 3 (AUC = 1.00)
ROC fold 4 (AUC = 1.00)
ROC fold 5 (AUC = 1.00)
ROC fold 6 (AUC = 1.00)
ROC fold 7 (AUC = 1.00)
ROC fold 8 (AUC = 1.00)
ROC fold 9 (AUC = 1.00)
ROC fold 10 (AUC = 1.00)
Chance
Mean ROC (AUC = 0.94 ± 0.15)
± 1 std. dev.
(AUC = 0.52)
Chance
(AUC = 0.52)
Chance
(AUC = 0.49)
Chance
ROC fold 1 (AUC = 0.59)
ROC fold 2 (AUC = 1.00)
ROC fold 3 (AUC = 0.95)
ROC fold 4 (AUC = 1.00)
ROC fold 5 (AUC = 1.00)
ROC fold 6 (AUC = 1.00)
ROC fold 7 (AUC = 1.00)
ROC fold 8 (AUC = 1.00)
ROC fold 9 (AUC = 1.00)
ROC fold 10 (AUC = 1.00)
Chance
Mean ROC (AUC = 0.95 ± 0.12)
± 1 std. dev.
ROC fold 1 (AUC = 0.60)
ROC fold 2 (AUC = 0.90)
ROC fold 3 (AUC = 0.95)
ROC fold 4 (AUC = 1.00)
ROC fold 5 (AUC = 1.00)
ROC fold 6 (AUC = 1.00)
ROC fold 7 (AUC = 1.00)
ROC fold 8 (AUC = 1.00)
ROC fold 9 (AUC = 1.00)
ROC fold 10 (AUC = 1.00)
Chance
Mean ROC (AUC = 0.94 ± 0.12)
± 1 std. dev.
ROC fold 1 (AUC = 0.47)
ROC fold 2 (AUC = 1.00)
ROC fold 3 (AUC = 1.00)
ROC fold 4 (AUC = 1.00)
ROC fold 5 (AUC = 1.00)
ROC fold 6 (AUC = 1.00)
ROC fold 7 (AUC = 1.00)
ROC fold 8 (AUC = 1.00)
ROC fold 9 (AUC = 1.00)
ROC fold 10 (AUC = 1.00)
Chance
Mean ROC (AUC = 0.94 ± 0.16)
± 1 std. dev.
(AUC = 0.53)
Chance
(AUC = 0.53)
Chance
(AUC = 0.51)
Chance

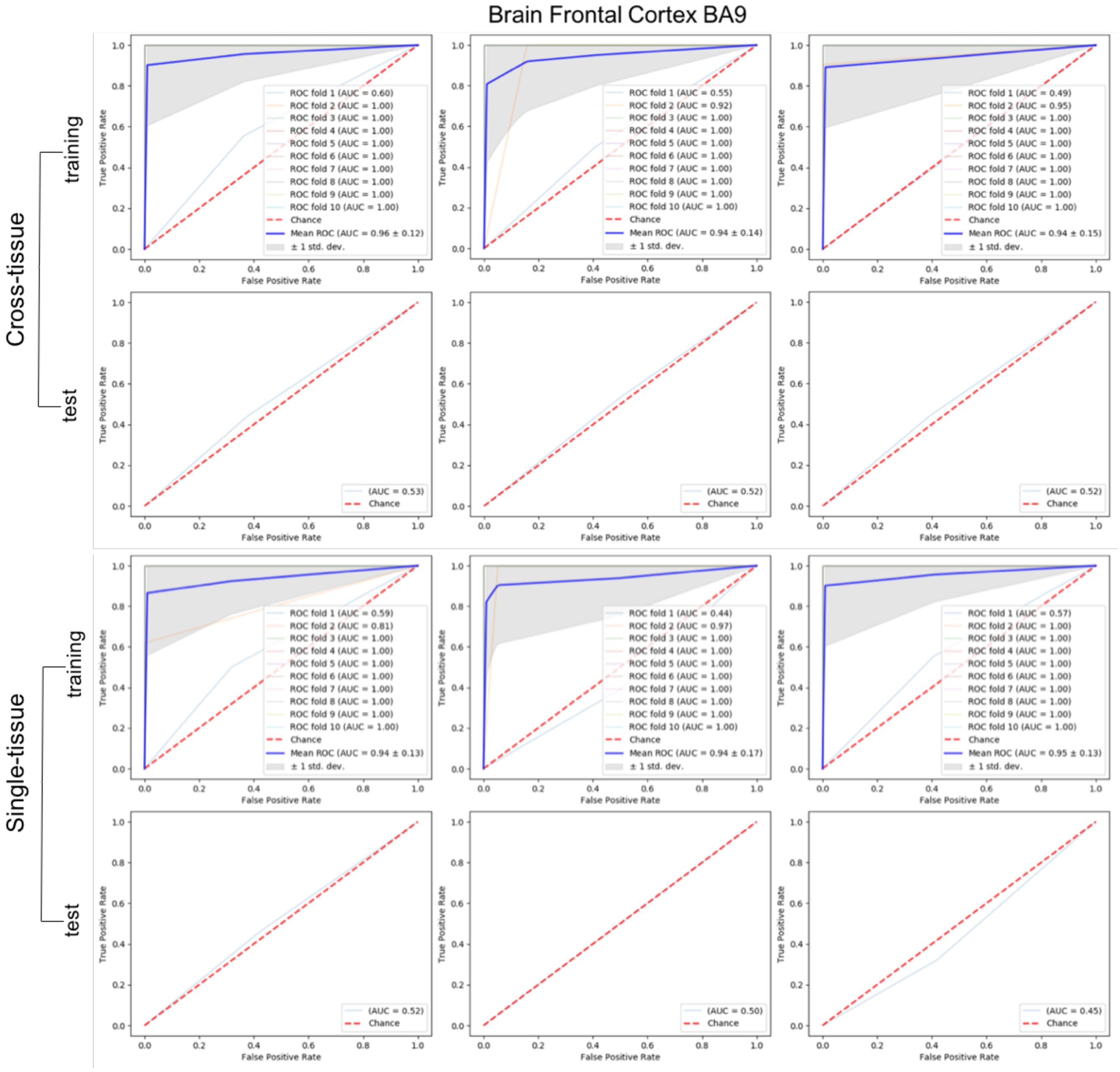
Brain Frontal Cortex BA9
Cross-tissue
training
test
Single-tissue
training
test
True Positive Rate
False Positive Rate
ROC fold 1 (AUC = 0.60)
Chance
Mean ROC (AUC = 0.96 ± 0.12)
± 1 std. dev.
ROC fold 1 (AUC = 0.55)
ROC fold 2 (AUC = 0.92)
Mean ROC (AUC = 0.94 ± 0.14)
ROC fold 1 (AUC = 0.49)
ROC fold 2 (AUC = 0.95)
Mean ROC (AUC = 0.94 ± 0.15)
(AUC = 0.53)
(AUC = 0.52)
(AUC = 0.52)
ROC fold 1 (AUC = 0.59)
ROC fold 2 (AUC = 0.81)
Mean ROC (AUC = 0.94 ± 0.13)
ROC fold 1 (AUC = 0.44)
ROC fold 2 (AUC = 0.97)
Mean ROC (AUC = 0.94 ± 0.17)
ROC fold 1 (AUC = 0.57)
ROC fold 2 (AUC = 1.00)
Mean ROC (AUC = 0.95 ± 0.13)
(AUC = 0.52)
(AUC = 0.50)
(AUC = 0.45)

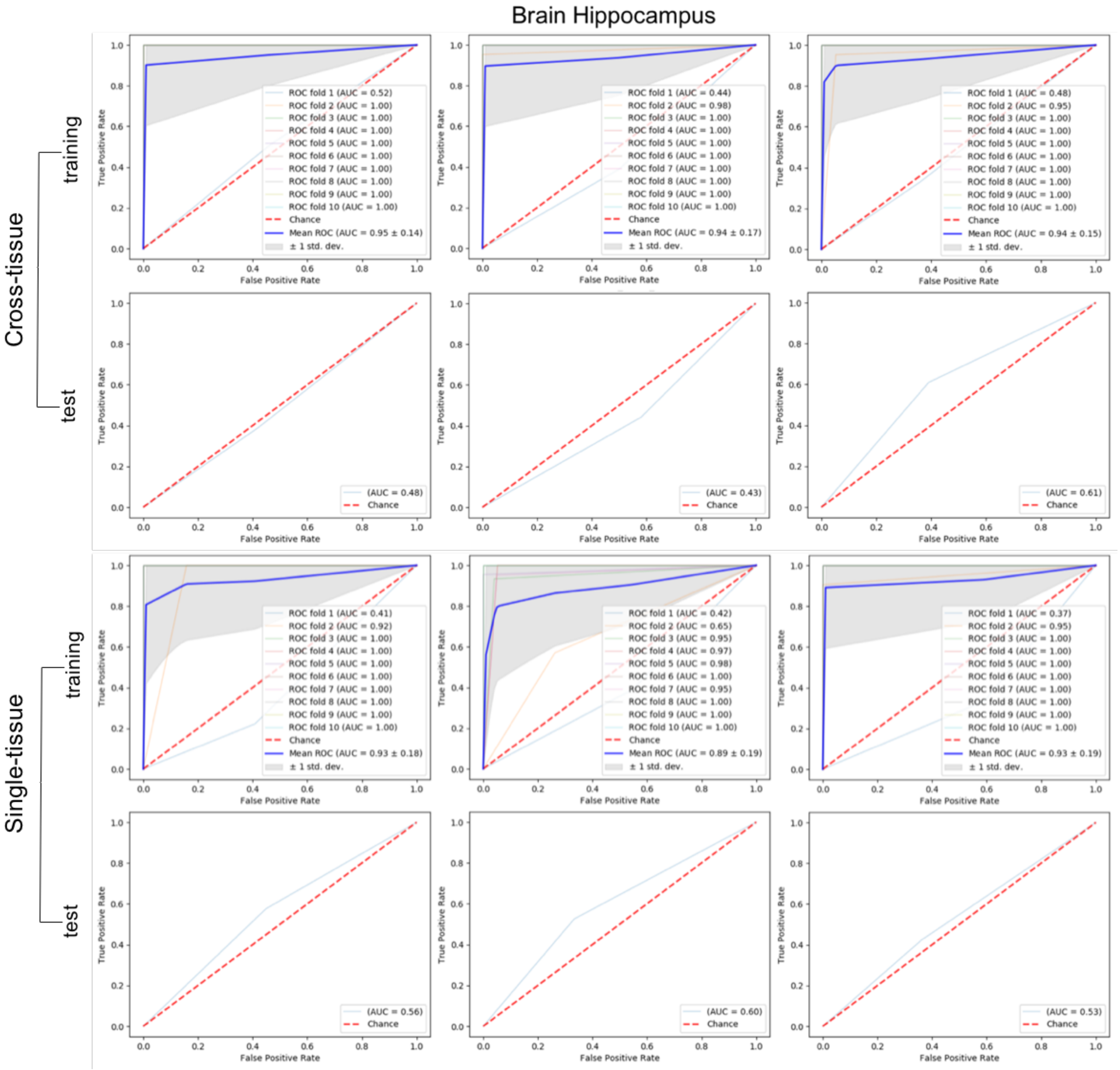
Brain Hippocampus
Cross-tissue
training
test
Single-tissue
training
test
True Positive Rate
False Positive Rate
ROC fold 1 (AUC = 0.52)
ROC fold 2 (AUC = 1.00)
ROC fold 3 (AUC = 1.00)
ROC fold 4 (AUC = 1.00)
ROC fold 5 (AUC = 1.00)
ROC fold 6 (AUC = 1.00)
ROC fold 7 (AUC = 1.00)
ROC fold 8 (AUC = 1.00)
ROC fold 9 (AUC = 1.00)
ROC fold 10 (AUC = 1.00)
Chance
Mean ROC (AUC = 0.95 ± 0.14)
± 1 std. dev.
ROC fold 1 (AUC = 0.44)
ROC fold 2 (AUC = 0.98)
ROC fold 3 (AUC = 1.00)
ROC fold 4 (AUC = 1.00)
ROC fold 5 (AUC = 1.00)
ROC fold 6 (AUC = 1.00)
ROC fold 7 (AUC = 1.00)
ROC fold 8 (AUC = 1.00)
ROC fold 9 (AUC = 1.00)
ROC fold 10 (AUC = 1.00)
Chance
Mean ROC (AUC = 0.94 ± 0.17)
± 1 std. dev.
ROC fold 1 (AUC = 0.48)
ROC fold 2 (AUC = 0.95)
ROC fold 3 (AUC = 1.00)
ROC fold 4 (AUC = 1.00)
ROC fold 5 (AUC = 1.00)
ROC fold 6 (AUC = 1.00)
ROC fold 7 (AUC = 1.00)
ROC fold 8 (AUC = 1.00)
ROC fold 9 (AUC = 1.00)
ROC fold 10 (AUC = 1.00)
Chance
Mean ROC (AUC = 0.94 ± 0.15)
± 1 std. dev.
(AUC = 0.48)
Chance
(AUC = 0.43)
Chance
(AUC = 0.61)
Chance
ROC fold 1 (AUC = 0.41)
ROC fold 2 (AUC = 0.92)
ROC fold 3 (AUC = 1.00)
ROC fold 4 (AUC = 1.00)
ROC fold 5 (AUC = 1.00)
ROC fold 6 (AUC = 1.00)
ROC fold 7 (AUC = 1.00)
ROC fold 8 (AUC = 1.00)
ROC fold 9 (AUC = 1.00)
ROC fold 10 (AUC = 1.00)
Chance
Mean ROC (AUC = 0.93 ± 0.18)
± 1 std. dev.
ROC fold 1 (AUC = 0.42)
ROC fold 2 (AUC = 0.65)
ROC fold 3 (AUC = 0.95)
ROC fold 4 (AUC = 0.97)
ROC fold 5 (AUC = 0.98)
ROC fold 6 (AUC = 1.00)
ROC fold 7 (AUC = 0.95)
ROC fold 8 (AUC = 1.00)
ROC fold 9 (AUC = 1.00)
ROC fold 10 (AUC = 1.00)
Chance
Mean ROC (AUC = 0.89 ± 0.19)
± 1 std. dev.
ROC fold 1 (AUC = 0.37)
ROC fold 2 (AUC = 0.95)
ROC fold 3 (AUC = 1.00)
ROC fold 4 (AUC = 1.00)
ROC fold 5 (AUC = 1.00)
ROC fold 6 (AUC = 1.00)
ROC fold 7 (AUC = 1.00)
ROC fold 8 (AUC = 1.00)
ROC fold 9 (AUC = 1.00)
ROC fold 10 (AUC = 1.00)
Chance
Mean ROC (AUC = 0.93 ± 0.19)
± 1 std. dev.
(AUC = 0.56)
Chance
(AUC = 0.60)
Chance
(AUC = 0.53)
Chance

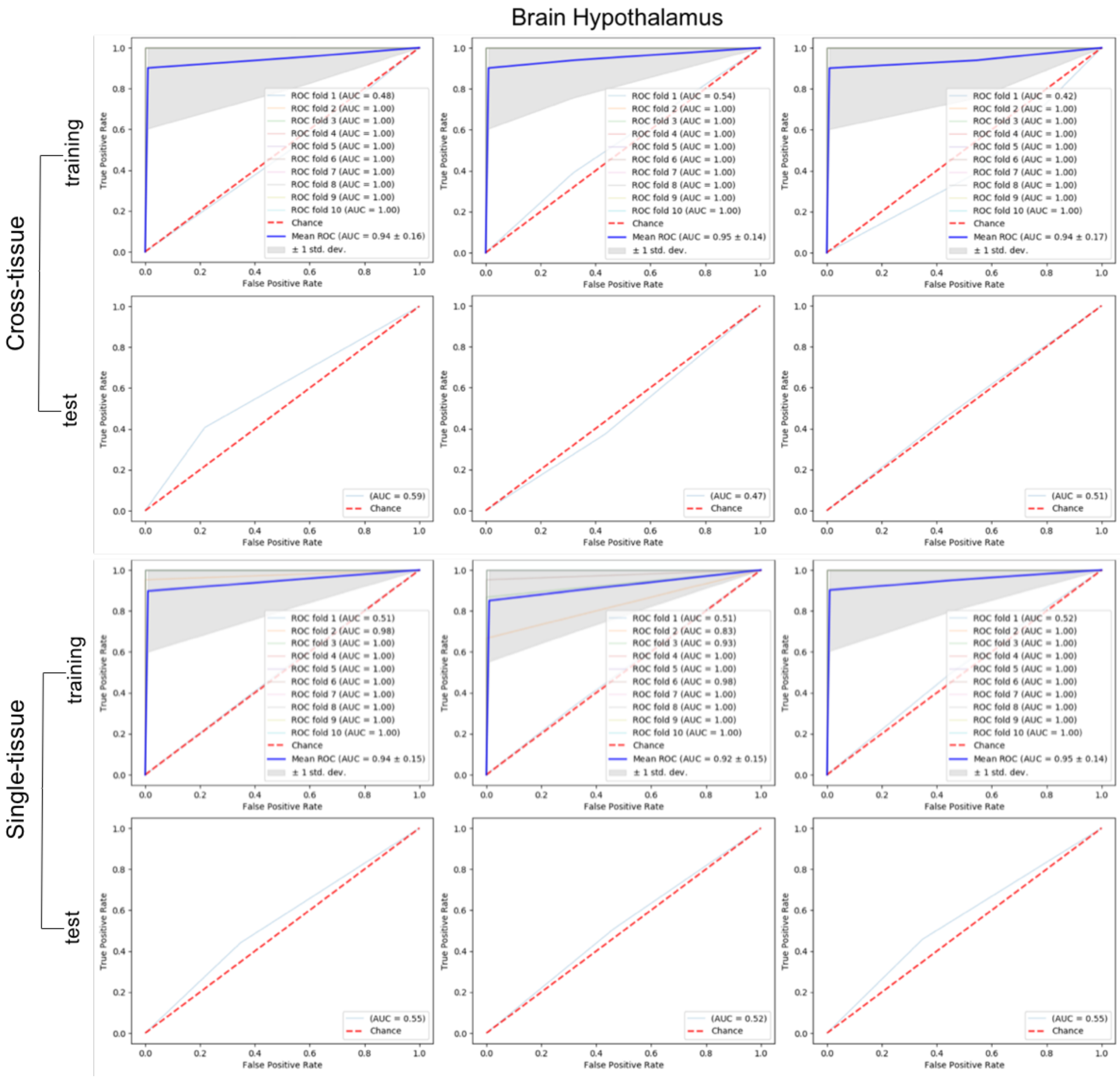
Brain Hypothalamus
Cross-tissue
Single-tissue
training
test
True Positive Rate
False Positive Rate
ROC fold 1 (AUC = 0.48)
ROC fold 2 (AUC = 1.00)
ROC fold 3 (AUC = 1.00)
ROC fold 4 (AUC = 1.00)
ROC fold 5 (AUC = 1.00)
ROC fold 6 (AUC = 1.00)
ROC fold 7 (AUC = 1.00)
ROC fold 8 (AUC = 1.00)
ROC fold 9 (AUC = 1.00)
ROC fold 10 (AUC = 1.00)
Chance
Mean ROC (AUC = 0.94 ± 0.16)
± 1 std. dev.
ROC fold 1 (AUC = 0.54)
ROC fold 2 (AUC = 1.00)
ROC fold 3 (AUC = 1.00)
ROC fold 4 (AUC = 1.00)
ROC fold 5 (AUC = 1.00)
ROC fold 6 (AUC = 1.00)
ROC fold 7 (AUC = 1.00)
ROC fold 8 (AUC = 1.00)
ROC fold 9 (AUC = 1.00)
ROC fold 10 (AUC = 1.00)
Chance
Mean ROC (AUC = 0.95 ± 0.14)
± 1 std. dev.
ROC fold 1 (AUC = 0.42)
ROC fold 2 (AUC = 1.00)
ROC fold 3 (AUC = 1.00)
ROC fold 4 (AUC = 1.00)
ROC fold 5 (AUC = 1.00)
ROC fold 6 (AUC = 1.00)
ROC fold 7 (AUC = 1.00)
ROC fold 8 (AUC = 1.00)
ROC fold 9 (AUC = 1.00)
ROC fold 10 (AUC = 1.00)
Chance
Mean ROC (AUC = 0.94 ± 0.17)
± 1 std. dev.
(AUC = 0.59)
Chance
(AUC = 0.47)
Chance
(AUC = 0.51)
Chance
ROC fold 1 (AUC = 0.51)
ROC fold 2 (AUC = 0.98)
ROC fold 3 (AUC = 1.00)
ROC fold 4 (AUC = 1.00)
ROC fold 5 (AUC = 1.00)
ROC fold 6 (AUC = 1.00)
ROC fold 7 (AUC = 1.00)
ROC fold 8 (AUC = 1.00)
ROC fold 9 (AUC = 1.00)
ROC fold 10 (AUC = 1.00)
Chance
Mean ROC (AUC = 0.94 ± 0.15)
± 1 std. dev.
ROC fold 1 (AUC = 0.51)
ROC fold 2 (AUC = 0.83)
ROC fold 3 (AUC = 0.93)
ROC fold 4 (AUC = 1.00)
ROC fold 5 (AUC = 1.00)
ROC fold 6 (AUC = 0.98)
ROC fold 7 (AUC = 1.00)
ROC fold 8 (AUC = 1.00)
ROC fold 9 (AUC = 1.00)
ROC fold 10 (AUC = 1.00)
Chance
Mean ROC (AUC = 0.92 ± 0.15)
± 1 std. dev.
ROC fold 1 (AUC = 0.52)
ROC fold 2 (AUC = 1.00)
ROC fold 3 (AUC = 1.00)
ROC fold 4 (AUC = 1.00)
ROC fold 5 (AUC = 1.00)
ROC fold 6 (AUC = 1.00)
ROC fold 7 (AUC = 1.00)
ROC fold 8 (AUC = 1.00)
ROC fold 9 (AUC = 1.00)
ROC fold 10 (AUC = 1.00)
Chance
Mean ROC (AUC = 0.95 ± 0.14)
± 1 std. dev.
(AUC = 0.55)
Chance
(AUC = 0.52)
Chance
(AUC = 0.55)
Chance

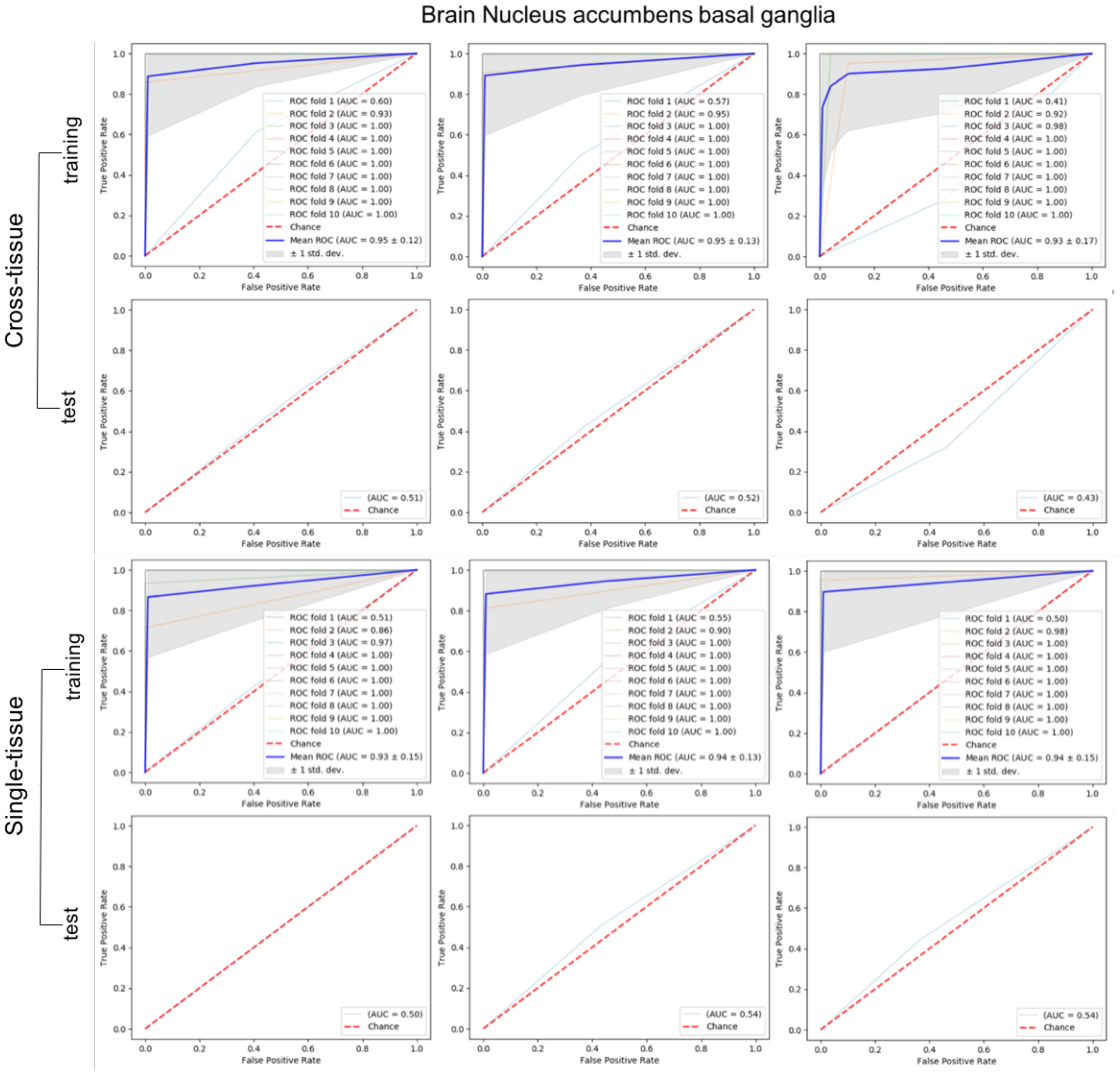
Brain Nucleus accumbens basal ganglia
Cross-tissue
training
test
Single-tissue
training
test
True Positive Rate
False Positive Rate
ROC fold 1 (AUC = 0.60)
ROC fold 2 (AUC = 0.93)
ROC fold 3 (AUC = 1.00)
ROC fold 4 (AUC = 1.00)
ROC fold 5 (AUC = 1.00)
ROC fold 6 (AUC = 1.00)
ROC fold 7 (AUC = 1.00)
ROC fold 8 (AUC = 1.00)
ROC fold 9 (AUC = 1.00)
ROC fold 10 (AUC = 1.00)
Chance
Mean ROC (AUC = 0.95 ± 0.12)
± 1 std. dev.
ROC fold 1 (AUC = 0.57)
ROC fold 2 (AUC = 0.95)
ROC fold 3 (AUC = 1.00)
ROC fold 4 (AUC = 1.00)
ROC fold 5 (AUC = 1.00)
ROC fold 6 (AUC = 1.00)
ROC fold 7 (AUC = 1.00)
ROC fold 8 (AUC = 1.00)
ROC fold 9 (AUC = 1.00)
ROC fold 10 (AUC = 1.00)
Chance
Mean ROC (AUC = 0.95 ± 0.13)
± 1 std. dev.
ROC fold 1 (AUC = 0.41)
ROC fold 2 (AUC = 0.92)
ROC fold 3 (AUC = 0.98)
ROC fold 4 (AUC = 1.00)
ROC fold 5 (AUC = 1.00)
ROC fold 6 (AUC = 1.00)
ROC fold 7 (AUC = 1.00)
ROC fold 8 (AUC = 1.00)
ROC fold 9 (AUC = 1.00)
ROC fold 10 (AUC = 1.00)
Chance
Mean ROC (AUC = 0.93 ± 0.17)
± 1 std. dev.
(AUC = 0.51)
Chance
(AUC = 0.52)
Chance
(AUC = 0.43)
Chance
ROC fold 1 (AUC = 0.51)
ROC fold 2 (AUC = 0.86)
ROC fold 3 (AUC = 0.97)
ROC fold 4 (AUC = 1.00)
ROC fold 5 (AUC = 1.00)
ROC fold 6 (AUC = 1.00)
ROC fold 7 (AUC = 1.00)
ROC fold 8 (AUC = 1.00)
ROC fold 9 (AUC = 1.00)
ROC fold 10 (AUC = 1.00)
Chance
Mean ROC (AUC = 0.93 ± 0.15)
± 1 std. dev.
ROC fold 1 (AUC = 0.55)
ROC fold 2 (AUC = 0.90)
ROC fold 3 (AUC = 1.00)
ROC fold 4 (AUC = 1.00)
ROC fold 5 (AUC = 1.00)
ROC fold 6 (AUC = 1.00)
ROC fold 7 (AUC = 1.00)
ROC fold 8 (AUC = 1.00)
ROC fold 9 (AUC = 1.00)
ROC fold 10 (AUC = 1.00)
Chance
Mean ROC (AUC = 0.94 ± 0.13)
± 1 std. dev.
ROC fold 1 (AUC = 0.50)
ROC fold 2 (AUC = 0.98)
ROC fold 3 (AUC = 1.00)
ROC fold 4 (AUC = 1.00)
ROC fold 5 (AUC = 1.00)
ROC fold 6 (AUC = 1.00)
ROC fold 7 (AUC = 1.00)
ROC fold 8 (AUC = 1.00)
ROC fold 9 (AUC = 1.00)
ROC fold 10 (AUC = 1.00)
Chance
Mean ROC (AUC = 0.94 ± 0.15)
± 1 std. dev.
(AUC = 0.50)
Chance
(AUC = 0.54)
Chance
(AUC = 0.54)
Chance

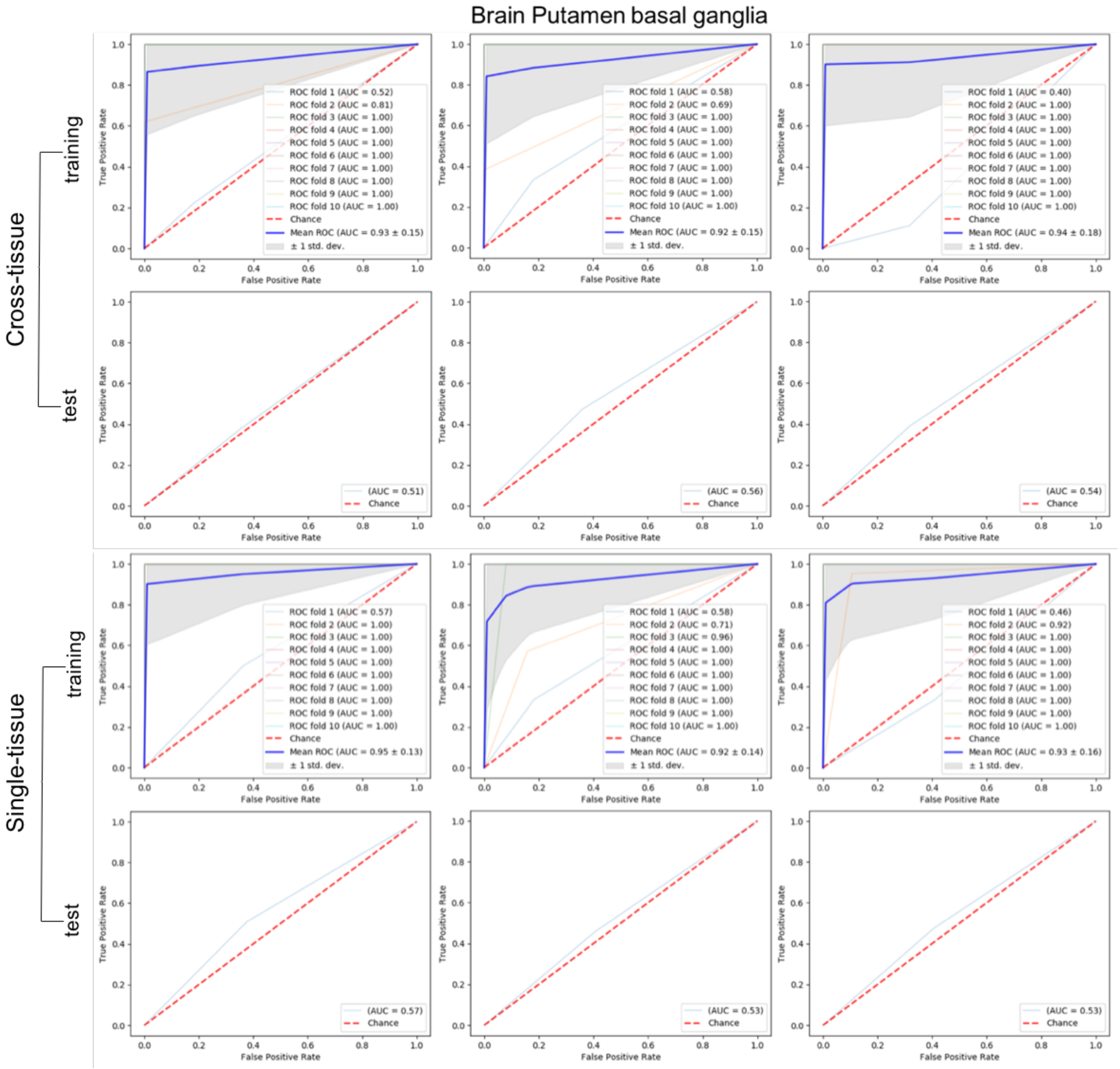
Brain Putamen basal ganglia
Cross-tissue
training
test
Single-tissue
training
test
True Positive Rate
False Positive Rate
ROC fold 1 (AUC = 0.52)
ROC fold 2 (AUC = 0.81)
ROC fold 3 (AUC = 1.00)
ROC fold 4 (AUC = 1.00)
ROC fold 5 (AUC = 1.00)
ROC fold 6 (AUC = 1.00)
ROC fold 7 (AUC = 1.00)
ROC fold 8 (AUC = 1.00)
ROC fold 9 (AUC = 1.00)
ROC fold 10 (AUC = 1.00)
Chance
Mean ROC (AUC = 0.93 ± 0.15)
± 1 std. dev.
ROC fold 1 (AUC = 0.58)
ROC fold 2 (AUC = 0.69)
ROC fold 3 (AUC = 1.00)
ROC fold 4 (AUC = 1.00)
ROC fold 5 (AUC = 1.00)
ROC fold 6 (AUC = 1.00)
ROC fold 7 (AUC = 1.00)
ROC fold 8 (AUC = 1.00)
ROC fold 9 (AUC = 1.00)
ROC fold 10 (AUC = 1.00)
Chance
Mean ROC (AUC = 0.92 ± 0.15)
± 1 std. dev.
ROC fold 1 (AUC = 0.40)
ROC fold 2 (AUC = 1.00)
ROC fold 3 (AUC = 1.00)
ROC fold 4 (AUC = 1.00)
ROC fold 5 (AUC = 1.00)
ROC fold 6 (AUC = 1.00)
ROC fold 7 (AUC = 1.00)
ROC fold 8 (AUC = 1.00)
ROC fold 9 (AUC = 1.00)
ROC fold 10 (AUC = 1.00)
Chance
Mean ROC (AUC = 0.94 ± 0.18)
± 1 std. dev.
(AUC = 0.51)
Chance
(AUC = 0.56)
Chance
(AUC = 0.54)
Chance
ROC fold 1 (AUC = 0.57)
ROC fold 2 (AUC = 1.00)
ROC fold 3 (AUC = 1.00)
ROC fold 4 (AUC = 1.00)
ROC fold 5 (AUC = 1.00)
ROC fold 6 (AUC = 1.00)
ROC fold 7 (AUC = 1.00)
ROC fold 8 (AUC = 1.00)
ROC fold 9 (AUC = 1.00)
ROC fold 10 (AUC = 1.00)
Chance
Mean ROC (AUC = 0.95 ± 0.13)
± 1 std. dev.
ROC fold 1 (AUC = 0.58)
ROC fold 2 (AUC = 0.71)
ROC fold 3 (AUC = 0.96)
ROC fold 4 (AUC = 1.00)
ROC fold 5 (AUC = 1.00)
ROC fold 6 (AUC = 1.00)
ROC fold 7 (AUC = 1.00)
ROC fold 8 (AUC = 1.00)
ROC fold 9 (AUC = 1.00)
ROC fold 10 (AUC = 1.00)
Chance
Mean ROC (AUC = 0.92 ± 0.14)
± 1 std. dev.
ROC fold 1 (AUC = 0.46)
ROC fold 2 (AUC = 0.92)
ROC fold 3 (AUC = 1.00)
ROC fold 4 (AUC = 1.00)
ROC fold 5 (AUC = 1.00)
ROC fold 6 (AUC = 1.00)
ROC fold 7 (AUC = 1.00)
ROC fold 8 (AUC = 1.00)
ROC fold 9 (AUC = 1.00)
ROC fold 10 (AUC = 1.00)
Chance
Mean ROC (AUC = 0.93 ± 0.16)
± 1 std. dev.
(AUC = 0.57)
Chance
(AUC = 0.53)
Chance
(AUC = 0.53)
Chance

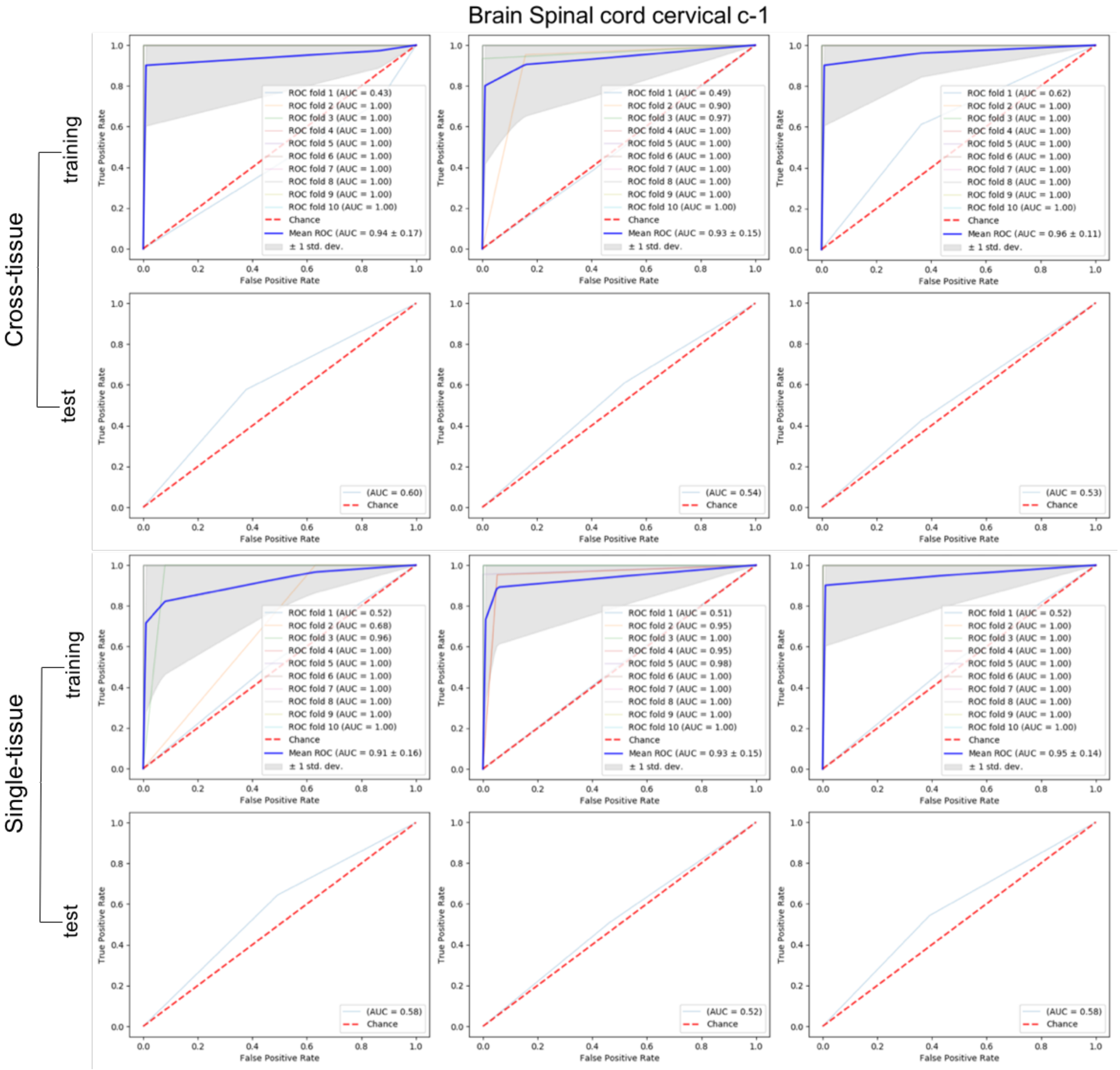
Brain Spinal cord cervical c-1
Cross-tissue
training
test
Single-tissue
training
test
True Positive Rate
False Positive Rate
ROC fold 1 (AUC = 0.43)
ROC fold 2 (AUC = 1.00)
ROC fold 3 (AUC = 1.00)
ROC fold 4 (AUC = 1.00)
ROC fold 5 (AUC = 1.00)
ROC fold 6 (AUC = 1.00)
ROC fold 7 (AUC = 1.00)
ROC fold 8 (AUC = 1.00)
ROC fold 9 (AUC = 1.00)
ROC fold 10 (AUC = 1.00)
Chance
Mean ROC (AUC = 0.94 ± 0.17)
± 1 std. dev.
ROC fold 1 (AUC = 0.49)
ROC fold 2 (AUC = 0.90)
ROC fold 3 (AUC = 0.97)
ROC fold 4 (AUC = 1.00)
ROC fold 5 (AUC = 1.00)
ROC fold 6 (AUC = 1.00)
ROC fold 7 (AUC = 1.00)
ROC fold 8 (AUC = 1.00)
ROC fold 9 (AUC = 1.00)
ROC fold 10 (AUC = 1.00)
Chance
Mean ROC (AUC = 0.93 ± 0.15)
± 1 std. dev.
ROC fold 1 (AUC = 0.62)
ROC fold 2 (AUC = 1.00)
ROC fold 3 (AUC = 1.00)
ROC fold 4 (AUC = 1.00)
ROC fold 5 (AUC = 1.00)
ROC fold 6 (AUC = 1.00)
ROC fold 7 (AUC = 1.00)
ROC fold 8 (AUC = 1.00)
ROC fold 9 (AUC = 1.00)
ROC fold 10 (AUC = 1.00)
Chance
Mean ROC (AUC = 0.96 ± 0.11)
± 1 std. dev.
(AUC = 0.60)
Chance
(AUC = 0.54)
Chance
(AUC = 0.53)
Chance
ROC fold 1 (AUC = 0.52)
ROC fold 2 (AUC = 0.68)
ROC fold 3 (AUC = 0.96)
ROC fold 4 (AUC = 1.00)
ROC fold 5 (AUC = 1.00)
ROC fold 6 (AUC = 1.00)
ROC fold 7 (AUC = 1.00)
ROC fold 8 (AUC = 1.00)
ROC fold 9 (AUC = 1.00)
ROC fold 10 (AUC = 1.00)
Chance
Mean ROC (AUC = 0.91 ± 0.16)
± 1 std. dev.
ROC fold 1 (AUC = 0.51)
ROC fold 2 (AUC = 0.95)
ROC fold 3 (AUC = 1.00)
ROC fold 4 (AUC = 0.95)
ROC fold 5 (AUC = 0.98)
ROC fold 6 (AUC = 1.00)
ROC fold 7 (AUC = 1.00)
ROC fold 8 (AUC = 1.00)
ROC fold 9 (AUC = 1.00)
ROC fold 10 (AUC = 1.00)
Chance
Mean ROC (AUC = 0.93 ± 0.15)
± 1 std. dev.
ROC fold 1 (AUC = 0.52)
ROC fold 2 (AUC = 1.00)
ROC fold 3 (AUC = 1.00)
ROC fold 4 (AUC = 1.00)
ROC fold 5 (AUC = 1.00)
ROC fold 6 (AUC = 1.00)
ROC fold 7 (AUC = 1.00)
ROC fold 8 (AUC = 1.00)
ROC fold 9 (AUC = 1.00)
ROC fold 10 (AUC = 1.00)
Chance
Mean ROC (AUC = 0.95 ± 0.14)
± 1 std. dev.
(AUC = 0.58)
Chance
(AUC = 0.52)
Chance
(AUC = 0.58)
Chance

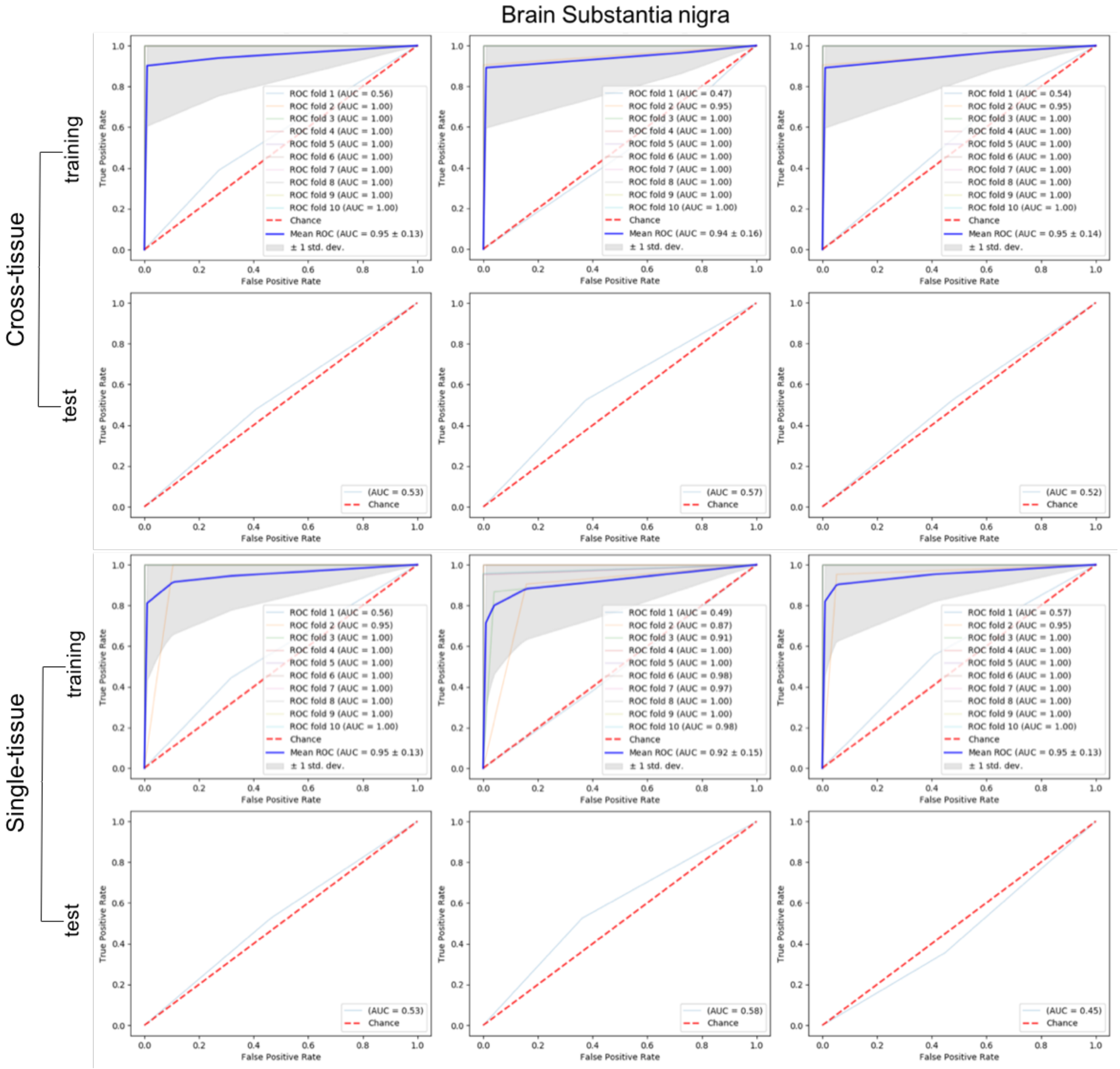
Brain Substantia nigra
Cross-tissue
training
test
Single-tissue
training
test
True Positive Rate
False Positive Rate
ROC fold 1 (AUC = 0.56)
ROC fold 2 (AUC = 1.00)
ROC fold 3 (AUC = 1.00)
ROC fold 4 (AUC = 1.00)
ROC fold 5 (AUC = 1.00)
ROC fold 6 (AUC = 1.00)
ROC fold 7 (AUC = 1.00)
ROC fold 8 (AUC = 1.00)
ROC fold 9 (AUC = 1.00)
ROC fold 10 (AUC = 1.00)
Chance
Mean ROC (AUC = 0.95 ± 0.13)
± 1 std. dev.
ROC fold 1 (AUC = 0.47)
ROC fold 2 (AUC = 0.95)
ROC fold 3 (AUC = 1.00)
ROC fold 4 (AUC = 1.00)
ROC fold 5 (AUC = 1.00)
ROC fold 6 (AUC = 1.00)
ROC fold 7 (AUC = 1.00)
ROC fold 8 (AUC = 1.00)
ROC fold 9 (AUC = 1.00)
ROC fold 10 (AUC = 1.00)
Chance
Mean ROC (AUC = 0.94 ± 0.16)
± 1 std. dev.
ROC fold 1 (AUC = 0.54)
ROC fold 2 (AUC = 0.95)
ROC fold 3 (AUC = 1.00)
ROC fold 4 (AUC = 1.00)
ROC fold 5 (AUC = 1.00)
ROC fold 6 (AUC = 1.00)
ROC fold 7 (AUC = 1.00)
ROC fold 8 (AUC = 1.00)
ROC fold 9 (AUC = 1.00)
ROC fold 10 (AUC = 1.00)
Chance
Mean ROC (AUC = 0.95 ± 0.14)
± 1 std. dev.
(AUC = 0.53)
Chance
(AUC = 0.57)
Chance
(AUC = 0.52)
Chance
ROC fold 1 (AUC = 0.56)
ROC fold 2 (AUC = 0.95)
ROC fold 3 (AUC = 1.00)
ROC fold 4 (AUC = 1.00)
ROC fold 5 (AUC = 1.00)
ROC fold 6 (AUC = 1.00)
ROC fold 7 (AUC = 1.00)
ROC fold 8 (AUC = 1.00)
ROC fold 9 (AUC = 1.00)
ROC fold 10 (AUC = 1.00)
Chance
Mean ROC (AUC = 0.95 ± 0.13)
± 1 std. dev.
ROC fold 1 (AUC = 0.49)
ROC fold 2 (AUC = 0.87)
ROC fold 3 (AUC = 0.91)
ROC fold 4 (AUC = 1.00)
ROC fold 5 (AUC = 1.00)
ROC fold 6 (AUC = 0.98)
ROC fold 7 (AUC = 0.97)
ROC fold 8 (AUC = 1.00)
ROC fold 9 (AUC = 1.00)
ROC fold 10 (AUC = 0.98)
Chance
Mean ROC (AUC = 0.92 ± 0.15)
± 1 std. dev.
ROC fold 1 (AUC = 0.57)
ROC fold 2 (AUC = 0.95)
ROC fold 3 (AUC = 1.00)
ROC fold 4 (AUC = 1.00)
ROC fold 5 (AUC = 1.00)
ROC fold 6 (AUC = 1.00)
ROC fold 7 (AUC = 1.00)
ROC fold 8 (AUC = 1.00)
ROC fold 9 (AUC = 1.00)
ROC fold 10 (AUC = 1.00)
Chance
Mean ROC (AUC = 0.95 ± 0.13)
± 1 std. dev.
(AUC = 0.53)
Chance
(AUC = 0.58)
Chance
(AUC = 0.45)
Chance

2.7 Filtering results

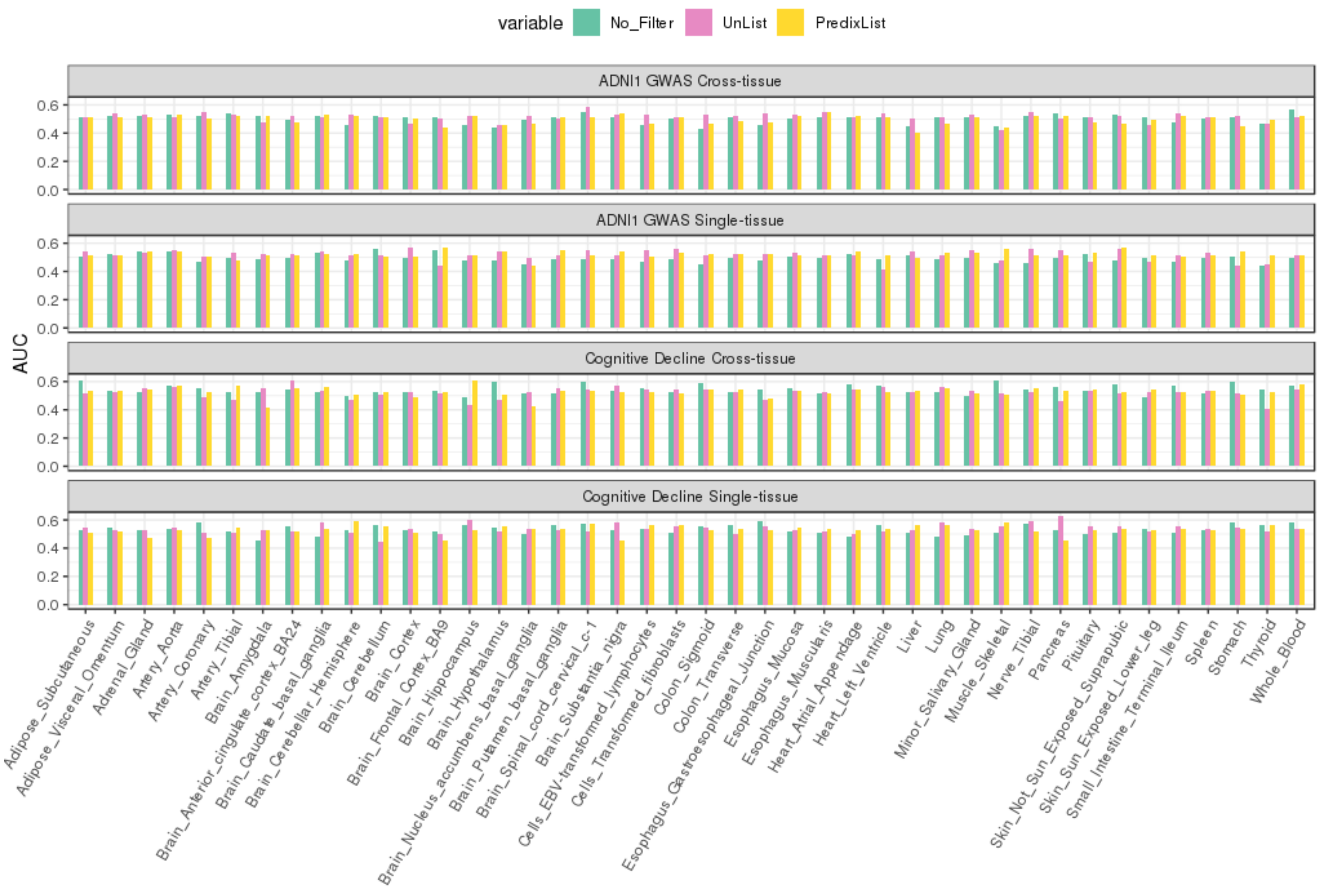

Figure S4: ADNI1-GWAS and Cognitive Decline feature selection evaluation. In green are reported the AUCs on test sets for the no filter application, in red for List-unsupervised and in yellow for List-PrediXcan. The top two panels reports respectively the cross-tissue and single-tissue performance for ADNI1-GWAS, the last two panels the cross-tissue and single-tissue performance for Cognitive Decline dataset. In both dataset, feature filtering improved the classification in almost all the Brain tissues.

2.8 Cognitive decline: boxplot

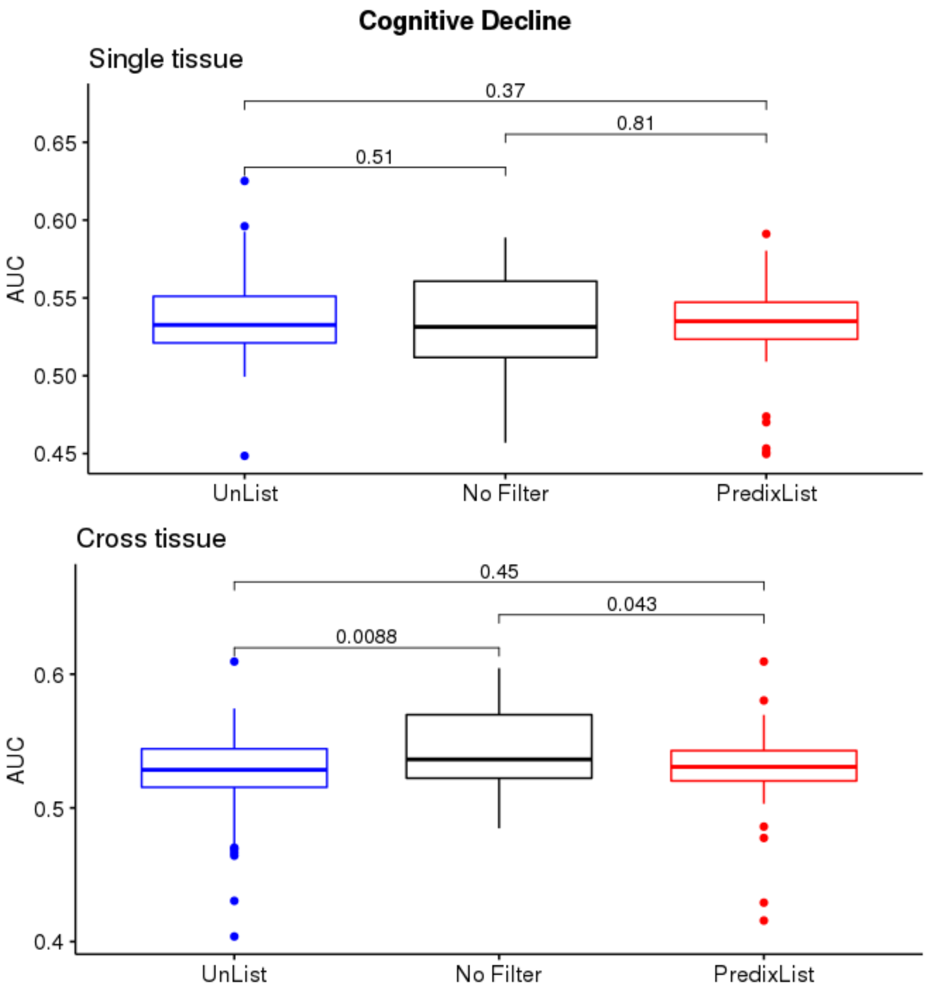

Figure S5: Cognitive Decline feature selection evaluation. In blue are reported the performance with List-unsupervised, in black without the filter and in red with List-PrediXcan for models trained for the single-tissue analysis (top panel) and cross-tissue analysis (bottom panel). We didn't have evident improvement with the filter approach.

2.9 Cognitive decline cross-tissue analysis: Heatmap

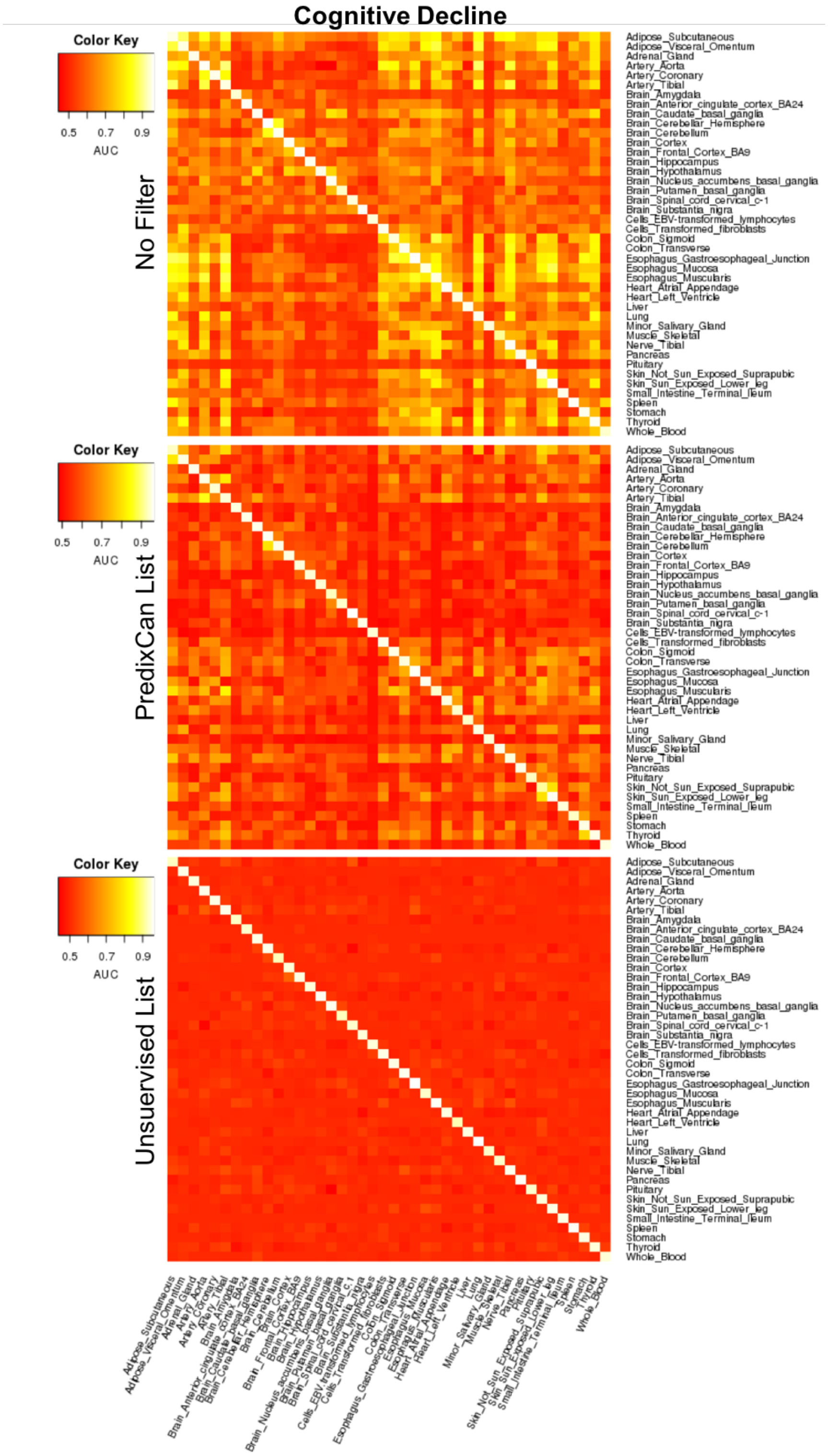

Figure S6: Cognitive Decline cross-tissues performance. By column we can observe how much a model trained on a tissue is able to recognize without mistakes (AUC) AD/not-AD subjects from data related to different tissues. On the diagonal is reported, for each tissue, the AUC obtained for that model during the training.

3 FILTERING LISTS

Table S5: List-Unsupervised

**Adipose Subcutaneous**	ENSG00000003400.10	ENSG00000004975.7	ENSG00000005448.12	ENSG00000006704.6
	ENSG00000008226.15	ENSG00000010017.9	ENSG00000010282.10	ENSG00000011143.12
	ENSG00000042062.7	ENSG00000047249.12	ENSG00000049541.6	ENSG00000058668.10
	ENSG00000061273.13	ENSG00000063241.3	ENSG00000068383.14	ENSG00000072135.8
	ENSG00000074696.8	ENSG00000075223.9	ENSG00000075886.10	ENSG00000076984.13
	ENSG00000085741.8	ENSG00000086065.9	ENSG00000086475.10	ENSG00000087494.11
	ENSG00000091129.15	ENSG00000099139.9	ENSG00000099246.12	ENSG00000100097.7
	ENSG00000100281.9	ENSG00000100365.10	ENSG00000100523.10	ENSG00000101150.13
	ENSG00000101624.6	ENSG00000101670.7	ENSG00000102780.12	ENSG00000103160.7
	ENSG00000104951.11	ENSG00000104960.11	ENSG00000105254.7	ENSG00000105707.9
	ENSG00000105792.15	ENSG00000106261.12	ENSG00000106608.12	ENSG00000106952.3
	ENSG00000107443.11	ENSG00000107625.8	ENSG00000108061.7	ENSG00000108387.10
	ENSG00000108953.12	ENSG00000109066.9	ENSG00000111262.4	ENSG00000111729.8
	ENSG00000112149.5	ENSG00000112186.7	ENSG00000112394.12	ENSG00000113161.11
	ENSG00000113209.6	ENSG00000113758.9	ENSG00000114738.6	ENSG00000115274.10
	ENSG00000116703.12	ENSG00000117620.8	ENSG00000117862.7	ENSG00000118894.10
	ENSG00000119703.12	ENSG00000120093.7	ENSG00000120686.7	ENSG00000121335.10
	ENSG00000121848.9	ENSG00000122733.11	ENSG00000123360.7	ENSG00000124215.12
	ENSG00000125817.7	ENSG00000126251.5	ENSG00000127511.5	ENSG00000128512.15
	ENSG00000130173.9	ENSG00000130303.8	ENSG00000132321.12	ENSG00000133048.8
	ENSG00000133114.13	ENSG00000134508.8	ENSG00000134571.6	ENSG00000134817.9
	ENSG00000135775.9	ENSG00000135945.5	ENSG00000136160.10	ENSG00000136267.9
	ENSG00000137273.3	ENSG00000137692.7	ENSG00000138160.4	ENSG00000138785.10
	ENSG00000139292.8	ENSG00000139567.8	ENSG00000139684.9	ENSG00000140254.8
	ENSG00000140280.9	ENSG00000140395.4	ENSG00000141441.11	ENSG00000141522.7
	ENSG00000142408.2	ENSG00000142949.12	ENSG00000143157.7	ENSG00000143546.5
	ENSG00000143740.10	ENSG00000144395.13	ENSG00000145088.4	ENSG00000147614.3
	ENSG00000148482.7	ENSG00000148926.5	ENSG00000149634.4	ENSG00000150459.8
	ENSG00000151014.4	ENSG00000151500.10	ENSG00000151502.6	ENSG00000152620.8
	ENSG00000153094.17	ENSG00000153575.6	ENSG00000154122.8	ENSG00000154359.8
	ENSG00000154654.10	ENSG00000155666.7	ENSG00000155755.14	ENSG00000156096.8
	ENSG00000156127.6	ENSG00000157954.10	ENSG00000158055.11	ENSG00000159189.7
	ENSG00000159674.7	ENSG00000160221.12	ENSG00000160602.9	ENSG00000161911.7
	ENSG00000162434.7	ENSG00000163106.6	ENSG00000163449.6	ENSG00000164087.3
	ENSG00000164620.4	ENSG00000164675.6	ENSG00000165156.10	ENSG00000165417.7
	ENSG00000165516.6	ENSG00000166091.15	ENSG00000166340.10	ENSG00000166342.14
	ENSG00000166471.6	ENSG00000166557.8	ENSG00000166704.7	ENSG00000167207.7
	ENSG00000167281.14	ENSG00000167972.9	ENSG00000167985.2	ENSG00000168079.12
	ENSG00000168658.14	ENSG00000168883.15	ENSG00000169174.9	ENSG00000169403.7
	ENSG00000169908.6	ENSG00000169955.6	ENSG00000170185.5	ENSG00000170584.6
	ENSG00000170921.10	ENSG00000170989.8	ENSG00000171219.8	ENSG00000171467.11
	ENSG00000173546.7	ENSG00000174013.7	ENSG00000174799.6	ENSG00000175564.8
	ENSG00000175664.5	ENSG00000176055.9	ENSG00000176153.10	ENSG00000176371.9
	ENSG00000177556.7	ENSG00000178177.10	ENSG00000178789.4	ENSG00000179270.6
	ENSG00000179580.5	ENSG00000179611.2	ENSG00000180178.6	ENSG00000180611.6

	ENSG00000180801.11	ENSG00000181744.4	ENSG00000181982.13	ENSG00000182796.8
	ENSG00000183055.5	ENSG00000183576.8	ENSG00000183801.3	ENSG00000183888.4
	ENSG00000184451.5	ENSG00000184786.4	ENSG00000184903.5	ENSG00000185527.7
	ENSG00000185594.4	ENSG00000185897.6	ENSG00000186188.6	ENSG00000186197.8
	ENSG00000187566.3	ENSG00000188242.4	ENSG00000188868.9	ENSG00000189068.5
	ENSG00000196639.6	ENSG00000196865.4	ENSG00000197409.6	ENSG00000197757.7
	ENSG00000197930.8	ENSG00000198517.5	ENSG00000203276.2	ENSG00000203814.5
	ENSG00000204150.7	ENSG00000204186.3	ENSG00000205629.7	ENSG00000213051.3
	ENSG00000213197.3	ENSG00000213225.6	ENSG00000213261.3	ENSG00000213492.2
	ENSG00000213930.7	ENSG00000214013.5	ENSG00000214146.2	ENSG00000214362.2
	ENSG00000214561.3	ENSG00000214688.4	ENSG00000216895.4	ENSG00000224186.4
	ENSG00000224956.5	ENSG00000225930.3	ENSG00000227082.1	ENSG00000227477.1
	ENSG00000228008.1	ENSG00000228223.1	ENSG00000228397.1	ENSG00000228522.2
	ENSG00000229809.4	ENSG00000230154.1	ENSG00000230850.3	ENSG00000231322.1
	ENSG00000232810.3	ENSG00000232833.4	ENSG00000233122.1	ENSG00000233610.1
	ENSG00000233895.1	ENSG00000234065.2	ENSG00000234383.1	ENSG00000234536.1
	ENSG00000234862.1	ENSG00000236136.1	ENSG00000236184.1	ENSG00000236976.1
	ENSG00000239486.1	ENSG00000239804.1	ENSG00000240350.1	ENSG00000240682.5
	ENSG00000244556.1	ENSG00000245651.2	ENSG00000246548.3	ENSG00000246922.4
	ENSG00000247134.2	ENSG00000248916.1	ENSG00000249679.1	ENSG00000250238.1
	ENSG00000251169.2	ENSG00000251229.1	ENSG00000253276.1	ENSG00000253304.1
	ENSG00000253516.1	ENSG00000254786.1	ENSG00000255561.2	ENSG00000255595.1
	ENSG00000255735.1	ENSG00000256167.1	ENSG00000256361.1	ENSG00000256540.1
	ENSG00000257259.1	ENSG00000258811.1	ENSG00000259758.1	ENSG00000260518.1
	ENSG00000263105.1	ENSG00000264016.2	ENSG00000266651.1	ENSG00000266935.1
	ENSG00000267018.1	ENSG00000267289.1	ENSG00000267416.1	ENSG00000268087.1
	ENSG00000268172.1	ENSG00000268734.1	ENSG00000269097.1	ENSG00000269510.1
	ENSG00000269543.1	ENSG00000271734.1	ENSG00000271856.1	ENSG00000272235.1
	ENSG00000272347.1	ENSG00000272425.1	ENSG00000272682.1	ENSG00000272908.1
	ENSG00000272966.1			
**Adipose Visceral Omentum**	ENSG00000004975.7	ENSG00000005448.12	ENSG00000006704.6	ENSG00000010282.10
	ENSG00000011028.9	ENSG00000023734.6	ENSG00000037280.11	ENSG00000047249.12
	ENSG00000058668.10	ENSG00000061656.5	ENSG00000066336.7	ENSG00000074696.8
	ENSG00000076513.12	ENSG00000079263.14	ENSG00000081052.10	ENSG00000081818.1
	ENSG00000085741.8	ENSG00000089048.10	ENSG00000092871.12	ENSG00000099194.5
	ENSG00000099246.12	ENSG00000100281.9	ENSG00000100365.10	ENSG00000100368.9
	ENSG00000103064.9	ENSG00000103160.7	ENSG00000103502.9	ENSG00000104341.12
	ENSG00000104549.7	ENSG00000104951.11	ENSG00000105968.14	ENSG00000106261.12
	ENSG00000108671.5	ENSG00000108953.12	ENSG00000109066.9	ENSG00000112531.12
	ENSG00000113161.11	ENSG00000114656.6	ENSG00000115274.10	ENSG00000116752.5
	ENSG00000120686.7	ENSG00000121848.9	ENSG00000122257.14	ENSG00000123064.8
	ENSG00000124006.10	ENSG00000126777.13	ENSG00000129993.10	ENSG00000130958.7
	ENSG00000133048.8	ENSG00000133114.13	ENSG00000133943.16	ENSG00000134571.6
	ENSG00000135365.11	ENSG00000135387.15	ENSG00000135679.17	ENSG00000136943.6
	ENSG00000137497.13	ENSG00000138785.10	ENSG00000139445.13	ENSG00000139687.9
	ENSG00000140280.9	ENSG00000141741.7	ENSG00000142627.9	ENSG00000142731.6
	ENSG00000143740.10	ENSG00000149634.4	ENSG00000150337.9	ENSG00000151014.4
	ENSG00000151491.8	ENSG00000153666.5	ENSG00000153993.9	ENSG00000154122.8
	ENSG00000157227.8	ENSG00000159189.7	ENSG00000161634.7	ENSG00000162434.7

	ENSG00000163347.5	ENSG00000163449.6	ENSG00000164074.10	ENSG00000165417.7
	ENSG00000166340.10	ENSG00000166557.8	ENSG00000166704.7	ENSG00000167196.9
	ENSG00000167210.12	ENSG00000167476.6	ENSG00000168658.14	ENSG00000169031.14
	ENSG00000169964.5	ENSG00000170037.9	ENSG00000170185.5	ENSG00000170899.6
	ENSG00000173511.5	ENSG00000174799.6	ENSG00000175029.12	ENSG00000176956.8
	ENSG00000177888.7	ENSG00000177954.7	ENSG00000178694.5	ENSG00000180178.6
	ENSG00000180801.11	ENSG00000181982.13	ENSG00000182050.9	ENSG00000183684.6
	ENSG00000184451.5	ENSG00000184786.4	ENSG00000185532.10	ENSG00000188404.4
	ENSG00000196754.6	ENSG00000198746.8	ENSG00000203943.4	ENSG00000204767.3
	ENSG00000213225.6	ENSG00000213593.5	ENSG00000213777.5	ENSG00000213930.7
	ENSG00000214146.2	ENSG00000214465.3	ENSG00000215187.5	ENSG00000215481.4
	ENSG00000218016.2	ENSG00000224870.3	ENSG00000225872.2	ENSG00000228397.1
	ENSG00000230850.3	ENSG00000232833.4	ENSG00000233762.2	ENSG00000236136.1
	ENSG00000237672.1	ENSG00000240682.5	ENSG00000240747.3	ENSG00000249565.2
	ENSG00000249679.1	ENSG00000251229.1	ENSG00000253304.1	ENSG00000255561.2
	ENSG00000255595.1	ENSG00000257464.1	ENSG00000258512.1	ENSG00000258811.1
	ENSG00000259120.2	ENSG00000261005.1	ENSG00000266651.1	ENSG00000267018.1
	ENSG00000267161.1	ENSG00000267416.1	ENSG00000268518.1	ENSG00000271856.1
	ENSG00000272002.1	ENSG00000272024.1	ENSG00000272377.1	ENSG00000272564.1
	ENSG00000272848.1	ENSG00000272942.1		
**Adrenal Gland**	ENSG00000047249.12	ENSG00000051128.14	ENSG00000072135.8	ENSG00000072201.9
	ENSG00000074696.8	ENSG00000087470.13	ENSG00000100281.9	ENSG00000100365.10
	ENSG00000100368.9	ENSG00000100938.13	ENSG00000104341.12	ENSG00000104722.9
	ENSG00000105707.9	ENSG00000109066.9	ENSG00000110011.9	ENSG00000113161.11
	ENSG00000118894.10	ENSG00000126107.10	ENSG00000131386.13	ENSG00000133048.8
	ENSG00000133114.13	ENSG00000134982.12	ENSG00000135775.9	ENSG00000144868.9
	ENSG00000147689.12	ENSG00000152128.13	ENSG00000155729.8	ENSG00000155755.14
	ENSG00000159189.7	ENSG00000159674.7	ENSG00000160602.9	ENSG00000162065.7
	ENSG00000162409.6	ENSG00000163449.6	ENSG00000164506.10	ENSG00000164744.8
	ENSG00000165417.7	ENSG00000165487.9	ENSG00000166091.15	ENSG00000166111.5
	ENSG00000166233.8	ENSG00000166326.5	ENSG00000166340.10	ENSG00000167207.7
	ENSG00000167862.5	ENSG00000169126.11	ENSG00000169379.11	ENSG00000170921.10
	ENSG00000172366.15	ENSG00000173404.3	ENSG00000174799.6	ENSG00000177888.7
	ENSG00000179935.5	ENSG00000179988.9	ENSG00000180178.6	ENSG00000181982.13
	ENSG00000182108.5	ENSG00000183654.8	ENSG00000185641.5	ENSG00000188100.8
	ENSG00000188827.6	ENSG00000197409.6	ENSG00000203780.6	ENSG00000205740.1
	ENSG00000213225.6	ENSG00000213355.3	ENSG00000214146.2	ENSG00000218016.2
	ENSG00000223922.1	ENSG00000224914.2	ENSG00000225370.1	ENSG00000227063.4
	ENSG00000228397.1	ENSG00000229180.4	ENSG00000229291.1	ENSG00000230850.3
	ENSG00000232833.4	ENSG00000233122.1	ENSG00000233996.1	ENSG00000236155.2
	ENSG00000240747.3	ENSG00000253390.1	ENSG00000255277.2	ENSG00000256340.4
	ENSG00000259404.1	ENSG00000264057.1	ENSG00000269028.2	ENSG00000270917.1
	ENSG00000272848.1			
**Artery Aorta**	ENSG00000004975.7	ENSG00000005189.15	ENSG00000006704.6	ENSG00000006712.10
	ENSG00000010282.10	ENSG00000023734.6	ENSG00000047249.12	ENSG00000050426.11
	ENSG00000056487.11	ENSG00000061794.8	ENSG00000072201.9	ENSG00000074696.8
	ENSG00000081052.10	ENSG00000085274.11	ENSG00000085741.8	ENSG00000090905.13
	ENSG00000097033.10	ENSG00000100246.8	ENSG00000100365.10	ENSG00000100567.8
	ENSG00000100918.8	ENSG00000103194.11	ENSG00000104131.8	ENSG00000104341.12

	ENSG00000104853.11	ENSG00000106483.7	ENSG00000108671.5	ENSG00000109066.9
	ENSG00000111339.6	ENSG00000113161.11	ENSG00000113209.6	ENSG00000114805.12
	ENSG00000115594.7	ENSG00000115998.3	ENSG00000117143.9	ENSG00000118518.11
	ENSG00000118894.10	ENSG00000119514.5	ENSG00000121848.9	ENSG00000126001.11
	ENSG00000126453.5	ENSG00000129566.8	ENSG00000130222.6	ENSG00000130590.9
	ENSG00000130844.12	ENSG00000132854.14	ENSG00000133114.13	ENSG00000133943.16
	ENSG00000134571.6	ENSG00000135775.9	ENSG00000135945.5	ENSG00000137941.12
	ENSG00000138778.7	ENSG00000138785.10	ENSG00000140280.9	ENSG00000142910.11
	ENSG00000143158.6	ENSG00000143196.4	ENSG00000143740.10	ENSG00000144560.9
	ENSG00000144642.16	ENSG00000144868.9	ENSG00000148053.11	ENSG00000148090.7
	ENSG00000148288.7	ENSG00000149212.6	ENSG00000151014.4	ENSG00000151079.6
	ENSG00000155282.7	ENSG00000155666.7	ENSG00000157657.10	ENSG00000158864.8
	ENSG00000159189.7	ENSG00000159307.14	ENSG00000160345.8	ENSG00000161149.7
	ENSG00000162227.3	ENSG00000162385.6	ENSG00000162434.7	ENSG00000162598.9
	ENSG00000162643.8	ENSG00000163793.8	ENSG00000166192.10	ENSG00000166340.10
	ENSG00000167112.7	ENSG00000167261.9	ENSG00000167941.2	ENSG00000167945.1
	ENSG00000168658.14	ENSG00000168883.15	ENSG00000169129.10	ENSG00000169989.2
	ENSG00000170921.10	ENSG00000171033.8	ENSG00000171443.6	ENSG00000171793.9
	ENSG00000172493.16	ENSG00000173281.4	ENSG00000173511.5	ENSG00000173988.8
	ENSG00000174799.6	ENSG00000175664.5	ENSG00000176095.7	ENSG00000176444.14
	ENSG00000176956.8	ENSG00000177599.8	ENSG00000180178.6	ENSG00000180801.11
	ENSG00000180891.8	ENSG00000181982.13	ENSG00000182175.9	ENSG00000183576.8
	ENSG00000184009.5	ENSG00000184471.6	ENSG00000186777.7	ENSG00000187013.2
	ENSG00000187566.3	ENSG00000196387.5	ENSG00000196453.7	ENSG00000197409.6
	ENSG00000198842.5	ENSG00000204396.6	ENSG00000205209.3	ENSG00000213079.5
	ENSG00000213225.6	ENSG00000213934.5	ENSG00000214510.5	ENSG00000215208.3
	ENSG00000220323.3	ENSG00000221955.6	ENSG00000224414.1	ENSG00000224914.2
	ENSG00000225708.2	ENSG00000227017.1	ENSG00000227063.4	ENSG00000227946.1
	ENSG00000228397.1	ENSG00000230850.3	ENSG00000231485.1	ENSG00000234509.1
	ENSG00000236136.1	ENSG00000236423.1	ENSG00000236675.1	ENSG00000237672.1
	ENSG00000248334.2	ENSG00000249306.1	ENSG00000251504.1	ENSG00000254618.1
	ENSG00000259158.1	ENSG00000259404.1	ENSG00000259612.1	ENSG00000260490.2
	ENSG00000260971.3	ENSG00000261090.1	ENSG00000264350.1	ENSG00000267416.1
	ENSG00000269543.1	ENSG00000270947.1	ENSG00000272144.1	ENSG00000272235.1
	ENSG00000272347.1			
**Artery Coronary**	ENSG00000004975.7	ENSG00000005700.10	ENSG00000024526.12	ENSG00000047249.12
	ENSG00000065328.12	ENSG00000077254.10	ENSG00000081248.6	ENSG00000103160.7
	ENSG00000105707.9	ENSG00000105968.14	ENSG00000114062.13	ENSG00000118985.10
	ENSG00000120318.11	ENSG00000135312.4	ENSG00000143222.7	ENSG00000145107.11
	ENSG00000148926.5	ENSG00000149212.6	ENSG00000153666.5	ENSG00000154479.8
	ENSG00000155282.7	ENSG00000155666.7	ENSG00000159189.7	ENSG00000162434.7
	ENSG00000162598.9	ENSG00000163449.6	ENSG00000163563.7	ENSG00000165417.7
	ENSG00000168621.10	ENSG00000168658.14	ENSG00000169964.5	ENSG00000170185.5
	ENSG00000171033.8	ENSG00000172425.6	ENSG00000173175.10	ENSG00000174748.14
	ENSG00000174799.6	ENSG00000176055.9	ENSG00000180611.6	ENSG00000183137.10
	ENSG00000184471.6	ENSG00000184557.3	ENSG00000187912.7	ENSG00000196754.6
	ENSG00000197409.6	ENSG00000198134.2	ENSG00000198842.5	ENSG00000213079.5
	ENSG00000213225.6	ENSG00000215481.4	ENSG00000215835.2	ENSG00000230850.3
	ENSG00000232810.3	ENSG00000233347.1	ENSG00000233775.1	ENSG00000236136.1

	ENSG00000242791.2	ENSG00000243627.4	ENSG00000250635.1	ENSG00000255561.2
	ENSG00000256582.1	ENSG00000257231.1	ENSG00000269543.1	
**Artery Tibial**	ENSG00000004975.7	ENSG00000005448.12	ENSG00000006704.6	ENSG00000007384.11
	ENSG00000010017.9	ENSG00000010282.10	ENSG00000010319.2	ENSG00000011028.9
	ENSG00000047249.12	ENSG00000048028.7	ENSG00000055332.12	ENSG00000061794.8
	ENSG00000065328.12	ENSG00000068305.13	ENSG00000068383.14	ENSG00000072135.8
	ENSG00000072201.9	ENSG00000073737.12	ENSG00000074696.8	ENSG00000076984.13
	ENSG00000081248.6	ENSG00000083635.7	ENSG00000083838.11	ENSG00000086991.8
	ENSG00000088367.16	ENSG00000088766.7	ENSG00000091436.12	ENSG00000099139.9
	ENSG00000100281.9	ENSG00000100365.10	ENSG00000101144.8	ENSG00000101624.6
	ENSG00000101670.7	ENSG00000103064.9	ENSG00000103160.7	ENSG00000103197.12
	ENSG00000104129.5	ENSG00000104369.4	ENSG00000104529.13	ENSG00000105792.15
	ENSG00000105851.6	ENSG00000105968.14	ENSG00000105997.18	ENSG00000106261.12
	ENSG00000106608.12	ENSG00000108641.10	ENSG00000109066.9	ENSG00000111011.13
	ENSG00000111012.5	ENSG00000112183.10	ENSG00000113209.6	ENSG00000113758.9
	ENSG00000114737.11	ENSG00000114738.6	ENSG00000114812.8	ENSG00000114841.13
	ENSG00000115274.10	ENSG00000116652.5	ENSG00000117632.16	ENSG00000118518.11
	ENSG00000118894.10	ENSG00000119574.8	ENSG00000120093.7	ENSG00000120318.11
	ENSG00000120686.7	ENSG00000121848.9	ENSG00000125249.6	ENSG00000126653.11
	ENSG00000128918.10	ENSG00000129993.10	ENSG00000131408.9	ENSG00000132405.14
	ENSG00000132958.13	ENSG00000133114.13	ENSG00000133943.16	ENSG00000134571.6
	ENSG00000134812.3	ENSG00000135312.4	ENSG00000135775.9	ENSG00000135945.5
	ENSG00000136160.10	ENSG00000136267.9	ENSG00000136925.10	ENSG00000137075.13
	ENSG00000138160.4	ENSG00000138231.8	ENSG00000138653.5	ENSG00000138709.13
	ENSG00000139547.6	ENSG00000139684.9	ENSG00000140254.8	ENSG00000140280.9
	ENSG00000140395.4	ENSG00000141027.16	ENSG00000143157.7	ENSG00000143314.8
	ENSG00000143740.10	ENSG00000144395.13	ENSG00000144468.12	ENSG00000144868.9
	ENSG00000145088.4	ENSG00000147573.12	ENSG00000149212.6	ENSG00000149634.4
	ENSG00000151014.4	ENSG00000152128.13	ENSG00000153094.17	ENSG00000154122.8
	ENSG00000154316.10	ENSG00000154654.10	ENSG00000155189.7	ENSG00000155282.7
	ENSG00000155666.7	ENSG00000158055.11	ENSG00000159189.7	ENSG00000159307.14
	ENSG00000159860.5	ENSG00000160013.4	ENSG00000160049.7	ENSG00000160602.9
	ENSG00000161642.13	ENSG00000162928.8	ENSG00000163145.8	ENSG00000163449.6
	ENSG00000163950.8	ENSG00000164054.11	ENSG00000164405.6	ENSG00000164620.4
	ENSG00000164742.10	ENSG00000164919.6	ENSG00000165417.7	ENSG00000166091.15
	ENSG00000166340.10	ENSG00000166342.14	ENSG00000167281.14	ENSG00000167862.5
	ENSG00000167941.2	ENSG00000167985.2	ENSG00000168172.4	ENSG00000168398.5
	ENSG00000168621.10	ENSG00000168658.14	ENSG00000168772.9	ENSG00000169031.14
	ENSG00000169129.10	ENSG00000169174.9	ENSG00000169403.7	ENSG00000170855.3
	ENSG00000170989.8	ENSG00000171016.7	ENSG00000171467.11	ENSG00000172243.13
	ENSG00000173281.4	ENSG00000173801.12	ENSG00000174576.4	ENSG00000174748.14
	ENSG00000174799.6	ENSG00000174917.4	ENSG00000176055.9	ENSG00000176105.9
	ENSG00000176153.10	ENSG00000176371.9	ENSG00000177192.9	ENSG00000177728.10
	ENSG00000177888.7	ENSG00000178031.11	ENSG00000178789.4	ENSG00000178826.6
	ENSG00000179387.5	ENSG00000179988.9	ENSG00000180178.6	ENSG00000180210.10
	ENSG00000180801.11	ENSG00000180828.1	ENSG00000181744.4	ENSG00000181982.13
	ENSG00000182108.5	ENSG00000182175.9	ENSG00000182183.10	ENSG00000182831.7
	ENSG00000183576.8	ENSG00000183801.3	ENSG00000184009.5	ENSG00000184471.6
	ENSG00000184557.3	ENSG00000184719.7	ENSG00000184752.8	ENSG00000185028.3

	ENSG00000185087.7	ENSG00000185532.10	ENSG00000186188.6	ENSG00000186940.6
	ENSG00000187498.10	ENSG00000187566.3	ENSG00000187778.9	ENSG00000187984.8
	ENSG00000188242.4	ENSG00000188641.8	ENSG00000189058.4	ENSG00000196182.6
	ENSG00000196453.7	ENSG00000196505.6	ENSG00000196581.6	ENSG00000196639.6
	ENSG00000196754.6	ENSG00000197409.6	ENSG00000197859.5	ENSG00000197956.5
	ENSG00000198003.7	ENSG00000198081.6	ENSG00000198553.4	ENSG00000198668.6
	ENSG00000204390.8	ENSG00000204767.3	ENSG00000205209.3	ENSG00000205302.2
	ENSG00000205629.7	ENSG00000206418.3	ENSG00000213079.5	ENSG00000213197.3
	ENSG00000213225.6	ENSG00000213492.2	ENSG00000214413.3	ENSG00000214510.5
	ENSG00000214688.4	ENSG00000224914.2	ENSG00000224956.5	ENSG00000225137.1
	ENSG00000225726.1	ENSG00000226086.5	ENSG00000227477.1	ENSG00000228008.1
	ENSG00000228339.1	ENSG00000228397.1	ENSG00000228797.2	ENSG00000229291.1
	ENSG00000230850.3	ENSG00000231169.3	ENSG00000231322.1	ENSG00000231485.1
	ENSG00000232833.4	ENSG00000233610.1	ENSG00000234065.2	ENSG00000234536.1
	ENSG00000236136.1	ENSG00000236184.1	ENSG00000236976.1	ENSG00000236998.2
	ENSG00000237441.5	ENSG00000240747.3	ENSG00000241634.1	ENSG00000245330.4
	ENSG00000245651.2	ENSG00000248632.1	ENSG00000248916.1	ENSG00000251139.1
	ENSG00000253304.1	ENSG00000253972.1	ENSG00000254618.1	ENSG00000256361.1
	ENSG00000257859.1	ENSG00000258418.1	ENSG00000259158.1	ENSG00000259404.1
	ENSG00000259758.1	ENSG00000260139.2	ENSG00000260619.1	ENSG00000261087.1
	ENSG00000261889.1	ENSG00000263105.1	ENSG00000263781.2	ENSG00000264016.2
	ENSG00000264148.1	ENSG00000266651.1	ENSG00000267416.1	ENSG00000267957.1
	ENSG00000269282.1	ENSG00000269510.1	ENSG00000269543.1	ENSG00000270326.1
	ENSG00000270457.1	ENSG00000270917.1	ENSG00000272235.1	ENSG00000272347.1
	ENSG00000272425.1	ENSG00000272966.1	ENSG00000273399.1	
**Brain Amygdala**	ENSG00000010282.10	ENSG00000047249.12	ENSG00000075223.9	ENSG00000092531.5
	ENSG00000126368.5	ENSG00000128228.4	ENSG00000128585.13	ENSG00000133114.13
	ENSG00000133606.6	ENSG00000135722.4	ENSG00000138709.13	ENSG00000143127.8
	ENSG00000159189.7	ENSG00000162949.12	ENSG00000164744.8	ENSG00000165066.11
	ENSG00000165417.7	ENSG00000167771.5	ENSG00000167862.5	ENSG00000169126.11
	ENSG00000170525.14	ENSG00000171790.11	ENSG00000187498.10	ENSG00000188674.6
	ENSG00000196754.6	ENSG00000213225.6	ENSG00000213355.3	ENSG00000214146.2
	ENSG00000227063.4	ENSG00000230850.3	ENSG00000234589.3	ENSG00000240751.1
	ENSG00000240907.1	ENSG00000250635.1	ENSG00000258418.1	ENSG00000261787.1
	ENSG00000262098.1	ENSG00000267319.1	ENSG00000271914.1	ENSG00000271927.1
	ENSG00000272428.1			
**Brain Anterior cingulate**	ENSG00000047249.12	ENSG00000051128.14	ENSG00000058668.10	ENSG00000062725.5
	ENSG00000064692.14	ENSG00000075223.9	ENSG00000076513.12	ENSG00000083635.7
	ENSG00000086065.9	ENSG00000089159.11	ENSG00000096080.7	ENSG00000100276.9
	ENSG00000100365.10	ENSG00000100938.13	ENSG00000101444.8	ENSG00000101898.5
	ENSG00000102738.6	ENSG00000104722.9	ENSG00000104853.11	ENSG00000111729.8
	ENSG00000114019.10	ENSG00000118292.4	ENSG00000118733.12	ENSG00000118985.10
	ENSG00000120913.19	ENSG00000123342.11	ENSG00000130717.8	ENSG00000132424.10
	ENSG00000133114.13	ENSG00000135378.3	ENSG00000136160.10	ENSG00000136297.10
	ENSG00000136536.10	ENSG00000139687.9	ENSG00000152977.5	ENSG00000154080.8
	ENSG00000155093.13	ENSG00000155666.7	ENSG00000156026.10	ENSG00000158864.8
	ENSG00000159189.7	ENSG00000162928.8	ENSG00000165417.7	ENSG00000166831.4
	ENSG00000167483.13	ENSG00000168658.14	ENSG00000169918.5	ENSG00000180178.6
	ENSG00000180801.11	ENSG00000182175.9	ENSG00000183260.5	ENSG00000183273.2

	ENSG00000183340.5	ENSG00000183814.11	ENSG00000184307.9	ENSG00000185482.3
	ENSG00000187498.10	ENSG00000196923.9	ENSG00000197471.7	ENSG00000203260.2
	ENSG00000204793.4	ENSG00000215187.5	ENSG00000218016.2	ENSG00000220323.3
	ENSG00000226567.1	ENSG00000227077.2	ENSG00000227088.1	ENSG00000230850.3
	ENSG00000231720.1	ENSG00000232626.3	ENSG00000232833.4	ENSG00000236136.1
	ENSG00000237094.7	ENSG00000239815.1	ENSG00000240747.3	ENSG00000241416.1
	ENSG00000250635.1	ENSG00000254714.1	ENSG00000258418.1	ENSG00000259404.1
	ENSG00000261609.3	ENSG00000267289.1	ENSG00000267416.1	ENSG00000269028.2
	ENSG00000269781.1	ENSG00000272567.1	ENSG00000272735.1	ENSG00000272942.1
**Brain Caudate basal ganglia**	ENSG00000010282.10	ENSG00000047249.12	ENSG00000048405.5	ENSG00000051128.14
	ENSG00000074696.8	ENSG00000086065.9	ENSG00000089159.11	ENSG00000092531.5
	ENSG00000104131.8	ENSG00000105610.4	ENSG00000106261.12	ENSG00000109066.9
	ENSG00000109586.7	ENSG00000111275.8	ENSG00000112855.10	ENSG00000113161.11
	ENSG00000113209.6	ENSG00000115267.5	ENSG00000116205.6	ENSG00000118985.10
	ENSG00000120913.19	ENSG00000124664.6	ENSG00000128309.12	ENSG00000131386.13
	ENSG00000133392.12	ENSG00000133606.6	ENSG00000133639.3	ENSG00000133742.9
	ENSG00000134352.15	ENSG00000135898.5	ENSG00000138160.4	ENSG00000140280.9
	ENSG00000144028.10	ENSG00000145287.6	ENSG00000147533.12	ENSG00000147573.12
	ENSG00000149634.4	ENSG00000153993.9	ENSG00000155666.7	ENSG00000158055.11
	ENSG00000159189.7	ENSG00000162419.8	ENSG00000162441.7	ENSG00000163206.5
	ENSG00000164744.8	ENSG00000165417.7	ENSG00000165672.5	ENSG00000167862.5
	ENSG00000168658.14	ENSG00000169926.5	ENSG00000169964.5	ENSG00000170525.14
	ENSG00000173250.2	ENSG00000174799.6	ENSG00000178741.7	ENSG00000178764.6
	ENSG00000179241.8	ENSG00000180801.11	ENSG00000181227.2	ENSG00000181982.13
	ENSG00000182175.9	ENSG00000183576.8	ENSG00000183814.11	ENSG00000187498.10
	ENSG00000187566.3	ENSG00000188404.4	ENSG00000188674.6	ENSG00000196581.6
	ENSG00000197702.7	ENSG00000213078.3	ENSG00000213111.4	ENSG00000213212.3
	ENSG00000213355.3	ENSG00000215187.5	ENSG00000218016.2	ENSG00000224914.2
	ENSG00000226567.1	ENSG00000228566.1	ENSG00000228970.5	ENSG00000230850.3
	ENSG00000231720.1	ENSG00000233487.6	ENSG00000234104.1	ENSG00000234769.4
	ENSG00000236136.1	ENSG00000236155.2	ENSG00000237094.7	ENSG00000240747.3
	ENSG00000242375.1	ENSG00000247134.2	ENSG00000248243.1	ENSG00000249129.1
	ENSG00000249889.1	ENSG00000250635.1	ENSG00000254714.1	ENSG00000257398.2
	ENSG00000257464.1	ENSG00000258418.1	ENSG00000259584.1	ENSG00000261052.1
	ENSG00000261787.1	ENSG00000267416.1	ENSG00000269907.1	ENSG00000271156.1
	ENSG00000272002.1	ENSG00000272144.1	ENSG00000272520.1	ENSG00000272583.1
	ENSG00000272795.1	ENSG00000272942.1	ENSG00000273181.1	
**Brain Cerebellar**	ENSG00000006704.6	ENSG00000047249.12	ENSG00000051128.14	ENSG00000058668.10
	ENSG00000062725.5	ENSG00000063322.9	ENSG00000064692.14	ENSG00000074696.8
	ENSG00000075223.9	ENSG00000081237.14	ENSG00000089159.11	ENSG00000092871.12
	ENSG00000099957.12	ENSG00000100519.7	ENSG00000100938.13	ENSG00000101444.8
	ENSG00000104722.9	ENSG00000105647.10	ENSG00000106261.12	ENSG00000108773.6
	ENSG00000109066.9	ENSG00000112144.11	ENSG00000114019.10	ENSG00000116544.7
	ENSG00000117143.9	ENSG00000117632.16	ENSG00000118733.12	ENSG00000119714.6
	ENSG00000120699.8	ENSG00000123342.11	ENSG00000124194.11	ENSG00000125304.8
	ENSG00000127824.9	ENSG00000129159.6	ENSG00000131386.13	ENSG00000132424.10
	ENSG00000132744.3	ENSG00000133048.8	ENSG00000133114.13	ENSG00000135378.3
	ENSG00000143995.15	ENSG00000148288.7	ENSG00000152128.13	ENSG00000155666.7
	ENSG00000157851.12	ENSG00000159189.7	ENSG00000159674.7	ENSG00000160218.8

	ENSG00000160602.9	ENSG00000163563.7	ENSG00000164109.9	ENSG00000164744.8
	ENSG00000164749.7	ENSG00000164897.8	ENSG00000165181.12	ENSG00000165417.7
	ENSG00000166340.10	ENSG00000167549.14	ENSG00000167862.5	ENSG00000168477.13
	ENSG00000168658.14	ENSG00000171777.11	ENSG00000172425.6	ENSG00000173175.10
	ENSG00000174799.6	ENSG00000177888.7	ENSG00000180801.11	ENSG00000182108.5
	ENSG00000183010.12	ENSG00000183576.8	ENSG00000183814.11	ENSG00000187498.10
	ENSG00000188827.6	ENSG00000196628.9	ENSG00000196666.3	ENSG00000197386.6
	ENSG00000198821.6	ENSG00000204231.6	ENSG00000204793.4	ENSG00000213225.6
	ENSG00000213355.3	ENSG00000215187.5	ENSG00000215481.4	ENSG00000217289.3
	ENSG00000218016.2	ENSG00000226055.2	ENSG00000226979.4	ENSG00000227331.1
	ENSG00000228397.1	ENSG00000229184.2	ENSG00000230850.3	ENSG00000231485.1
	ENSG00000231720.1	ENSG00000232626.3	ENSG00000233469.1	ENSG00000233762.2
	ENSG00000234690.2	ENSG00000235319.1	ENSG00000235957.1	ENSG00000236136.1
	ENSG00000240747.3	ENSG00000242445.1	ENSG00000248334.2	ENSG00000248487.4
	ENSG00000249487.2	ENSG00000249565.2	ENSG00000250392.2	ENSG00000250635.1
	ENSG00000251189.1	ENSG00000251410.1	ENSG00000254100.1	ENSG00000254990.1
	ENSG00000255735.1	ENSG00000257464.1	ENSG00000258418.1	ENSG00000259435.2
	ENSG00000261787.1	ENSG00000266651.1	ENSG00000267416.1	ENSG00000267665.1
	ENSG00000269028.2	ENSG00000269866.1	ENSG00000270098.1	ENSG00000270270.1
	ENSG00000270917.1	ENSG00000271200.1	ENSG00000271737.1	ENSG00000272002.1
	ENSG00000272144.1	ENSG00000272347.1	ENSG00000272520.1	ENSG00000272942.1
	ENSG00000272988.1	ENSG00000273325.1	ENSG00000273365.1	
**Brain Cerebellum**	ENSG00000004660.10	ENSG00000004866.14	ENSG00000005700.10	ENSG00000005884.13
	ENSG00000006704.6	ENSG00000007545.11	ENSG00000023734.6	ENSG00000047249.12
	ENSG00000051128.14	ENSG00000053328.8	ENSG00000053702.10	ENSG00000058056.4
	ENSG00000058668.10	ENSG00000064692.14	ENSG00000072201.9	ENSG00000074696.8
	ENSG00000075223.9	ENSG00000076513.12	ENSG00000077522.8	ENSG00000085741.8
	ENSG00000089159.11	ENSG00000092871.12	ENSG00000100276.9	ENSG00000100281.9
	ENSG00000100410.3	ENSG00000101605.8	ENSG00000104131.8	ENSG00000104205.8
	ENSG00000105467.4	ENSG00000105647.10	ENSG00000105792.15	ENSG00000106682.10
	ENSG00000108773.6	ENSG00000109066.9	ENSG00000109586.7	ENSG00000111218.7
	ENSG00000111275.8	ENSG00000112769.14	ENSG00000112981.3	ENSG00000115274.10
	ENSG00000115286.15	ENSG00000116670.10	ENSG00000117143.9	ENSG00000118518.11
	ENSG00000118985.10	ENSG00000119514.5	ENSG00000119636.11	ENSG00000119685.15
	ENSG00000123064.8	ENSG00000124194.11	ENSG00000125304.8	ENSG00000131386.13
	ENSG00000132424.10	ENSG00000133048.8	ENSG00000133114.13	ENSG00000133193.8
	ENSG00000133639.3	ENSG00000133943.16	ENSG00000134571.6	ENSG00000134853.7
	ENSG00000135378.3	ENSG00000135722.4	ENSG00000135898.5	ENSG00000136925.10
	ENSG00000137941.12	ENSG00000138615.4	ENSG00000138785.10	ENSG00000139350.7
	ENSG00000139546.6	ENSG00000140280.9	ENSG00000140740.6	ENSG00000143158.6
	ENSG00000143179.8	ENSG00000143340.6	ENSG00000143556.4	ENSG00000143740.10
	ENSG00000144285.11	ENSG00000144395.13	ENSG00000144868.9	ENSG00000145088.4
	ENSG00000149634.4	ENSG00000152620.8	ENSG00000155666.7	ENSG00000157388.9
	ENSG00000159189.7	ENSG00000159674.7	ENSG00000160602.9	ENSG00000160741.12
	ENSG00000160908.14	ENSG00000162434.7	ENSG00000162928.8	ENSG00000163046.11
	ENSG00000163206.5	ENSG00000163486.8	ENSG00000163563.7	ENSG00000164744.8
	ENSG00000164749.7	ENSG00000164816.6	ENSG00000165417.7	ENSG00000165644.6
	ENSG00000166091.15	ENSG00000166340.10	ENSG00000166575.12	ENSG00000166704.7
	ENSG00000167550.6	ENSG00000167771.5	ENSG00000167862.5	ENSG00000168477.13

	ENSG00000168658.14	ENSG00000170540.10	ENSG00000170921.10	ENSG00000171777.11
	ENSG00000172725.9	ENSG00000174799.6	ENSG00000177888.7	ENSG00000180178.6
	ENSG00000180210.10	ENSG00000180801.11	ENSG00000181227.2	ENSG00000181982.13
	ENSG00000183137.10	ENSG00000183260.5	ENSG00000183273.2	ENSG00000183576.8
	ENSG00000183921.5	ENSG00000184009.5	ENSG00000185250.11	ENSG00000185955.4
	ENSG00000187187.9	ENSG00000187288.6	ENSG00000187498.10	ENSG00000187889.8
	ENSG00000187984.8	ENSG00000188827.6	ENSG00000196116.6	ENSG00000196581.6
	ENSG00000196628.9	ENSG00000196754.6	ENSG00000197386.6	ENSG00000197409.6
	ENSG00000197471.7	ENSG00000197575.5	ENSG00000198821.6	ENSG00000213225.6
	ENSG00000213355.3	ENSG00000213859.3	ENSG00000215187.5	ENSG00000217289.3
	ENSG00000218016.2	ENSG00000220008.2	ENSG00000225526.4	ENSG00000226055.2
	ENSG00000226852.2	ENSG00000227745.1	ENSG00000228127.1	ENSG00000228223.1
	ENSG00000228397.1	ENSG00000229184.2	ENSG00000230850.3	ENSG00000231485.1
	ENSG00000231720.1	ENSG00000232626.3	ENSG00000232833.4	ENSG00000233087.4
	ENSG00000233469.1	ENSG00000234690.2	ENSG00000236136.1	ENSG00000236814.1
	ENSG00000237094.7	ENSG00000237289.5	ENSG00000240747.3	ENSG00000242299.1
	ENSG00000248487.4	ENSG00000249471.3	ENSG00000249889.1	ENSG00000250392.2
	ENSG00000250635.1	ENSG00000254714.1	ENSG00000255624.1	ENSG00000255735.1
	ENSG00000257194.2	ENSG00000257464.1	ENSG00000257859.1	ENSG00000258418.1
	ENSG00000259404.1	ENSG00000259435.2	ENSG00000259502.1	ENSG00000260619.1
	ENSG00000261787.1	ENSG00000264281.2	ENSG00000267319.1	ENSG00000267416.1
	ENSG00000269097.1	ENSG00000269866.1	ENSG00000269873.1	ENSG00000270270.1
	ENSG00000270750.1	ENSG00000270755.1	ENSG00000271143.1	ENSG00000271275.1
	ENSG00000271737.1	ENSG00000272108.1	ENSG00000272201.1	ENSG00000272663.1
	ENSG00000272798.1	ENSG00000272942.1	ENSG00000272988.1	ENSG00000273325.1
	ENSG00000273365.1			
	ENSG00000007545.11	ENSG00000051128.14	ENSG00000053328.8	ENSG00000058668.10
	ENSG00000063322.9	ENSG00000074696.8	ENSG00000083635.7	ENSG00000089159.11
	ENSG00000100246.8	ENSG00000100938.13	ENSG00000103064.9	ENSG00000103175.6
	ENSG00000103522.11	ENSG00000104408.5	ENSG00000104722.9	ENSG00000107443.11
	ENSG00000107929.10	ENSG00000108773.6	ENSG00000109066.9	ENSG00000110066.10
	ENSG00000111845.4	ENSG00000113327.10	ENSG00000114019.10	ENSG00000115274.10
	ENSG00000118733.12	ENSG00000118985.10	ENSG00000119636.11	ENSG00000119703.12
	ENSG00000122574.6	ENSG00000126353.3	ENSG00000126368.5	ENSG00000131386.13
	ENSG00000132424.10	ENSG00000133048.8	ENSG00000133392.12	ENSG00000134508.8
**Brain Cortex**	ENSG00000134571.6	ENSG00000135823.9	ENSG00000135898.5	ENSG00000136943.6
	ENSG00000137941.12	ENSG00000138435.10	ENSG00000139684.9	ENSG00000143340.6
	ENSG00000148288.7	ENSG00000149634.4	ENSG00000151023.12	ENSG00000151790.4
	ENSG00000152556.11	ENSG00000153162.8	ENSG00000153666.5	ENSG00000154122.8
	ENSG00000154316.10	ENSG00000157851.12	ENSG00000159189.7	ENSG00000159674.7
	ENSG00000162434.7	ENSG00000162949.12	ENSG00000163563.7	ENSG00000164744.8
	ENSG00000165417.7	ENSG00000165527.5	ENSG00000166340.10	ENSG00000166840.9
	ENSG00000167862.5	ENSG00000167941.2	ENSG00000168398.5	ENSG00000168658.14
	ENSG00000168763.11	ENSG00000169964.5	ENSG00000170185.5	ENSG00000173404.3
	ENSG00000174744.9	ENSG00000174799.6	ENSG00000175029.12	ENSG00000176956.8
	ENSG00000178460.13	ENSG00000181982.13	ENSG00000182108.5	ENSG00000182324.5
	ENSG00000183010.12	ENSG00000183576.8	ENSG00000185269.7	ENSG00000188674.6
	ENSG00000196754.6	ENSG00000196776.10	ENSG00000198668.6	ENSG00000204261.4
	ENSG00000204793.4	ENSG00000205871.4	ENSG00000205882.4	ENSG00000213225.6

	ENSG00000213355.3	ENSG00000213380.9	ENSG00000214708.4	ENSG00000218016.2
	ENSG00000224914.2	ENSG00000226580.1	ENSG00000227869.1	ENSG00000228127.1
	ENSG00000228817.3	ENSG00000230850.3	ENSG00000232626.3	ENSG00000232833.4
	ENSG00000233469.1	ENSG00000235478.1	ENSG00000236136.1	ENSG00000237094.7
	ENSG00000237166.1	ENSG00000240393.1	ENSG00000240907.1	ENSG00000244411.2
	ENSG00000247134.2	ENSG00000248334.2	ENSG00000250635.1	ENSG00000254100.1
	ENSG00000254714.1	ENSG00000257194.2	ENSG00000257464.1	ENSG00000258418.1
	ENSG00000261787.1	ENSG00000262098.1	ENSG00000264281.2	ENSG00000265369.2
	ENSG00000267010.1	ENSG00000267416.1	ENSG00000267723.1	ENSG00000270326.1
	ENSG00000270947.1	ENSG00000271275.1	ENSG00000272347.1	ENSG00000272377.1
	ENSG00000272663.1			
**Brain Frontal Cortex**	ENSG00000001630.11	ENSG00000038427.11	ENSG00000047249.12	ENSG00000074696.8
	ENSG00000089159.11	ENSG00000100312.6	ENSG00000100410.3	ENSG00000100938.13
	ENSG00000103064.9	ENSG00000104517.8	ENSG00000105085.6	ENSG00000108773.6
	ENSG00000109066.9	ENSG00000111252.6	ENSG00000111644.3	ENSG00000112983.13
	ENSG00000118523.5	ENSG00000118985.10	ENSG00000130635.11	ENSG00000132424.10
	ENSG00000133114.13	ENSG00000135898.5	ENSG00000138435.10	ENSG00000143340.6
	ENSG00000146670.5	ENSG00000147588.6	ENSG00000151790.4	ENSG00000153922.6
	ENSG00000154122.8	ENSG00000155666.7	ENSG00000159189.7	ENSG00000163209.10
	ENSG00000164744.8	ENSG00000165417.7	ENSG00000166091.15	ENSG00000166831.4
	ENSG00000167862.5	ENSG00000167941.2	ENSG00000168658.14	ENSG00000169031.14
	ENSG00000172830.8	ENSG00000174799.6	ENSG00000175857.4	ENSG00000181227.2
	ENSG00000181982.13	ENSG00000182183.10	ENSG00000183576.8	ENSG00000183647.6
	ENSG00000184261.4	ENSG00000196666.3	ENSG00000196754.6	ENSG00000197471.7
	ENSG00000205189.7	ENSG00000205871.4	ENSG00000213225.6	ENSG00000213355.3
	ENSG00000213608.5	ENSG00000218016.2	ENSG00000223945.2	ENSG00000224914.2
	ENSG00000226567.1	ENSG00000226580.1	ENSG00000227745.1	ENSG00000228541.1
	ENSG00000230850.3	ENSG00000231485.1	ENSG00000231720.1	ENSG00000232626.3
	ENSG00000232833.4	ENSG00000233347.1	ENSG00000233469.1	ENSG00000234589.3
	ENSG00000234797.4	ENSG00000235655.2	ENSG00000236136.1	ENSG00000236155.2
	ENSG00000237094.7	ENSG00000240747.3	ENSG00000242445.1	ENSG00000242791.2
	ENSG00000244411.2	ENSG00000250635.1	ENSG00000250723.1	ENSG00000254639.1
	ENSG00000254714.1	ENSG00000254990.1	ENSG00000256540.1	ENSG00000257464.1
	ENSG00000258418.1	ENSG00000258986.2	ENSG00000261787.1	ENSG00000264281.2
	ENSG00000267264.1	ENSG00000267416.1	ENSG00000269165.1	ENSG00000270244.1
	ENSG00000271396.1	ENSG00000272428.1	ENSG00000272828.1	ENSG00000272942.1
**Brain Hippocampus**	ENSG00000047249.12	ENSG00000058668.10	ENSG00000086991.8	ENSG00000089723.5
	ENSG00000099246.12	ENSG00000100938.13	ENSG00000101342.5	ENSG00000103522.11
	ENSG00000104332.7	ENSG00000108424.5	ENSG00000109066.9	ENSG00000112981.3
	ENSG00000133328.3	ENSG00000134571.6	ENSG00000145088.4	ENSG00000155666.7
	ENSG00000157851.12	ENSG00000159189.7	ENSG00000161634.7	ENSG00000162510.5
	ENSG00000165417.7	ENSG00000168398.5	ENSG00000170584.6	ENSG00000171560.10
	ENSG00000172725.9	ENSG00000180178.6	ENSG00000181982.13	ENSG00000183273.2
	ENSG00000183576.8	ENSG00000188674.6	ENSG00000196581.6	ENSG00000198824.4
	ENSG00000213225.6	ENSG00000213260.3	ENSG00000213593.5	ENSG00000214381.4
	ENSG00000226567.1	ENSG00000230850.3	ENSG00000231720.1	ENSG00000232040.2
	ENSG00000232626.3	ENSG00000241416.1	ENSG00000247134.2	ENSG00000249061.1
	ENSG00000250635.1	ENSG00000258418.1	ENSG00000261787.1	ENSG00000267416.1
	ENSG00000272002.1	ENSG00000272347.1	ENSG00000272885.1	

	ENSG00000039650.5	ENSG00000047249.12	ENSG00000058668.10	ENSG00000061656.5
	ENSG00000074696.8	ENSG00000087086.9	ENSG00000100351.12	ENSG00000101412.9
	ENSG00000108773.6	ENSG00000109066.9	ENSG00000111012.5	ENSG00000115966.12
	ENSG00000116863.10	ENSG00000120699.8	ENSG00000124194.11	ENSG00000125910.4
**Brain**	ENSG00000132424.10	ENSG00000143340.6	ENSG00000144224.12	ENSG00000149634.4
**Hypothalamus**	ENSG00000152620.8	ENSG00000155666.7	ENSG00000155714.9	ENSG00000159189.7
	ENSG00000161970.8	ENSG00000164744.8	ENSG00000165417.7	ENSG00000166340.10
	ENSG00000168172.4	ENSG00000169964.5	ENSG00000173175.10	ENSG00000176095.7
	ENSG00000180801.11	ENSG00000181982.13	ENSG00000183576.8	ENSG00000184261.4
	ENSG00000196923.9	ENSG00000198911.7	ENSG00000204231.6	ENSG00000213225.6
	ENSG00000213355.3	ENSG00000223922.1	ENSG00000224425.2	ENSG00000224566.2
	ENSG00000225614.2	ENSG00000226197.2	ENSG00000226852.2	ENSG00000227920.2
	ENSG00000228541.1	ENSG00000229291.1	ENSG00000229344.1	ENSG00000229611.1
	ENSG00000229789.1	ENSG00000230850.3	ENSG00000231684.2	ENSG00000232833.4
	ENSG00000234076.1	ENSG00000235213.1	ENSG00000236136.1	ENSG00000240747.3
	ENSG00000243449.2	ENSG00000244411.2	ENSG00000250635.1	ENSG00000251410.1
	ENSG00000258418.1	ENSG00000261787.1	ENSG00000267416.1	ENSG00000267570.1
	ENSG00000270270.1	ENSG00000271499.1	ENSG00000272942.1	
	ENSG00000023734.6	ENSG00000038427.11	ENSG00000039650.5	ENSG00000047249.12
	ENSG00000053702.10	ENSG00000058668.10	ENSG00000085741.8	ENSG00000089159.11
	ENSG00000100142.10	ENSG00000100938.13	ENSG00000105610.4	ENSG00000108773.6
	ENSG00000109066.9	ENSG00000111252.6	ENSG00000113161.11	ENSG00000115274.10
**Brain**	ENSG00000115966.12	ENSG00000119927.9	ENSG00000124194.11	ENSG00000132424.10
**Nucleus**	ENSG00000132744.3	ENSG00000149506.6	ENSG00000149634.4	ENSG00000152128.13
	ENSG00000152556.11	ENSG00000154479.8	ENSG00000159189.7	ENSG00000162889.6
	ENSG00000163206.5	ENSG00000163563.7	ENSG00000164744.8	ENSG00000165417.7
	ENSG00000166340.10	ENSG00000167862.5	ENSG00000168490.9	ENSG00000168658.14
	ENSG00000169499.10	ENSG00000169964.5	ENSG00000170144.14	ENSG00000170498.7
	ENSG00000173250.2	ENSG00000174799.6	ENSG00000176225.8	ENSG00000176533.8
	ENSG00000177954.7	ENSG00000179041.2	ENSG00000180210.10	ENSG00000180801.11
	ENSG00000181315.6	ENSG00000181982.13	ENSG00000182108.5	ENSG00000182631.5
	ENSG00000183273.2	ENSG00000188674.6	ENSG00000203260.2	ENSG00000205871.4
	ENSG00000213225.6	ENSG00000213355.3	ENSG00000213859.3	ENSG00000215187.5
	ENSG00000218016.2	ENSG00000224425.2	ENSG00000225178.4	ENSG00000226567.1
	ENSG00000226979.4	ENSG00000227745.1	ENSG00000228397.1	ENSG00000229007.1
	ENSG00000229184.2	ENSG00000230850.3	ENSG00000231720.1	ENSG00000232626.3
	ENSG00000232810.3	ENSG00000232815.1	ENSG00000233469.1	ENSG00000235478.1
	ENSG00000236155.2	ENSG00000237749.3	ENSG00000242791.2	ENSG00000248487.4
	ENSG00000249565.2	ENSG00000249715.5	ENSG00000249855.1	ENSG00000250635.1
	ENSG00000251410.1	ENSG00000254714.1	ENSG00000257464.1	ENSG00000258418.1
	ENSG00000259032.2	ENSG00000261787.1	ENSG00000266964.1	ENSG00000267416.1
	ENSG00000270098.1	ENSG00000271499.1	ENSG00000272428.1	ENSG00000272663.1
	ENSG00000272942.1			
	ENSG00000004660.10	ENSG00000005884.13	ENSG00000053702.10	ENSG00000058668.10
	ENSG00000085274.11	ENSG00000089159.11	ENSG00000100938.13	ENSG00000101146.8
	ENSG00000104722.9	ENSG00000108773.6	ENSG00000109787.8	ENSG00000115414.14
	ENSG00000131386.13	ENSG00000132424.10	ENSG00000132744.3	ENSG00000133302.8
**Brain**	ENSG00000133639.3	ENSG00000136943.6	ENSG00000145088.4	ENSG00000145451.8
**Putamen**	ENSG00000149634.4	ENSG00000155666.7	ENSG00000157851.12	ENSG00000159189.7

	ENSG00000163046.11	ENSG00000164744.8	ENSG00000165417.7	ENSG00000166340.10
	ENSG00000166575.12	ENSG00000169908.6	ENSG00000171307.14	ENSG00000173250.2
	ENSG00000173404.3	ENSG00000174799.6	ENSG00000176343.5	ENSG00000179546.3
	ENSG00000180178.6	ENSG00000181982.13	ENSG00000182324.5	ENSG00000183576.8
	ENSG00000184378.2	ENSG00000184451.5	ENSG00000184786.4	ENSG00000187498.10
	ENSG00000196754.6	ENSG00000197536.6	ENSG00000204793.4	ENSG00000213355.3
	ENSG00000224425.2	ENSG00000230850.3	ENSG00000232626.3	ENSG00000236136.1
	ENSG00000236155.2	ENSG00000240747.3	ENSG00000242791.2	ENSG00000250635.1
	ENSG00000251410.1	ENSG00000258418.1	ENSG00000261787.1	ENSG00000266651.1
	ENSG00000267416.1	ENSG00000272663.1		
**Brain Spinal cord**	ENSG00000006704.6	ENSG00000047249.12	ENSG00000051128.14	ENSG00000058668.10
	ENSG00000070495.10	ENSG00000087086.9	ENSG00000089159.11	ENSG00000100365.10
	ENSG00000100938.13	ENSG00000101412.9	ENSG00000103310.6	ENSG00000105254.7
	ENSG00000107295.8	ENSG00000109066.9	ENSG00000111012.5	ENSG00000116544.7
	ENSG00000118733.12	ENSG00000120318.11	ENSG00000122435.5	ENSG00000132424.10
	ENSG00000135801.5	ENSG00000154316.10	ENSG00000155666.7	ENSG00000159189.7
	ENSG00000163739.4	ENSG00000164074.10	ENSG00000164744.8	ENSG00000165417.7
	ENSG00000166340.10	ENSG00000167862.5	ENSG00000168096.10	ENSG00000171595.9
	ENSG00000174799.6	ENSG00000178741.7	ENSG00000180178.6	ENSG00000182108.5
	ENSG00000182324.5	ENSG00000183576.8	ENSG00000185955.4	ENSG00000188674.6
	ENSG00000196628.9	ENSG00000197409.6	ENSG00000203499.6	ENSG00000205871.4
	ENSG00000213225.6	ENSG00000218016.2	ENSG00000224786.1	ENSG00000228397.1
	ENSG00000229291.1	ENSG00000230850.3	ENSG00000232626.3	ENSG00000233913.6
	ENSG00000236136.1	ENSG00000236155.2	ENSG00000240747.3	ENSG00000241416.1
	ENSG00000250215.1	ENSG00000250635.1	ENSG00000254847.1	ENSG00000258418.1
	ENSG00000271737.1	ENSG00000272048.1		
**Brain Substantia nigra**	ENSG00000087086.9	ENSG00000100365.10	ENSG00000100938.13	ENSG00000103064.9
	ENSG00000105929.11	ENSG00000132424.10	ENSG00000143127.8	ENSG00000146670.5
	ENSG00000149634.4	ENSG00000153162.8	ENSG00000154080.8	ENSG00000159189.7
	ENSG00000160602.9	ENSG00000162434.7	ENSG00000162928.8	ENSG00000164744.8
	ENSG00000164749.7	ENSG00000165417.7	ENSG00000166340.10	ENSG00000167862.5
	ENSG00000170584.6	ENSG00000176055.9	ENSG00000182108.5	ENSG00000186660.14
	ENSG00000196214.6	ENSG00000196646.7	ENSG00000197322.1	ENSG00000203260.2
	ENSG00000213225.6	ENSG00000213355.3	ENSG00000229344.1	ENSG00000230850.3
	ENSG00000231720.1	ENSG00000232626.3	ENSG00000232833.4	ENSG00000236136.1
	ENSG00000242615.1	ENSG00000248208.1	ENSG00000250635.1	ENSG00000251410.1
	ENSG00000258418.1	ENSG00000261787.1	ENSG00000271143.1	
**Cells EBV**	ENSG00000009724.12	ENSG00000010282.10	ENSG00000047249.12	ENSG00000058668.10
	ENSG00000074696.8	ENSG00000075223.9	ENSG00000079263.14	ENSG00000081189.9
	ENSG00000083093.5	ENSG00000083635.7	ENSG00000092470.7	ENSG00000096696.9
	ENSG00000100365.10	ENSG00000100938.13	ENSG00000103064.9	ENSG00000104951.11
	ENSG00000104976.7	ENSG00000105948.9	ENSG00000105997.18	ENSG00000108094.10
	ENSG00000109189.8	ENSG00000113161.11	ENSG00000114019.10	ENSG00000115274.10
	ENSG00000120438.7	ENSG00000124491.11	ENSG00000125319.10	ENSG00000125910.4
	ENSG00000128191.9	ENSG00000132716.14	ENSG00000133114.13	ENSG00000133943.16
	ENSG00000136653.15	ENSG00000137817.12	ENSG00000140464.15	ENSG00000141380.9
	ENSG00000143740.10	ENSG00000144868.9	ENSG00000145833.11	ENSG00000154920.10
	ENSG00000155666.7	ENSG00000159189.7	ENSG00000159593.10	ENSG00000159674.7
	ENSG00000160602.9	ENSG00000160789.15	ENSG00000162434.7	ENSG00000163788.9

	ENSG00000165029.11	ENSG00000165417.7	ENSG00000166340.10	ENSG00000167700.4
	ENSG00000168658.14	ENSG00000169435.9	ENSG00000169964.5	ENSG00000170921.10
	ENSG00000171033.8	ENSG00000172331.7	ENSG00000173281.4	ENSG00000173545.4
	ENSG00000174799.6	ENSG00000176153.10	ENSG00000176371.9	ENSG00000179151.7
	ENSG00000180178.6	ENSG00000181744.4	ENSG00000181773.6	ENSG00000181982.13
	ENSG00000183473.5	ENSG00000183576.8	ENSG00000183801.3	ENSG00000184261.4
	ENSG00000184682.5	ENSG00000185955.4	ENSG00000187498.10	ENSG00000196453.7
	ENSG00000196700.3	ENSG00000196754.6	ENSG00000197776.3	ENSG00000198668.6
	ENSG00000213394.3	ENSG00000217555.8	ENSG00000223945.2	ENSG00000224221.1
	ENSG00000224565.1	ENSG00000228397.1	ENSG00000230850.3	ENSG00000231485.1
	ENSG00000233610.1	ENSG00000236136.1	ENSG00000237522.1	ENSG00000248161.1
	ENSG00000248487.4	ENSG00000248626.1	ENSG00000250635.1	ENSG00000255624.1
	ENSG00000261787.1	ENSG00000262488.1	ENSG00000264714.1	ENSG00000265095.1
	ENSG00000266604.1	ENSG00000266853.1	ENSG00000267416.1	ENSG00000269165.1
	ENSG00000272798.1	ENSG00000272828.1	ENSG00000272942.1	
**Cells Transformed**	ENSG00000004660.10	ENSG00000004776.7	ENSG00000004975.7	ENSG00000005189.15
	ENSG00000006704.6	ENSG00000008394.8	ENSG00000010282.10	ENSG00000024526.12
	ENSG00000047249.12	ENSG00000049541.6	ENSG00000051128.14	ENSG00000058262.5
	ENSG00000058668.10	ENSG00000060971.13	ENSG00000068383.14	ENSG00000071564.10
	ENSG00000074696.8	ENSG00000081052.10	ENSG00000081277.7	ENSG00000089723.5
	ENSG00000091009.6	ENSG00000099139.9	ENSG00000099246.12	ENSG00000100150.12
	ENSG00000100281.9	ENSG00000100365.10	ENSG00000100938.13	ENSG00000103064.9
	ENSG00000103160.7	ENSG00000104369.4	ENSG00000104951.11	ENSG00000104960.11
	ENSG00000105221.12	ENSG00000105792.15	ENSG00000105974.7	ENSG00000107443.11
	ENSG00000109066.9	ENSG00000109819.4	ENSG00000112144.11	ENSG00000112659.9
	ENSG00000113161.11	ENSG00000114019.10	ENSG00000114841.13	ENSG00000115274.10
	ENSG00000117475.9	ENSG00000117620.8	ENSG00000118894.10	ENSG00000118985.10
	ENSG00000119927.9	ENSG00000120686.7	ENSG00000121335.10	ENSG00000121774.13
	ENSG00000123500.5	ENSG00000124491.11	ENSG00000124813.16	ENSG00000130584.6
	ENSG00000131386.13	ENSG00000131931.4	ENSG00000133114.13	ENSG00000133247.9
	ENSG00000133943.16	ENSG00000134508.8	ENSG00000134571.6	ENSG00000134853.7
	ENSG00000134884.9	ENSG00000135324.5	ENSG00000135775.9	ENSG00000135898.5
	ENSG00000136021.13	ENSG00000138160.4	ENSG00000138675.12	ENSG00000139218.13
	ENSG00000139567.8	ENSG00000139687.9	ENSG00000140280.9	ENSG00000140464.15
	ENSG00000140577.11	ENSG00000140740.6	ENSG00000141255.8	ENSG00000142534.2
	ENSG00000142949.12	ENSG00000143157.7	ENSG00000144868.9	ENSG00000145088.4
	ENSG00000147573.12	ENSG00000148288.7	ENSG00000148926.5	ENSG00000149212.6
	ENSG00000149634.4	ENSG00000152128.13	ENSG00000154122.8	ENSG00000154479.8
	ENSG00000155097.7	ENSG00000155282.7	ENSG00000155313.11	ENSG00000155666.7
	ENSG00000155755.14	ENSG00000156170.8	ENSG00000156875.9	ENSG00000157227.8
	ENSG00000158055.11	ENSG00000159189.7	ENSG00000159674.7	ENSG00000160460.11
	ENSG00000160602.9	ENSG00000162434.7	ENSG00000163145.8	ENSG00000164169.8
	ENSG00000164620.4	ENSG00000164742.10	ENSG00000164744.8	ENSG00000165029.11
	ENSG00000165417.7	ENSG00000165527.5	ENSG00000165644.6	ENSG00000166091.15
	ENSG00000166233.8	ENSG00000166340.10	ENSG00000166482.7	ENSG00000166704.7
	ENSG00000167862.5	ENSG00000168096.10	ENSG00000168374.6	ENSG00000168398.5
	ENSG00000168490.9	ENSG00000168658.14	ENSG00000169403.7	ENSG00000170027.5
	ENSG00000170584.6	ENSG00000170899.6	ENSG00000170921.10	ENSG00000171033.8
	ENSG00000171621.9	ENSG00000172663.4	ENSG00000173175.10	ENSG00000173545.4

	ENSG00000174799.6	ENSG00000175564.8	ENSG00000176095.7	ENSG00000176153.10
	ENSG00000178694.5	ENSG00000178826.6	ENSG00000178878.8	ENSG00000179935.5
	ENSG00000180178.6	ENSG00000180712.3	ENSG00000180801.11	ENSG00000180891.8
	ENSG00000181773.6	ENSG00000181982.13	ENSG00000182175.9	ENSG00000183576.8
	ENSG00000184009.5	ENSG00000185567.6	ENSG00000186777.7	ENSG00000187498.10
	ENSG00000187742.10	ENSG00000188242.4	ENSG00000189298.9	ENSG00000196453.7
	ENSG00000197111.11	ENSG00000197386.6	ENSG00000197409.6	ENSG00000197429.6
	ENSG00000197771.8	ENSG00000197961.7	ENSG00000198134.2	ENSG00000198668.6
	ENSG00000205629.7	ENSG00000205726.9	ENSG00000213225.6	ENSG00000213355.3
	ENSG00000213394.3	ENSG00000214013.5	ENSG00000214146.2	ENSG00000215158.5
	ENSG00000215788.5	ENSG00000217241.1	ENSG00000218016.2	ENSG00000221955.6
	ENSG00000222047.4	ENSG00000223945.2	ENSG00000224914.2	ENSG00000225526.4
	ENSG00000225614.2	ENSG00000226197.2	ENSG00000228223.1	ENSG00000228397.1
	ENSG00000229291.1	ENSG00000229373.4	ENSG00000230850.3	ENSG00000231485.1
	ENSG00000231720.1	ENSG00000232316.1	ENSG00000232833.4	ENSG00000233077.1
	ENSG00000233762.2	ENSG00000234371.5	ENSG00000234536.1	ENSG00000235272.1
	ENSG00000235957.1	ENSG00000236136.1	ENSG00000236155.2	ENSG00000236700.1
	ENSG00000237094.7	ENSG00000240350.1	ENSG00000240682.5	ENSG00000240747.3
	ENSG00000248334.2	ENSG00000248632.1	ENSG00000250548.2	ENSG00000250635.1
	ENSG00000253304.1	ENSG00000253390.1	ENSG00000255010.1	ENSG00000255282.2
	ENSG00000255624.1	ENSG00000256060.2	ENSG00000256540.1	ENSG00000257464.1
	ENSG00000258418.1	ENSG00000259404.1	ENSG00000259502.1	ENSG00000261787.1
	ENSG00000262454.1	ENSG00000266853.1	ENSG00000267010.1	ENSG00000267416.1
	ENSG00000272308.1	ENSG00000272347.1	ENSG00000272377.1	ENSG00000272828.1
	ENSG00000272942.1			
**Colon Sigmoid**	ENSG00000004975.7	ENSG00000015592.12	ENSG00000047249.12	ENSG00000058668.10
	ENSG00000061656.5	ENSG00000074696.8	ENSG00000108773.6	ENSG00000109066.9
	ENSG00000111275.8	ENSG00000115163.10	ENSG00000115274.10	ENSG00000118894.10
	ENSG00000121848.9	ENSG00000131386.13	ENSG00000133114.13	ENSG00000133943.16
	ENSG00000139926.11	ENSG00000149634.4	ENSG00000151014.4	ENSG00000153993.9
	ENSG00000155282.7	ENSG00000155666.7	ENSG00000159164.5	ENSG00000159189.7
	ENSG00000159314.7	ENSG00000160221.12	ENSG00000162434.7	ENSG00000164744.8
	ENSG00000165525.13	ENSG00000168658.14	ENSG00000174407.7	ENSG00000174799.6
	ENSG00000178741.7	ENSG00000179523.4	ENSG00000180178.6	ENSG00000183576.8
	ENSG00000186844.4	ENSG00000187566.3	ENSG00000198668.6	ENSG00000204231.6
	ENSG00000213225.6	ENSG00000213355.3	ENSG00000214413.3	ENSG00000215481.4
	ENSG00000224106.1	ENSG00000228778.1	ENSG00000228804.1	ENSG00000230850.3
	ENSG00000232833.4	ENSG00000233610.1	ENSG00000235363.1	ENSG00000236136.1
	ENSG00000236814.1	ENSG00000237672.1	ENSG00000242791.2	ENSG00000249430.1
	ENSG00000249485.1	ENSG00000249889.1	ENSG00000255277.2	ENSG00000257464.1
	ENSG00000260727.1	ENSG00000261787.1	ENSG00000267416.1	ENSG00000267741.1
	ENSG00000271927.1	ENSG00000272201.1		
**Colon Transverse**	ENSG00000006704.6	ENSG00000010282.10	ENSG00000023734.6	ENSG00000047249.12
	ENSG00000063244.8	ENSG00000074696.8	ENSG00000081248.6	ENSG00000082269.12
	ENSG00000085741.8	ENSG00000099246.12	ENSG00000101624.6	ENSG00000103064.9
	ENSG00000103160.7	ENSG00000104164.6	ENSG00000104537.12	ENSG00000105063.14
	ENSG00000108773.6	ENSG00000109066.9	ENSG00000111701.6	ENSG00000111897.6
	ENSG00000112312.5	ENSG00000113161.11	ENSG00000114656.6	ENSG00000114737.11
	ENSG00000115274.10	ENSG00000118733.12	ENSG00000118816.5	ENSG00000118894.10

	ENSG00000119714.6	ENSG00000121892.10	ENSG00000132554.15	ENSG00000132958.13
	ENSG00000133114.13	ENSG00000134571.6	ENSG00000135722.4	ENSG00000135775.9
	ENSG00000135898.5	ENSG00000135945.5	ENSG00000136504.7	ENSG00000138785.10
	ENSG00000139351.10	ENSG00000140280.9	ENSG00000140939.10	ENSG00000144028.10
	ENSG00000148926.5	ENSG00000149212.6	ENSG00000154316.10	ENSG00000154485.4
	ENSG00000155666.7	ENSG00000157851.12	ENSG00000159164.5	ENSG00000159189.7
	ENSG00000162065.7	ENSG00000162434.7	ENSG00000163449.6	ENSG00000163811.7
	ENSG00000165417.7	ENSG00000166557.8	ENSG00000167476.6	ENSG00000167862.5
	ENSG00000168036.12	ENSG00000168260.1	ENSG00000168374.6	ENSG00000168398.5
	ENSG00000168658.14	ENSG00000170855.3	ENSG00000171033.8	ENSG00000174799.6
	ENSG00000175206.6	ENSG00000177888.7	ENSG00000178741.7	ENSG00000180061.5
	ENSG00000180178.6	ENSG00000181744.4	ENSG00000181982.13	ENSG00000183273.2
	ENSG00000183576.8	ENSG00000183684.6	ENSG00000186076.4	ENSG00000187498.10
	ENSG00000188404.4	ENSG00000189068.5	ENSG00000197386.6	ENSG00000197409.6
	ENSG00000197930.8	ENSG00000198668.6	ENSG00000204150.7	ENSG00000205629.7
	ENSG00000205838.8	ENSG00000213225.6	ENSG00000214146.2	ENSG00000214381.4
	ENSG00000223823.1	ENSG00000227742.1	ENSG00000228397.1	ENSG00000228797.2
	ENSG00000230850.3	ENSG00000231485.1	ENSG00000233469.1	ENSG00000234383.1
	ENSG00000236136.1	ENSG00000236814.1	ENSG00000243536.3	ENSG00000245651.2
	ENSG00000248243.1	ENSG00000254305.1	ENSG00000254573.1	ENSG00000254639.1
	ENSG00000254982.1	ENSG00000257464.1	ENSG00000257859.1	ENSG00000258418.1
	ENSG00000259158.1	ENSG00000259404.1	ENSG00000265095.1	ENSG00000267289.1
	ENSG00000267416.1	ENSG00000268172.1	ENSG00000272942.1	
**Esophagus Gastroesophageal**	ENSG00000005448.12	ENSG00000011143.12	ENSG00000047249.12	ENSG00000061676.10
	ENSG00000074696.8	ENSG00000085741.8	ENSG00000100281.9	ENSG00000104976.7
	ENSG00000105948.9	ENSG00000109066.9	ENSG00000116273.5	ENSG00000118733.12
	ENSG00000124194.11	ENSG00000128052.8	ENSG00000130173.9	ENSG00000132405.14
	ENSG00000134812.3	ENSG00000138160.4	ENSG00000144868.9	ENSG00000159189.7
	ENSG00000160218.8	ENSG00000162409.6	ENSG00000162434.7	ENSG00000162643.8
	ENSG00000165417.7	ENSG00000166340.10	ENSG00000166704.7	ENSG00000167862.5
	ENSG00000174799.6	ENSG00000177192.9	ENSG00000180210.10	ENSG00000182050.9
	ENSG00000182796.8	ENSG00000185955.4	ENSG00000187498.10	ENSG00000188827.6
	ENSG00000198821.6	ENSG00000213225.6	ENSG00000227331.1	ENSG00000230850.3
	ENSG00000233859.1	ENSG00000235145.2	ENSG00000236136.1	ENSG00000242960.1
	ENSG00000248927.1	ENSG00000254122.1	ENSG00000255277.2	ENSG00000257210.1
	ENSG00000257231.1	ENSG00000260518.1	ENSG00000267289.1	ENSG00000267416.1
	ENSG00000267680.1	ENSG00000268172.1	ENSG00000270270.1	ENSG00000272108.1
	ENSG00000272428.1	ENSG00000272430.1	ENSG00000272828.1	
**Esophagus Mucosa**	ENSG00000004975.7	ENSG00000005175.5	ENSG00000005448.12	ENSG00000005700.10
	ENSG00000006534.11	ENSG00000008394.8	ENSG00000010282.10	ENSG00000013588.5
	ENSG00000019186.5	ENSG00000034510.4	ENSG00000047249.12	ENSG00000058056.4
	ENSG00000058668.10	ENSG00000063322.9	ENSG00000069011.11	ENSG00000072201.9
	ENSG00000072571.15	ENSG00000074696.8	ENSG00000075223.9	ENSG00000078081.3
	ENSG00000081052.10	ENSG00000083635.7	ENSG00000085741.8	ENSG00000086967.9
	ENSG00000087303.12	ENSG00000088766.7	ENSG00000089723.5	ENSG00000092531.5
	ENSG00000099139.9	ENSG00000099204.14	ENSG00000099246.12	ENSG00000100246.8
	ENSG00000100281.9	ENSG00000100365.10	ENSG00000100938.13	ENSG00000101144.8
	ENSG00000101624.6	ENSG00000101670.7	ENSG00000103064.9	ENSG00000103160.7
	ENSG00000103742.7	ENSG00000104687.8	ENSG00000104951.11	ENSG00000104960.11

ENSG00000105379.5	ENSG00000105467.4	ENSG00000105792.15	ENSG00000105929.11
ENSG00000106299.7	ENSG00000107341.4	ENSG00000108094.10	ENSG00000108242.8
ENSG00000108773.6	ENSG00000108953.12	ENSG00000109066.9	ENSG00000109189.8
ENSG00000109458.4	ENSG00000110200.4	ENSG00000112149.5	ENSG00000112175.6
ENSG00000112312.5	ENSG00000112378.11	ENSG00000112394.12	ENSG00000112981.3
ENSG00000113161.11	ENSG00000113758.9	ENSG00000113790.6	ENSG00000114019.10
ENSG00000115091.7	ENSG00000115594.7	ENSG00000116544.7	ENSG00000116703.12
ENSG00000117448.9	ENSG00000117620.8	ENSG00000117862.7	ENSG00000118242.11
ENSG00000118496.4	ENSG00000118520.9	ENSG00000118680.8	ENSG00000118894.10
ENSG00000118985.10	ENSG00000120686.7	ENSG00000120756.8	ENSG00000121900.14
ENSG00000121905.5	ENSG00000124253.9	ENSG00000125910.4	ENSG00000125995.11
ENSG00000126107.10	ENSG00000126368.5	ENSG00000127743.5	ENSG00000128052.8
ENSG00000130584.6	ENSG00000130707.13	ENSG00000130844.12	ENSG00000132254.8
ENSG00000132604.6	ENSG00000133048.8	ENSG00000133114.13	ENSG00000134250.13
ENSG00000134571.6	ENSG00000134884.9	ENSG00000135324.5	ENSG00000135503.8
ENSG00000135775.9	ENSG00000135945.5	ENSG00000136160.10	ENSG00000136267.9
ENSG00000136319.7	ENSG00000136536.10	ENSG00000138160.4	ENSG00000138642.10
ENSG00000139546.6	ENSG00000139988.5	ENSG00000140254.8	ENSG00000140280.9
ENSG00000140395.4	ENSG00000142627.9	ENSG00000143106.8	ENSG00000143740.10
ENSG00000144395.13	ENSG00000144868.9	ENSG00000145700.5	ENSG00000146859.6
ENSG00000147689.12	ENSG00000148926.5	ENSG00000149418.6	ENSG00000149634.4
ENSG00000149929.11	ENSG00000151500.10	ENSG00000152128.13	ENSG00000153094.17
ENSG00000153993.9	ENSG00000154479.8	ENSG00000155189.7	ENSG00000155666.7
ENSG00000155755.14	ENSG00000155918.3	ENSG00000156026.10	ENSG00000157538.9
ENSG00000157657.10	ENSG00000158055.11	ENSG00000158864.8	ENSG00000159189.7
ENSG00000159674.7	ENSG00000160602.9	ENSG00000160710.11	ENSG00000162434.7
ENSG00000162888.4	ENSG00000163113.10	ENSG00000163449.6	ENSG00000163486.8
ENSG00000164054.11	ENSG00000165417.7	ENSG00000165487.9	ENSG00000165659.12
ENSG00000165672.5	ENSG00000166068.8	ENSG00000166091.15	ENSG00000166340.10
ENSG00000166557.8	ENSG00000166575.12	ENSG00000166704.7	ENSG00000166823.5
ENSG00000166840.9	ENSG00000167112.7	ENSG00000167281.14	ENSG00000167757.9
ENSG00000167759.8	ENSG00000167862.5	ENSG00000167972.9	ENSG00000168061.9
ENSG00000168079.12	ENSG00000168216.6	ENSG00000168389.13	ENSG00000168398.5
ENSG00000168564.5	ENSG00000168658.14	ENSG00000169129.10	ENSG00000169403.7
ENSG00000169435.9	ENSG00000169599.8	ENSG00000169908.6	ENSG00000171219.8
ENSG00000171467.11	ENSG00000171793.9	ENSG00000171858.13	ENSG00000172243.13
ENSG00000172432.14	ENSG00000172717.11	ENSG00000173175.10	ENSG00000174576.4
ENSG00000174672.11	ENSG00000174799.6	ENSG00000176095.7	ENSG00000176153.10
ENSG00000176771.11	ENSG00000177888.7	ENSG00000177943.9	ENSG00000178234.8
ENSG00000178401.10	ENSG00000178826.6	ENSG00000180178.6	ENSG00000180801.11
ENSG00000180828.1	ENSG00000180871.3	ENSG00000181634.7	ENSG00000181744.4
ENSG00000181982.13	ENSG00000182175.9	ENSG00000183055.5	ENSG00000183576.8
ENSG00000185475.4	ENSG00000186188.6	ENSG00000186197.8	ENSG00000187017.10
ENSG00000187566.3	ENSG00000188242.4	ENSG00000189060.4	ENSG00000189159.11
ENSG00000196581.6	ENSG00000196639.6	ENSG00000196754.6	ENSG00000196776.10
ENSG00000197647.7	ENSG00000197756.5	ENSG00000197757.7	ENSG00000197905.4
ENSG00000197930.8	ENSG00000197956.5	ENSG00000198033.7	ENSG00000198454.2
ENSG00000198517.5	ENSG00000198554.7	ENSG00000203499.6	ENSG00000203602.1
ENSG00000203688.4	ENSG00000203711.7	ENSG00000203859.5	ENSG00000204544.5

	ENSG00000205629.7	ENSG00000213225.6	ENSG00000213355.3	ENSG00000213376.4
	ENSG00000213551.4	ENSG00000213593.5	ENSG00000214013.5	ENSG00000214146.2
	ENSG00000214362.2	ENSG00000215187.5	ENSG00000215481.4	ENSG00000215571.4
	ENSG00000215788.5	ENSG00000221887.4	ENSG00000223823.1	ENSG00000224186.4
	ENSG00000224956.5	ENSG00000225930.3	ENSG00000228223.1	ENSG00000228397.1
	ENSG00000229291.1	ENSG00000230850.3	ENSG00000231720.1	ENSG00000232815.1
	ENSG00000232833.4	ENSG00000233610.1	ENSG00000233901.1	ENSG00000234536.1
	ENSG00000234797.4	ENSG00000234862.1	ENSG00000235725.1	ENSG00000236136.1
	ENSG00000236983.1	ENSG00000237697.2	ENSG00000240350.1	ENSG00000241134.2
	ENSG00000241678.1	ENSG00000242114.1	ENSG00000243300.2	ENSG00000246922.4
	ENSG00000248632.1	ENSG00000248916.1	ENSG00000253390.1	ENSG00000254332.1
	ENSG00000254398.1	ENSG00000255277.2	ENSG00000255561.2	ENSG00000255595.1
	ENSG00000256060.2	ENSG00000256167.1	ENSG00000256720.1	ENSG00000256968.1
	ENSG00000257464.1	ENSG00000257515.1	ENSG00000258418.1	ENSG00000258811.1
	ENSG00000258929.2	ENSG00000259158.1	ENSG00000259404.1	ENSG00000259807.1
	ENSG00000260402.1	ENSG00000262973.1	ENSG00000266498.1	ENSG00000266946.1
	ENSG00000267114.1	ENSG00000267416.1	ENSG00000268087.1	ENSG00000269962.1
	ENSG00000271020.1	ENSG00000271711.1	ENSG00000271856.1	ENSG00000272235.1
	ENSG00000272274.1	ENSG00000272347.1	ENSG00000272520.1	ENSG00000272523.1
NSG00000272567.1	ENSG00000272908.1	ENSG00000272942.1	ENSG00000272966.1	
	ENSG00000004975.7	ENSG00000005448.12	ENSG00000005700.10	ENSG00000007384.11
	ENSG00000010282.10	ENSG00000047249.12	ENSG00000061987.10	ENSG00000063241.3
	ENSG00000068383.14	ENSG00000074054.13	ENSG00000074696.8	ENSG00000076604.10
	ENSG00000077522.8	ENSG00000079156.12	ENSG00000085741.8	ENSG00000089159.11
	ENSG00000100281.9	ENSG00000100365.10	ENSG00000101082.9	ENSG00000103042.4
	ENSG00000103064.9	ENSG00000103160.7	ENSG00000104369.4	ENSG00000104517.8
	ENSG00000104960.11	ENSG00000105372.2	ENSG00000105792.15	ENSG00000105948.9
	ENSG00000105968.14	ENSG00000106261.12	ENSG00000108387.10	ENSG00000108641.10
	ENSG00000109066.9	ENSG00000109738.6	ENSG00000112659.9	ENSG00000112981.3
**Esophagus**	ENSG00000113161.11	ENSG00000113209.6	ENSG00000114353.12	ENSG00000115163.10
**Muscularis**	ENSG00000115274.10	ENSG00000115840.9	ENSG00000116337.11	ENSG00000117862.7
	ENSG00000118733.12	ENSG00000118894.10	ENSG00000119514.5	ENSG00000119703.12
	ENSG00000120686.7	ENSG00000120937.8	ENSG00000123505.10	ENSG00000125743.6
	ENSG00000128052.8	ENSG00000130173.9	ENSG00000130584.6	ENSG00000130958.7
	ENSG00000132326.7	ENSG00000132405.14	ENSG00000132975.6	ENSG00000133114.13
	ENSG00000133943.16	ENSG00000134508.8	ENSG00000134571.6	ENSG00000134812.3
	ENSG00000134884.9	ENSG00000135775.9	ENSG00000135821.12	ENSG00000135898.5
	ENSG00000136160.10	ENSG00000136267.9	ENSG00000136485.10	ENSG00000136925.10
	ENSG00000137941.12	ENSG00000138160.4	ENSG00000139269.2	ENSG00000139684.9
	ENSG00000140254.8	ENSG00000140280.9	ENSG00000140395.4	ENSG00000141441.11
	ENSG00000141665.7	ENSG00000143157.7	ENSG00000144426.14	ENSG00000144868.9
	ENSG00000145388.10	ENSG00000149634.4	ENSG00000150625.12	ENSG00000152620.8
	ENSG00000152818.14	ENSG00000154080.8	ENSG00000154122.8	ENSG00000154316.10
	ENSG00000154479.8	ENSG00000154589.2	ENSG00000154654.10	ENSG00000155666.7
	ENSG00000158055.11	ENSG00000159189.7	ENSG00000159374.13	ENSG00000159674.7
	ENSG00000160221.12	ENSG00000162373.8	ENSG00000162434.7	ENSG00000163145.8
	ENSG00000164054.11	ENSG00000164188.4	ENSG00000164620.4	ENSG00000165417.7
	ENSG00000166106.2	ENSG00000166340.10	ENSG00000166482.7	ENSG00000166704.7
	ENSG00000167196.9	ENSG00000167604.9	ENSG00000167862.5	ENSG00000167985.2

	ENSG00000168081.4	ENSG00000168389.13	ENSG00000168564.5	ENSG00000168658.14
	ENSG00000169031.14	ENSG00000169733.7	ENSG00000170540.10	ENSG00000170899.6
	ENSG00000171219.8	ENSG00000171606.13	ENSG00000171777.11	ENSG00000171819.4
	ENSG00000173801.12	ENSG00000174407.7	ENSG00000174576.4	ENSG00000174672.11
	ENSG00000174748.14	ENSG00000174799.6	ENSG00000175029.12	ENSG00000175426.6
	ENSG00000175664.5	ENSG00000176105.9	ENSG00000176153.10	ENSG00000176371.9
	ENSG00000177888.7	ENSG00000178096.8	ENSG00000178826.6	ENSG00000179902.8
	ENSG00000180178.6	ENSG00000180210.10	ENSG00000180611.6	ENSG00000180801.11
	ENSG00000180828.1	ENSG00000181234.8	ENSG00000181778.4	ENSG00000181982.13
	ENSG00000182108.5	ENSG00000182831.7	ENSG00000183801.3	ENSG00000184009.5
	ENSG00000184752.8	ENSG00000184999.7	ENSG00000186188.6	ENSG00000186960.6
	ENSG00000187498.10	ENSG00000187566.3	ENSG00000187607.11	ENSG00000187742.10
	ENSG00000187796.9	ENSG00000188038.3	ENSG00000188242.4	ENSG00000196155.8
	ENSG00000196182.6	ENSG00000198517.5	ENSG00000198668.6	ENSG00000198821.6
	ENSG00000198865.5	ENSG00000204150.7	ENSG00000205129.4	ENSG00000205189.7
	ENSG00000205629.7	ENSG00000206432.4	ENSG00000206579.7	ENSG00000213212.3
	ENSG00000213225.6	ENSG00000213930.7	ENSG00000215158.5	ENSG00000221955.6
	ENSG00000224914.2	ENSG00000226005.3	ENSG00000226374.1	ENSG00000228008.1
	ENSG00000228397.1	ENSG00000229291.1	ENSG00000230850.3	ENSG00000231322.1
	ENSG00000232273.1	ENSG00000232833.4	ENSG00000233087.4	ENSG00000233593.2
	ENSG00000234065.2	ENSG00000235957.1	ENSG00000236136.1	ENSG00000236976.1
	ENSG00000237522.1	ENSG00000237668.1	ENSG00000240747.3	ENSG00000241163.3
	ENSG00000243234.1	ENSG00000243300.2	ENSG00000250238.1	ENSG00000253304.1
	ENSG00000255561.2	ENSG00000256361.1	ENSG00000257259.1	ENSG00000257464.1
	ENSG00000258569.1	ENSG00000258811.1	ENSG00000259158.1	ENSG00000259404.1
	ENSG00000259758.1	ENSG00000260464.1	ENSG00000264057.1	ENSG00000266932.1
	ENSG00000267416.1	ENSG00000268079.1	ENSG00000268087.1	ENSG00000269282.1
	ENSG00000269510.1	ENSG00000269873.1	ENSG00000272942.1	ENSG00000273181.1
**Heart Atrial**	ENSG00000004975.7	ENSG00000023734.6	ENSG00000047249.12	ENSG00000051128.14
	ENSG00000069667.11	ENSG00000072201.9	ENSG00000074696.8	ENSG00000075223.9
	ENSG00000077044.5	ENSG00000081818.1	ENSG00000085274.11	ENSG00000085741.8
	ENSG00000086065.9	ENSG00000092531.5	ENSG00000100281.9	ENSG00000100351.12
	ENSG00000100365.10	ENSG00000101898.5	ENSG00000103160.7	ENSG00000103197.12
	ENSG00000104341.12	ENSG00000104408.5	ENSG00000105366.11	ENSG00000109066.9
	ENSG00000113161.11	ENSG00000113209.6	ENSG00000114648.7	ENSG00000115274.10
	ENSG00000116273.5	ENSG00000116754.9	ENSG00000120686.7	ENSG00000120833.9
	ENSG00000128918.10	ENSG00000130935.5	ENSG00000133114.13	ENSG00000133392.12
	ENSG00000134352.15	ENSG00000134571.6	ENSG00000135945.5	ENSG00000137941.12
	ENSG00000139233.2	ENSG00000140280.9	ENSG00000140937.9	ENSG00000141255.8
	ENSG00000142627.9	ENSG00000144868.9	ENSG00000148288.7	ENSG00000149634.4
	ENSG00000150048.6	ENSG00000151014.4	ENSG00000159189.7	ENSG00000159674.7
	ENSG00000160050.10	ENSG00000162009.7	ENSG00000162889.6	ENSG00000163449.6
	ENSG00000165417.7	ENSG00000165623.5	ENSG00000166091.15	ENSG00000166340.10
	ENSG00000167210.12	ENSG00000167941.2	ENSG00000168172.4	ENSG00000168658.14
	ENSG00000169964.5	ENSG00000170921.10	ENSG00000173175.10	ENSG00000174799.6
	ENSG00000180801.11	ENSG00000181744.4	ENSG00000181982.13	ENSG00000183576.8
	ENSG00000185532.10	ENSG00000186188.6	ENSG00000187498.10	ENSG00000188242.4
	ENSG00000196754.6	ENSG00000198668.6	ENSG00000204231.6	ENSG00000204396.6
	ENSG00000206432.4	ENSG00000213225.6	ENSG00000213355.3	ENSG00000213551.4

	ENSG00000213930.7	ENSG00000215208.3	ENSG00000224566.2	ENSG00000228397.1
	ENSG00000229291.1	ENSG00000230799.1	ENSG00000230850.3	ENSG00000231369.1
	ENSG00000231615.2	ENSG00000236136.1	ENSG00000236155.2	ENSG00000238102.1
	ENSG00000242114.1	ENSG00000249306.1	ENSG00000250635.1	ENSG00000253433.1
	ENSG00000254618.1	ENSG00000258418.1	ENSG00000268518.1	ENSG00000268573.1
	ENSG00000270987.1	ENSG00000272567.1		
**Heart Left Ventricle**	ENSG00000004975.7	ENSG00000023734.6	ENSG00000047249.12	ENSG00000049246.10
	ENSG00000051128.14	ENSG00000058668.10	ENSG00000058866.10	ENSG00000063244.8
	ENSG00000068305.13	ENSG00000074696.8	ENSG00000077044.5	ENSG00000080493.9
	ENSG00000084734.4	ENSG00000089159.11	ENSG00000100365.10	ENSG00000103064.9
	ENSG00000104408.5	ENSG00000105255.6	ENSG00000105968.14	ENSG00000106689.6
	ENSG00000109066.9	ENSG00000109787.8	ENSG00000114648.7	ENSG00000115274.10
	ENSG00000116273.5	ENSG00000119514.5	ENSG00000120686.7	ENSG00000121879.3
	ENSG00000131386.13	ENSG00000131931.4	ENSG00000133114.13	ENSG00000134571.6
	ENSG00000135898.5	ENSG00000137941.12	ENSG00000138785.10	ENSG00000139546.6
	ENSG00000141456.10	ENSG00000143222.7	ENSG00000143740.10	ENSG00000144868.9
	ENSG00000147588.6	ENSG00000149634.4	ENSG00000152128.13	ENSG00000154122.8
	ENSG00000155666.7	ENSG00000159189.7	ENSG00000159674.7	ENSG00000162434.7
	ENSG00000162889.6	ENSG00000163449.6	ENSG00000164744.8	ENSG00000165417.7
	ENSG00000166340.10	ENSG00000167862.5	ENSG00000168658.14	ENSG00000170921.10
	ENSG00000170989.8	ENSG00000171033.8	ENSG00000172264.12	ENSG00000174748.14
	ENSG00000174799.6	ENSG00000178826.6	ENSG00000180178.6	ENSG00000181035.9
	ENSG00000181227.2	ENSG00000181315.6	ENSG00000183576.8	ENSG00000184261.4
	ENSG00000184307.9	ENSG00000184557.3	ENSG00000186141.4	ENSG00000186197.8
	ENSG00000196104.6	ENSG00000196754.6	ENSG00000196757.3	ENSG00000197744.5
	ENSG00000203614.3	ENSG00000204396.6	ENSG00000213225.6	ENSG00000214203.4
	ENSG00000226005.3	ENSG00000227176.1	ENSG00000227934.1	ENSG00000228397.1
	ENSG00000228797.2	ENSG00000229859.4	ENSG00000230850.3	ENSG00000231684.2
	ENSG00000232815.1	ENSG00000233775.1	ENSG00000234589.3	ENSG00000234664.1
	ENSG00000236155.2	ENSG00000238102.1	ENSG00000240907.1	ENSG00000250635.1
	ENSG00000253641.1	ENSG00000253669.3	ENSG00000255561.2	ENSG00000255595.1
	ENSG00000256443.1	ENSG00000257464.1	ENSG00000259404.1	ENSG00000260518.1
	ENSG00000260747.1	ENSG00000261609.3	ENSG00000261787.1	ENSG00000268573.1
	ENSG00000269165.1	ENSG00000269895.1	ENSG00000270750.1	ENSG00000270947.1
	ENSG00000271882.1	ENSG00000272428.1	ENSG00000272682.1	ENSG00000272906.1
**Liver**	ENSG00000009724.12	ENSG00000010282.10	ENSG00000047249.12	ENSG00000051128.14
	ENSG00000061794.8	ENSG00000068001.9	ENSG00000074696.8	ENSG00000075223.9
	ENSG00000085741.8	ENSG00000089159.11	ENSG00000101204.11	ENSG00000103064.9
	ENSG00000106948.12	ENSG00000115998.3	ENSG00000116752.5	ENSG00000125846.11
	ENSG00000125910.4	ENSG00000125970.7	ENSG00000129071.5	ENSG00000130305.12
	ENSG00000131236.12	ENSG00000132467.2	ENSG00000133048.8	ENSG00000133114.13
	ENSG00000134352.15	ENSG00000134571.6	ENSG00000136149.6	ENSG00000137941.12
	ENSG00000140488.10	ENSG00000142541.12	ENSG00000145088.4	ENSG00000148123.10
	ENSG00000149634.4	ENSG00000151014.4	ENSG00000152128.13	ENSG00000155666.7
	ENSG00000156026.10	ENSG00000159189.7	ENSG00000162434.7	ENSG00000163113.10
	ENSG00000165417.7	ENSG00000165527.5	ENSG00000166340.10	ENSG00000167487.7
	ENSG00000172243.13	ENSG00000174799.6	ENSG00000176055.9	ENSG00000176182.5
	ENSG00000176371.9	ENSG00000180178.6	ENSG00000181634.7	ENSG00000181982.13
	ENSG00000183576.8	ENSG00000185594.4	ENSG00000186141.4	ENSG00000186188.6

	ENSG00000187013.2	ENSG00000189409.8	ENSG00000212766.5	ENSG00000214146.2
	ENSG00000215187.5	ENSG00000218016.2	ENSG00000224003.1	ENSG00000224914.2
	ENSG00000228397.1	ENSG00000230850.3	ENSG00000231684.2	ENSG00000232815.1
	ENSG00000232833.4	ENSG00000236136.1	ENSG00000236155.2	ENSG00000238102.1
	ENSG00000240747.3	ENSG00000248208.1	ENSG00000255277.2	ENSG00000256162.2
	ENSG00000257464.1	ENSG00000266402.2	ENSG00000266498.1	ENSG00000267416.1
	ENSG00000270244.1			
**Lung**	ENSG00000004975.7	ENSG00000005189.15	ENSG00000005448.12	ENSG00000006704.6
	ENSG00000008226.15	ENSG00000010282.10	ENSG00000010327.6	ENSG00000033170.12
	ENSG00000047249.12	ENSG00000051128.14	ENSG00000053328.8	ENSG00000063241.3
	ENSG00000069431.6	ENSG00000071242.7	ENSG00000072135.8	ENSG00000072518.16
	ENSG00000073861.2	ENSG00000074696.8	ENSG00000081818.1	ENSG00000085274.11
	ENSG00000085741.8	ENSG00000086065.9	ENSG00000091704.5	ENSG00000092871.12
	ENSG00000096996.11	ENSG00000100281.9	ENSG00000100365.10	ENSG00000100368.9
	ENSG00000100938.13	ENSG00000101624.6	ENSG00000101670.7	ENSG00000102898.7
	ENSG00000102970.6	ENSG00000103064.9	ENSG00000103160.7	ENSG00000104408.5
	ENSG00000104970.6	ENSG00000107175.6	ENSG00000107295.8	ENSG00000108094.10
	ENSG00000108953.12	ENSG00000109066.9	ENSG00000109193.6	ENSG00000111729.8
	ENSG00000111837.7	ENSG00000112149.5	ENSG00000112640.10	ENSG00000112769.14
	ENSG00000113209.6	ENSG00000113790.6	ENSG00000115274.10	ENSG00000116711.8
	ENSG00000116752.5	ENSG00000118518.11	ENSG00000118894.10	ENSG00000120093.7
	ENSG00000120149.7	ENSG00000120686.7	ENSG00000120833.9	ENSG00000120913.19
	ENSG00000121690.5	ENSG00000121848.9	ENSG00000122188.8	ENSG00000123505.10
	ENSG00000125772.8	ENSG00000125910.4	ENSG00000126067.7	ENSG00000128052.8
	ENSG00000128266.7	ENSG00000130201.3	ENSG00000130487.4	ENSG00000130584.6
	ENSG00000131236.12	ENSG00000132842.9	ENSG00000133114.13	ENSG00000133606.6
	ENSG00000133943.16	ENSG00000134250.13	ENSG00000134571.6	ENSG00000135503.8
	ENSG00000135775.9	ENSG00000135898.5	ENSG00000135945.5	ENSG00000136160.10
	ENSG00000136319.7	ENSG00000136943.6	ENSG00000138160.4	ENSG00000140254.8
	ENSG00000140280.9	ENSG00000140395.4	ENSG00000141255.8	ENSG00000142687.13
	ENSG00000143190.17	ENSG00000143314.8	ENSG00000143515.12	ENSG00000144395.13
	ENSG00000144868.9	ENSG00000145088.4	ENSG00000146013.6	ENSG00000146166.12
	ENSG00000146476.6	ENSG00000147570.5	ENSG00000148356.9	ENSG00000148926.5
	ENSG00000149634.4	ENSG00000149735.2	ENSG00000149929.11	ENSG00000151014.4
	ENSG00000152595.12	ENSG00000152620.8	ENSG00000153094.17	ENSG00000155666.7
	ENSG00000159189.7	ENSG00000159674.7	ENSG00000160710.11	ENSG00000161671.12
	ENSG00000161911.7	ENSG00000162399.6	ENSG00000162434.7	ENSG00000163145.8
	ENSG00000163347.5	ENSG00000163395.12	ENSG00000163421.4	ENSG00000163449.6
	ENSG00000163618.13	ENSG00000163704.7	ENSG00000164074.10	ENSG00000164620.4
	ENSG00000164675.6	ENSG00000164736.5	ENSG00000165417.7	ENSG00000166091.15
	ENSG00000166340.10	ENSG00000166557.8	ENSG00000166704.7	ENSG00000167613.11
	ENSG00000167862.5	ENSG00000168079.12	ENSG00000168421.8	ENSG00000168658.14
	ENSG00000169174.9	ENSG00000169403.7	ENSG00000169599.8	ENSG00000169925.12
	ENSG00000170584.6	ENSG00000170832.8	ENSG00000171219.8	ENSG00000172538.6
	ENSG00000174799.6	ENSG00000175029.12	ENSG00000175664.5	ENSG00000176095.7
	ENSG00000176153.10	ENSG00000176371.9	ENSG00000176855.11	ENSG00000177888.7
	ENSG00000178826.6	ENSG00000179546.3	ENSG00000179902.8	ENSG00000180178.6
	ENSG00000180801.11	ENSG00000181035.9	ENSG00000181227.2	ENSG00000181744.4
	ENSG00000181982.13	ENSG00000182175.9	ENSG00000183576.8	ENSG00000184009.5

	ENSG00000184261.4	ENSG00000184378.2	ENSG00000184451.5	ENSG00000185641.5
	ENSG00000187116.9	ENSG00000187566.3	ENSG00000188404.4	ENSG00000189068.5
	ENSG00000189127.3	ENSG00000196116.6	ENSG00000197275.8	ENSG00000197386.6
	ENSG00000197409.6	ENSG00000197838.4	ENSG00000197894.6	ENSG00000197930.8
	ENSG00000198380.8	ENSG00000198384.7	ENSG00000198399.10	ENSG00000198517.5
	ENSG00000198788.7	ENSG00000198865.5	ENSG00000204150.7	ENSG00000204544.5
	ENSG00000205250.4	ENSG00000206418.3	ENSG00000213197.3	ENSG00000213225.6
	ENSG00000213598.3	ENSG00000213920.4	ENSG00000213930.7	ENSG00000214146.2
	ENSG00000216895.4	ENSG00000221955.6	ENSG00000224186.4	ENSG00000225079.2
	ENSG00000225614.2	ENSG00000227063.4	ENSG00000227077.2	ENSG00000227239.2
	ENSG00000227920.2	ENSG00000228000.1	ENSG00000228223.1	ENSG00000228397.1
	ENSG00000229291.1	ENSG00000229373.4	ENSG00000230850.3	ENSG00000231720.1
	ENSG00000232815.1	ENSG00000232837.1	ENSG00000234093.2	ENSG00000235373.1
	ENSG00000236136.1	ENSG00000237253.1	ENSG00000237372.1	ENSG00000240350.1
	ENSG00000240393.1	ENSG00000240747.3	ENSG00000243627.4	ENSG00000243824.1
	ENSG00000245651.2	ENSG00000248334.2	ENSG00000249601.2	ENSG00000250237.1
	ENSG00000254416.1	ENSG00000254786.1	ENSG00000255561.2	ENSG00000255735.1
	ENSG00000255864.1	ENSG00000256167.1	ENSG00000257210.1	ENSG00000257464.1
	ENSG00000258418.1	ENSG00000258780.1	ENSG00000258811.1	ENSG00000258929.2
	ENSG00000259158.1	ENSG00000259404.1	ENSG00000259508.1	ENSG00000261090.1
	ENSG00000261172.1	ENSG00000261353.1	ENSG00000261787.1	ENSG00000261839.1
	ENSG00000262194.1	ENSG00000264016.2	ENSG00000266498.1	ENSG00000267416.1
	ENSG00000267643.1	ENSG00000268087.1	ENSG00000268518.1	ENSG00000269271.1
	ENSG00000269282.1	ENSG00000270607.1	ENSG00000271587.1	ENSG00000271699.1
	ENSG00000271734.1	ENSG00000271856.1	ENSG00000272416.1	ENSG00000272908.1
	ENSG00000272960.1	ENSG00000273476.1		
**Minor Salivary**	ENSG00000005194.10	ENSG00000007314.7	ENSG00000010319.2	ENSG00000072201.9
	ENSG00000108773.6	ENSG00000115274.10	ENSG00000140835.8	ENSG00000149634.4
	ENSG00000152620.8	ENSG00000155666.7	ENSG00000159189.7	ENSG00000165417.7
	ENSG00000167862.5	ENSG00000169964.5	ENSG00000180178.6	ENSG00000180712.3
	ENSG00000182175.9	ENSG00000185250.11	ENSG00000185641.5	ENSG00000186076.4
	ENSG00000224914.2	ENSG00000225370.1	ENSG00000227934.1	ENSG00000228778.1
	ENSG00000230832.3	ENSG00000230850.3	ENSG00000232833.4	ENSG00000235701.1
	ENSG00000236155.2	ENSG00000255277.2	ENSG00000255595.1	ENSG00000258256.1
	ENSG00000272942.1			
**Muscle Skeletal**	ENSG00000006704.6	ENSG00000010282.10	ENSG00000011028.9	ENSG00000023734.6
	ENSG00000047249.12	ENSG00000047410.9	ENSG00000055332.12	ENSG00000056487.11
	ENSG00000058668.10	ENSG00000061676.10	ENSG00000061794.8	ENSG00000063241.3
	ENSG00000069667.11	ENSG00000072135.8	ENSG00000072195.10	ENSG00000072201.9
	ENSG00000074696.8	ENSG00000075223.9	ENSG00000077044.5	ENSG00000085741.8
	ENSG00000089022.9	ENSG00000092871.12	ENSG00000097033.10	ENSG00000100281.9
	ENSG00000100365.10	ENSG00000100938.13	ENSG00000101624.6	ENSG00000101782.10
	ENSG00000103064.9	ENSG00000104164.6	ENSG00000104205.8	ENSG00000104369.4
	ENSG00000104408.5	ENSG00000105401.2	ENSG00000105821.10	ENSG00000106948.12
	ENSG00000108671.5	ENSG00000108773.6	ENSG00000109066.9	ENSG00000109787.8
	ENSG00000111897.6	ENSG00000112701.13	ENSG00000112769.14	ENSG00000112855.10
	ENSG00000113048.12	ENSG00000113161.11	ENSG00000113205.2	ENSG00000113209.6
	ENSG00000113312.6	ENSG00000114902.9	ENSG00000115274.10	ENSG00000115514.7
	ENSG00000115998.3	ENSG00000116205.6	ENSG00000117425.9	ENSG00000118894.10

	ENSG00000118946.7	ENSG00000118985.10	ENSG00000119514.5	ENSG00000119703.12
	ENSG00000120686.7	ENSG00000121848.9	ENSG00000125148.6	ENSG00000125304.8
	ENSG00000128052.8	ENSG00000130723.13	ENSG00000131386.13	ENSG00000131981.11
	ENSG00000133114.13	ENSG00000133392.12	ENSG00000134508.8	ENSG00000134571.6
	ENSG00000134884.9	ENSG00000134982.12	ENSG00000135503.8	ENSG00000135775.9
	ENSG00000135945.5	ENSG00000137936.12	ENSG00000137941.12	ENSG00000138160.4
	ENSG00000138735.11	ENSG00000140395.4	ENSG00000142627.9	ENSG00000142910.11
	ENSG00000142949.12	ENSG00000144868.9	ENSG00000147421.13	ENSG00000148288.7
	ENSG00000148842.13	ENSG00000149634.4	ENSG00000152128.13	ENSG00000152556.11
	ENSG00000154122.8	ENSG00000154479.8	ENSG00000154589.2	ENSG00000155282.7
	ENSG00000155304.4	ENSG00000155666.7	ENSG00000155755.14	ENSG00000157014.6
	ENSG00000159189.7	ENSG00000160401.10	ENSG00000160741.12	ENSG00000162736.11
	ENSG00000162928.8	ENSG00000163563.7	ENSG00000163950.8	ENSG00000164620.4
	ENSG00000164742.10	ENSG00000164744.8	ENSG00000165029.11	ENSG00000165417.7
	ENSG00000165671.14	ENSG00000166340.10	ENSG00000166377.15	ENSG00000166704.7
	ENSG00000167487.7	ENSG00000167550.6	ENSG00000167972.9	ENSG00000168438.10
	ENSG00000168621.10	ENSG00000168658.14	ENSG00000169129.10	ENSG00000169964.5
	ENSG00000170037.9	ENSG00000170921.10	ENSG00000171219.8	ENSG00000171443.6
	ENSG00000172366.15	ENSG00000173868.7	ENSG00000174407.7	ENSG00000174473.11
	ENSG00000174799.6	ENSG00000176095.7	ENSG00000176771.11	ENSG00000177888.7
	ENSG00000178460.13	ENSG00000178826.6	ENSG00000180178.6	ENSG00000181982.13
	ENSG00000182108.5	ENSG00000182177.9	ENSG00000183576.8	ENSG00000184009.5
	ENSG00000184261.4	ENSG00000185028.3	ENSG00000185527.7	ENSG00000185532.10
	ENSG00000185955.4	ENSG00000186326.3	ENSG00000187013.2	ENSG00000187498.10
	ENSG00000187566.3	ENSG00000188242.4	ENSG00000196182.6	ENSG00000196581.6
	ENSG00000196754.6	ENSG00000196923.9	ENSG00000197121.10	ENSG00000197386.6
	ENSG00000197409.6	ENSG00000197471.7	ENSG00000197536.6	ENSG00000197776.3
	ENSG00000197930.8	ENSG00000197956.5	ENSG00000198373.8	ENSG00000198546.10
	ENSG00000198668.6	ENSG00000198728.6	ENSG00000198821.6	ENSG00000198925.6
	ENSG00000203999.4	ENSG00000204396.6	ENSG00000213225.6	ENSG00000213355.3
	ENSG00000213385.3	ENSG00000213551.4	ENSG00000215158.5	ENSG00000215187.5
	ENSG00000215481.4	ENSG00000216285.4	ENSG00000218016.2	ENSG00000221955.6
	ENSG00000224786.1	ENSG00000228223.1	ENSG00000228397.1	ENSG00000228817.3
	ENSG00000229420.1	ENSG00000230850.3	ENSG00000231485.1	ENSG00000231507.1
	ENSG00000232815.1	ENSG00000233996.1	ENSG00000235213.1	ENSG00000235957.1
	ENSG00000236136.1	ENSG00000236155.2	ENSG00000238102.1	ENSG00000240747.3
	ENSG00000243629.1	ENSG00000244411.2	ENSG00000248927.1	ENSG00000250635.1
	ENSG00000251410.1	ENSG00000251665.1	ENSG00000254305.1	ENSG00000254714.1
	ENSG00000254786.1	ENSG00000255010.1	ENSG00000256211.1	ENSG00000256540.1
	ENSG00000257464.1	ENSG00000258418.1	ENSG00000259584.1	ENSG00000260464.1
	ENSG00000261609.3	ENSG00000261787.1	ENSG00000261925.1	ENSG00000262454.1
	ENSG00000267093.1	ENSG00000267416.1	ENSG00000268573.1	ENSG00000268852.1
	ENSG00000269282.1	ENSG00000270039.1	ENSG00000270917.1	ENSG00000272053.1
	ENSG00000272108.1	ENSG00000272325.1	ENSG00000272347.1	ENSG00000272377.1
	ENSG00000272428.1	ENSG00000272942.1	ENSG00000273399.1	
	ENSG00000004975.7	ENSG00000005448.12	ENSG00000006704.6	ENSG00000008226.15
	ENSG00000010017.9	ENSG00000010282.10	ENSG00000018510.8	ENSG00000018625.10
	ENSG00000019991.11	ENSG00000047249.12	ENSG00000049541.6	ENSG00000058262.5
	ENSG00000063241.3	ENSG00000065328.12	ENSG00000068305.13	ENSG00000068383.14

Nerve Tibial

ENSG00000071082.6	ENSG00000074696.8	ENSG00000075223.9	ENSG00000076984.13
ENSG00000080031.5	ENSG00000091009.6	ENSG00000099139.9	ENSG00000100281.9
ENSG00000100312.6	ENSG00000100365.10	ENSG00000100767.11	ENSG00000101670.7
ENSG00000103064.9	ENSG00000103160.7	ENSG00000103522.11	ENSG00000104129.5
ENSG00000104951.11	ENSG00000104960.11	ENSG00000105792.15	ENSG00000106355.5
ENSG00000107341.4	ENSG00000107929.10	ENSG00000108387.10	ENSG00000108953.12
ENSG00000109066.9	ENSG00000109189.8	ENSG00000110651.7	ENSG00000111262.4
ENSG00000112175.6	ENSG00000112186.7	ENSG00000112297.10	ENSG00000112394.12
ENSG00000113161.11	ENSG00000113209.6	ENSG00000113758.9	ENSG00000113790.6
ENSG00000114902.9	ENSG00000115091.7	ENSG00000115163.10	ENSG00000115274.10
ENSG00000115353.6	ENSG00000116711.8	ENSG00000117620.8	ENSG00000117862.7
ENSG00000118733.12	ENSG00000118894.10	ENSG00000119636.11	ENSG00000119703.12
ENSG00000120686.7	ENSG00000120885.15	ENSG00000125743.6	ENSG00000125910.4
ENSG00000127743.5	ENSG00000130173.9	ENSG00000132405.14	ENSG00000132478.5
ENSG00000133114.13	ENSG00000133193.8	ENSG00000133943.16	ENSG00000134250.13
ENSG00000134508.8	ENSG00000134571.6	ENSG00000134884.9	ENSG00000135945.5
ENSG00000136160.10	ENSG00000136267.9	ENSG00000136943.6	ENSG00000137273.3
ENSG00000138160.4	ENSG00000138785.10	ENSG00000139684.9	ENSG00000140254.8
ENSG00000140280.9	ENSG00000140395.4	ENSG00000140968.6	ENSG00000141441.11
ENSG00000143740.10	ENSG00000143995.15	ENSG00000144868.9	ENSG00000145088.4
ENSG00000148737.11	ENSG00000148926.5	ENSG00000149212.6	ENSG00000149634.4
ENSG00000150527.12	ENSG00000151014.4	ENSG00000153094.17	ENSG00000153993.9
ENSG00000154262.8	ENSG00000155282.7	ENSG00000155666.7	ENSG00000155903.7
ENSG00000157657.10	ENSG00000157954.10	ENSG00000158055.11	ENSG00000158545.11
ENSG00000159189.7	ENSG00000159674.7	ENSG00000160218.8	ENSG00000160221.12
ENSG00000160602.9	ENSG00000161911.7	ENSG00000162434.7	ENSG00000162551.9
ENSG00000163106.6	ENSG00000163291.10	ENSG00000163449.6	ENSG00000164054.11
ENSG00000164087.3	ENSG00000164197.7	ENSG00000164620.4	ENSG00000165178.8
ENSG00000165487.9	ENSG00000166091.15	ENSG00000166340.10	ENSG00000166342.14
ENSG00000166471.6	ENSG00000166557.8	ENSG00000166704.7	ENSG00000166856.1
ENSG00000167281.14	ENSG00000167759.8	ENSG00000167985.2	ENSG00000168079.12
ENSG00000168152.8	ENSG00000168389.13	ENSG00000168658.14	ENSG00000168883.15
ENSG00000169129.10	ENSG00000169174.9	ENSG00000169403.7	ENSG00000169908.6
ENSG00000169955.6	ENSG00000169992.5	ENSG00000170989.8	ENSG00000171219.8
ENSG00000171735.14	ENSG00000172005.6	ENSG00000172037.9	ENSG00000172243.13
ENSG00000172845.9	ENSG00000174576.4	ENSG00000174799.6	ENSG00000174827.9
ENSG00000176055.9	ENSG00000176153.10	ENSG00000177888.7	ENSG00000178031.11
ENSG00000178789.4	ENSG00000180008.8	ENSG00000180178.6	ENSG00000180801.11
ENSG00000181982.13	ENSG00000182902.9	ENSG00000183055.5	ENSG00000183576.8
ENSG00000183801.3	ENSG00000184009.5	ENSG00000184451.5	ENSG00000184786.4
ENSG00000185022.7	ENSG00000186188.6	ENSG00000186197.8	ENSG00000186517.9
ENSG00000186998.11	ENSG00000187498.10	ENSG00000187566.3	ENSG00000187742.10
ENSG00000187944.2	ENSG00000196218.7	ENSG00000196453.7	ENSG00000196639.6
ENSG00000197702.7	ENSG00000197763.8	ENSG00000197838.4	ENSG00000198081.6
ENSG00000198198.9	ENSG00000198517.5	ENSG00000204963.4	ENSG00000205302.2
ENSG00000205629.7	ENSG00000213079.5	ENSG00000213197.3	ENSG00000213225.6
ENSG00000213551.4	ENSG00000214146.2	ENSG00000214362.2	ENSG00000214413.3
ENSG00000214510.5	ENSG00000214561.3	ENSG00000215158.5	ENSG00000216895.4
ENSG00000224795.1	ENSG00000224858.4	ENSG00000224956.5	ENSG00000225137.1

	ENSG00000225930.3	ENSG00000227063.4	ENSG00000227477.1	ENSG00000227999.1
	ENSG00000228008.1	ENSG00000228397.1	ENSG00000228522.2	ENSG00000229291.1
	ENSG00000229605.4	ENSG00000229809.4	ENSG00000230850.3	ENSG00000231322.1
	ENSG00000231485.1	ENSG00000231690.2	ENSG00000232453.1	ENSG00000233077.1
	ENSG00000233469.1	ENSG00000233610.1	ENSG00000234065.2	ENSG00000234383.1
	ENSG00000234722.3	ENSG00000234862.1	ENSG00000235253.1	ENSG00000235373.1
	ENSG00000236136.1	ENSG00000236184.1	ENSG00000239486.1	ENSG00000240132.1
	ENSG00000240350.1	ENSG00000240747.3	ENSG00000243300.2	ENSG00000244171.3
	ENSG00000246548.3	ENSG00000248916.1	ENSG00000249430.1	ENSG00000249767.1
	ENSG00000253304.1	ENSG00000253328.2	ENSG00000254332.1	ENSG00000255277.2
	ENSG00000255561.2	ENSG00000255735.1	ENSG00000256167.1	ENSG00000256361.1
	ENSG00000256913.1	ENSG00000257464.1	ENSG00000257501.2	ENSG00000258811.1
	ENSG00000259158.1	ENSG00000259404.1	ENSG00000260464.1	ENSG00000263105.1
	ENSG00000264016.2	ENSG00000266498.1	ENSG00000266651.1	ENSG00000267289.1
	ENSG00000267316.1	ENSG00000267319.1	ENSG00000267416.1	ENSG00000267957.1
	ENSG00000268087.1	ENSG00000268172.1	ENSG00000268734.1	ENSG00000269510.1
	ENSG00000269543.1	ENSG00000271856.1	ENSG00000272108.1	ENSG00000272235.1
	ENSG00000272347.1	ENSG00000272384.1	ENSG00000272425.1	ENSG00000272523.1
	ENSG00000272682.1	ENSG00000272908.1		
**Pancreas**	ENSG00000010282.10	ENSG00000047249.12	ENSG00000050426.11	ENSG00000058668.10
	ENSG00000061794.8	ENSG00000064201.11	ENSG00000065154.7	ENSG00000065328.12
	ENSG00000071082.6	ENSG00000073067.9	ENSG00000074696.8	ENSG00000089250.14
	ENSG00000094916.9	ENSG00000099139.9	ENSG00000100938.13	ENSG00000101670.7
	ENSG00000103064.9	ENSG00000103160.7	ENSG00000104408.5	ENSG00000105647.10
	ENSG00000105792.15	ENSG00000105974.7	ENSG00000108773.6	ENSG00000109066.9
	ENSG00000109684.10	ENSG00000109971.9	ENSG00000111275.8	ENSG00000113161.11
	ENSG00000113263.8	ENSG00000113384.9	ENSG00000115274.10	ENSG00000116752.5
	ENSG00000117143.9	ENSG00000118518.11	ENSG00000119514.5	ENSG00000119703.12
	ENSG00000122435.5	ENSG00000122497.13	ENSG00000126368.5	ENSG00000129048.6
	ENSG00000130818.6	ENSG00000133110.10	ENSG00000134460.11	ENSG00000134508.8
	ENSG00000134884.9	ENSG00000135094.6	ENSG00000135898.5	ENSG00000135945.5
	ENSG00000136267.9	ENSG00000137941.12	ENSG00000138785.10	ENSG00000140280.9
	ENSG00000140740.6	ENSG00000144868.9	ENSG00000145088.4	ENSG00000149591.12
	ENSG00000149634.4	ENSG00000151014.4	ENSG00000152128.13	ENSG00000154479.8
	ENSG00000155666.7	ENSG00000156170.8	ENSG00000159189.7	ENSG00000161970.8
	ENSG00000162434.7	ENSG00000163075.8	ENSG00000163508.8	ENSG00000163739.4
	ENSG00000164744.8	ENSG00000165417.7	ENSG00000166091.15	ENSG00000166340.10
	ENSG00000167862.5	ENSG00000168564.5	ENSG00000169964.5	ENSG00000171219.8
	ENSG00000173175.10	ENSG00000173557.10	ENSG00000173801.12	ENSG00000174799.6
	ENSG00000175029.12	ENSG00000175606.6	ENSG00000177888.7	ENSG00000178764.6
	ENSG00000179387.5	ENSG00000179935.5	ENSG00000180178.6	ENSG00000180801.11
	ENSG00000181982.13	ENSG00000183576.8	ENSG00000184451.5	ENSG00000186197.8
	ENSG00000186660.14	ENSG00000187017.10	ENSG00000187498.10	ENSG00000188060.6
	ENSG00000188242.4	ENSG00000196754.6	ENSG00000197409.6	ENSG00000198668.6
	ENSG00000204371.7	ENSG00000205882.4	ENSG00000213225.6	ENSG00000213700.3
	ENSG00000214146.2	ENSG00000214268.2	ENSG00000215187.5	ENSG00000221869.4
	ENSG00000224261.2	ENSG00000224914.2	ENSG00000226055.2	ENSG00000226852.2
	ENSG00000228223.1	ENSG00000228397.1	ENSG00000228817.3	ENSG00000230850.3
	ENSG00000232040.2	ENSG00000233337.1	ENSG00000234284.2	ENSG00000236136.1

	ENSG00000237821.1	ENSG00000239306.4	ENSG00000240747.3	ENSG00000243449.2
	ENSG00000247627.2	ENSG00000248487.4	ENSG00000248771.1	ENSG00000248927.1
	ENSG00000250635.1	ENSG00000253390.1	ENSG00000253942.1	ENSG00000254714.1
	ENSG00000256540.1	ENSG00000257464.1	ENSG00000258418.1	ENSG00000258792.1
	ENSG00000258884.1	ENSG00000258919.1	ENSG00000259203.1	ENSG00000261621.1
	ENSG00000265817.1	ENSG00000267132.1	ENSG00000267259.1	ENSG00000267416.1
	ENSG00000270917.1	ENSG00000272942.1		
**Pituitary**	ENSG00000005884.13	ENSG00000010282.10	ENSG00000011143.12	ENSG00000023734.6
	ENSG00000047249.12	ENSG00000051128.14	ENSG00000056487.11	ENSG00000058668.10
	ENSG00000074696.8	ENSG00000075223.9	ENSG00000082269.12	ENSG00000089159.11
	ENSG00000089250.14	ENSG00000100365.10	ENSG00000100479.8	ENSG00000100938.13
	ENSG00000103160.7	ENSG00000103522.11	ENSG00000104408.5	ENSG00000109066.9
	ENSG00000112312.5	ENSG00000113161.11	ENSG00000114019.10	ENSG00000118733.12
	ENSG00000125910.4	ENSG00000132326.7	ENSG00000133943.16	ENSG00000134571.6
	ENSG00000135898.5	ENSG00000136943.6	ENSG00000138709.13	ENSG00000139926.11
	ENSG00000140395.4	ENSG00000143179.8	ENSG00000145107.11	ENSG00000145451.8
	ENSG00000149634.4	ENSG00000153162.8	ENSG00000153815.12	ENSG00000154316.10
	ENSG00000156973.9	ENSG00000157851.12	ENSG00000159189.7	ENSG00000159674.7
	ENSG00000160218.8	ENSG00000160602.9	ENSG00000160908.14	ENSG00000161911.7
	ENSG00000163617.6	ENSG00000164675.6	ENSG00000164744.8	ENSG00000165417.7
	ENSG00000166091.15	ENSG00000168658.14	ENSG00000169194.5	ENSG00000169992.5
	ENSG00000170921.10	ENSG00000171777.11	ENSG00000174744.9	ENSG00000174799.6
	ENSG00000176095.7	ENSG00000176444.14	ENSG00000176956.8	ENSG00000181982.13
	ENSG00000182175.9	ENSG00000183576.8	ENSG00000183814.11	ENSG00000183921.5
	ENSG00000184009.5	ENSG00000185532.10	ENSG00000187498.10	ENSG00000187889.8
	ENSG00000189409.8	ENSG00000196116.6	ENSG00000196923.9	ENSG00000197409.6
	ENSG00000198821.6	ENSG00000198842.5	ENSG00000204150.7	ENSG00000213111.4
	ENSG00000213225.6	ENSG00000213355.3	ENSG00000213551.4	ENSG00000213930.7
	ENSG00000214146.2	ENSG00000215187.5	ENSG00000217241.1	ENSG00000218175.2
	ENSG00000220575.3	ENSG00000223601.2	ENSG00000224914.2	ENSG00000225333.5
	ENSG00000227071.1	ENSG00000228397.1	ENSG00000230330.1	ENSG00000230850.3
	ENSG00000231720.1	ENSG00000232810.3	ENSG00000233469.1	ENSG00000234076.1
	ENSG00000234104.1	ENSG00000235349.1	ENSG00000235373.1	ENSG00000236136.1
	ENSG00000240747.3	ENSG00000241954.1	ENSG00000243629.1	ENSG00000248243.1
	ENSG00000248487.4	ENSG00000249621.1	ENSG00000250635.1	ENSG00000254786.1
	ENSG00000255162.1	ENSG00000257231.1	ENSG00000257464.1	ENSG00000258256.1
	ENSG00000258418.1	ENSG00000258884.1	ENSG00000259404.1	ENSG00000261787.1
	ENSG00000262098.1	ENSG00000263146.2	ENSG00000264016.2	ENSG00000264359.1
	ENSG00000267416.1	ENSG00000269895.1	ENSG00000270750.1	ENSG00000270917.1
	ENSG00000272108.1	ENSG00000272942.1	ENSG00000272966.1	ENSG00000273325.1
**Skin Not Sun**	ENSG00000002587.5	ENSG00000004866.14	ENSG00000004975.7	ENSG00000005448.12
	ENSG00000005700.10	ENSG00000005961.13	ENSG00000006704.6	ENSG00000010282.10
	ENSG00000010932.11	ENSG00000038427.11	ENSG00000047249.12	ENSG00000049541.6
	ENSG00000063169.6	ENSG00000066468.16	ENSG00000068383.14	ENSG00000072135.8
	ENSG00000072201.9	ENSG00000074696.8	ENSG00000075223.9	ENSG00000078081.3
	ENSG00000081052.10	ENSG00000085274.11	ENSG00000091436.12	ENSG00000099385.7
	ENSG00000100246.8	ENSG00000100281.9	ENSG00000100365.10	ENSG00000100938.13
	ENSG00000101624.6	ENSG00000101670.7	ENSG00000103042.4	ENSG00000103043.10
	ENSG00000103502.9	ENSG00000104853.11	ENSG00000104951.11	ENSG00000104960.11

ENSG00000105352.6	ENSG00000105366.11	ENSG00000105792.15	ENSG00000106261.12
ENSG00000107331.12	ENSG00000108094.10	ENSG00000108953.12	ENSG00000109066.9
ENSG00000109458.4	ENSG00000110011.9	ENSG00000110104.7	ENSG00000110200.4
ENSG00000111729.8	ENSG00000112146.12	ENSG00000113161.11	ENSG00000113758.9
ENSG00000114019.10	ENSG00000114353.12	ENSG00000115286.15	ENSG00000116337.11
ENSG00000116544.7	ENSG00000117862.7	ENSG00000118496.4	ENSG00000118520.9
ENSG00000118894.10	ENSG00000120093.7	ENSG00000120471.10	ENSG00000121274.8
ENSG00000125846.11	ENSG00000125910.4	ENSG00000127743.5	ENSG00000128512.15
ENSG00000130635.11	ENSG00000132405.14	ENSG00000133114.13	ENSG00000133265.6
ENSG00000133943.16	ENSG00000134250.13	ENSG00000134571.6	ENSG00000135775.9
ENSG00000135801.5	ENSG00000136160.10	ENSG00000136267.9	ENSG00000138160.4
ENSG00000139684.9	ENSG00000139990.13	ENSG00000140254.8	ENSG00000140280.9
ENSG00000140577.11	ENSG00000141380.9	ENSG00000144868.9	ENSG00000145088.4
ENSG00000145700.5	ENSG00000148288.7	ENSG00000148356.9	ENSG00000148926.5
ENSG00000149634.4	ENSG00000149636.11	ENSG00000151014.4	ENSG00000151067.16
ENSG00000151502.6	ENSG00000152128.13	ENSG00000152620.8	ENSG00000154122.8
ENSG00000154654.10	ENSG00000155666.7	ENSG00000155755.14	ENSG00000156026.10
ENSG00000156639.7	ENSG00000156689.2	ENSG00000156804.3	ENSG00000157734.9
ENSG00000158055.11	ENSG00000159189.7	ENSG00000160049.7	ENSG00000160224.12
ENSG00000160602.9	ENSG00000162434.7	ENSG00000164054.11	ENSG00000164099.3
ENSG00000164620.4	ENSG00000164675.6	ENSG00000165417.7	ENSG00000165487.9
ENSG00000166091.15	ENSG00000166340.10	ENSG00000166557.8	ENSG00000166704.7
ENSG00000166710.13	ENSG00000166840.9	ENSG00000167674.10	ENSG00000167757.9
ENSG00000167759.8	ENSG00000168658.14	ENSG00000168876.4	ENSG00000169129.10
ENSG00000169397.3	ENSG00000169908.6	ENSG00000172243.13	ENSG00000172663.4
ENSG00000172752.10	ENSG00000172830.8	ENSG00000172893.11	ENSG00000174156.9
ENSG00000174799.6	ENSG00000174946.5	ENSG00000175029.12	ENSG00000176095.7
ENSG00000176153.10	ENSG00000176697.14	ENSG00000177311.6	ENSG00000177888.7
ENSG00000178982.5	ENSG00000180178.6	ENSG00000181744.4	ENSG00000181778.4
ENSG00000181982.13	ENSG00000182366.5	ENSG00000183055.5	ENSG00000183273.2
ENSG00000183474.11	ENSG00000183576.8	ENSG00000184682.5	ENSG00000185069.2
ENSG00000185567.6	ENSG00000186197.8	ENSG00000187017.10	ENSG00000187498.10
ENSG00000187566.3	ENSG00000188242.4	ENSG00000189057.6	ENSG00000189292.11
ENSG00000196639.6	ENSG00000196776.10	ENSG00000197153.3	ENSG00000197647.7
ENSG00000197905.4	ENSG00000197930.8	ENSG00000198454.2	ENSG00000198517.5
ENSG00000198618.4	ENSG00000203688.4	ENSG00000203999.4	ENSG00000205209.3
ENSG00000205629.7	ENSG00000213225.6	ENSG00000213376.4	ENSG00000213551.4
ENSG00000214362.2	ENSG00000215187.5	ENSG00000224207.2	ENSG00000224271.1
ENSG00000224543.3	ENSG00000225056.1	ENSG00000226005.3	ENSG00000227096.1
ENSG00000228223.1	ENSG00000228397.1	ENSG00000228559.1	ENSG00000229291.1
ENSG00000230850.3	ENSG00000231485.1	ENSG00000231684.2	ENSG00000232833.4
ENSG00000233895.1	ENSG00000233901.1	ENSG00000234797.4	ENSG00000234862.1
ENSG00000235328.1	ENSG00000235725.1	ENSG00000236136.1	ENSG00000237611.1
ENSG00000237821.1	ENSG00000239789.1	ENSG00000240021.5	ENSG00000242198.1
ENSG00000243300.2	ENSG00000243672.1	ENSG00000243802.2	ENSG00000244134.1
ENSG00000246922.4	ENSG00000248564.1	ENSG00000248626.1	ENSG00000250726.1
ENSG00000250853.1	ENSG00000253304.1	ENSG00000254290.1	ENSG00000254332.1
ENSG00000254610.1	ENSG00000255624.1	ENSG00000256167.1	ENSG00000256540.1
ENSG00000256875.2	ENSG00000257464.1	ENSG00000258404.1	ENSG00000259158.1

	ENSG00000259517.1	ENSG00000260192.1	ENSG00000260455.1	ENSG00000261005.1
	ENSG00000261353.1	ENSG00000262663.1	ENSG00000266733.1	ENSG00000266946.1
	ENSG00000267010.1	ENSG00000267289.1	ENSG00000268087.1	ENSG00000269119.1
	ENSG00000269421.1	ENSG00000271020.1	ENSG00000271856.1	ENSG00000272024.1
	ENSG00000272087.1	ENSG00000272347.1	ENSG00000272377.1	ENSG00000272425.1
	ENSG00000272520.1	ENSG00000272682.1	ENSG00000272848.1	ENSG00000272931.1
	ENSG00000272942.1	ENSG00000272966.1		
**Skin Sun Exposed**	ENSG00000002587.5	ENSG00000004975.7	ENSG00000005448.12	ENSG00000005961.13
	ENSG00000006704.6	ENSG00000008394.8	ENSG00000010282.10	ENSG00000010319.2
	ENSG00000034510.4	ENSG00000047249.12	ENSG00000058668.10	ENSG00000063169.6
	ENSG00000063241.3	ENSG00000065154.7	ENSG00000072201.9	ENSG00000074696.8
	ENSG00000075223.9	ENSG00000075886.10	ENSG00000076351.8	ENSG00000084070.7
	ENSG00000085274.11	ENSG00000085377.9	ENSG00000086475.10	ENSG00000099139.9
	ENSG00000099246.12	ENSG00000099385.7	ENSG00000100246.8	ENSG00000100281.9
	ENSG00000100365.10	ENSG00000100938.13	ENSG00000101670.7	ENSG00000104221.8
	ENSG00000104853.11	ENSG00000104951.11	ENSG00000104960.11	ENSG00000105792.15
	ENSG00000105948.9	ENSG00000106588.6	ENSG00000106948.12	ENSG00000108094.10
	ENSG00000108953.12	ENSG00000109066.9	ENSG00000109787.8	ENSG00000110200.4
	ENSG00000111729.8	ENSG00000112146.12	ENSG00000112297.10	ENSG00000112394.12
	ENSG00000113161.11	ENSG00000114902.9	ENSG00000115091.7	ENSG00000115274.10
	ENSG00000115504.10	ENSG00000116544.7	ENSG00000117448.9	ENSG00000117862.7
	ENSG00000118496.4	ENSG00000118520.9	ENSG00000118557.11	ENSG00000118680.8
	ENSG00000118894.10	ENSG00000118985.10	ENSG00000120093.7	ENSG00000120129.5
	ENSG00000120686.7	ENSG00000121335.10	ENSG00000121900.14	ENSG00000125846.11
	ENSG00000125910.4	ENSG00000126107.10	ENSG00000126653.11	ENSG00000127743.5
	ENSG00000129071.5	ENSG00000129460.11	ENSG00000129473.5	ENSG00000130173.9
	ENSG00000132405.14	ENSG00000132613.10	ENSG00000132854.14	ENSG00000133114.13
	ENSG00000133943.16	ENSG00000134250.13	ENSG00000134571.6	ENSG00000135324.5
	ENSG00000135775.9	ENSG00000135801.5	ENSG00000135945.5	ENSG00000136160.10
	ENSG00000136267.9	ENSG00000138160.4	ENSG00000138785.10	ENSG00000139684.9
	ENSG00000140254.8	ENSG00000140280.9	ENSG00000140835.8	ENSG00000141522.7
	ENSG00000142609.13	ENSG00000143140.6	ENSG00000143157.7	ENSG00000143546.5
	ENSG00000143740.10	ENSG00000144395.13	ENSG00000144868.9	ENSG00000145088.4
	ENSG00000145700.5	ENSG00000147573.12	ENSG00000147687.12	ENSG00000148356.9
	ENSG00000148926.5	ENSG00000149634.4	ENSG00000150459.8	ENSG00000151014.4
	ENSG00000151067.16	ENSG00000151500.10	ENSG00000151502.6	ENSG00000152193.7
	ENSG00000152620.8	ENSG00000153094.17	ENSG00000153575.6	ENSG00000154122.8
	ENSG00000154654.10	ENSG00000154734.10	ENSG00000155666.7	ENSG00000155755.14
	ENSG00000156026.10	ENSG00000158055.11	ENSG00000159189.7	ENSG00000160049.7
	ENSG00000160050.10	ENSG00000160224.12	ENSG00000160602.9	ENSG00000162434.7
	ENSG00000162777.12	ENSG00000162888.4	ENSG00000163145.8	ENSG00000163319.6
	ENSG00000163347.5	ENSG00000163449.6	ENSG00000164054.11	ENSG00000164087.3
	ENSG00000164099.3	ENSG00000164620.4	ENSG00000164675.6	ENSG00000165131.6
	ENSG00000165181.12	ENSG00000165417.7	ENSG00000165480.11	ENSG00000166091.15
	ENSG00000166340.10	ENSG00000166342.14	ENSG00000166557.8	ENSG00000166704.7
	ENSG00000166840.9	ENSG00000167281.14	ENSG00000167550.6	ENSG00000167759.8
	ENSG00000168158.2	ENSG00000168658.14	ENSG00000169129.10	ENSG00000169251.8
	ENSG00000169397.3	ENSG00000169599.8	ENSG00000169855.15	ENSG00000169908.6
	ENSG00000170006.7	ENSG00000170899.6	ENSG00000171219.8	ENSG00000171560.10

	ENSG00000171606.13	ENSG00000172005.6	ENSG00000172663.4	ENSG00000172893.11
	ENSG00000174744.9	ENSG00000174799.6	ENSG00000175029.12	ENSG00000175426.6
	ENSG00000176095.7	ENSG00000176153.10	ENSG00000177191.2	ENSG00000177888.7
	ENSG00000178982.5	ENSG00000179941.6	ENSG00000180178.6	ENSG00000181982.13
	ENSG00000182077.6	ENSG00000182175.9	ENSG00000182742.5	ENSG00000183055.5
	ENSG00000183273.2	ENSG00000183576.8	ENSG00000183729.3	ENSG00000183801.3
	ENSG00000184009.5	ENSG00000184451.5	ENSG00000185069.2	ENSG00000186197.8
	ENSG00000186790.4	ENSG00000187017.10	ENSG00000187498.10	ENSG00000187566.3
	ENSG00000188100.8	ENSG00000188242.4	ENSG00000189057.6	ENSG00000189292.11
	ENSG00000196083.5	ENSG00000196581.6	ENSG00000196639.6	ENSG00000196776.10
	ENSG00000197153.3	ENSG00000197409.6	ENSG00000197647.7	ENSG00000197757.7
	ENSG00000197776.3	ENSG00000197859.5	ENSG00000198198.9	ENSG00000198300.8
	ENSG00000198454.2	ENSG00000198517.5	ENSG00000198668.6	ENSG00000205629.7
	ENSG00000211460.7	ENSG00000213225.6	ENSG00000213376.4	ENSG00000213551.4
	ENSG00000213970.3	ENSG00000214013.5	ENSG00000214146.2	ENSG00000214362.2
	ENSG00000215187.5	ENSG00000223823.1	ENSG00000224271.1	ENSG00000224956.5
	ENSG00000225663.3	ENSG00000225726.1	ENSG00000225930.3	ENSG00000226243.1
	ENSG00000227096.1	ENSG00000228223.1	ENSG00000228397.1	ENSG00000228559.1
	ENSG00000229291.1	ENSG00000230850.3	ENSG00000231485.1	ENSG00000231684.2
	ENSG00000232699.2	ENSG00000232833.4	ENSG00000233895.1	ENSG00000233901.1
	ENSG00000234383.1	ENSG00000234536.1	ENSG00000234709.2	ENSG00000234797.4
	ENSG00000234862.1	ENSG00000235725.1	ENSG00000236136.1	ENSG00000237821.1
	ENSG00000240021.5	ENSG00000243300.2	ENSG00000243802.2	ENSG00000244405.3
	ENSG00000245330.4	ENSG00000248334.2	ENSG00000248916.1	ENSG00000253304.1
	ENSG00000254332.1	ENSG00000254618.1	ENSG00000254786.1	ENSG00000255277.2
	ENSG00000255561.2	ENSG00000255624.1	ENSG00000256167.1	ENSG00000256361.1
	ENSG00000256875.2	ENSG00000257243.1	ENSG00000257464.1	ENSG00000257515.1
	ENSG00000258404.1	ENSG00000258811.1	ENSG00000259158.1	ENSG00000259404.1
	ENSG00000259517.1	ENSG00000260469.1	ENSG00000260673.1	ENSG00000261005.1
	ENSG00000264016.2	ENSG00000266498.1	ENSG00000266651.1	ENSG00000266946.1
	ENSG00000267416.1	ENSG00000268087.1	ENSG00000269510.1	ENSG00000271856.1
	ENSG00000272024.1	ENSG00000272108.1	ENSG00000272235.1	ENSG00000272347.1
	ENSG00000272354.1	ENSG00000272377.1	ENSG00000272425.1	ENSG00000272520.1
	ENSG00000272523.1	ENSG00000272682.1	ENSG00000272829.1	ENSG00000272942.1
	ENSG00000272966.1			
**Small Intestine**	ENSG00000011243.13	ENSG00000047249.12	ENSG00000048649.9	ENSG00000051128.14
	ENSG00000074696.8	ENSG00000092470.7	ENSG00000094916.9	ENSG00000100410.3
	ENSG00000103064.9	ENSG00000109066.9	ENSG00000113161.11	ENSG00000113384.9
	ENSG00000114648.7	ENSG00000114902.9	ENSG00000130201.3	ENSG00000133943.16
	ENSG00000140280.9	ENSG00000144868.9	ENSG00000149634.4	ENSG00000149929.11
	ENSG00000151023.12	ENSG00000156853.7	ENSG00000159189.7	ENSG00000162949.12
	ENSG00000164744.8	ENSG00000165417.7	ENSG00000166340.10	ENSG00000170899.6
	ENSG00000171403.5	ENSG00000172366.15	ENSG00000174799.6	ENSG00000176371.9
	ENSG00000177954.7	ENSG00000178982.5	ENSG00000179869.10	ENSG00000180801.11
	ENSG00000181982.13	ENSG00000186105.7	ENSG00000187498.10	ENSG00000196331.5
	ENSG00000196642.11	ENSG00000197409.6	ENSG00000198604.6	ENSG00000203614.3
	ENSG00000204544.5	ENSG00000213212.3	ENSG00000214146.2	ENSG00000224914.2
	ENSG00000227920.2	ENSG00000228397.1	ENSG00000229291.1	ENSG00000229859.4
	ENSG00000230330.1	ENSG00000230850.3	ENSG00000232040.2	ENSG00000232150.3

	ENSG00000232815.1	ENSG00000232837.1	ENSG00000235145.2	ENSG00000236136.1
	ENSG00000250215.1	ENSG00000250635.1	ENSG00000254122.1	ENSG00000258418.1
	ENSG00000259404.1	ENSG00000260781.1	ENSG00000267416.1	ENSG00000270689.1
	ENSG00000271711.1	ENSG00000272824.1	ENSG00000272942.1	
**Spleen**	ENSG00000010282.10	ENSG00000013375.11	ENSG00000024526.12	ENSG00000058056.4
	ENSG00000058668.10	ENSG00000073737.12	ENSG00000074696.8	ENSG00000075223.9
	ENSG00000085741.8	ENSG00000092871.12	ENSG00000100142.10	ENSG00000100276.9
	ENSG00000103064.9	ENSG00000103160.7	ENSG00000104131.8	ENSG00000106948.12
	ENSG00000106952.3	ENSG00000109066.9	ENSG00000113161.11	ENSG00000115415.14
	ENSG00000116586.7	ENSG00000118496.4	ENSG00000121892.10	ENSG00000122497.13
	ENSG00000124120.6	ENSG00000124491.11	ENSG00000125817.7	ENSG00000125910.4
	ENSG00000128052.8	ENSG00000128266.7	ENSG00000129559.8	ENSG00000130584.6
	ENSG00000134571.6	ENSG00000135775.9	ENSG00000135945.5	ENSG00000136634.5
	ENSG00000138081.15	ENSG00000138785.10	ENSG00000142687.13	ENSG00000142910.11
	ENSG00000145088.4	ENSG00000145107.11	ENSG00000147689.12	ENSG00000148356.9
	ENSG00000149634.4	ENSG00000150625.12	ENSG00000151014.4	ENSG00000155282.7
	ENSG00000155313.11	ENSG00000155666.7	ENSG00000156170.8	ENSG00000157014.6
	ENSG00000158748.3	ENSG00000159189.7	ENSG00000159674.7	ENSG00000162409.6
	ENSG00000162888.4	ENSG00000163319.6	ENSG00000164074.10	ENSG00000164742.10
	ENSG00000164744.8	ENSG00000164897.8	ENSG00000165066.11	ENSG00000165417.7
	ENSG00000166091.15	ENSG00000166340.10	ENSG00000166704.7	ENSG00000166855.5
	ENSG00000167862.5	ENSG00000168079.12	ENSG00000168658.14	ENSG00000170579.10
	ENSG00000170584.6	ENSG00000170921.10	ENSG00000174799.6	ENSG00000175029.12
	ENSG00000175895.3	ENSG00000176533.8	ENSG00000178741.7	ENSG00000178826.6
	ENSG00000178982.5	ENSG00000179580.5	ENSG00000180178.6	ENSG00000180801.11
	ENSG00000181982.13	ENSG00000183576.8	ENSG00000183578.5	ENSG00000187742.10
	ENSG00000189164.10	ENSG00000196104.6	ENSG00000196331.5	ENSG00000196754.6
	ENSG00000197409.6	ENSG00000197769.5	ENSG00000197961.7	ENSG00000198134.2
	ENSG00000198668.6	ENSG00000204794.6	ENSG00000213225.6	ENSG00000214146.2
	ENSG00000224003.1	ENSG00000224565.1	ENSG00000228000.1	ENSG00000228223.1
	ENSG00000228238.1	ENSG00000228397.1	ENSG00000229859.4	ENSG00000230185.4
	ENSG00000230850.3	ENSG00000231184.2	ENSG00000232833.4	ENSG00000234797.4
	ENSG00000236047.1	ENSG00000236136.1	ENSG00000236155.2	ENSG00000241954.1
	ENSG00000242960.1	ENSG00000243627.4	ENSG00000248208.1	ENSG00000248487.4
	ENSG00000249715.5	ENSG00000250635.1	ENSG00000254786.1	ENSG00000256276.1
	ENSG00000256540.1	ENSG00000257061.1	ENSG00000257464.1	ENSG00000258780.1
	ENSG00000260747.1	ENSG00000266604.1	ENSG00000267353.1	ENSG00000267416.1
	ENSG00000269490.1	ENSG00000269781.1	ENSG00000271511.1	ENSG00000271856.1
	ENSG00000271914.1	ENSG00000272942.1		
**Stomach**	ENSG00000010282.10	ENSG00000023734.6	ENSG00000031003.6	ENSG00000049246.10
	ENSG00000074696.8	ENSG00000086065.9	ENSG00000101057.11	ENSG00000101670.7
	ENSG00000108094.10	ENSG00000108953.12	ENSG00000109066.9	ENSG00000113161.11
	ENSG00000115274.10	ENSG00000116711.8	ENSG00000118985.10	ENSG00000119514.5
	ENSG00000119685.15	ENSG00000120093.7	ENSG00000129173.8	ENSG00000130173.9
	ENSG00000130717.8	ENSG00000134571.6	ENSG00000135898.5	ENSG00000138785.10
	ENSG00000140835.8	ENSG00000140968.6	ENSG00000144868.9	ENSG00000149634.4
	ENSG00000152128.13	ENSG00000152977.5	ENSG00000155980.7	ENSG00000157227.8
	ENSG00000159189.7	ENSG00000162065.7	ENSG00000162434.7	ENSG00000163449.6
	ENSG00000163563.7	ENSG00000165417.7	ENSG00000166091.15	ENSG00000166831.4

	ENSG00000168658.14	ENSG00000169733.7	ENSG00000173281.4	ENSG00000174744.9
	ENSG00000174799.6	ENSG00000175029.12	ENSG00000177599.8	ENSG00000177943.9
	ENSG00000181982.13	ENSG00000183576.8	ENSG00000188404.4	ENSG00000197409.6
	ENSG00000197471.7	ENSG00000198198.9	ENSG00000198553.4	ENSG00000204544.5
	ENSG00000204872.2	ENSG00000205209.3	ENSG00000224221.1	ENSG00000226101.1
	ENSG00000228223.1	ENSG00000228397.1	ENSG00000230832.3	ENSG00000230850.3
	ENSG00000231169.3	ENSG00000236136.1	ENSG00000240747.3	ENSG00000240751.1
	ENSG00000248487.4	ENSG00000250635.1	ENSG00000253390.1	ENSG00000255864.1
	ENSG00000256162.2	ENSG00000257464.1	ENSG00000262194.1	ENSG00000265369.2
	ENSG00000267416.1	ENSG00000269165.1	ENSG00000270427.1	ENSG00000272048.1
	ENSG00000272235.1	ENSG00000272885.1	ENSG00000272942.1	
**Thyroid**	ENSG00000004975.7	ENSG00000005189.15	ENSG00000005194.10	ENSG00000005448.12
	ENSG00000006210.6	ENSG00000006704.6	ENSG00000010282.10	ENSG00000025796.9
	ENSG00000031823.10	ENSG00000047249.12	ENSG00000058262.5	ENSG00000058668.10
	ENSG00000063241.3	ENSG00000068305.13	ENSG00000068383.14	ENSG00000070985.9
	ENSG00000072135.8	ENSG00000074696.8	ENSG00000076984.13	ENSG00000077147.10
	ENSG00000080200.5	ENSG00000085415.11	ENSG00000086065.9	ENSG00000086589.7
	ENSG00000087303.12	ENSG00000092470.7	ENSG00000100220.7	ENSG00000100281.9
	ENSG00000100365.10	ENSG00000103160.7	ENSG00000104129.5	ENSG00000108387.10
	ENSG00000108953.12	ENSG00000109066.9	ENSG00000109819.4	ENSG00000110955.4
	ENSG00000111203.7	ENSG00000112186.7	ENSG00000112378.11	ENSG00000112394.12
	ENSG00000112739.12	ENSG00000113161.11	ENSG00000113209.6	ENSG00000113790.6
	ENSG00000115274.10	ENSG00000116171.12	ENSG00000116299.12	ENSG00000117862.7
	ENSG00000118733.12	ENSG00000118894.10	ENSG00000119636.11	ENSG00000120093.7
	ENSG00000120686.7	ENSG00000125037.8	ENSG00000125817.7	ENSG00000127743.5
	ENSG00000128578.5	ENSG00000129173.8	ENSG00000129559.8	ENSG00000130173.9
	ENSG00000130584.6	ENSG00000132661.3	ENSG00000133114.13	ENSG00000133193.8
	ENSG00000134250.13	ENSG00000134508.8	ENSG00000134571.6	ENSG00000134817.9
	ENSG00000135503.8	ENSG00000135775.9	ENSG00000135823.9	ENSG00000135945.5
	ENSG00000136160.10	ENSG00000136943.6	ENSG00000138160.4	ENSG00000139684.9
	ENSG00000140280.9	ENSG00000140395.4	ENSG00000143157.7	ENSG00000143314.8
	ENSG00000143401.10	ENSG00000143740.10	ENSG00000144868.9	ENSG00000147689.12
	ENSG00000148356.9	ENSG00000148926.5	ENSG00000151500.10	ENSG00000151502.6
	ENSG00000152022.7	ENSG00000153094.17	ENSG00000153993.9	ENSG00000155304.4
	ENSG00000155666.7	ENSG00000158055.11	ENSG00000158545.11	ENSG00000159189.7
	ENSG00000159674.7	ENSG00000160218.8	ENSG00000160221.12	ENSG00000160602.9
	ENSG00000161911.7	ENSG00000162434.7	ENSG00000163106.6	ENSG00000163449.6
	ENSG00000164054.11	ENSG00000164099.3	ENSG00000165209.14	ENSG00000165417.7
	ENSG00000165527.5	ENSG00000166091.15	ENSG00000166340.10	ENSG00000166342.14
	ENSG00000166557.8	ENSG00000166704.7	ENSG00000166823.5	ENSG00000166831.4
	ENSG00000166856.1	ENSG00000166900.10	ENSG00000167112.7	ENSG00000167281.14
	ENSG00000167380.12	ENSG00000167550.6	ENSG00000167757.9	ENSG00000168350.6
	ENSG00000168389.13	ENSG00000168658.14	ENSG00000169129.10	ENSG00000169174.9
	ENSG00000169194.5	ENSG00000169403.7	ENSG00000169429.6	ENSG00000169550.8
	ENSG00000169908.6	ENSG00000169964.5	ENSG00000169992.5	ENSG00000170921.10
	ENSG00000171016.7	ENSG00000171219.8	ENSG00000171812.6	ENSG00000171819.4
	ENSG00000172005.6	ENSG00000172037.9	ENSG00000172243.13	ENSG00000172845.9
	ENSG00000173281.4	ENSG00000174576.4	ENSG00000174799.6	ENSG00000175029.12
	ENSG00000176095.7	ENSG00000176153.10	ENSG00000177494.5	ENSG00000178602.3

	ENSG00000178789.4	ENSG00000179869.10	ENSG00000180611.6	ENSG00000181982.13
	ENSG00000182077.6	ENSG00000182324.5	ENSG00000183055.5	ENSG00000183137.10
	ENSG00000183475.8	ENSG00000183576.8	ENSG00000184838.10	ENSG00000185567.6
	ENSG00000187566.3	ENSG00000187742.10	ENSG00000188242.4	ENSG00000189409.8
	ENSG00000196116.6	ENSG00000196639.6	ENSG00000197409.6	ENSG00000197763.8
	ENSG00000197838.4	ENSG00000197880.4	ENSG00000197930.8	ENSG00000198454.2
	ENSG00000198517.5	ENSG00000198668.6	ENSG00000204572.5	ENSG00000204963.4
	ENSG00000213197.3	ENSG00000213225.6	ENSG00000213551.4	ENSG00000214013.5
	ENSG00000214146.2	ENSG00000214362.2	ENSG00000214561.3	ENSG00000215187.5
	ENSG00000215481.4	ENSG00000218175.2	ENSG00000221869.4	ENSG00000223601.2
	ENSG00000224186.4	ENSG00000224858.4	ENSG00000224956.5	ENSG00000225930.3
	ENSG00000227477.1	ENSG00000227920.2	ENSG00000228008.1	ENSG00000228058.1
	ENSG00000228223.1	ENSG00000228397.1	ENSG00000228522.2	ENSG00000229291.1
	ENSG00000230850.3	ENSG00000231322.1	ENSG00000231485.1	ENSG00000232833.4
	ENSG00000233122.1	ENSG00000233469.1	ENSG00000233895.1	ENSG00000234043.3
	ENSG00000234383.1	ENSG00000234536.1	ENSG00000234797.4	ENSG00000234862.1
	ENSG00000235373.1	ENSG00000236136.1	ENSG00000239486.1	ENSG00000239804.1
	ENSG00000240021.5	ENSG00000240132.1	ENSG00000240350.1	ENSG00000240747.3
	ENSG00000242375.1	ENSG00000243478.3	ENSG00000243672.1	ENSG00000244005.8
	ENSG00000245651.2	ENSG00000248916.1	ENSG00000249430.1	ENSG00000253304.1
	ENSG00000254332.1	ENSG00000254618.1	ENSG00000254786.1	ENSG00000255277.2
	ENSG00000255561.2	ENSG00000255595.1	ENSG00000255735.1	ENSG00000256167.1
	ENSG00000256361.1	ENSG00000256540.1	ENSG00000257061.1	ENSG00000257464.1
	ENSG00000258811.1	ENSG00000259158.1	ENSG00000259404.1	ENSG00000259751.1
	ENSG00000260464.1	ENSG00000261007.2	ENSG00000263105.1	ENSG00000264016.2
	ENSG00000266498.1	ENSG00000266651.1	ENSG00000266946.1	ENSG00000267416.1
	ENSG00000267654.1	ENSG00000268087.1	ENSG00000269271.1	ENSG00000269510.1
	ENSG00000269543.1	ENSG00000270557.1	ENSG00000270917.1	ENSG00000271734.1
	ENSG00000271856.1	ENSG00000272108.1	ENSG00000272235.1	ENSG00000272425.1
	ENSG00000272966.1			
**Whole Blood**	ENSG00000001629.5	ENSG00000006704.6	ENSG00000010282.10	ENSG00000010327.6
	ENSG00000047249.12	ENSG00000049246.10	ENSG00000057593.9	ENSG00000058668.10
	ENSG00000061794.8	ENSG00000068001.9	ENSG00000070495.10	ENSG00000072135.8
	ENSG00000072195.10	ENSG00000072201.9	ENSG00000073737.12	ENSG00000074696.8
	ENSG00000085741.8	ENSG00000087086.9	ENSG00000089159.11	ENSG00000091704.5
	ENSG00000092531.5	ENSG00000096696.9	ENSG00000099139.9	ENSG00000099246.12
	ENSG00000100142.10	ENSG00000100150.12	ENSG00000100351.12	ENSG00000100365.10
	ENSG00000100938.13	ENSG00000102524.7	ENSG00000103064.9	ENSG00000103160.7
	ENSG00000104131.8	ENSG00000104951.11	ENSG00000104976.7	ENSG00000105948.9
	ENSG00000106211.8	ENSG00000108773.6	ENSG00000109066.9	ENSG00000109452.8
	ENSG00000109819.4	ENSG00000111490.8	ENSG00000112195.8	ENSG00000112312.5
	ENSG00000112531.12	ENSG00000112769.14	ENSG00000113161.11	ENSG00000113758.9
	ENSG00000114019.10	ENSG00000114439.14	ENSG00000115274.10	ENSG00000115325.9
	ENSG00000116096.5	ENSG00000116171.12	ENSG00000116711.8	ENSG00000118518.11
	ENSG00000118520.9	ENSG00000118733.12	ENSG00000119685.15	ENSG00000120318.11
	ENSG00000120686.7	ENSG00000120913.19	ENSG00000121774.13	ENSG00000121848.9
	ENSG00000121933.13	ENSG00000123505.10	ENSG00000124102.4	ENSG00000125107.12
	ENSG00000125910.4	ENSG00000128918.10	ENSG00000129559.8	ENSG00000130487.4
	ENSG00000130584.6	ENSG00000131386.13	ENSG00000132405.14	ENSG00000132963.7

ENSG00000133114.13	ENSG00000134250.13	ENSG00000134571.6	ENSG00000135378.3
ENSG00000135387.15	ENSG00000135679.17	ENSG00000135899.12	ENSG00000135945.5
ENSG00000136634.5	ENSG00000136653.15	ENSG00000136943.6	ENSG00000138785.10
ENSG00000139218.13	ENSG00000139546.6	ENSG00000140280.9	ENSG00000140395.4
ENSG00000140675.8	ENSG00000141564.9	ENSG00000143127.8	ENSG00000143515.12
ENSG00000143740.10	ENSG00000144868.9	ENSG00000145088.4	ENSG00000145107.11
ENSG00000147689.12	ENSG00000148356.9	ENSG00000148481.9	ENSG00000148926.5
ENSG00000149591.12	ENSG00000149634.4	ENSG00000150625.12	ENSG00000151014.4
ENSG00000151500.10	ENSG00000152128.13	ENSG00000152380.5	ENSG00000154122.8
ENSG00000155666.7	ENSG00000155755.14	ENSG00000159189.7	ENSG00000159674.7
ENSG00000160602.9	ENSG00000160993.3	ENSG00000161911.7	ENSG00000162434.7
ENSG00000162777.12	ENSG00000163421.4	ENSG00000163486.8	ENSG00000163491.12
ENSG00000163563.7	ENSG00000163704.7	ENSG00000164620.4	ENSG00000164744.8
ENSG00000165417.7	ENSG00000165487.9	ENSG00000166147.9	ENSG00000166340.10
ENSG00000166704.7	ENSG00000167196.9	ENSG00000167210.12	ENSG00000167862.5
ENSG00000168374.6	ENSG00000168658.14	ENSG00000169174.9	ENSG00000169397.3
ENSG00000169964.5	ENSG00000170027.5	ENSG00000170185.5	ENSG00000170584.6
ENSG00000170855.3	ENSG00000170921.10	ENSG00000171033.8	ENSG00000171467.11
ENSG00000172830.8	ENSG00000173175.10	ENSG00000173281.4	ENSG00000174799.6
ENSG00000175029.12	ENSG00000175756.9	ENSG00000175857.4	ENSG00000175931.8
ENSG00000176261.11	ENSG00000176371.9	ENSG00000176956.8	ENSG00000177156.6
ENSG00000177954.7	ENSG00000178878.8	ENSG00000180178.6	ENSG00000181982.13
ENSG00000182831.7	ENSG00000183576.8	ENSG00000184261.4	ENSG00000184363.5
ENSG00000184451.5	ENSG00000185955.4	ENSG00000186010.14	ENSG00000186076.4
ENSG00000187037.4	ENSG00000187116.9	ENSG00000187498.10	ENSG00000188404.4
ENSG00000188523.4	ENSG00000189409.8	ENSG00000196116.6	ENSG00000196453.7
ENSG00000196754.6	ENSG00000197386.6	ENSG00000197409.6	ENSG00000197429.6
ENSG00000198728.6	ENSG00000203943.4	ENSG00000204261.4	ENSG00000204390.8
ENSG00000204963.4	ENSG00000213225.6	ENSG00000213355.3	ENSG00000213380.9
ENSG00000213934.5	ENSG00000214146.2	ENSG00000214413.3	ENSG00000215187.5
ENSG00000215481.4	ENSG00000217644.4	ENSG00000218016.2	ENSG00000225872.2
ENSG00000226567.1	ENSG00000228223.1	ENSG00000228397.1	ENSG00000229180.4
ENSG00000229291.1	ENSG00000229314.4	ENSG00000230185.4	ENSG00000230850.3
ENSG00000231645.2	ENSG00000231720.1	ENSG00000232815.1	ENSG00000232833.4
ENSG00000232837.1	ENSG00000233077.1	ENSG00000233111.1	ENSG00000233122.1
ENSG00000234284.2	ENSG00000236136.1	ENSG00000236155.2	ENSG00000236675.1
ENSG00000240350.1	ENSG00000240747.3	ENSG00000241954.1	ENSG00000243627.4
ENSG00000247134.2	ENSG00000248698.1	ENSG00000249709.3	ENSG00000249715.5
ENSG00000250635.1	ENSG00000253641.1	ENSG00000254786.1	ENSG00000255561.2
ENSG00000255595.1	ENSG00000255624.1	ENSG00000256540.1	ENSG00000256582.1
ENSG00000256720.1	ENSG00000257464.1	ENSG00000258418.1	ENSG00000258512.1
ENSG00000260971.3	ENSG00000261787.1	ENSG00000262951.1	ENSG00000266498.1
ENSG00000266853.1	ENSG00000267416.1	ENSG00000268865.1	ENSG00000269165.1
ENSG00000269907.1	ENSG00000270966.1	ENSG00000271511.1	ENSG00000271927.1
ENSG00000272942.1			

Table S6: List-PrediXcan

**Adipose Subcutaneous**	ENSG00000002330.9	ENSG00000002822.11	ENSG00000005238.15	ENSG00000005955.8
	ENSG00000005981.8	ENSG00000006432.11	ENSG00000006453.9	ENSG00000008226.15
	ENSG00000008324.6	ENSG00000010072.11	ENSG00000011638.6	ENSG00000013288.4
	ENSG00000014641.13	ENSG00000029725.12	ENSG00000035720.3	ENSG00000043514.11
	ENSG00000047315.10	ENSG00000047579.15	ENSG00000050327.10	ENSG00000051596.5
	ENSG00000054219.9	ENSG00000058091.12	ENSG00000066855.11	ENSG00000068781.16
	ENSG00000070759.12	ENSG00000070831.11	ENSG00000072849.6	ENSG00000073008.10
	ENSG00000074047.16	ENSG00000076242.10	ENSG00000076826.5	ENSG00000077063.6
	ENSG00000079841.14	ENSG00000080815.14	ENSG00000080823.17	ENSG00000083099.6
	ENSG00000086015.16	ENSG00000086065.9	ENSG00000091181.15	ENSG00000092036.12
	ENSG00000096996.11	ENSG00000099338.18	ENSG00000099954.14	ENSG00000099956.13
	ENSG00000099957.12	ENSG00000100027.10	ENSG00000100079.5	ENSG00000100280.12
	ENSG00000100330.11	ENSG00000100360.10	ENSG00000100767.11	ENSG00000101443.13
	ENSG00000101639.14	ENSG00000103257.4	ENSG00000103710.6	ENSG00000103942.8
	ENSG00000104728.11	ENSG00000104852.10	ENSG00000104980.3	ENSG00000105088.4
	ENSG00000105426.10	ENSG00000105707.9	ENSG00000105852.6	ENSG00000106397.7
	ENSG00000106638.11	ENSG00000107438.4	ENSG00000107854.5	ENSG00000108264.12
	ENSG00000108381.6	ENSG00000108439.5	ENSG00000108559.7	ENSG00000108786.6
	ENSG00000109576.9	ENSG00000110660.10	ENSG00000111237.14	ENSG00000111361.8
	ENSG00000111596.7	ENSG00000111652.5	ENSG00000111664.6	ENSG00000111665.7
	ENSG00000112294.8	ENSG00000112419.10	ENSG00000112667.8	ENSG00000112763.11
	ENSG00000112941.8	ENSG00000113318.9	ENSG00000114200.5	ENSG00000115041.8
	ENSG00000115350.7	ENSG00000115808.7	ENSG00000115947.9	ENSG00000116260.12
	ENSG00000116641.11	ENSG00000116785.9	ENSG00000116957.8	ENSG00000118363.7
	ENSG00000118777.6	ENSG00000119321.4	ENSG00000119537.11	ENSG00000119986.6
	ENSG00000120451.6	ENSG00000121236.15	ENSG00000122025.10	ENSG00000122952.12
	ENSG00000123643.8	ENSG00000123870.9	ENSG00000124215.12	ENSG00000124356.11
	ENSG00000124570.13	ENSG00000125743.6	ENSG00000126226.17	ENSG00000126602.6
	ENSG00000126749.10	ENSG00000127249.10	ENSG00000129197.10	ENSG00000129538.9
	ENSG00000129596.4	ENSG00000130684.9	ENSG00000130762.10	ENSG00000131697.13
	ENSG00000132376.15	ENSG00000132854.14	ENSG00000133731.5	ENSG00000133812.10
	ENSG00000133983.10	ENSG00000135018.9	ENSG00000135040.11	ENSG00000135164.14
	ENSG00000135931.13	ENSG00000136371.5	ENSG00000136715.13	ENSG00000136810.8
	ENSG00000136827.11	ENSG00000136877.10	ENSG00000137509.6	ENSG00000137558.3
	ENSG00000137996.8	ENSG00000138172.6	ENSG00000138400.8	ENSG00000138448.7
	ENSG00000138801.4	ENSG00000139508.10	ENSG00000139572.3	ENSG00000139780.7
	ENSG00000141140.12	ENSG00000141150.3	ENSG00000141404.11	ENSG00000142599.13
	ENSG00000142856.12	ENSG00000142973.8	ENSG00000143067.4	ENSG00000143303.7
	ENSG00000143575.10	ENSG00000143740.10	ENSG00000143776.14	ENSG00000143924.14
	ENSG00000143951.11	ENSG00000144021.2	ENSG00000145390.7	ENSG00000145506.9
	ENSG00000147679.7	ENSG00000148672.7	ENSG00000148843.9	ENSG00000149179.9
	ENSG00000149308.12	ENSG00000150459.8	ENSG00000150753.7	ENSG00000151348.9
	ENSG00000151458.7	ENSG00000151553.10	ENSG00000151692.10	ENSG00000152253.4
	ENSG00000153048.6	ENSG00000153246.7	ENSG00000153291.11	ENSG00000153395.5
	ENSG00000153786.8	ENSG00000154359.8	ENSG00000154760.9	ENSG00000155324.5
	ENSG00000155754.10	ENSG00000155761.9	ENSG00000157184.5	ENSG00000157193.10
	ENSG00000157426.9	ENSG00000157578.9	ENSG00000157911.5	ENSG00000158106.8

ENSG00000158552.8	ENSG00000158669.7	ENSG00000160172.6	ENSG00000161326.8
ENSG00000163959.5	ENSG00000164038.10	ENSG00000164096.10	ENSG00000164338.5
ENSG00000164603.7	ENSG00000164626.8	ENSG00000164880.11	ENSG00000165124.13
ENSG00000165406.11	ENSG00000165650.7	ENSG00000165695.5	ENSG00000165912.11
ENSG00000166220.8	ENSG00000166333.9	ENSG00000166337.5	ENSG00000166402.4
ENSG00000166938.8	ENSG00000167191.7	ENSG00000167377.13	ENSG00000167642.8
ENSG00000167653.4	ENSG00000167670.11	ENSG00000167842.11	ENSG00000168329.9
ENSG00000168356.7	ENSG00000168394.9	ENSG00000168488.14	ENSG00000168634.4
ENSG00000168924.10	ENSG00000169609.9	ENSG00000169764.10	ENSG00000170275.10
ENSG00000170448.7	ENSG00000171943.7	ENSG00000171954.8	ENSG00000172175.8
ENSG00000173200.8	ENSG00000173915.8	ENSG00000173992.4	ENSG00000174194.11
ENSG00000174226.4	ENSG00000174652.13	ENSG00000174950.6	ENSG00000175899.10
ENSG00000176155.14	ENSG00000177302.10	ENSG00000177370.4	ENSG00000177628.11
ENSG00000178055.8	ENSG00000178177.10	ENSG00000178217.9	ENSG00000178381.7
ENSG00000178386.8	ENSG00000178397.8	ENSG00000179242.11	ENSG00000179296.9
ENSG00000179580.5	ENSG00000179774.7	ENSG00000179862.5	ENSG00000180185.7
ENSG00000180481.6	ENSG00000180537.8	ENSG00000180881.15	ENSG00000181007.7
ENSG00000181061.9	ENSG00000181315.6	ENSG00000181744.4	ENSG00000182057.4
ENSG00000182704.6	ENSG00000182771.13	ENSG00000183087.10	ENSG00000183662.6
ENSG00000183748.4	ENSG00000183785.10	ENSG00000184014.3	ENSG00000184344.3
ENSG00000184389.8	ENSG00000184517.7	ENSG00000184574.5	ENSG00000185298.8
ENSG00000185340.11	ENSG00000185437.9	ENSG00000186026.6	ENSG00000186153.12
ENSG00000186160.4	ENSG00000186377.6	ENSG00000186448.10	ENSG00000186470.9
ENSG00000186472.15	ENSG00000187189.9	ENSG00000187193.8	ENSG00000188610.8
ENSG00000188659.5	ENSG00000189196.4	ENSG00000189280.3	ENSG00000196345.8
ENSG00000196403.4	ENSG00000196458.6	ENSG00000196743.4	ENSG00000197081.8
ENSG00000197093.6	ENSG00000197146.2	ENSG00000197165.6	ENSG00000197291.4
ENSG00000197444.5	ENSG00000197498.8	ENSG00000197566.5	ENSG00000197646.6
ENSG00000197747.4	ENSG00000198453.8	ENSG00000198870.6	ENSG00000198885.5
ENSG00000198931.6	ENSG00000198945.3	ENSG00000203843.3	ENSG00000204228.3
ENSG00000204237.4	ENSG00000204287.9	ENSG00000204301.5	ENSG00000204498.6
ENSG00000204520.8	ENSG00000204529.3	ENSG00000204574.8	ENSG00000204959.3
ENSG00000204977.5	ENSG00000204978.2	ENSG00000205045.4	ENSG00000205822.6
ENSG00000206028.1	ENSG00000206129.3	ENSG00000212864.2	ENSG00000213244.3
ENSG00000213492.2	ENSG00000213626.7	ENSG00000213753.6	ENSG00000214754.3
ENSG00000215241.3	ENSG00000215440.7	ENSG00000215861.4	ENSG00000217783.2
ENSG00000220563.1	ENSG00000223496.1	ENSG00000223561.2	ENSG00000223956.1
ENSG00000224577.1	ENSG00000224961.1	ENSG00000225241.3	ENSG00000225684.3
ENSG00000225968.4	ENSG00000226318.1	ENSG00000226816.2	ENSG00000227359.1
ENSG00000227676.2	ENSG00000228307.1	ENSG00000228600.1	ENSG00000229474.2
ENSG00000232202.1	ENSG00000232224.1	ENSG00000232372.1	ENSG00000232677.2
ENSG00000232810.3	ENSG00000233232.2	ENSG00000233754.1	ENSG00000233961.1
ENSG00000233967.2	ENSG00000234112.2	ENSG00000235098.4	ENSG00000235117.2
ENSG00000235286.1	ENSG00000236474.1	ENSG00000236624.4	ENSG00000237510.3
ENSG00000240163.1	ENSG00000240563.1	ENSG00000241043.1	ENSG00000241484.5
ENSG00000242247.6	ENSG00000242441.3	ENSG00000242689.1	ENSG00000245937.3
ENSG00000247134.2	ENSG00000248610.1	ENSG00000249646.2	ENSG00000249915.3
ENSG00000250334.1	ENSG00000250337.1	ENSG00000250411.1	ENSG00000250571.2
ENSG00000250786.1	ENSG00000251521.2	ENSG00000251593.1	ENSG00000254272.1

	ENSG00000254285.2	ENSG00000254681.2	ENSG00000254761.1	ENSG00000254943.1
	ENSG00000255121.2	ENSG00000256223.1	ENSG00000258713.2	ENSG00000259051.1
	ENSG00000259539.1	ENSG00000261455.1	ENSG00000261556.4	ENSG00000263142.1
	ENSG00000263508.1	ENSG00000266554.1	ENSG00000266930.2	ENSG00000267056.2
	ENSG00000267325.1	ENSG00000267369.1	ENSG00000267405.1	ENSG00000267575.2
	ENSG00000267623.2	ENSG00000267651.1	ENSG00000267886.1	ENSG00000267939.1
	ENSG00000268172.1	ENSG00000268707.1	ENSG00000269086.2	ENSG00000269430.1
	ENSG00000269439.1	ENSG00000269746.1	ENSG00000271095.1	ENSG00000271109.1
	ENSG00000271550.1	ENSG00000271623.1	ENSG00000271889.1	ENSG00000271897.1
	ENSG00000272129.1	ENSG00000272462.2	ENSG00000272537.1	ENSG00000272542.1
	ENSG00000272892.1	ENSG00000273139.1	ENSG00000273218.1	
**Adipose Visceral Omentum**	ENSG00000004777.14	ENSG00000005955.8	ENSG00000005981.8	ENSG00000006114.11
	ENSG00000014641.13	ENSG00000018510.8	ENSG00000019991.11	ENSG00000035687.9
	ENSG00000050327.10	ENSG00000060709.9	ENSG00000066855.11	ENSG00000067836.8
	ENSG00000069966.14	ENSG00000074211.9	ENSG00000075073.10	ENSG00000075461.5
	ENSG00000082258.8	ENSG00000083099.6	ENSG00000086232.8	ENSG00000086288.7
	ENSG00000088538.12	ENSG00000089693.6	ENSG00000090372.10	ENSG00000090565.11
	ENSG00000091262.10	ENSG00000092036.12	ENSG00000092929.7	ENSG00000099246.12
	ENSG00000099956.13	ENSG00000100461.13	ENSG00000100478.10	ENSG00000101236.12
	ENSG00000101265.11	ENSG00000101639.14	ENSG00000102781.9	ENSG00000103599.15
	ENSG00000104852.10	ENSG00000104980.3	ENSG00000106125.14	ENSG00000106526.6
	ENSG00000107854.5	ENSG00000108176.10	ENSG00000108439.5	ENSG00000108733.5
	ENSG00000109390.7	ENSG00000109576.9	ENSG00000109991.4	ENSG00000111237.14
	ENSG00000111664.6	ENSG00000111665.7	ENSG00000111816.6	ENSG00000113318.9
	ENSG00000114735.5	ENSG00000115350.7	ENSG00000116690.7	ENSG00000116785.9
	ENSG00000116957.8	ENSG00000117395.6	ENSG00000117419.10	ENSG00000119147.5
	ENSG00000119321.4	ENSG00000119537.11	ENSG00000120008.11	ENSG00000121440.10
	ENSG00000121542.7	ENSG00000121864.5	ENSG00000122025.10	ENSG00000122376.7
	ENSG00000122547.6	ENSG00000122692.7	ENSG00000123191.9	ENSG00000124006.10
	ENSG00000124212.5	ENSG00000125743.6	ENSG00000126267.4	ENSG00000126749.10
	ENSG00000127870.12	ENSG00000128052.8	ENSG00000128710.5	ENSG00000129197.10
	ENSG00000129467.9	ENSG00000131389.12	ENSG00000131697.13	ENSG00000132842.9
	ENSG00000133731.5	ENSG00000133983.10	ENSG00000134905.12	ENSG00000135093.8
	ENSG00000135999.7	ENSG00000137070.13	ENSG00000137996.8	ENSG00000138101.14
	ENSG00000138777.15	ENSG00000138829.6	ENSG00000139160.9	ENSG00000139178.6
	ENSG00000139445.13	ENSG00000140451.8	ENSG00000141127.10	ENSG00000141140.12
	ENSG00000142319.14	ENSG00000142599.13	ENSG00000142973.8	ENSG00000143036.12
	ENSG00000143951.11	ENSG00000144021.2	ENSG00000144026.7	ENSG00000144362.7
	ENSG00000144589.16	ENSG00000144837.4	ENSG00000145388.10	ENSG00000145390.7
	ENSG00000145555.10	ENSG00000145569.5	ENSG00000146066.2	ENSG00000146083.7
	ENSG00000146094.9	ENSG00000146904.4	ENSG00000147647.8	ENSG00000148908.10
	ENSG00000149311.13	ENSG00000151348.9	ENSG00000151553.10	ENSG00000152253.4
	ENSG00000152315.4	ENSG00000152359.10	ENSG00000152433.10	ENSG00000152683.10
	ENSG00000153395.5	ENSG00000153993.9	ENSG00000154065.12	ENSG00000154767.10
	ENSG00000155066.11	ENSG00000155254.8	ENSG00000156011.12	ENSG00000156510.11
	ENSG00000157379.9	ENSG00000157837.11	ENSG00000159387.7	ENSG00000160172.6
	ENSG00000160207.4	ENSG00000162384.9	ENSG00000162643.8	ENSG00000162997.11
	ENSG00000163364.5	ENSG00000164089.4	ENSG00000164338.5	ENSG00000164880.11
	ENSG00000165406.11	ENSG00000165487.9	ENSG00000165650.7	ENSG00000166471.6

ENSG00000166938.8	ENSG00000166947.7	ENSG00000167236.2	ENSG00000167565.8
ENSG00000167785.4	ENSG00000167842.11	ENSG00000168079.12	ENSG00000168411.9
ENSG00000168614.13	ENSG00000169629.7	ENSG00000169764.10	ENSG00000169894.13
ENSG00000169914.5	ENSG00000169994.14	ENSG00000170903.6	ENSG00000171722.7
ENSG00000171943.7	ENSG00000172901.15	ENSG00000173226.12	ENSG00000174226.4
ENSG00000174652.13	ENSG00000175764.10	ENSG00000175879.7	ENSG00000175899.10
ENSG00000176155.14	ENSG00000176358.11	ENSG00000176956.8	ENSG00000177590.6
ENSG00000178188.10	ENSG00000178201.3	ENSG00000178386.8	ENSG00000178425.9
ENSG00000179364.9	ENSG00000179611.2	ENSG00000179673.3	ENSG00000179841.8
ENSG00000179978.10	ENSG00000180185.7	ENSG00000180353.6	ENSG00000180481.6
ENSG00000180773.10	ENSG00000180881.15	ENSG00000180953.7	ENSG00000182196.9
ENSG00000182218.5	ENSG00000182771.13	ENSG00000184014.3	ENSG00000184163.3
ENSG00000184389.8	ENSG00000184517.7	ENSG00000184602.5	ENSG00000184992.10
ENSG00000185347.13	ENSG00000186275.7	ENSG00000186470.9	ENSG00000186766.7
ENSG00000188610.8	ENSG00000188659.5	ENSG00000188818.8	ENSG00000188846.9
ENSG00000189014.6	ENSG00000189280.3	ENSG00000196189.8	ENSG00000196611.4
ENSG00000196743.4	ENSG00000197165.6	ENSG00000197261.7	ENSG00000197291.4
ENSG00000197496.4	ENSG00000197646.6	ENSG00000197712.7	ENSG00000198028.3
ENSG00000198171.8	ENSG00000198722.8	ENSG00000198885.5	ENSG00000198945.3
ENSG00000203843.3	ENSG00000204020.5	ENSG00000204301.5	ENSG00000204305.9
ENSG00000204520.8	ENSG00000204529.3	ENSG00000204709.4	ENSG00000204856.7
ENSG00000204929.7	ENSG00000204959.3	ENSG00000204977.5	ENSG00000205045.4
ENSG00000205771.2	ENSG00000205822.6	ENSG00000206503.7	ENSG00000212126.3
ENSG00000213626.7	ENSG00000213753.6	ENSG00000213839.4	ENSG00000214194.4
ENSG00000214946.9	ENSG00000215712.6	ENSG00000215908.5	ENSG00000216895.4
ENSG00000219200.6	ENSG00000223345.3	ENSG00000223496.1	ENSG00000224577.1
ENSG00000225241.3	ENSG00000225784.5	ENSG00000225851.1	ENSG00000226542.1
ENSG00000226598.1	ENSG00000227676.2	ENSG00000228307.1	ENSG00000228998.3
ENSG00000229268.1	ENSG00000229657.2	ENSG00000229827.1	ENSG00000231793.4
ENSG00000231816.1	ENSG00000231925.7	ENSG00000232063.1	ENSG00000232872.2
ENSG00000233232.2	ENSG00000233295.3	ENSG00000235098.4	ENSG00000235117.2
ENSG00000235472.1	ENSG00000236094.1	ENSG00000236624.4	ENSG00000237510.3
ENSG00000237560.1	ENSG00000237672.1	ENSG00000240356.2	ENSG00000240970.1
ENSG00000241015.2	ENSG00000242247.6	ENSG00000242441.3	ENSG00000242611.1
ENSG00000242950.2	ENSG00000244045.6	ENSG00000244414.2	ENSG00000244753.2
ENSG00000246922.4	ENSG00000248408.1	ENSG00000249646.2	ENSG00000249908.1
ENSG00000249915.3	ENSG00000250334.1	ENSG00000250786.1	ENSG00000251580.1
ENSG00000253558.1	ENSG00000253598.1	ENSG00000253853.1	ENSG00000254319.1
ENSG00000254595.1	ENSG00000254761.1	ENSG00000255374.1	ENSG00000257114.1
ENSG00000257877.1	ENSG00000258331.1	ENSG00000259494.1	ENSG00000261229.1
ENSG00000261455.1	ENSG00000261556.4	ENSG00000261575.2	ENSG00000261770.1
ENSG00000262370.1	ENSG00000263142.1	ENSG00000263164.1	ENSG00000263603.1
ENSG00000267575.2	ENSG00000267623.2	ENSG00000267939.1	ENSG00000269086.2
ENSG00000269190.1	ENSG00000270469.1	ENSG00000271109.1	ENSG00000271889.1
ENSG00000272024.1	ENSG00000272129.1	ENSG00000272236.1	ENSG00000272455.1
ENSG00000272462.2	ENSG00000272537.1	ENSG00000272908.1	

ENSG00000003249.9	ENSG00000005955.8	ENSG00000013288.4	ENSG00000014641.13
ENSG00000030110.8	ENSG00000043514.11	ENSG00000047188.11	ENSG00000049449.4
ENSG00000057252.8	ENSG00000065150.14	ENSG00000071794.11	ENSG00000075239.9

Adrenal

ENSG00000086189.5	ENSG00000086232.8	ENSG00000088038.13	ENSG00000089169.10
ENSG00000091262.10	ENSG00000092036.12	ENSG00000101166.11	ENSG00000101558.9
ENSG00000101639.14	ENSG00000103226.13	ENSG00000103351.8	ENSG00000104888.5
ENSG00000105088.4	ENSG00000105698.11	ENSG00000105738.6	ENSG00000106628.6
ENSG00000108064.6	ENSG00000108264.12	ENSG00000108433.11	ENSG00000108963.13
ENSG00000109466.9	ENSG00000109991.4	ENSG00000110455.9	ENSG00000111196.5
ENSG00000111215.7	ENSG00000112667.8	ENSG00000112977.11	ENSG00000113318.9
ENSG00000116688.12	ENSG00000116957.8	ENSG00000117226.7	ENSG00000120451.6
ENSG00000121904.13	ENSG00000122376.7	ENSG00000123159.11	ENSG00000123191.9
ENSG00000123643.8	ENSG00000124613.4	ENSG00000126218.7	ENSG00000126602.6
ENSG00000130957.4	ENSG00000131188.7	ENSG00000131730.11	ENSG00000133731.5
ENSG00000134755.10	ENSG00000136379.7	ENSG00000136682.10	ENSG00000137817.12
ENSG00000138376.6	ENSG00000138400.8	ENSG00000138801.4	ENSG00000139405.11
ENSG00000140009.14	ENSG00000140511.7	ENSG00000140990.10	ENSG00000141127.10
ENSG00000141150.3	ENSG00000141562.13	ENSG00000141580.11	ENSG00000142856.12
ENSG00000144021.2	ENSG00000144026.7	ENSG00000144724.14	ENSG00000145147.15
ENSG00000145348.12	ENSG00000148814.13	ENSG00000149115.9	ENSG00000149260.10
ENSG00000149311.13	ENSG00000152894.10	ENSG00000153246.7	ENSG00000153291.11
ENSG00000154864.7	ENSG00000155254.8	ENSG00000157259.6	ENSG00000157335.15
ENSG00000157578.9	ENSG00000157837.11	ENSG00000158714.6	ENSG00000161091.8
ENSG00000161896.6	ENSG00000162779.16	ENSG00000162909.13	ENSG00000162946.16
ENSG00000162994.11	ENSG00000164828.13	ENSG00000164855.11	ENSG00000164880.11
ENSG00000164904.11	ENSG00000165055.11	ENSG00000165392.5	ENSG00000165650.7
ENSG00000166938.8	ENSG00000167080.4	ENSG00000167207.7	ENSG00000167208.10
ENSG00000168116.9	ENSG00000168411.9	ENSG00000169964.5	ENSG00000170175.6
ENSG00000170545.12	ENSG00000171943.7	ENSG00000172803.13	ENSG00000173218.10
ENSG00000173226.12	ENSG00000173915.8	ENSG00000174652.13	ENSG00000175764.10
ENSG00000176155.14	ENSG00000176222.7	ENSG00000176654.8	ENSG00000176998.3
ENSG00000177106.10	ENSG00000177427.8	ENSG00000177548.8	ENSG00000178301.3
ENSG00000180185.7	ENSG00000180481.6	ENSG00000180891.8	ENSG00000182397.10
ENSG00000182853.7	ENSG00000183617.4	ENSG00000185261.9	ENSG00000185689.11
ENSG00000186448.10	ENSG00000186470.9	ENSG00000186952.10	ENSG00000187522.9
ENSG00000187741.10	ENSG00000188171.10	ENSG00000188388.9	ENSG00000188629.7
ENSG00000188659.5	ENSG00000188827.6	ENSG00000188897.4	ENSG00000188958.5
ENSG00000189050.10	ENSG00000196247.7	ENSG00000196313.7	ENSG00000196458.6
ENSG00000196743.4	ENSG00000196814.10	ENSG00000196843.11	ENSG00000197165.6
ENSG00000197321.10	ENSG00000198019.8	ENSG00000198774.3	ENSG00000198836.4
ENSG00000198959.7	ENSG00000203907.5	ENSG00000204092.2	ENSG00000204520.8
ENSG00000204574.8	ENSG00000204977.5	ENSG00000205746.5	ENSG00000213872.3
ENSG00000214456.4	ENSG00000215908.5	ENSG00000220161.4	ENSG00000223345.3
ENSG00000223496.1	ENSG00000223922.1	ENSG00000224389.4	ENSG00000225496.1
ENSG00000225784.5	ENSG00000227141.2	ENSG00000227676.2	ENSG00000228376.3
ENSG00000230175.1	ENSG00000232063.1	ENSG00000232224.1	ENSG00000232411.1
ENSG00000234350.1	ENSG00000234420.3	ENSG00000234965.1	ENSG00000235098.4
ENSG00000236624.4	ENSG00000237232.3	ENSG00000237248.3	ENSG00000241043.1
ENSG00000241288.3	ENSG00000242247.6	ENSG00000242265.1	ENSG00000243478.3
ENSG00000244414.2	ENSG00000247373.2	ENSG00000248408.1	ENSG00000250334.1
ENSG00000254416.1	ENSG00000254481.1	ENSG00000254858.5	ENSG00000258408.1
ENSG00000259344.1	ENSG00000260077.1	ENSG00000260645.1	ENSG00000260807.2

	ENSG00000260951.1	ENSG00000262165.1	ENSG00000269343.2	ENSG00000269746.1
	ENSG00000270614.1	ENSG00000271550.1	ENSG00000272129.1	ENSG00000272221.1
	ENSG00000272462.2	ENSG00000272537.1	ENSG00000272563.1	ENSG00000272711.1
	ENSG00000273340.1			
**Artery Aorta**	ENSG00000003056.3	ENSG00000005238.15	ENSG00000005955.8	ENSG00000006432.11
	ENSG00000006715.11	ENSG00000006747.10	ENSG00000010244.12	ENSG00000051341.9
	ENSG00000051620.6	ENSG00000053372.4	ENSG00000056487.11	ENSG00000064218.4
	ENSG00000072182.8	ENSG00000075131.5	ENSG00000077984.4	ENSG00000079785.10
	ENSG00000081913.9	ENSG00000083123.10	ENSG00000086506.2	ENSG00000086598.6
	ENSG00000088538.12	ENSG00000089220.4	ENSG00000089327.10	ENSG00000092036.12
	ENSG00000096717.7	ENSG00000097033.10	ENSG00000099624.3	ENSG00000099956.13
	ENSG00000100124.8	ENSG00000100266.13	ENSG00000101460.8	ENSG00000101608.8
	ENSG00000101639.14	ENSG00000103966.5	ENSG00000104131.8	ENSG00000105290.7
	ENSG00000105854.8	ENSG00000106511.5	ENSG00000106638.11	ENSG00000106688.7
	ENSG00000107854.5	ENSG00000108264.12	ENSG00000108272.9	ENSG00000108381.6
	ENSG00000108439.5	ENSG00000108576.5	ENSG00000108786.6	ENSG00000109576.9
	ENSG00000111199.6	ENSG00000111664.6	ENSG00000112378.11	ENSG00000112619.6
	ENSG00000112667.8	ENSG00000112761.14	ENSG00000112902.7	ENSG00000113318.9
	ENSG00000113569.11	ENSG00000114200.5	ENSG00000114279.9	ENSG00000115641.14
	ENSG00000115828.11	ENSG00000116785.9	ENSG00000117226.7	ENSG00000117477.8
	ENSG00000117834.8	ENSG00000118777.6	ENSG00000119537.11	ENSG00000119661.10
	ENSG00000119777.14	ENSG00000120451.6	ENSG00000120519.10	ENSG00000120907.13
	ENSG00000121067.13	ENSG00000121542.7	ENSG00000121577.9	ENSG00000122376.7
	ENSG00000122778.5	ENSG00000122870.7	ENSG00000123191.9	ENSG00000124614.9
	ENSG00000124839.8	ENSG00000125633.6	ENSG00000126246.5	ENSG00000131398.9
	ENSG00000131788.11	ENSG00000132613.10	ENSG00000132849.14	ENSG00000133740.6
	ENSG00000133983.10	ENSG00000134905.12	ENSG00000135164.14	ENSG00000136014.7
	ENSG00000136237.14	ENSG00000136827.11	ENSG00000136877.10	ENSG00000137075.13
	ENSG00000137310.7	ENSG00000137992.10	ENSG00000137996.8	ENSG00000138039.10
	ENSG00000138152.7	ENSG00000138386.12	ENSG00000138400.8	ENSG00000138777.15
	ENSG00000139168.3	ENSG00000139211.5	ENSG00000139343.6	ENSG00000140299.7
	ENSG00000140326.8	ENSG00000140905.5	ENSG00000141012.8	ENSG00000141127.10
	ENSG00000141140.12	ENSG00000141384.7	ENSG00000142065.9	ENSG00000142207.5
	ENSG00000142235.4	ENSG00000142279.8	ENSG00000142973.8	ENSG00000143314.8
	ENSG00000144021.2	ENSG00000144026.7	ENSG00000144134.14	ENSG00000144161.8
	ENSG00000144362.7	ENSG00000145390.7	ENSG00000145780.6	ENSG00000148331.7
	ENSG00000148843.9	ENSG00000149311.13	ENSG00000150527.12	ENSG00000150967.13
	ENSG00000151176.3	ENSG00000151348.9	ENSG00000152465.13	ENSG00000153446.11
	ENSG00000153786.8	ENSG00000154645.9	ENSG00000155254.8	ENSG00000157335.15
	ENSG00000160200.13	ENSG00000160593.13	ENSG00000161326.8	ENSG00000163257.6
	ENSG00000163827.8	ENSG00000164556.7	ENSG00000164941.9	ENSG00000165406.11
	ENSG00000165646.7	ENSG00000165650.7	ENSG00000165661.11	ENSG00000165757.8
	ENSG00000166261.6	ENSG00000166333.9	ENSG00000166398.8	ENSG00000166923.6
	ENSG00000167333.8	ENSG00000167383.4	ENSG00000167785.4	ENSG00000167842.11
	ENSG00000167969.8	ENSG00000168291.8	ENSG00000168394.9	ENSG00000168542.8
	ENSG00000168778.7	ENSG00000168899.4	ENSG00000169019.9	ENSG00000169509.5
	ENSG00000169609.9	ENSG00000169612.3	ENSG00000169660.11	ENSG00000170145.4
	ENSG00000170190.11	ENSG00000170379.15	ENSG00000170837.2	ENSG00000171105.9
	ENSG00000171865.5	ENSG00000171943.7	ENSG00000172340.10	ENSG00000173175.10

	ENSG00000174194.11	ENSG00000174514.8	ENSG00000174607.6	ENSG00000174652.13
	ENSG00000175764.10	ENSG00000176046.7	ENSG00000176155.14	ENSG00000176597.7
	ENSG00000176933.4	ENSG00000176998.3	ENSG00000177879.10	ENSG00000178297.8
	ENSG00000178386.8	ENSG00000179715.8	ENSG00000180185.7	ENSG00000180481.6
	ENSG00000180881.15	ENSG00000181104.6	ENSG00000182372.6	ENSG00000182575.7
	ENSG00000182704.6	ENSG00000182771.13	ENSG00000183748.4	ENSG00000184156.11
	ENSG00000184389.8	ENSG00000185344.9	ENSG00000186283.9	ENSG00000186470.9
	ENSG00000186652.5	ENSG00000187193.8	ENSG00000187792.3	ENSG00000188322.4
	ENSG00000188626.5	ENSG00000188659.5	ENSG00000188735.8	ENSG00000188886.3
	ENSG00000189362.7	ENSG00000196345.8	ENSG00000196843.11	ENSG00000197165.6
	ENSG00000197183.8	ENSG00000197465.9	ENSG00000197646.6	ENSG00000198768.6
	ENSG00000198885.5	ENSG00000198931.6	ENSG00000198945.3	ENSG00000203843.3
	ENSG00000204209.6	ENSG00000204237.4	ENSG00000204520.8	ENSG00000204655.7
	ENSG00000204713.6	ENSG00000204839.4	ENSG00000204899.5	ENSG00000204959.3
	ENSG00000213190.2	ENSG00000213626.7	ENSG00000213760.6	ENSG00000213780.6
	ENSG00000215861.4	ENSG00000223496.1	ENSG00000225241.3	ENSG00000225784.5
	ENSG00000225851.1	ENSG00000227725.2	ENSG00000228216.1	ENSG00000228789.2
	ENSG00000230615.2	ENSG00000230836.1	ENSG00000231360.2	ENSG00000231861.1
	ENSG00000231925.7	ENSG00000232040.2	ENSG00000232063.1	ENSG00000232677.2
	ENSG00000233232.2	ENSG00000233974.3	ENSG00000234949.2	ENSG00000235574.1
	ENSG00000235677.1	ENSG00000236624.4	ENSG00000237021.2	ENSG00000237176.3
	ENSG00000237510.3	ENSG00000240050.1	ENSG00000242441.3	ENSG00000243244.1
	ENSG00000243444.3	ENSG00000244731.3	ENSG00000245937.3	ENSG00000248098.6
	ENSG00000250334.1	ENSG00000251417.1	ENSG00000251580.1	ENSG00000253598.1
	ENSG00000254131.1	ENSG00000254319.1	ENSG00000255566.1	ENSG00000256223.1
	ENSG00000256232.1	ENSG00000258352.1	ENSG00000259083.1	ENSG00000259539.1
	ENSG00000259959.1	ENSG00000260105.2	ENSG00000261455.1	ENSG00000261556.4
	ENSG00000261770.1	ENSG00000262165.1	ENSG00000263603.1	ENSG00000267056.2
	ENSG00000267939.1	ENSG00000268912.1	ENSG00000269396.1	ENSG00000269489.1
	ENSG00000271824.1	ENSG00000272043.1	ENSG00000272144.1	ENSG00000272462.2
	ENSG00000272659.1	ENSG00000272810.1		
**Artery Coronary**	ENSG00000043514.11	ENSG00000049245.8	ENSG00000049246.10	ENSG00000065717.10
	ENSG00000075131.5	ENSG00000088538.12	ENSG00000089335.16	ENSG00000089818.12
	ENSG00000092036.12	ENSG00000093072.11	ENSG00000099956.13	ENSG00000100079.5
	ENSG00000100147.9	ENSG00000100314.3	ENSG00000100461.13	ENSG00000101255.6
	ENSG00000101365.16	ENSG00000102554.9	ENSG00000103351.8	ENSG00000104976.7
	ENSG00000105793.11	ENSG00000107854.5	ENSG00000108264.12	ENSG00000108352.7
	ENSG00000110455.9	ENSG00000110881.7	ENSG00000111271.10	ENSG00000111581.5
	ENSG00000112276.9	ENSG00000113318.9	ENSG00000116663.6	ENSG00000117226.7
	ENSG00000119227.3	ENSG00000119537.11	ENSG00000120451.6	ENSG00000120669.11
	ENSG00000120885.15	ENSG00000122376.7	ENSG00000124212.5	ENSG00000125885.9
	ENSG00000126858.12	ENSG00000130202.5	ENSG00000130299.12	ENSG00000131558.10
	ENSG00000133731.5	ENSG00000134030.9	ENSG00000136319.7	ENSG00000137996.8
	ENSG00000139547.6	ENSG00000141968.3	ENSG00000144026.7	ENSG00000145779.7
	ENSG00000146556.10	ENSG00000147852.11	ENSG00000148356.9	ENSG00000149054.10
	ENSG00000151553.10	ENSG00000151692.10	ENSG00000152253.4	ENSG00000153786.8
	ENSG00000154309.7	ENSG00000155363.14	ENSG00000156990.10	ENSG00000163682.11
	ENSG00000164334.11	ENSG00000164845.12	ENSG00000164880.11	ENSG00000165113.8
	ENSG00000165156.10	ENSG00000165646.7	ENSG00000165650.7	ENSG00000165983.10

	ENSG00000166664.9	ENSG00000166938.8	ENSG00000167377.13	ENSG00000167646.9
	ENSG00000167842.11	ENSG00000167920.4	ENSG00000168334.8	ENSG00000169660.11
	ENSG00000170558.4	ENSG00000171903.12	ENSG00000171928.9	ENSG00000171943.7
	ENSG00000172007.5	ENSG00000172379.14	ENSG00000173085.9	ENSG00000174194.11
	ENSG00000174652.13	ENSG00000175334.3	ENSG00000176155.14	ENSG00000176371.9
	ENSG00000176597.7	ENSG00000176998.3	ENSG00000178055.8	ENSG00000178386.8
	ENSG00000179796.7	ENSG00000180104.11	ENSG00000180185.7	ENSG00000180481.6
	ENSG00000180771.10	ENSG00000180881.15	ENSG00000180953.7	ENSG00000182771.13
	ENSG00000183605.12	ENSG00000183615.5	ENSG00000184517.7	ENSG00000187193.8
	ENSG00000188388.9	ENSG00000188659.5	ENSG00000188868.9	ENSG00000189164.10
	ENSG00000196993.4	ENSG00000197165.6	ENSG00000197646.6	ENSG00000198089.10
	ENSG00000203780.6	ENSG00000204463.8	ENSG00000204520.8	ENSG00000204954.5
	ENSG00000205822.6	ENSG00000213578.4	ENSG00000214113.6	ENSG00000214562.9
	ENSG00000214946.9	ENSG00000214982.6	ENSG00000215861.4	ENSG00000216901.1
	ENSG00000223345.3	ENSG00000223496.1	ENSG00000223804.1	ENSG00000224870.3
	ENSG00000225398.2	ENSG00000226360.4	ENSG00000228897.1	ENSG00000228998.3
	ENSG00000230911.1	ENSG00000231389.3	ENSG00000232063.1	ENSG00000232499.2
	ENSG00000233232.2	ENSG00000235098.4	ENSG00000236296.3	ENSG00000236297.1
	ENSG00000236624.4	ENSG00000237510.3	ENSG00000238132.2	ENSG00000240729.1
	ENSG00000241015.2	ENSG00000248712.3	ENSG00000249646.2	ENSG00000250334.1
	ENSG00000251580.1	ENSG00000253598.1	ENSG00000254761.1	ENSG00000254810.1
	ENSG00000255080.1	ENSG00000255362.1	ENSG00000259539.1	ENSG00000261556.4
	ENSG00000261589.1	ENSG00000261770.1	ENSG00000267534.1	ENSG00000267575.2
	ENSG00000267767.2	ENSG00000268442.1	ENSG00000269996.1	ENSG00000270173.1
	ENSG00000270775.1	ENSG00000271040.1	ENSG00000272462.2	ENSG00000273008.1
	ENSG00000005513.9	ENSG00000005955.8	ENSG00000006432.11	ENSG00000008128.18
	ENSG00000011083.4	ENSG00000014641.13	ENSG00000018510.8	ENSG00000027847.9
	ENSG00000032444.11	ENSG00000037042.8	ENSG00000043462.7	ENSG00000047579.15
	ENSG00000051620.6	ENSG00000061455.10	ENSG00000064763.6	ENSG00000065308.4
	ENSG00000070770.4	ENSG00000071242.7	ENSG00000074047.16	ENSG00000074657.9
	ENSG00000075131.5	ENSG00000078902.11	ENSG00000079785.10	ENSG00000081087.10
	ENSG00000083099.6	ENSG00000083123.10	ENSG00000084090.9	ENSG00000085719.7
	ENSG00000086289.7	ENSG00000087266.11	ENSG00000087448.5	ENSG00000088538.12
	ENSG00000089123.11	ENSG00000089220.4	ENSG00000089818.12	ENSG00000092036.12
	ENSG00000092871.12	ENSG00000096093.10	ENSG00000099956.13	ENSG00000099957.12
	ENSG00000100280.12	ENSG00000100612.9	ENSG00000101150.13	ENSG00000101193.6
	ENSG00000101255.6	ENSG00000101460.8	ENSG00000101608.8	ENSG00000101680.9
	ENSG00000101752.7	ENSG00000102743.10	ENSG00000103342.8	ENSG00000103496.10
	ENSG00000103591.8	ENSG00000103599.15	ENSG00000103710.6	ENSG00000104852.10
	ENSG00000105379.5	ENSG00000105383.10	ENSG00000106069.16	ENSG00000106638.11
	ENSG00000107185.8	ENSG00000107854.5	ENSG00000108264.12	ENSG00000108272.9
	ENSG00000108306.7	ENSG00000108384.10	ENSG00000108559.7	ENSG00000108786.6
	ENSG00000109084.9	ENSG00000109158.6	ENSG00000109576.9	ENSG00000110076.14
	ENSG00000110455.9	ENSG00000111144.5	ENSG00000111237.14	ENSG00000111530.8
**Artery Tibial**	ENSG00000111664.6	ENSG00000111665.7	ENSG00000111885.5	ENSG00000112164.5
	ENSG00000113296.10	ENSG00000113318.9	ENSG00000114790.8	ENSG00000114956.15
	ENSG00000115041.8	ENSG00000115350.7	ENSG00000115392.7	ENSG00000115446.7
	ENSG00000115539.9	ENSG00000115641.14	ENSG00000115649.11	ENSG00000117226.7
	ENSG00000117228.9	ENSG00000117682.12	ENSG00000117834.8	ENSG00000119537.11

ENSG00000120008.11	ENSG00000120451.6	ENSG00000121236.15	ENSG00000121542.7
ENSG00000122025.10	ENSG00000122729.14	ENSG00000122958.10	ENSG00000122970.11
ENSG00000123191.9	ENSG00000123836.10	ENSG00000124212.5	ENSG00000124614.9
ENSG00000124780.9	ENSG00000125633.6	ENSG00000126246.5	ENSG00000126804.9
ENSG00000129128.8	ENSG00000129197.10	ENSG00000129484.9	ENSG00000129596.4
ENSG00000130684.9	ENSG00000133055.4	ENSG00000133105.3	ENSG00000133731.5
ENSG00000133740.6	ENSG00000134013.11	ENSG00000134200.2	ENSG00000134905.12
ENSG00000136436.10	ENSG00000136717.10	ENSG00000136859.5	ENSG00000136875.8
ENSG00000137075.13	ENSG00000137411.12	ENSG00000137693.9	ENSG00000137992.10
ENSG00000137996.8	ENSG00000138036.14	ENSG00000138152.7	ENSG00000138231.8
ENSG00000138430.11	ENSG00000138463.8	ENSG00000138653.5	ENSG00000138801.4
ENSG00000139187.5	ENSG00000139211.5	ENSG00000139278.5	ENSG00000139343.6
ENSG00000139624.8	ENSG00000140326.8	ENSG00000140548.5	ENSG00000140632.12
ENSG00000140694.12	ENSG00000140750.12	ENSG00000140853.11	ENSG00000140948.7
ENSG00000140990.10	ENSG00000140995.12	ENSG00000141012.8	ENSG00000141140.12
ENSG00000141384.7	ENSG00000141404.11	ENSG00000141540.6	ENSG00000142973.8
ENSG00000143409.11	ENSG00000143450.10	ENSG00000143622.6	ENSG00000144021.2
ENSG00000144026.7	ENSG00000144283.17	ENSG00000145390.7	ENSG00000146477.4
ENSG00000147419.12	ENSG00000148843.9	ENSG00000149054.10	ENSG00000149308.12
ENSG00000149311.13	ENSG00000150753.7	ENSG00000150756.9	ENSG00000151176.3
ENSG00000151320.6	ENSG00000151348.9	ENSG00000151458.7	ENSG00000152253.4
ENSG00000152465.13	ENSG00000152778.7	ENSG00000153246.7	ENSG00000153786.8
ENSG00000153936.12	ENSG00000154645.9	ENSG00000155066.11	ENSG00000155254.8
ENSG00000155256.13	ENSG00000155324.5	ENSG00000156269.4	ENSG00000157087.12
ENSG00000157335.15	ENSG00000157570.7	ENSG00000157837.11	ENSG00000158714.6
ENSG00000159873.5	ENSG00000160233.6	ENSG00000161326.8	ENSG00000163288.9
ENSG00000163354.10	ENSG00000163359.11	ENSG00000163520.9	ENSG00000163904.8
ENSG00000164185.4	ENSG00000164338.5	ENSG00000164754.8	ENSG00000164855.11
ENSG00000164880.11	ENSG00000165006.9	ENSG00000165084.11	ENSG00000165646.7
ENSG00000165650.7	ENSG00000165757.8	ENSG00000166333.9	ENSG00000166913.8
ENSG00000167081.12	ENSG00000167383.4	ENSG00000167384.6	ENSG00000167535.3
ENSG00000167670.11	ENSG00000167754.8	ENSG00000167785.4	ENSG00000168394.9
ENSG00000168411.9	ENSG00000168569.7	ENSG00000168614.13	ENSG00000168743.8
ENSG00000168803.10	ENSG00000169087.6	ENSG00000169116.7	ENSG00000169710.6
ENSG00000169764.10	ENSG00000169860.4	ENSG00000169905.8	ENSG00000169994.14
ENSG00000170270.4	ENSG00000170899.6	ENSG00000170906.11	ENSG00000171649.7
ENSG00000172399.5	ENSG00000172493.16	ENSG00000172661.13	ENSG00000172938.3
ENSG00000172985.8	ENSG00000173064.6	ENSG00000173226.12	ENSG00000173432.6
ENSG00000173511.5	ENSG00000173692.8	ENSG00000174194.11	ENSG00000174652.13
ENSG00000175764.10	ENSG00000175899.10	ENSG00000176155.14	ENSG00000177030.12
ENSG00000177917.6	ENSG00000178449.4	ENSG00000178665.10	ENSG00000178802.13
ENSG00000178966.11	ENSG00000179774.7	ENSG00000179978.10	ENSG00000180185.7
ENSG00000180432.4	ENSG00000180481.6	ENSG00000180881.15	ENSG00000181315.6
ENSG00000182704.6	ENSG00000182771.13	ENSG00000182903.11	ENSG00000183020.9
ENSG00000183208.8	ENSG00000183248.7	ENSG00000183354.7	ENSG00000183423.7
ENSG00000184014.3	ENSG00000184110.10	ENSG00000184156.11	ENSG00000184274.3
ENSG00000184471.6	ENSG00000184517.7	ENSG00000184939.11	ENSG00000185344.9
ENSG00000186160.4	ENSG00000186470.9	ENSG00000186889.5	ENSG00000186907.3
ENSG00000187193.8	ENSG00000187954.8	ENSG00000188659.5	ENSG00000188906.9

	ENSG00000189280.3	ENSG00000189362.7	ENSG00000196345.8	ENSG00000196458.6
	ENSG00000196576.10	ENSG00000196712.12	ENSG00000196743.4	ENSG00000196814.10
	ENSG00000196843.11	ENSG00000197124.7	ENSG00000197146.2	ENSG00000197375.8
	ENSG00000197465.9	ENSG00000197498.8	ENSG00000197646.6	ENSG00000197747.4
	ENSG00000197885.6	ENSG00000197971.10	ENSG00000198198.9	ENSG00000198373.8
	ENSG00000198399.10	ENSG00000204301.5	ENSG00000204305.9	ENSG00000204444.6
	ENSG00000204482.6	ENSG00000204954.5	ENSG00000204959.3	ENSG00000204977.5
	ENSG00000205771.2	ENSG00000205822.6	ENSG00000213373.3	ENSG00000213626.7
	ENSG00000213753.6	ENSG00000213830.3	ENSG00000214982.6	ENSG00000215712.6
	ENSG00000215861.4	ENSG00000216775.2	ENSG00000221990.2	ENSG00000223496.1
	ENSG00000225398.2	ENSG00000225492.2	ENSG00000225784.5	ENSG00000225851.1
	ENSG00000226133.1	ENSG00000226314.3	ENSG00000227938.1	ENSG00000228008.1
	ENSG00000228307.1	ENSG00000228789.2	ENSG00000229390.1	ENSG00000230013.1
	ENSG00000230869.1	ENSG00000231360.2	ENSG00000231925.7	ENSG00000232063.1
	ENSG00000232259.1	ENSG00000233232.2	ENSG00000233594.2	ENSG00000234112.2
	ENSG00000234231.2	ENSG00000235098.4	ENSG00000235117.2	ENSG00000235374.1
	ENSG00000236233.3	ENSG00000236624.4	ENSG00000237510.3	ENSG00000237973.1
	ENSG00000238035.4	ENSG00000238243.2	ENSG00000239473.1	ENSG00000240038.2
	ENSG00000240563.1	ENSG00000242193.5	ENSG00000242441.3	ENSG00000242574.4
	ENSG00000243480.3	ENSG00000244754.4	ENSG00000245937.3	ENSG00000248098.6
	ENSG00000250138.3	ENSG00000250334.1	ENSG00000250571.2	ENSG00000250903.4
	ENSG00000251580.1	ENSG00000253317.1	ENSG00000253816.2	ENSG00000254810.1
	ENSG00000256223.1	ENSG00000256433.1	ENSG00000257210.1	ENSG00000258090.1
	ENSG00000258818.2	ENSG00000259258.1	ENSG00000259539.1	ENSG00000259959.1
	ENSG00000260006.1	ENSG00000260105.2	ENSG00000260274.1	ENSG00000260916.1
	ENSG00000261423.1	ENSG00000261556.4	ENSG00000261672.1	ENSG00000262165.1
	ENSG00000263179.1	ENSG00000263931.1	ENSG00000267922.1	ENSG00000267939.1
	ENSG00000268912.1	ENSG00000269190.1	ENSG00000269430.1	ENSG00000270081.1
	ENSG00000270424.1	ENSG00000271239.1	ENSG00000271623.1	ENSG00000271824.1
	ENSG00000271912.1	ENSG00000272129.1	ENSG00000272138.1	ENSG00000272462.2
	ENSG00000272644.1	ENSG00000272810.1		
	ENSG00000001561.6	ENSG00000006432.11	ENSG00000009724.12	ENSG00000013725.10
	ENSG00000040531.10	ENSG00000078053.12	ENSG00000085982.9	ENSG00000089597.12
	ENSG00000092036.12	ENSG00000101255.6	ENSG00000101639.14	ENSG00000102996.4
**Brain Amygdala**	ENSG00000106993.7	ENSG00000108272.9	ENSG00000110455.9	ENSG00000111880.11
	ENSG00000112796.5	ENSG00000113119.8	ENSG00000115234.6	ENSG00000115507.5
	ENSG00000116774.7	ENSG00000122547.6	ENSG00000124275.10	ENSG00000126773.8
	ENSG00000128591.11	ENSG00000135643.4	ENSG00000136059.10	ENSG00000138587.5
	ENSG00000138767.8	ENSG00000140265.8	ENSG00000141127.10	ENSG00000142188.12
	ENSG00000142856.12	ENSG00000143107.4	ENSG00000143194.8	ENSG00000144026.7
	ENSG00000144028.10	ENSG00000149451.13	ENSG00000151327.8	ENSG00000152952.7
	ENSG00000154252.11	ENSG00000154917.6	ENSG00000156304.10	ENSG00000160404.13
	ENSG00000160886.9	ENSG00000161395.8	ENSG00000162039.10	ENSG00000162298.12
	ENSG00000163682.11	ENSG00000163888.3	ENSG00000164744.8	ENSG00000164880.11
	ENSG00000164904.11	ENSG00000165406.11	ENSG00000167208.10	ENSG00000167670.11
	ENSG00000167700.4	ENSG00000170525.14	ENSG00000170745.7	ENSG00000171130.13
	ENSG00000171790.11	ENSG00000173041.7	ENSG00000174136.7	ENSG00000174194.11
	ENSG00000174652.13	ENSG00000176998.3	ENSG00000178201.3	ENSG00000178209.10
	ENSG00000180113.11	ENSG00000180185.7	ENSG00000180481.6	ENSG00000180878.2

	ENSG00000181754.6	ENSG00000182326.10	ENSG00000183048.7	ENSG00000183833.12
	ENSG00000186960.6	ENSG00000187135.7	ENSG00000196139.7	ENSG00000196993.4
	ENSG00000197343.6	ENSG00000205822.6	ENSG00000215114.3	ENSG00000221843.2
	ENSG00000223345.3	ENSG00000225241.3	ENSG00000235098.4	ENSG00000236624.4
	ENSG00000240356.2	ENSG00000243244.1	ENSG00000249096.2	ENSG00000253967.1
	ENSG00000254648.1	ENSG00000255537.1	ENSG00000255595.1	ENSG00000256968.1
	ENSG00000259262.1	ENSG00000260804.2	ENSG00000261098.1	ENSG00000261504.1
	ENSG00000261556.4	ENSG00000261770.1	ENSG00000262165.1	ENSG00000266402.2
	ENSG00000267264.1	ENSG00000268927.1	ENSG00000269202.1	ENSG00000269416.1
	ENSG00000270996.1	ENSG00000271361.1	ENSG00000271743.1	ENSG00000272462.2
	ENSG00000273011.1	ENSG00000273281.1		
**Brain Anterior cingulate**	ENSG00000006118.10	ENSG00000018236.10	ENSG00000027001.7	ENSG00000067248.5
	ENSG00000067836.8	ENSG00000086200.12	ENSG00000091262.10	ENSG00000092036.12
	ENSG00000099800.3	ENSG00000100209.5	ENSG00000100330.11	ENSG00000100373.5
	ENSG00000101104.8	ENSG00000101639.14	ENSG00000103460.12	ENSG00000105426.10
	ENSG00000105519.8	ENSG00000107362.9	ENSG00000108272.9	ENSG00000108278.7
	ENSG00000110455.9	ENSG00000111237.14	ENSG00000114019.10	ENSG00000117450.9
	ENSG00000117834.8	ENSG00000123080.6	ENSG00000124275.10	ENSG00000125337.12
	ENSG00000125814.13	ENSG00000125835.13	ENSG00000126773.8	ENSG00000128039.6
	ENSG00000128218.7	ENSG00000128886.7	ENSG00000131435.8	ENSG00000132481.2
	ENSG00000135972.4	ENSG00000137312.10	ENSG00000137411.12	ENSG00000138193.10
	ENSG00000138400.8	ENSG00000140015.15	ENSG00000140829.7	ENSG00000141140.12
	ENSG00000142065.9	ENSG00000143194.8	ENSG00000143921.6	ENSG00000144021.2
	ENSG00000144857.10	ENSG00000146112.7	ENSG00000148300.7	ENSG00000150275.13
	ENSG00000150459.8	ENSG00000152022.7	ENSG00000157837.11	ENSG00000158987.15
	ENSG00000160097.11	ENSG00000160716.4	ENSG00000161055.3	ENSG00000161798.6
	ENSG00000162039.10	ENSG00000162520.10	ENSG00000162994.11	ENSG00000163083.5
	ENSG00000163959.5	ENSG00000164880.11	ENSG00000165646.7	ENSG00000167384.6
	ENSG00000167842.11	ENSG00000169241.13	ENSG00000169515.5	ENSG00000169609.9
	ENSG00000169994.14	ENSG00000170837.2	ENSG00000170899.6	ENSG00000171130.13
	ENSG00000171612.6	ENSG00000171853.11	ENSG00000171984.10	ENSG00000173200.8
	ENSG00000174194.11	ENSG00000174226.4	ENSG00000174652.13	ENSG00000176386.4
	ENSG00000176998.3	ENSG00000179604.8	ENSG00000180185.7	ENSG00000180481.6
	ENSG00000183161.3	ENSG00000183291.11	ENSG00000186300.7	ENSG00000186470.9
	ENSG00000187953.6	ENSG00000188388.9	ENSG00000188659.5	ENSG00000196247.7
	ENSG00000196503.2	ENSG00000197165.6	ENSG00000197948.6	ENSG00000198885.5
	ENSG00000203602.1	ENSG00000203697.7	ENSG00000204128.5	ENSG00000204138.8
	ENSG00000204681.6	ENSG00000204792.2	ENSG00000204793.4	ENSG00000205078.5
	ENSG00000215861.4	ENSG00000225241.3	ENSG00000225398.2	ENSG00000229150.1
	ENSG00000232063.1	ENSG00000232987.1	ENSG00000233967.2	ENSG00000235098.4
	ENSG00000235290.1	ENSG00000236155.2	ENSG00000236624.4	ENSG00000237560.1
	ENSG00000240038.2	ENSG00000241043.1	ENSG00000241644.2	ENSG00000243244.1
	ENSG00000245937.3	ENSG00000248971.2	ENSG00000249694.2	ENSG00000251575.2
	ENSG00000253203.2	ENSG00000253520.1	ENSG00000253540.1	ENSG00000259539.1
	ENSG00000261064.1	ENSG00000261556.4	ENSG00000261788.1	ENSG00000266490.1
	ENSG00000267612.1	ENSG00000267623.2	ENSG00000268442.1	ENSG00000269979.1
	ENSG00000270081.1	ENSG00000271344.1	ENSG00000272216.1	ENSG00000272274.1
	ENSG00000001460.13	ENSG00000001629.5	ENSG00000005844.13	ENSG00000005955.8
	ENSG00000033867.12	ENSG00000037042.8	ENSG00000050748.13	ENSG00000066117.10

Brain

ENSG00000071794.11	ENSG00000072803.13	ENSG00000075429.4	ENSG00000087301.4
ENSG00000090520.6	ENSG00000091262.10	ENSG00000092036.12	ENSG00000093167.13
ENSG00000099800.3	ENSG00000100263.9	ENSG00000100276.9	ENSG00000100596.2
ENSG00000101132.5	ENSG00000101204.11	ENSG00000101255.6	ENSG00000101574.10
ENSG00000104818.14	ENSG00000105341.14	ENSG00000108270.6	ENSG00000108272.9
ENSG00000111328.2	ENSG00000111664.6	ENSG00000112706.7	ENSG00000112796.5
ENSG00000113262.10	ENSG00000116791.9	ENSG00000117226.7	ENSG00000117477.8
ENSG00000117834.8	ENSG00000120008.11	ENSG00000120586.4	ENSG00000120949.10
ENSG00000124275.10	ENSG00000125885.9	ENSG00000125895.5	ENSG00000127054.14
ENSG00000128581.11	ENSG00000130035.2	ENSG00000133983.10	ENSG00000135414.5
ENSG00000136051.9	ENSG00000136235.11	ENSG00000136682.10	ENSG00000136816.11
ENSG00000137078.4	ENSG00000137275.9	ENSG00000137500.5	ENSG00000137558.3
ENSG00000138029.9	ENSG00000138119.12	ENSG00000138152.7	ENSG00000140905.5
ENSG00000141012.8	ENSG00000141013.10	ENSG00000141524.11	ENSG00000142065.9
ENSG00000143036.12	ENSG00000143194.8	ENSG00000143751.9	ENSG00000144028.10
ENSG00000145949.8	ENSG00000146540.10	ENSG00000151388.6	ENSG00000151746.9
ENSG00000153291.11	ENSG00000156711.12	ENSG00000157578.9	ENSG00000158747.9
ENSG00000160867.10	ENSG00000161640.11	ENSG00000162039.10	ENSG00000162104.5
ENSG00000162722.8	ENSG00000162994.11	ENSG00000164880.11	ENSG00000164967.5
ENSG00000165269.8	ENSG00000165646.7	ENSG00000165650.7	ENSG00000165730.10
ENSG00000167074.10	ENSG00000167130.13	ENSG00000167377.13	ENSG00000167792.7
ENSG00000167842.11	ENSG00000168040.4	ENSG00000168397.12	ENSG00000168787.4
ENSG00000168994.9	ENSG00000170175.6	ENSG00000170606.9	ENSG00000170903.6
ENSG00000171130.13	ENSG00000171928.9	ENSG00000171943.7	ENSG00000172062.12
ENSG00000172752.10	ENSG00000174059.12	ENSG00000174194.11	ENSG00000174226.4
ENSG00000174374.9	ENSG00000174473.11	ENSG00000174652.13	ENSG00000176393.6
ENSG00000176998.3	ENSG00000178802.13	ENSG00000179241.8	ENSG00000179886.4
ENSG00000180185.7	ENSG00000180481.6	ENSG00000180953.7	ENSG00000183067.5
ENSG00000183748.4	ENSG00000184009.5	ENSG00000184293.3	ENSG00000184602.5
ENSG00000186470.9	ENSG00000186952.10	ENSG00000188089.9	ENSG00000188388.9
ENSG00000188404.4	ENSG00000188610.8	ENSG00000188659.5	ENSG00000188897.4
ENSG00000189127.3	ENSG00000196431.3	ENSG00000196458.6	ENSG00000197483.8
ENSG00000197646.6	ENSG00000198691.7	ENSG00000203896.5	ENSG00000204252.8
ENSG00000204381.7	ENSG00000204520.8	ENSG00000204681.6	ENSG00000205822.6
ENSG00000213658.6	ENSG00000214102.3	ENSG00000215712.6	ENSG00000223496.1
ENSG00000225156.2	ENSG00000225398.2	ENSG00000228340.1	ENSG00000231861.1
ENSG00000231971.1	ENSG00000231976.3	ENSG00000232699.2	ENSG00000233232.2
ENSG00000234147.1	ENSG00000236297.1	ENSG00000236624.4	ENSG00000243244.1
ENSG00000245937.3	ENSG00000247157.2	ENSG00000248243.1	ENSG00000248587.2
ENSG00000250786.1	ENSG00000251575.2	ENSG00000254054.2	ENSG00000254057.1
ENSG00000258483.1	ENSG00000258711.2	ENSG00000258769.1	ENSG00000259539.1
ENSG00000260979.1	ENSG00000261556.4	ENSG00000261770.1	ENSG00000262165.1
ENSG00000263063.1	ENSG00000263627.1	ENSG00000268358.1	ENSG00000269908.1
ENSG00000270081.1	ENSG00000270445.1	ENSG00000271788.1	ENSG00000272375.1
ENSG00000272583.1	ENSG00000272899.1	ENSG00000273117.1	
ENSG00000004864.9	ENSG00000008283.11	ENSG00000010319.2	ENSG00000018510.8
ENSG00000025434.14	ENSG00000047188.11	ENSG00000048392.7	ENSG00000054219.9
ENSG00000054392.8	ENSG00000055955.11	ENSG00000058453.12	ENSG00000067221.9
ENSG00000075239.9	ENSG00000077348.4	ENSG00000080573.6	ENSG00000086232.8

ENSG00000086289.7	ENSG00000087206.12	ENSG00000090539.11	ENSG00000091490.6
ENSG00000092036.12	ENSG00000097007.13	ENSG00000099800.3	ENSG00000099956.13
ENSG00000100154.10	ENSG00000100354.16	ENSG00000100461.13	ENSG00000101204.11
ENSG00000104907.8	ENSG00000105664.6	ENSG00000105793.11	ENSG00000107077.13
ENSG00000107854.5	ENSG00000107874.6	ENSG00000108264.12	ENSG00000108272.9
ENSG00000108559.7	ENSG00000108669.12	ENSG00000108785.7	ENSG00000109158.6
ENSG00000109943.4	ENSG00000110906.8	ENSG00000111664.6	ENSG00000112175.6
ENSG00000112796.5	ENSG00000113231.9	ENSG00000113240.8	ENSG00000114125.9
ENSG00000115325.9	ENSG00000115484.10	ENSG00000115677.12	ENSG00000116745.6
ENSG00000117174.6	ENSG00000117226.7	ENSG00000117533.10	ENSG00000117593.8
ENSG00000117601.9	ENSG00000117899.6	ENSG00000118816.5	ENSG00000119487.12
ENSG00000119599.12	ENSG00000119714.6	ENSG00000120451.6	ENSG00000122376.7
ENSG00000122694.11	ENSG00000122729.14	ENSG00000124570.13	ENSG00000126773.8
ENSG00000126804.9	ENSG00000127054.14	ENSG00000129467.9	ENSG00000131389.12
ENSG00000131697.13	ENSG00000132970.8	ENSG00000134802.13	ENSG00000134874.13
ENSG00000136114.11	ENSG00000136235.11	ENSG00000136717.10	ENSG00000137070.13
ENSG00000137101.8	ENSG00000137103.12	ENSG00000137161.12	ENSG00000137642.8
ENSG00000137996.8	ENSG00000138449.6	ENSG00000138769.6	ENSG00000139725.3
ENSG00000139746.11	ENSG00000140326.8	ENSG00000141013.10	ENSG00000141127.10
ENSG00000141433.8	ENSG00000144580.9	ENSG00000145901.10	ENSG00000148843.9
ENSG00000149418.6	ENSG00000151748.10	ENSG00000152465.13	ENSG00000153560.7
ENSG00000156502.9	ENSG00000156521.9	ENSG00000156564.8	ENSG00000157911.5
ENSG00000159128.10	ENSG00000160294.6	ENSG00000160401.10	ENSG00000160404.13
ENSG00000160716.4	ENSG00000162076.8	ENSG00000163029.11	ENSG00000163354.10
ENSG00000164318.13	ENSG00000164880.11	ENSG00000164972.8	ENSG00000165113.8
ENSG00000165124.13	ENSG00000165181.12	ENSG00000165626.12	ENSG00000165646.7
ENSG00000165650.7	ENSG00000165730.10	ENSG00000166845.9	ENSG00000167642.8
ENSG00000167670.11	ENSG00000167720.8	ENSG00000167723.10	ENSG00000168228.10
ENSG00000168411.9	ENSG00000169136.4	ENSG00000169609.9	ENSG00000170502.8
ENSG00000170915.8	ENSG00000171864.4	ENSG00000172661.13	ENSG00000173226.12
ENSG00000174194.11	ENSG00000174628.12	ENSG00000174652.13	ENSG00000174886.8
ENSG00000176155.14	ENSG00000176659.5	ENSG00000176723.5	ENSG00000176915.10
ENSG00000176956.8	ENSG00000177191.2	ENSG00000178202.8	ENSG00000179294.5
ENSG00000179314.9	ENSG00000179978.10	ENSG00000180113.11	ENSG00000180185.7
ENSG00000180481.6	ENSG00000181045.10	ENSG00000182752.8	ENSG00000182870.8
ENSG00000183628.8	ENSG00000184389.8	ENSG00000184990.8	ENSG00000185019.12
ENSG00000185088.8	ENSG00000185340.11	ENSG00000185986.10	ENSG00000186446.7
ENSG00000186470.9	ENSG00000187145.10	ENSG00000188277.8	ENSG00000188659.5
ENSG00000196653.7	ENSG00000198553.4	ENSG00000198690.5	ENSG00000198826.6
ENSG00000198885.5	ENSG00000203666.8	ENSG00000203843.3	ENSG00000204092.2
ENSG00000204356.7	ENSG00000204381.7	ENSG00000204516.5	ENSG00000204856.7
ENSG00000204962.4	ENSG00000205085.4	ENSG00000205693.3	ENSG00000206190.7
ENSG00000213023.5	ENSG00000213753.6	ENSG00000214046.4	ENSG00000214820.3
ENSG00000215712.6	ENSG00000215861.4	ENSG00000216740.2	ENSG00000216775.2
ENSG00000217442.3	ENSG00000223496.1	ENSG00000224050.1	ENSG00000225241.3
ENSG00000225784.5	ENSG00000226070.1	ENSG00000227455.2	ENSG00000229391.3
ENSG00000231205.7	ENSG00000231861.1	ENSG00000235098.4	ENSG00000236624.4
ENSG00000239388.4	ENSG00000240204.2	ENSG00000243480.3	ENSG00000249694.2
ENSG00000249915.3	ENSG00000251189.1	ENSG00000251580.1	ENSG00000253175.1

	ENSG00000253395.1	ENSG00000253853.1	ENSG00000254651.1	ENSG00000254667.1
	ENSG00000258591.2	ENSG00000258667.1	ENSG00000259539.1	ENSG00000259845.1
	ENSG00000260382.1	ENSG00000260769.1	ENSG00000261556.4	ENSG00000261628.1
	ENSG00000261770.1	ENSG00000263179.1	ENSG00000266714.2	ENSG00000267264.1
	ENSG00000267623.2	ENSG00000268442.1	ENSG00000270081.1	ENSG00000270178.1
	ENSG00000271623.1	ENSG00000272462.2	ENSG00000273230.1	ENSG00000273454.1
	ENSG00000003056.3	ENSG00000004864.9	ENSG00000004866.14	ENSG00000011021.17
	ENSG00000022567.5	ENSG00000023171.10	ENSG00000028137.12	ENSG00000029725.12
	ENSG00000050165.13	ENSG00000054118.9	ENSG00000068781.16	ENSG00000070269.9
	ENSG00000076513.12	ENSG00000077348.4	ENSG00000081181.3	ENSG00000084090.9
	ENSG00000085982.9	ENSG00000087157.14	ENSG00000088836.8	ENSG00000090861.11
	ENSG00000091490.6	ENSG00000092036.12	ENSG00000095564.9	ENSG00000099953.5
	ENSG00000099956.13	ENSG00000100276.9	ENSG00000100461.13	ENSG00000101310.10
	ENSG00000101460.8	ENSG00000103423.9	ENSG00000104938.12	ENSG00000105664.6
	ENSG00000105793.11	ENSG00000105854.8	ENSG00000107077.13	ENSG00000107282.5
	ENSG00000107611.10	ENSG00000107679.10	ENSG00000107854.5	ENSG00000107874.6
	ENSG00000107890.12	ENSG00000108264.12	ENSG00000108272.9	ENSG00000108785.7
	ENSG00000108786.6	ENSG00000108799.8	ENSG00000109736.10	ENSG00000110756.13
	ENSG00000110906.8	ENSG00000111664.6	ENSG00000112584.9	ENSG00000112667.8
	ENSG00000112796.5	ENSG00000113231.9	ENSG00000115170.9	ENSG00000115289.8
	ENSG00000116690.7	ENSG00000117174.6	ENSG00000117226.7	ENSG00000117682.12
	ENSG00000118873.11	ENSG00000119231.6	ENSG00000119321.4	ENSG00000119714.6
	ENSG00000120314.14	ENSG00000120451.6	ENSG00000120539.10	ENSG00000120685.15
	ENSG00000121742.11	ENSG00000122694.11	ENSG00000122729.14	ENSG00000123143.8
	ENSG00000123178.10	ENSG00000123219.8	ENSG00000124145.5	ENSG00000124275.10
**Brain Cerebellum**	ENSG00000124570.13	ENSG00000124588.15	ENSG00000124614.9	ENSG00000125170.6
	ENSG00000125827.4	ENSG00000126746.13	ENSG00000126773.8	ENSG00000126777.13
	ENSG00000127054.14	ENSG00000129158.6	ENSG00000129467.9	ENSG00000130684.9
	ENSG00000131389.12	ENSG00000131470.10	ENSG00000131558.10	ENSG00000132517.10
	ENSG00000132849.14	ENSG00000133816.9	ENSG00000133983.10	ENSG00000134830.3
	ENSG00000134987.7	ENSG00000135144.3	ENSG00000135905.14	ENSG00000136235.11
	ENSG00000136247.10	ENSG00000136842.9	ENSG00000136897.6	ENSG00000136999.4
	ENSG00000137101.8	ENSG00000137275.9	ENSG00000137310.7	ENSG00000137843.7
	ENSG00000137992.10	ENSG00000138085.12	ENSG00000138316.6	ENSG00000138606.15
	ENSG00000139343.6	ENSG00000139350.7	ENSG00000139926.11	ENSG00000140263.9
	ENSG00000140326.8	ENSG00000140400.10	ENSG00000140691.12	ENSG00000141013.10
	ENSG00000141294.5	ENSG00000141441.11	ENSG00000141503.11	ENSG00000142185.12
	ENSG00000143799.8	ENSG00000145901.10	ENSG00000146090.11	ENSG00000147481.9
	ENSG00000147535.12	ENSG00000147573.12	ENSG00000150722.6	ENSG00000151657.7
	ENSG00000152465.13	ENSG00000153291.11	ENSG00000153914.11	ENSG00000155066.11
	ENSG00000156502.9	ENSG00000156564.8	ENSG00000157837.11	ENSG00000157895.7
	ENSG00000158079.10	ENSG00000158220.9	ENSG00000158717.6	ENSG00000159128.10
	ENSG00000160190.9	ENSG00000160957.8	ENSG00000161217.7	ENSG00000163093.7
	ENSG00000163682.11	ENSG00000163788.9	ENSG00000163882.5	ENSG00000163902.7
	ENSG00000164107.7	ENSG00000164880.11	ENSG00000164967.5	ENSG00000165215.5
	ENSG00000165474.5	ENSG00000165646.7	ENSG00000165650.7	ENSG00000165730.10
	ENSG00000165837.7	ENSG00000166311.5	ENSG00000166869.2	ENSG00000167695.10
	ENSG00000167771.5	ENSG00000169020.5	ENSG00000170175.6	ENSG00000170275.10
	ENSG00000170802.11	ENSG00000170915.8	ENSG00000171130.13	ENSG00000171864.4

	ENSG00000171867.12	ENSG00000171928.9	ENSG00000172493.16	ENSG00000172661.13
	ENSG00000172766.14	ENSG00000173065.9	ENSG00000173212.4	ENSG00000173852.9
	ENSG00000174151.10	ENSG00000174194.11	ENSG00000174233.7	ENSG00000174652.13
	ENSG00000174792.6	ENSG00000175920.11	ENSG00000176155.14	ENSG00000176386.4
	ENSG00000176476.4	ENSG00000176659.5	ENSG00000176723.5	ENSG00000176890.11
	ENSG00000177191.2	ENSG00000178631.7	ENSG00000179314.9	ENSG00000180113.11
	ENSG00000180185.7	ENSG00000180370.6	ENSG00000180376.12	ENSG00000180481.6
	ENSG00000181220.11	ENSG00000181227.2	ENSG00000182568.12	ENSG00000183117.13
	ENSG00000183281.10	ENSG00000183617.4	ENSG00000183722.7	ENSG00000184305.10
	ENSG00000184389.8	ENSG00000184524.5	ENSG00000184995.6	ENSG00000185274.7
	ENSG00000185340.11	ENSG00000186198.3	ENSG00000186283.9	ENSG00000186889.5
	ENSG00000186907.3	ENSG00000187535.9	ENSG00000187726.4	ENSG00000187984.8
	ENSG00000188064.5	ENSG00000188242.4	ENSG00000188321.9	ENSG00000188511.8
	ENSG00000188629.7	ENSG00000188659.5	ENSG00000188735.8	ENSG00000189212.8
	ENSG00000196636.7	ENSG00000196993.4	ENSG00000197375.8	ENSG00000197747.4
	ENSG00000198642.5	ENSG00000198690.5	ENSG00000198721.8	ENSG00000198885.5
	ENSG00000198954.4	ENSG00000203667.5	ENSG00000204092.2	ENSG00000204381.7
	ENSG00000204420.4	ENSG00000204516.5	ENSG00000204970.5	ENSG00000204977.5
	ENSG00000205571.8	ENSG00000213722.4	ENSG00000213927.3	ENSG00000214433.4
	ENSG00000214982.6	ENSG00000215187.5	ENSG00000215712.6	ENSG00000215861.4
	ENSG00000215915.5	ENSG00000216775.2	ENSG00000217702.1	ENSG00000223496.1
	ENSG00000223768.1	ENSG00000224051.2	ENSG00000225241.3	ENSG00000225784.5
	ENSG00000227999.1	ENSG00000231205.7	ENSG00000233196.2	ENSG00000233232.2
	ENSG00000233396.3	ENSG00000234840.1	ENSG00000235098.4	ENSG00000236624.4
	ENSG00000236844.1	ENSG00000237176.3	ENSG00000237541.3	ENSG00000239648.1
	ENSG00000239670.1	ENSG00000240204.2	ENSG00000241043.1	ENSG00000245937.3
	ENSG00000246640.1	ENSG00000248533.1	ENSG00000251323.2	ENSG00000251580.1
	ENSG00000253696.2	ENSG00000254319.1	ENSG00000255455.2	ENSG00000255748.1
	ENSG00000256424.1	ENSG00000259539.1	ENSG00000259577.1	ENSG00000259658.3
	ENSG00000259845.1	ENSG00000260382.1	ENSG00000260743.1	ENSG00000260769.1
	ENSG00000260916.1	ENSG00000261377.1	ENSG00000261455.1	ENSG00000261556.4
	ENSG00000261628.1	ENSG00000262165.1	ENSG00000263050.1	ENSG00000263179.1
	ENSG00000266490.1	ENSG00000267053.2	ENSG00000267264.1	ENSG00000267472.1
	ENSG00000267689.1	ENSG00000267939.1	ENSG00000269489.1	ENSG00000269514.1
	ENSG00000270081.1	ENSG00000270346.1	ENSG00000270614.1	ENSG00000270750.1
	ENSG00000271361.1	ENSG00000271743.1	ENSG00000272462.2	ENSG00000272583.1
	ENSG00000272709.1	ENSG00000272899.1	ENSG00000273230.1	
	ENSG00000003056.3	ENSG00000039319.12	ENSG00000047188.11	ENSG00000053372.4
	ENSG00000054219.9	ENSG00000064703.7	ENSG00000071626.12	ENSG00000075131.5
	ENSG00000075790.6	ENSG00000083099.6	ENSG00000089159.11	ENSG00000092036.12
	ENSG00000092421.12	ENSG00000094975.9	ENSG00000096093.10	ENSG00000099800.3
	ENSG00000100239.11	ENSG00000100249.4	ENSG00000100612.9	ENSG00000101197.8
	ENSG00000101224.13	ENSG00000101639.14	ENSG00000103023.7	ENSG00000104093.9
	ENSG00000105519.8	ENSG00000107854.5	ENSG00000108100.13	ENSG00000108176.10
	ENSG00000108272.9	ENSG00000108439.5	ENSG00000108559.7	ENSG00000108953.12
	ENSG00000109576.9	ENSG00000111237.14	ENSG00000111664.6	ENSG00000111802.9
**Brain Cortex**	ENSG00000112852.4	ENSG00000113318.9	ENSG00000114446.4	ENSG00000115207.9
	ENSG00000116652.5	ENSG00000116688.12	ENSG00000117281.11	ENSG00000117834.8
	ENSG00000117899.6	ENSG00000117983.13	ENSG00000120669.11	ENSG00000120837.3

	ENSG00000120910.10	ENSG00000124275.10	ENSG00000125170.6	ENSG00000126215.9
	ENSG00000126247.6	ENSG00000126602.6	ENSG00000126773.8	ENSG00000128218.7
	ENSG00000128578.5	ENSG00000131944.5	ENSG00000133983.10	ENSG00000135164.14
	ENSG00000135709.8	ENSG00000135845.5	ENSG00000136235.11	ENSG00000136319.7
	ENSG00000136861.13	ENSG00000138316.6	ENSG00000138472.6	ENSG00000138600.5
	ENSG00000138615.4	ENSG00000139193.3	ENSG00000139263.7	ENSG00000139620.8
	ENSG00000139624.8	ENSG00000142449.8	ENSG00000143995.15	ENSG00000144580.9
	ENSG00000145194.13	ENSG00000148843.9	ENSG00000149633.7	ENSG00000150873.7
	ENSG00000151304.5	ENSG00000151348.9	ENSG00000152954.7	ENSG00000153291.11
	ENSG00000154767.10	ENSG00000156414.14	ENSG00000157837.11	ENSG00000160838.9
	ENSG00000161055.3	ENSG00000161149.7	ENSG00000162994.11	ENSG00000163072.10
	ENSG00000163563.7	ENSG00000163827.8	ENSG00000164251.4	ENSG00000164880.11
	ENSG00000165282.9	ENSG00000165646.7	ENSG00000165650.7	ENSG00000166848.5
	ENSG00000166947.7	ENSG00000167333.8	ENSG00000167384.6	ENSG00000167535.3
	ENSG00000167842.11	ENSG00000168297.11	ENSG00000168916.11	ENSG00000169515.5
	ENSG00000169609.9	ENSG00000170175.6	ENSG00000170893.3	ENSG00000170906.11
	ENSG00000170915.8	ENSG00000171130.13	ENSG00000171208.5	ENSG00000171943.7
	ENSG00000173852.9	ENSG00000174194.11	ENSG00000174373.11	ENSG00000174652.13
	ENSG00000175115.7	ENSG00000176155.14	ENSG00000176386.4	ENSG00000177283.4
	ENSG00000177302.10	ENSG00000178021.9	ENSG00000178773.10	ENSG00000180113.11
	ENSG00000180185.7	ENSG00000180481.6	ENSG00000180953.7	ENSG00000183527.7
	ENSG00000184454.6	ENSG00000184990.8	ENSG00000185088.8	ENSG00000185523.6
	ENSG00000185532.10	ENSG00000185730.3	ENSG00000186026.6	ENSG00000186446.7
	ENSG00000186470.9	ENSG00000188321.9	ENSG00000188388.9	ENSG00000189091.8
	ENSG00000196418.8	ENSG00000196993.4	ENSG00000197016.7	ENSG00000197183.8
	ENSG00000197467.9	ENSG00000197483.8	ENSG00000197712.7	ENSG00000198373.8
	ENSG00000198945.3	ENSG00000203684.5	ENSG00000203999.4	ENSG00000204308.6
	ENSG00000204963.4	ENSG00000204978.2	ENSG00000205822.6	ENSG00000213471.4
	ENSG00000213871.3	ENSG00000214043.3	ENSG00000214297.3	ENSG00000214562.9
	ENSG00000214597.4	ENSG00000215790.2	ENSG00000215861.4	ENSG00000221819.2
	ENSG00000221990.2	ENSG00000223496.1	ENSG00000223804.1	ENSG00000224389.4
	ENSG00000224683.1	ENSG00000225385.3	ENSG00000225398.2	ENSG00000226070.1
	ENSG00000226191.3	ENSG00000227201.2	ENSG00000228789.2	ENSG00000229150.1
	ENSG00000229391.3	ENSG00000230133.1	ENSG00000230869.1	ENSG00000231466.2
	ENSG00000233196.2	ENSG00000233961.1	ENSG00000234515.1	ENSG00000235098.4
	ENSG00000236624.4	ENSG00000237510.3	ENSG00000241043.1	ENSG00000242715.3
	ENSG00000243244.1	ENSG00000244155.1	ENSG00000248593.3	ENSG00000250786.1
	ENSG00000251580.1	ENSG00000253161.1	ENSG00000255853.1	ENSG00000256494.1
	ENSG00000257061.1	ENSG00000258181.1	ENSG00000259905.1	ENSG00000261121.1
	ENSG00000261455.1	ENSG00000261556.4	ENSG00000261794.1	ENSG00000266912.1
	ENSG00000267692.1	ENSG00000268266.1	ENSG00000270346.1	ENSG00000270604.1
	ENSG00000271109.1	ENSG00000271234.1	ENSG00000272097.1	ENSG00000272542.1
	ENSG00000272583.1	ENSG00000272828.1	ENSG00000272899.1	ENSG00000272922.1
	ENSG00000273230.1			
**Brain Frontal Cortex**	ENSG00000044459.10	ENSG00000044574.7	ENSG00000054219.9	ENSG00000055955.11
	ENSG00000065060.12	ENSG00000071794.11	ENSG00000071994.6	ENSG00000074370.13
	ENSG00000077420.11	ENSG00000083099.6	ENSG00000099800.3	ENSG00000100461.13
	ENSG00000100612.9	ENSG00000101004.10	ENSG00000101104.8	ENSG00000101166.11
	ENSG00000101639.14	ENSG00000102606.13	ENSG00000105341.14	ENSG00000105668.3

	ENSG00000105717.9	ENSG00000106028.6	ENSG00000107623.4	ENSG00000108272.9
	ENSG00000108379.5	ENSG00000108799.8	ENSG00000111664.6	ENSG00000112796.5
	ENSG00000115282.15	ENSG00000117174.6	ENSG00000117899.6	ENSG00000119986.6
	ENSG00000120008.11	ENSG00000120910.10	ENSG00000121289.13	ENSG00000121335.10
	ENSG00000121417.9	ENSG00000124275.10	ENSG00000124508.12	ENSG00000126773.8
	ENSG00000133247.9	ENSG00000133731.5	ENSG00000135775.9	ENSG00000136235.11
	ENSG00000136634.5	ENSG00000137225.8	ENSG00000138071.9	ENSG00000138400.8
	ENSG00000139624.8	ENSG00000140323.4	ENSG00000141140.12	ENSG00000143740.10
	ENSG00000144026.7	ENSG00000148143.8	ENSG00000152580.8	ENSG00000156398.8
	ENSG00000157693.10	ENSG00000158234.8	ENSG00000158406.2	ENSG00000159111.8
	ENSG00000160360.7	ENSG00000160867.10	ENSG00000161055.3	ENSG00000163069.8
	ENSG00000163576.13	ENSG00000165269.8	ENSG00000165646.7	ENSG00000166359.6
	ENSG00000166452.7	ENSG00000166676.10	ENSG00000167842.11	ENSG00000168005.4
	ENSG00000168404.8	ENSG00000169668.7	ENSG00000169914.5	ENSG00000170522.5
	ENSG00000172071.7	ENSG00000172935.8	ENSG00000174194.11	ENSG00000174652.13
	ENSG00000175463.7	ENSG00000176155.14	ENSG00000176386.4	ENSG00000176909.7
	ENSG00000176998.3	ENSG00000177990.7	ENSG00000180113.11	ENSG00000180185.7
	ENSG00000180481.6	ENSG00000180535.3	ENSG00000182771.13	ENSG00000183423.7
	ENSG00000185088.8	ENSG00000185532.10	ENSG00000186283.9	ENSG00000186470.9
	ENSG00000187624.7	ENSG00000187848.8	ENSG00000188566.7	ENSG00000196418.8
	ENSG00000197747.4	ENSG00000198246.7	ENSG00000198734.6	ENSG00000204381.7
	ENSG00000204520.8	ENSG00000204657.2	ENSG00000204677.6	ENSG00000205189.7
	ENSG00000205413.3	ENSG00000205683.7	ENSG00000205710.3	ENSG00000205838.8
	ENSG00000213625.4	ENSG00000213722.4	ENSG00000214043.3	ENSG00000215861.4
	ENSG00000216775.2	ENSG00000216895.4	ENSG00000223345.3	ENSG00000223496.1
	ENSG00000225241.3	ENSG00000226328.2	ENSG00000228022.1	ENSG00000228162.1
	ENSG00000230044.1	ENSG00000230156.2	ENSG00000233961.1	ENSG00000235098.4
	ENSG00000235475.1	ENSG00000237510.3	ENSG00000237560.1	ENSG00000240399.1
	ENSG00000243244.1	ENSG00000244041.3	ENSG00000251417.1	ENSG00000251580.1
	ENSG00000251595.3	ENSG00000251655.2	ENSG00000256073.2	ENSG00000259539.1
	ENSG00000259728.1	ENSG00000260091.1	ENSG00000261425.1	ENSG00000261556.4
	ENSG00000261617.1	ENSG00000261705.1	ENSG00000262031.1	ENSG00000267053.2
	ENSG00000267264.1	ENSG00000267939.1	ENSG00000270480.1	ENSG00000272005.1
	ENSG00000272221.1	ENSG00000272274.1	ENSG00000272417.1	ENSG00000272828.1
	ENSG00000273270.1			
	ENSG00000005955.8	ENSG00000009335.13	ENSG00000037042.8	ENSG00000040531.10
	ENSG00000048649.9	ENSG00000050327.10	ENSG00000075131.5	ENSG00000079393.16
	ENSG00000085760.10	ENSG00000091262.10	ENSG00000092036.12	ENSG00000100603.9
	ENSG00000101342.5	ENSG00000101639.14	ENSG00000105668.3	ENSG00000105855.5
**Brain Hippocampus**	ENSG00000107099.11	ENSG00000108272.9	ENSG00000108448.16	ENSG00000111364.11
	ENSG00000111581.5	ENSG00000111912.14	ENSG00000112394.12	ENSG00000117899.6
	ENSG00000122376.7	ENSG00000123684.8	ENSG00000124275.10	ENSG00000132763.10
	ENSG00000133983.10	ENSG00000137672.8	ENSG00000140326.8	ENSG00000142065.9
	ENSG00000143194.8	ENSG00000143801.12	ENSG00000143819.8	ENSG00000146112.7
	ENSG00000153291.11	ENSG00000157693.10	ENSG00000157911.5	ENSG00000159915.8
	ENSG00000160949.12	ENSG00000161217.7	ENSG00000162994.11	ENSG00000163394.5
	ENSG00000163794.6	ENSG00000164692.13	ENSG00000164880.11	ENSG00000165650.7
	ENSG00000166173.9	ENSG00000166938.8	ENSG00000167670.11	ENSG00000167842.11
	ENSG00000168216.6	ENSG00000170275.10	ENSG00000171130.13	ENSG00000171928.9

	ENSG00000172053.10	ENSG00000174194.11	ENSG00000174226.4	ENSG00000174652.13
	ENSG00000176678.4	ENSG00000176998.3	ENSG00000177990.7	ENSG00000179061.1
	ENSG00000180113.11	ENSG00000180185.7	ENSG00000180481.6	ENSG00000180884.9
	ENSG00000180953.7	ENSG00000182700.3	ENSG00000185112.4	ENSG00000185523.6
	ENSG00000186470.9	ENSG00000197372.5	ENSG00000197451.6	ENSG00000198089.10
	ENSG00000198920.5	ENSG00000204381.7	ENSG00000204406.7	ENSG00000204520.8
	ENSG00000204622.6	ENSG00000205045.4	ENSG00000205822.6	ENSG00000215861.4
	ENSG00000216775.2	ENSG00000223361.5	ENSG00000224924.2	ENSG00000225171.2
	ENSG00000225241.3	ENSG00000225398.2	ENSG00000225916.1	ENSG00000226745.2
	ENSG00000230795.2	ENSG00000232063.1	ENSG00000235098.4	ENSG00000235736.1
	ENSG00000236537.1	ENSG00000236624.4	ENSG00000242247.6	ENSG00000244414.2
	ENSG00000245937.3	ENSG00000248184.1	ENSG00000259905.1	ENSG00000260077.1
	ENSG00000261512.2	ENSG00000261556.4	ENSG00000261770.1	ENSG00000267079.1
	ENSG00000267554.1	ENSG00000267575.2	ENSG00000267623.2	ENSG00000270154.1
	ENSG00000271821.1			
**Brain Hypothalamus**	ENSG00000037042.8	ENSG00000038002.4	ENSG00000051620.6	ENSG00000054219.9
	ENSG00000083099.6	ENSG00000090097.16	ENSG00000092036.12	ENSG00000099800.3
	ENSG00000100612.9	ENSG00000101098.8	ENSG00000101255.6	ENSG00000101489.14
	ENSG00000101639.14	ENSG00000103168.12	ENSG00000104728.11	ENSG00000105672.10
	ENSG00000105866.9	ENSG00000106829.14	ENSG00000106853.12	ENSG00000108272.9
	ENSG00000111364.11	ENSG00000112799.4	ENSG00000114107.4	ENSG00000117226.7
	ENSG00000122376.7	ENSG00000122696.8	ENSG00000126602.6	ENSG00000127054.14
	ENSG00000127364.2	ENSG00000128274.11	ENSG00000131653.8	ENSG00000132664.7
	ENSG00000136235.11	ENSG00000136875.8	ENSG00000138029.9	ENSG00000138316.6
	ENSG00000138400.8	ENSG00000141127.10	ENSG00000144026.7	ENSG00000144029.7
	ENSG00000148123.10	ENSG00000148296.5	ENSG00000148572.10	ENSG00000148935.6
	ENSG00000149488.11	ENSG00000156414.14	ENSG00000156931.11	ENSG00000157578.9
	ENSG00000157734.9	ENSG00000159399.5	ENSG00000162777.12	ENSG00000164880.11
	ENSG00000165272.10	ENSG00000165650.7	ENSG00000166398.8	ENSG00000166436.11
	ENSG00000168439.12	ENSG00000168944.11	ENSG00000169609.9	ENSG00000170275.10
	ENSG00000170906.11	ENSG00000171130.13	ENSG00000173226.12	ENSG00000173599.9
	ENSG00000173947.9	ENSG00000173992.4	ENSG00000174194.11	ENSG00000174226.4
	ENSG00000174652.13	ENSG00000175764.10	ENSG00000176386.4	ENSG00000176396.9
	ENSG00000178021.9	ENSG00000179242.11	ENSG00000180113.11	ENSG00000180185.7
	ENSG00000180198.11	ENSG00000180481.6	ENSG00000183571.9	ENSG00000184261.4
	ENSG00000185088.8	ENSG00000185189.11	ENSG00000185955.4	ENSG00000185963.9
	ENSG00000186470.9	ENSG00000187135.7	ENSG00000196502.7	ENSG00000197165.6
	ENSG00000197646.6	ENSG00000198721.8	ENSG00000204160.7	ENSG00000204463.8
	ENSG00000204520.8	ENSG00000205822.6	ENSG00000213171.2	ENSG00000213293.4
	ENSG00000215861.4	ENSG00000223345.3	ENSG00000223496.1	ENSG00000223572.5
	ENSG00000224265.1	ENSG00000225880.4	ENSG00000227742.1	ENSG00000228623.2
	ENSG00000231709.1	ENSG00000235007.2	ENSG00000235098.4	ENSG00000237560.1
	ENSG00000238286.1	ENSG00000240089.2	ENSG00000243517.1	ENSG00000243709.1
	ENSG00000246465.1	ENSG00000248712.3	ENSG00000261556.4	ENSG00000261770.1
	ENSG00000261889.1	ENSG00000263627.1	ENSG00000266145.1	ENSG00000267053.2
	ENSG00000267264.1	ENSG00000267623.2	ENSG00000268458.1	ENSG00000269399.1
	ENSG00000270081.1	ENSG00000270672.1	ENSG00000271040.1	ENSG00000271387.1
	ENSG00000272216.1	ENSG00000272462.2	ENSG00000272667.1	
**Brain Nucleus**	ENSG00000007908.11	ENSG00000010704.14	ENSG00000038002.4	ENSG00000054219.9

	ENSG00000088836.8	ENSG00000092036.12	ENSG00000099800.3	ENSG00000101104.8
	ENSG00000101255.6	ENSG00000101574.10	ENSG00000101639.14	ENSG00000103168.12
	ENSG00000103351.8	ENSG00000105793.11	ENSG00000106823.8	ENSG00000108272.9
	ENSG00000108278.7	ENSG00000110448.6	ENSG00000112144.11	ENSG00000112763.11
	ENSG00000113318.9	ENSG00000115255.6	ENSG00000117226.7	ENSG00000117899.6
	ENSG00000119335.12	ENSG00000120318.11	ENSG00000120910.10	ENSG00000122203.10
	ENSG00000122376.7	ENSG00000122550.13	ENSG00000124275.10	ENSG00000125885.9
	ENSG00000127054.14	ENSG00000127528.5	ENSG00000128563.9	ENSG00000129467.9
	ENSG00000130305.12	ENSG00000130695.9	ENSG00000132321.12	ENSG00000132749.6
	ENSG00000133983.10	ENSG00000134917.9	ENSG00000136897.6	ENSG00000138029.9
	ENSG00000139343.6	ENSG00000139433.5	ENSG00000139624.8	ENSG00000139973.11
	ENSG00000141255.8	ENSG00000141664.5	ENSG00000143194.8	ENSG00000143951.11
	ENSG00000144026.7	ENSG00000144893.8	ENSG00000145945.5	ENSG00000147649.5
	ENSG00000147955.12	ENSG00000151388.6	ENSG00000151575.10	ENSG00000153291.11
	ENSG00000155324.5	ENSG00000156374.10	ENSG00000157259.6	ENSG00000157578.9
	ENSG00000158109.10	ENSG00000158747.9	ENSG00000162039.10	ENSG00000162493.12
	ENSG00000162753.10	ENSG00000162909.13	ENSG00000163959.5	ENSG00000164736.5
	ENSG00000164880.11	ENSG00000167384.6	ENSG00000170276.4	ENSG00000171130.13
	ENSG00000171522.5	ENSG00000172818.5	ENSG00000173226.12	ENSG00000174514.8
	ENSG00000174652.13	ENSG00000175198.10	ENSG00000175395.11	ENSG00000176386.4
	ENSG00000176659.5	ENSG00000177963.8	ENSG00000178202.8	ENSG00000179057.9
	ENSG00000180185.7	ENSG00000180481.6	ENSG00000180535.3	ENSG00000180953.7
	ENSG00000181322.9	ENSG00000182704.6	ENSG00000183423.7	ENSG00000185186.4
	ENSG00000185344.9	ENSG00000185730.3	ENSG00000185963.9	ENSG00000186470.9
	ENSG00000188388.9	ENSG00000188629.7	ENSG00000188659.5	ENSG00000196193.4
	ENSG00000196366.1	ENSG00000196418.8	ENSG00000198156.6	ENSG00000198792.8
	ENSG00000198829.5	ENSG00000204520.8	ENSG00000204540.6	ENSG00000205822.6
	ENSG00000213760.6	ENSG00000214141.4	ENSG00000215114.3	ENSG00000215712.6
	ENSG00000215861.4	ENSG00000223865.6	ENSG00000225241.3	ENSG00000225398.2
	ENSG00000228521.2	ENSG00000231381.2	ENSG00000231861.1	ENSG00000233319.1
	ENSG00000235012.1	ENSG00000235098.4	ENSG00000235241.1	ENSG00000236297.1
	ENSG00000236624.4	ENSG00000236780.1	ENSG00000240038.2	ENSG00000241043.1
	ENSG00000242485.1	ENSG00000243244.1	ENSG00000248489.1	ENSG00000250312.2
	ENSG00000251293.1	ENSG00000251655.2	ENSG00000253309.2	ENSG00000255513.1
	ENSG00000256053.3	ENSG00000258072.1	ENSG00000259539.1	ENSG00000260396.1
	ENSG00000260725.1	ENSG00000261556.4	ENSG00000261770.1	ENSG00000263142.1
	ENSG00000264880.1	ENSG00000267340.1	ENSG00000269430.1	ENSG00000271743.1
	ENSG00000271788.1	ENSG00000271889.1	ENSG00000272942.1	
**Brain Putamen**	ENSG00000001617.7	ENSG00000004777.14	ENSG00000052749.9	ENSG00000055813.5
	ENSG00000060491.12	ENSG00000065060.12	ENSG00000073578.12	ENSG00000080644.11
	ENSG00000083099.6	ENSG00000085719.7	ENSG00000088836.8	ENSG00000091262.10
	ENSG00000096093.10	ENSG00000099800.3	ENSG00000100288.15	ENSG00000100461.13
	ENSG00000100644.12	ENSG00000101104.8	ENSG00000101204.11	ENSG00000101255.6
	ENSG00000104818.14	ENSG00000105732.9	ENSG00000106018.9	ENSG00000108272.9
	ENSG00000110066.10	ENSG00000110906.8	ENSG00000113119.8	ENSG00000117477.8
	ENSG00000119927.9	ENSG00000122729.14	ENSG00000123685.4	ENSG00000124275.10
	ENSG00000124562.5	ENSG00000127995.12	ENSG00000128218.7	ENSG00000128524.4
	ENSG00000130193.7	ENSG00000134186.7	ENSG00000135439.7	ENSG00000136235.11
	ENSG00000136783.9	ENSG00000137269.10	ENSG00000138376.6	ENSG00000139168.3

	ENSG00000140905.5	ENSG00000141127.10	ENSG00000142065.9	ENSG00000142694.6
	ENSG00000142765.13	ENSG00000142856.12	ENSG00000144559.6	ENSG00000147419.12
	ENSG00000149311.13	ENSG00000150361.7	ENSG00000151388.6	ENSG00000151611.9
	ENSG00000154768.4	ENSG00000157578.9	ENSG00000158747.9	ENSG00000159352.11
	ENSG00000159904.7	ENSG00000162994.11	ENSG00000163629.8	ENSG00000164105.3
	ENSG00000164880.11	ENSG00000165646.7	ENSG00000165650.7	ENSG00000165724.5
	ENSG00000166451.9	ENSG00000166664.9	ENSG00000166780.6	ENSG00000167094.11
	ENSG00000167183.2	ENSG00000167654.13	ENSG00000169515.5	ENSG00000169609.9
	ENSG00000169750.4	ENSG00000171130.13	ENSG00000172780.12	ENSG00000174194.11
	ENSG00000174652.13	ENSG00000174839.8	ENSG00000176155.14	ENSG00000176396.9
	ENSG00000177963.8	ENSG00000178802.13	ENSG00000180113.11	ENSG00000180185.7
	ENSG00000180481.6	ENSG00000183067.5	ENSG00000183454.9	ENSG00000184209.14
	ENSG00000184445.7	ENSG00000185088.8	ENSG00000185684.8	ENSG00000186470.9
	ENSG00000188388.9	ENSG00000188610.8	ENSG00000188659.5	ENSG00000196071.3
	ENSG00000196233.7	ENSG00000197291.4	ENSG00000198931.6	ENSG00000204381.7
	ENSG00000204444.6	ENSG00000204520.8	ENSG00000205822.6	ENSG00000205930.4
	ENSG00000213398.3	ENSG00000214439.3	ENSG00000215861.4	ENSG00000217442.3
	ENSG00000223496.1	ENSG00000224683.1	ENSG00000228979.3	ENSG00000229360.1
	ENSG00000230387.1	ENSG00000230510.2	ENSG00000231841.1	ENSG00000232629.4
	ENSG00000232694.2	ENSG00000233200.1	ENSG00000233961.1	ENSG00000236624.4
	ENSG00000237510.3	ENSG00000240875.1	ENSG00000242247.6	ENSG00000244731.3
	ENSG00000248641.1	ENSG00000249797.1	ENSG00000250786.1	ENSG00000250878.3
	ENSG00000251575.2	ENSG00000251655.2	ENSG00000257464.1	ENSG00000260077.1
	ENSG00000260776.1	ENSG00000261011.1	ENSG00000261556.4	ENSG00000261770.1
	ENSG00000263503.1	ENSG00000266490.1	ENSG00000266964.1	ENSG00000267623.2
	ENSG00000270604.1	ENSG00000271361.1	ENSG00000272462.2	ENSG00000273230.1
	ENSG00000273287.1	ENSG00000273293.1		
**Brain Spinal cord**	ENSG00000012223.8	ENSG00000013288.4	ENSG00000021488.8	ENSG00000063854.8
	ENSG00000064102.10	ENSG00000080189.10	ENSG00000086288.7	ENSG00000089050.10
	ENSG00000092036.12	ENSG00000093183.9	ENSG00000095970.12	ENSG00000100714.11
	ENSG00000100938.13	ENSG00000101639.14	ENSG00000103150.4	ENSG00000104783.7
	ENSG00000104980.3	ENSG00000110274.10	ENSG00000111725.6	ENSG00000111912.14
	ENSG00000112763.11	ENSG00000112796.5	ENSG00000117226.7	ENSG00000117519.11
	ENSG00000124120.6	ENSG00000126246.5	ENSG00000130592.9	ENSG00000130958.7
	ENSG00000131069.15	ENSG00000132196.9	ENSG00000132330.12	ENSG00000132763.10
	ENSG00000133606.6	ENSG00000134375.6	ENSG00000137992.10	ENSG00000138029.9
	ENSG00000138606.15	ENSG00000138760.4	ENSG00000139197.6	ENSG00000141295.9
	ENSG00000142173.10	ENSG00000143321.14	ENSG00000143970.12	ENSG00000144026.7
	ENSG00000150403.13	ENSG00000152348.11	ENSG00000153291.11	ENSG00000154162.9
	ENSG00000156875.9	ENSG00000158470.5	ENSG00000160050.10	ENSG00000160226.11
	ENSG00000161326.8	ENSG00000161654.5	ENSG00000162878.8	ENSG00000163430.5
	ENSG00000163576.13	ENSG00000165650.7	ENSG00000166839.12	ENSG00000167377.13
	ENSG00000167670.11	ENSG00000167964.8	ENSG00000169228.9	ENSG00000169429.6
	ENSG00000169609.9	ENSG00000169764.10	ENSG00000170325.10	ENSG00000170906.11
	ENSG00000173406.11	ENSG00000175073.7	ENSG00000175416.8	ENSG00000176386.4
	ENSG00000176399.3	ENSG00000176998.3	ENSG00000177106.10	ENSG00000177674.11
	ENSG00000177707.6	ENSG00000177963.8	ENSG00000179988.9	ENSG00000180185.7
	ENSG00000180481.6	ENSG00000182919.10	ENSG00000183748.4	ENSG00000184471.6
	ENSG00000185823.2	ENSG00000185933.6	ENSG00000186470.9	ENSG00000187792.3

	ENSG00000188659.5	ENSG00000188786.9	ENSG00000196081.5	ENSG00000196502.7
	ENSG00000196628.9	ENSG00000198150.2	ENSG00000198246.7	ENSG00000204525.10
	ENSG00000205871.4	ENSG00000215861.4	ENSG00000223496.1	ENSG00000225241.3
	ENSG00000227666.1	ENSG00000232677.2	ENSG00000233200.1	ENSG00000236289.2
	ENSG00000236297.1	ENSG00000236809.2	ENSG00000237541.3	ENSG00000241316.2
	ENSG00000246596.2	ENSG00000250215.1	ENSG00000250571.2	ENSG00000251575.2
	ENSG00000254595.1	ENSG00000254667.1	ENSG00000259556.2	ENSG00000259592.1
	ENSG00000259630.2	ENSG00000261556.4	ENSG00000261770.1	ENSG00000267107.2
	ENSG00000267623.2	ENSG00000269783.1	ENSG00000273230.1	ENSG00000273356.1
**Brain Substantia nigra**	ENSG00000010438.12	ENSG00000065060.12	ENSG00000068781.16	ENSG00000072952.14
	ENSG00000092036.12	ENSG00000092531.5	ENSG00000099817.7	ENSG00000100664.6
	ENSG00000101639.14	ENSG00000103051.14	ENSG00000103351.8	ENSG00000105889.10
	ENSG00000106034.13	ENSG00000107566.9	ENSG00000108551.4	ENSG00000109956.8
	ENSG00000111817.12	ENSG00000111912.14	ENSG00000114353.12	ENSG00000123965.12
	ENSG00000124160.7	ENSG00000125409.8	ENSG00000134184.8	ENSG00000134853.7
	ENSG00000135747.7	ENSG00000135916.11	ENSG00000137434.7	ENSG00000139624.8
	ENSG00000140400.10	ENSG00000140905.5	ENSG00000141431.5	ENSG00000143740.10
	ENSG00000143933.12	ENSG00000146556.10	ENSG00000147996.12	ENSG00000152942.14
	ENSG00000157322.12	ENSG00000159596.6	ENSG00000160062.10	ENSG00000161558.6
	ENSG00000163964.9	ENSG00000164330.12	ENSG00000165644.6	ENSG00000166140.13
	ENSG00000167670.11	ENSG00000169218.9	ENSG00000170866.7	ENSG00000171130.13
	ENSG00000172456.12	ENSG00000174652.13	ENSG00000176105.9	ENSG00000176998.3
	ENSG00000177951.13	ENSG00000178741.7	ENSG00000180113.11	ENSG00000180245.4
	ENSG00000180481.6	ENSG00000181915.3	ENSG00000183844.12	ENSG00000186198.3
	ENSG00000186470.9	ENSG00000187068.2	ENSG00000188659.5	ENSG00000196735.7
	ENSG00000197362.9	ENSG00000204130.8	ENSG00000204138.8	ENSG00000204520.8
	ENSG00000206527.5	ENSG00000215154.2	ENSG00000215861.4	ENSG00000221953.2
	ENSG00000228789.2	ENSG00000230156.2	ENSG00000235098.4	ENSG00000242247.6
	ENSG00000249908.1	ENSG00000250564.1	ENSG00000254999.2	ENSG00000258599.2
	ENSG00000259495.1	ENSG00000259905.1	ENSG00000261770.1	ENSG00000262165.1
	ENSG00000267623.2	ENSG00000271788.1	ENSG00000272783.1	ENSG00000273281.1
**Cells EBV**	ENSG00000005100.8	ENSG00000005955.8	ENSG00000009724.12	ENSG00000013288.4
	ENSG00000013523.5	ENSG00000026950.12	ENSG00000048028.7	ENSG00000051620.6
	ENSG00000055483.15	ENSG00000068796.12	ENSG00000076555.11	ENSG00000086232.8
	ENSG00000089737.11	ENSG00000089775.7	ENSG00000100300.13	ENSG00000102710.15
	ENSG00000103202.8	ENSG00000103415.7	ENSG00000105258.4	ENSG00000107560.6
	ENSG00000107625.8	ENSG00000107679.10	ENSG00000108272.9	ENSG00000108278.7
	ENSG00000108559.7	ENSG00000110092.3	ENSG00000111361.8	ENSG00000111845.4
	ENSG00000116957.8	ENSG00000117010.11	ENSG00000118873.11	ENSG00000119326.10
	ENSG00000120539.10	ENSG00000124275.10	ENSG00000124588.15	ENSG00000126860.7
	ENSG00000127054.14	ENSG00000127249.10	ENSG00000127366.4	ENSG00000129204.12
	ENSG00000130940.10	ENSG00000131475.2	ENSG00000132481.2	ENSG00000132849.14
	ENSG00000133065.6	ENSG00000133731.5	ENSG00000133812.10	ENSG00000136371.5
	ENSG00000136875.8	ENSG00000137198.5	ENSG00000137404.10	ENSG00000137996.8
	ENSG00000138346.10	ENSG00000138801.4	ENSG00000140400.10	ENSG00000140905.5
	ENSG00000141424.8	ENSG00000142920.12	ENSG00000144021.2	ENSG00000144401.10
	ENSG00000144642.16	ENSG00000144792.5	ENSG00000146457.10	ENSG00000147419.12
	ENSG00000150627.11	ENSG00000151690.10	ENSG00000152439.8	ENSG00000153558.9
	ENSG00000153786.8	ENSG00000154330.8	ENSG00000157999.5	ENSG00000158122.7

	ENSG00000159882.8	ENSG00000159958.3	ENSG00000160226.11	ENSG00000160392.9
	ENSG00000161265.10	ENSG00000161929.10	ENSG00000162627.12	ENSG00000163050.12
	ENSG00000163249.5	ENSG00000163788.9	ENSG00000164880.11	ENSG00000165392.5
	ENSG00000165646.7	ENSG00000165650.7	ENSG00000166268.6	ENSG00000167202.7
	ENSG00000167842.11	ENSG00000169372.8	ENSG00000169609.9	ENSG00000169967.12
	ENSG00000170298.11	ENSG00000171234.9	ENSG00000173077.10	ENSG00000173915.8
	ENSG00000174038.8	ENSG00000174123.6	ENSG00000174125.3	ENSG00000174130.8
	ENSG00000174194.11	ENSG00000174652.13	ENSG00000177302.10	ENSG00000177674.11
	ENSG00000178386.8	ENSG00000179988.9	ENSG00000180185.7	ENSG00000180481.6
	ENSG00000180539.4	ENSG00000181074.3	ENSG00000181450.13	ENSG00000181773.6
	ENSG00000182782.7	ENSG00000186470.9	ENSG00000196345.8	ENSG00000196371.2
	ENSG00000196458.6	ENSG00000196743.4	ENSG00000197375.8	ENSG00000198467.9
	ENSG00000198885.5	ENSG00000198945.3	ENSG00000203546.3	ENSG00000204959.3
	ENSG00000205302.2	ENSG00000205583.9	ENSG00000206077.6	ENSG00000206527.5
	ENSG00000214290.3	ENSG00000215784.4	ENSG00000216775.2	ENSG00000225784.5
	ENSG00000226491.1	ENSG00000228499.1	ENSG00000228716.2	ENSG00000236297.1
	ENSG00000236624.4	ENSG00000236946.2	ENSG00000240007.1	ENSG00000240875.1
	ENSG00000243566.2	ENSG00000248835.2	ENSG00000256673.1	ENSG00000257093.2
	ENSG00000257594.2	ENSG00000259959.1	ENSG00000259982.1	ENSG00000260655.1
	ENSG00000261455.1	ENSG00000261556.4	ENSG00000269165.1	ENSG00000271550.1
	ENSG00000271581.1	ENSG00000272325.1		
**Cells Transformed**	ENSG00000002549.8	ENSG00000005955.8	ENSG00000013288.4	ENSG00000018510.8
	ENSG00000035115.17	ENSG00000051620.6	ENSG00000054219.9	ENSG00000058056.4
	ENSG00000059758.3	ENSG00000063854.8	ENSG00000070770.4	ENSG00000075131.5
	ENSG00000075239.9	ENSG00000075336.7	ENSG00000075399.8	ENSG00000078124.7
	ENSG00000079785.10	ENSG00000079950.9	ENSG00000083099.6	ENSG00000084090.9
	ENSG00000085415.11	ENSG00000085982.9	ENSG00000087206.12	ENSG00000088836.8
	ENSG00000090861.11	ENSG00000091536.12	ENSG00000092036.12	ENSG00000092094.6
	ENSG00000095383.15	ENSG00000095564.9	ENSG00000096093.10	ENSG00000099139.9
	ENSG00000099956.13	ENSG00000100364.14	ENSG00000100478.10	ENSG00000101224.13
	ENSG00000101460.8	ENSG00000101608.8	ENSG00000101639.14	ENSG00000103042.4
	ENSG00000103168.12	ENSG00000103472.5	ENSG00000103599.15	ENSG00000104435.9
	ENSG00000104852.10	ENSG00000105341.14	ENSG00000105849.5	ENSG00000105963.9
	ENSG00000106327.8	ENSG00000107854.5	ENSG00000108278.7	ENSG00000108352.7
	ENSG00000108559.7	ENSG00000108604.11	ENSG00000109680.6	ENSG00000110076.14
	ENSG00000111144.5	ENSG00000111237.14	ENSG00000111331.8	ENSG00000111364.11
	ENSG00000111530.8	ENSG00000111664.6	ENSG00000111816.6	ENSG00000111913.11
	ENSG00000112144.11	ENSG00000112667.8	ENSG00000112679.10	ENSG00000113318.9
	ENSG00000113721.9	ENSG00000115137.7	ENSG00000115355.11	ENSG00000115507.5
	ENSG00000115935.12	ENSG00000116678.14	ENSG00000117226.7	ENSG00000117834.8
	ENSG00000119927.9	ENSG00000120008.11	ENSG00000120156.16	ENSG00000120314.14
	ENSG00000120333.4	ENSG00000120337.7	ENSG00000120539.10	ENSG00000120696.8
	ENSG00000121848.9	ENSG00000123219.8	ENSG00000125633.6	ENSG00000125637.11
	ENSG00000126861.4	ENSG00000128408.7	ENSG00000129003.11	ENSG00000129197.10
	ENSG00000129467.9	ENSG00000131015.4	ENSG00000132388.8	ENSG00000133703.7
	ENSG00000133731.5	ENSG00000133983.10	ENSG00000134905.12	ENSG00000135040.11
	ENSG00000135341.13	ENSG00000135905.14	ENSG00000136243.12	ENSG00000136877.10
	ENSG00000137103.12	ENSG00000137221.10	ENSG00000137273.3	ENSG00000137413.11
	ENSG00000138119.12	ENSG00000138172.6	ENSG00000138207.8	ENSG00000138297.9

ENSG00000138347.11	ENSG00000138400.8	ENSG00000138735.11	ENSG00000139116.13
ENSG00000139531.8	ENSG00000139629.11	ENSG00000140262.13	ENSG00000140416.15
ENSG00000140937.9	ENSG00000140950.11	ENSG00000141140.12	ENSG00000141404.11
ENSG00000141452.5	ENSG00000142599.13	ENSG00000142611.12	ENSG00000142856.12
ENSG00000143799.8	ENSG00000143862.3	ENSG00000144021.2	ENSG00000144410.4
ENSG00000145506.9	ENSG00000145623.8	ENSG00000145949.8	ENSG00000147488.7
ENSG00000148153.9	ENSG00000149054.10	ENSG00000151327.8	ENSG00000151348.9
ENSG00000151692.10	ENSG00000152061.17	ENSG00000153066.8	ENSG00000153246.7
ENSG00000153786.8	ENSG00000156304.10	ENSG00000157259.6	ENSG00000158220.9
ENSG00000158555.10	ENSG00000159433.7	ENSG00000159596.6	ENSG00000160007.13
ENSG00000160408.10	ENSG00000160972.5	ENSG00000161204.7	ENSG00000162039.10
ENSG00000162551.9	ENSG00000162643.8	ENSG00000162869.11	ENSG00000162994.11
ENSG00000163092.15	ENSG00000163328.9	ENSG00000163686.9	ENSG00000163959.5
ENSG00000164136.12	ENSG00000164713.5	ENSG00000165406.11	ENSG00000165646.7
ENSG00000165650.7	ENSG00000166016.4	ENSG00000166333.9	ENSG00000166342.14
ENSG00000166396.8	ENSG00000167333.8	ENSG00000167797.3	ENSG00000167815.7
ENSG00000167842.11	ENSG00000168228.10	ENSG00000168374.6	ENSG00000168394.9
ENSG00000168502.13	ENSG00000168899.4	ENSG00000169605.5	ENSG00000169609.9
ENSG00000170745.7	ENSG00000171621.9	ENSG00000171649.7	ENSG00000171729.9
ENSG00000172264.12	ENSG00000172296.8	ENSG00000172318.4	ENSG00000172889.11
ENSG00000173226.12	ENSG00000173267.9	ENSG00000173918.10	ENSG00000174130.8
ENSG00000174165.3	ENSG00000174194.11	ENSG00000174370.5	ENSG00000174437.12
ENSG00000174574.11	ENSG00000174652.13	ENSG00000174705.7	ENSG00000175356.8
ENSG00000175564.8	ENSG00000175832.8	ENSG00000175899.10	ENSG00000176155.14
ENSG00000176531.6	ENSG00000176998.3	ENSG00000177302.10	ENSG00000177427.8
ENSG00000177963.8	ENSG00000178297.8	ENSG00000178381.7	ENSG00000180185.7
ENSG00000180481.6	ENSG00000180771.10	ENSG00000180773.10	ENSG00000180953.7
ENSG00000181619.11	ENSG00000182771.13	ENSG00000182899.10	ENSG00000183579.11
ENSG00000183762.8	ENSG00000183765.16	ENSG00000184178.11	ENSG00000184389.8
ENSG00000184428.8	ENSG00000184900.11	ENSG00000184986.6	ENSG00000185298.8
ENSG00000186020.8	ENSG00000186026.6	ENSG00000187944.2	ENSG00000188610.8
ENSG00000188659.5	ENSG00000188735.8	ENSG00000189050.10	ENSG00000189134.3
ENSG00000189280.3	ENSG00000189433.5	ENSG00000196345.8	ENSG00000196526.6
ENSG00000196843.11	ENSG00000197146.2	ENSG00000197498.8	ENSG00000197566.5
ENSG00000197646.6	ENSG00000197747.4	ENSG00000197885.6	ENSG00000198576.2
ENSG00000198589.6	ENSG00000203724.6	ENSG00000203843.3	ENSG00000204209.6
ENSG00000204520.8	ENSG00000204556.4	ENSG00000204920.6	ENSG00000204959.3
ENSG00000205307.6	ENSG00000205549.4	ENSG00000206043.6	ENSG00000213244.3
ENSG00000214290.3	ENSG00000214944.5	ENSG00000221990.2	ENSG00000223496.1
ENSG00000225851.1	ENSG00000226328.2	ENSG00000226598.1	ENSG00000227345.4
ENSG00000229771.1	ENSG00000230415.1	ENSG00000230638.3	ENSG00000230869.1
ENSG00000231925.7	ENSG00000232063.1	ENSG00000232224.1	ENSG00000232527.3
ENSG00000232677.2	ENSG00000235098.4	ENSG00000235413.3	ENSG00000236039.1
ENSG00000236624.4	ENSG00000237176.3	ENSG00000237510.3	ENSG00000240038.2
ENSG00000240583.6	ENSG00000241494.1	ENSG00000242173.4	ENSG00000242992.2
ENSG00000244041.3	ENSG00000244414.2	ENSG00000244731.3	ENSG00000245937.3
ENSG00000248161.1	ENSG00000248487.4	ENSG00000249476.1	ENSG00000250786.1
ENSG00000250790.3	ENSG00000251521.2	ENSG00000251580.1	ENSG00000253368.3
ENSG00000253853.1	ENSG00000254285.2	ENSG00000254319.1	ENSG00000254772.5

	ENSG00000254986.3	ENSG00000256223.1	ENSG00000257093.2	ENSG00000258818.2
	ENSG00000258952.1	ENSG00000259577.1	ENSG00000260274.1	ENSG00000260452.1
	ENSG00000260971.3	ENSG00000261455.1	ENSG00000261556.4	ENSG00000262097.1
	ENSG00000262454.1	ENSG00000265787.1	ENSG00000265916.1	ENSG00000266490.1
	ENSG00000267053.2	ENSG00000267575.2	ENSG00000267669.1	ENSG00000267939.1
	ENSG00000268746.1	ENSG00000270775.1	ENSG00000271550.1	ENSG00000272005.1
	ENSG00000272459.1	ENSG00000272462.2	ENSG00000272573.1	ENSG00000272810.1
	ENSG00000272828.1	ENSG00000273230.1	ENSG00000273243.1	ENSG00000273287.1
**Colon Sigmoid**	ENSG00000005486.12	ENSG00000005981.8	ENSG00000008324.6	ENSG00000011332.15
	ENSG00000013288.4	ENSG00000030110.8	ENSG00000047579.15	ENSG00000050327.10
	ENSG00000054219.9	ENSG00000069020.14	ENSG00000082641.11	ENSG00000091592.11
	ENSG00000092036.12	ENSG00000099810.14	ENSG00000099956.13	ENSG00000100280.12
	ENSG00000100461.13	ENSG00000100577.14	ENSG00000101255.6	ENSG00000101460.8
	ENSG00000101639.14	ENSG00000102900.8	ENSG00000103351.8	ENSG00000104783.7
	ENSG00000105204.9	ENSG00000105281.8	ENSG00000105612.4	ENSG00000105856.9
	ENSG00000107159.8	ENSG00000107679.10	ENSG00000107854.5	ENSG00000108187.11
	ENSG00000108272.9	ENSG00000108785.7	ENSG00000108786.6	ENSG00000110455.9
	ENSG00000111581.5	ENSG00000111962.7	ENSG00000112276.9	ENSG00000113318.9
	ENSG00000113356.6	ENSG00000116039.7	ENSG00000116199.7	ENSG00000116663.6
	ENSG00000116785.9	ENSG00000117226.7	ENSG00000119227.3	ENSG00000120334.11
	ENSG00000120451.6	ENSG00000122376.7	ENSG00000122870.7	ENSG00000123191.9
	ENSG00000124207.12	ENSG00000124275.10	ENSG00000124508.12	ENSG00000125885.9
	ENSG00000128609.10	ENSG00000129270.11	ENSG00000130540.9	ENSG00000130653.11
	ENSG00000130684.9	ENSG00000132972.14	ENSG00000133059.12	ENSG00000133731.5
	ENSG00000133983.10	ENSG00000134905.12	ENSG00000135451.8	ENSG00000135709.8
	ENSG00000136247.10	ENSG00000136315.3	ENSG00000136856.13	ENSG00000137312.10
	ENSG00000137414.5	ENSG00000137996.8	ENSG00000138119.12	ENSG00000138152.7
	ENSG00000138172.6	ENSG00000138400.8	ENSG00000138777.15	ENSG00000139343.6
	ENSG00000139722.2	ENSG00000140262.13	ENSG00000141140.12	ENSG00000141576.10
	ENSG00000142082.10	ENSG00000143374.10	ENSG00000143924.14	ENSG00000143951.11
	ENSG00000144021.2	ENSG00000144134.14	ENSG00000145331.9	ENSG00000146540.10
	ENSG00000146904.4	ENSG00000149294.12	ENSG00000149596.6	ENSG00000149634.4
	ENSG00000152253.4	ENSG00000152465.13	ENSG00000153246.7	ENSG00000153291.11
	ENSG00000153446.11	ENSG00000155816.15	ENSG00000156521.9	ENSG00000157259.6
	ENSG00000157322.12	ENSG00000157379.9	ENSG00000157837.11	ENSG00000158109.10
	ENSG00000163071.6	ENSG00000163682.11	ENSG00000163754.13	ENSG00000164081.8
	ENSG00000164338.5	ENSG00000164849.7	ENSG00000164855.11	ENSG00000164880.11
	ENSG00000164938.9	ENSG00000164941.9	ENSG00000165650.7	ENSG00000166471.6
	ENSG00000167377.13	ENSG00000167670.11	ENSG00000167840.9	ENSG00000167842.11
	ENSG00000169075.7	ENSG00000170906.11	ENSG00000171903.12	ENSG00000172379.14
	ENSG00000172938.3	ENSG00000173175.10	ENSG00000173226.12	ENSG00000174125.3
	ENSG00000174194.11	ENSG00000174226.4	ENSG00000174407.7	ENSG00000174652.13
	ENSG00000175166.12	ENSG00000175691.8	ENSG00000176261.11	ENSG00000176386.4
	ENSG00000176998.3	ENSG00000177106.10	ENSG00000177463.11	ENSG00000178248.10
	ENSG00000178381.7	ENSG00000178386.8	ENSG00000178741.7	ENSG00000179242.11
	ENSG00000179978.10	ENSG00000180113.11	ENSG00000180185.7	ENSG00000180481.6
	ENSG00000180881.15	ENSG00000180953.7	ENSG00000181450.13	ENSG00000181915.3
	ENSG00000182541.13	ENSG00000182771.13	ENSG00000183571.9	ENSG00000183617.4
	ENSG00000185344.9	ENSG00000185442.8	ENSG00000185946.11	ENSG00000186470.9

	ENSG00000186889.5	ENSG00000187145.10	ENSG00000188000.2	ENSG00000188659.5
	ENSG00000189306.6	ENSG00000197124.7	ENSG00000197146.2	ENSG00000197165.6
	ENSG00000197535.10	ENSG00000197646.6	ENSG00000197747.4	ENSG00000198035.9
	ENSG00000198089.10	ENSG00000198336.5	ENSG00000198648.6	ENSG00000198722.8
	ENSG00000203364.2	ENSG00000204231.6	ENSG00000204438.6	ENSG00000204616.6
	ENSG00000204618.4	ENSG00000204655.7	ENSG00000204866.4	ENSG00000204920.6
	ENSG00000205578.4	ENSG00000205822.6	ENSG00000213240.7	ENSG00000213760.6
	ENSG00000214188.5	ENSG00000214944.5	ENSG00000215861.4	ENSG00000216775.2
	ENSG00000219607.2	ENSG00000223431.1	ENSG00000223496.1	ENSG00000223505.2
	ENSG00000225241.3	ENSG00000225784.5	ENSG00000225851.1	ENSG00000226374.1
	ENSG00000226816.2	ENSG00000227080.2	ENSG00000227262.3	ENSG00000227775.3
	ENSG00000228022.1	ENSG00000228106.1	ENSG00000230911.1	ENSG00000231360.2
	ENSG00000231861.1	ENSG00000231925.7	ENSG00000232677.2	ENSG00000233448.2
	ENSG00000233690.1	ENSG00000233961.1	ENSG00000233967.2	ENSG00000234928.1
	ENSG00000236624.4	ENSG00000237489.2	ENSG00000237510.3	ENSG00000237560.1
	ENSG00000237732.5	ENSG00000238109.1	ENSG00000240356.2	ENSG00000241015.2
	ENSG00000241316.2	ENSG00000242441.3	ENSG00000243414.4	ENSG00000243480.3
	ENSG00000245937.3	ENSG00000246922.4	ENSG00000249771.1	ENSG00000250334.1
	ENSG00000250786.1	ENSG00000251580.1	ENSG00000251669.1	ENSG00000253853.1
	ENSG00000256223.1	ENSG00000256433.1	ENSG00000258647.1	ENSG00000259153.1
	ENSG00000259539.1	ENSG00000260105.2	ENSG00000260179.1	ENSG00000261556.4
	ENSG00000267034.1	ENSG00000267056.2	ENSG00000267058.1	ENSG00000267466.1
	ENSG00000267623.2	ENSG00000269086.2	ENSG00000269976.1	ENSG00000270071.1
	ENSG00000270326.1	ENSG00000271109.1	ENSG00000271581.1	ENSG00000272462.2
	ENSG00000000971.11	ENSG00000005421.4	ENSG00000005955.8	ENSG00000013288.4
	ENSG00000018236.10	ENSG00000024862.12	ENSG00000047579.15	ENSG00000047662.4
	ENSG00000051620.6	ENSG00000058404.15	ENSG00000063180.4	ENSG00000069275.12
	ENSG00000073417.10	ENSG00000075131.5	ENSG00000078725.8	ENSG00000079432.3
**Colon Transverse**	ENSG00000080293.5	ENSG00000083099.6	ENSG00000085982.9	ENSG00000090612.16
	ENSG00000091972.14	ENSG00000092036.12	ENSG00000099956.13	ENSG00000100330.11
	ENSG00000100890.11	ENSG00000101639.14	ENSG00000103599.15	ENSG00000104218.9
	ENSG00000104852.10	ENSG00000106069.16	ENSG00000106105.9	ENSG00000106853.12
	ENSG00000107201.5	ENSG00000107854.5	ENSG00000108264.12	ENSG00000108278.7
	ENSG00000108559.7	ENSG00000108786.6	ENSG00000110700.2	ENSG00000111664.6
	ENSG00000111816.6	ENSG00000113269.9	ENSG00000113318.9	ENSG00000113580.10
	ENSG00000115306.11	ENSG00000115419.8	ENSG00000115514.7	ENSG00000116785.9
	ENSG00000116922.10	ENSG00000116957.8	ENSG00000117226.7	ENSG00000120451.6
	ENSG00000120669.11	ENSG00000122783.12	ENSG00000123191.9	ENSG00000123843.8
	ENSG00000124508.12	ENSG00000125743.6	ENSG00000125885.9	ENSG00000126804.9
	ENSG00000127129.5	ENSG00000128833.8	ENSG00000131620.13	ENSG00000132561.9
	ENSG00000132842.9	ENSG00000132965.5	ENSG00000133731.5	ENSG00000133983.10
	ENSG00000134046.7	ENSG00000134759.9	ENSG00000134905.12	ENSG00000135047.10
	ENSG00000135870.7	ENSG00000135929.4	ENSG00000135956.4	ENSG00000136156.8
	ENSG00000136717.10	ENSG00000137288.5	ENSG00000137502.5	ENSG00000137962.8
	ENSG00000138029.9	ENSG00000138101.14	ENSG00000138119.12	ENSG00000138152.7
	ENSG00000138172.6	ENSG00000138376.6	ENSG00000138400.8	ENSG00000138760.4
	ENSG00000138801.4	ENSG00000140506.12	ENSG00000140623.9	ENSG00000140990.10
	ENSG00000141127.10	ENSG00000141140.12	ENSG00000141314.8	ENSG00000142856.12
	ENSG00000142973.8	ENSG00000143162.7	ENSG00000143412.5	ENSG00000143751.9

	ENSG00000143921.6	ENSG00000144021.2	ENSG00000144026.7	ENSG00000144827.4
	ENSG00000145390.7	ENSG00000146147.10	ENSG00000146247.13	ENSG00000150753.7
	ENSG00000150967.13	ENSG00000152253.4	ENSG00000153291.11	ENSG00000153774.4
	ENSG00000155254.8	ENSG00000155324.5	ENSG00000156103.11	ENSG00000156219.12
	ENSG00000157212.14	ENSG00000157322.12	ENSG00000157837.11	ENSG00000158773.10
	ENSG00000159212.8	ENSG00000160255.12	ENSG00000162040.5	ENSG00000162576.12
	ENSG00000162747.5	ENSG00000162994.11	ENSG00000163071.6	ENSG00000163121.5
	ENSG00000163827.8	ENSG00000164081.8	ENSG00000164465.14	ENSG00000164506.10
	ENSG00000164880.11	ENSG00000165406.11	ENSG00000165646.7	ENSG00000165650.7
	ENSG00000166311.5	ENSG00000166402.4	ENSG00000166471.6	ENSG00000167377.13
	ENSG00000167670.11	ENSG00000167791.7	ENSG00000167800.9	ENSG00000167842.11
	ENSG00000168778.7	ENSG00000169609.9	ENSG00000170175.6	ENSG00000170899.6
	ENSG00000171649.7	ENSG00000171877.15	ENSG00000171943.7	ENSG00000172379.14
	ENSG00000172809.8	ENSG00000173226.12	ENSG00000173264.9	ENSG00000173578.6
	ENSG00000174194.11	ENSG00000174226.4	ENSG00000174652.13	ENSG00000176155.14
	ENSG00000176974.13	ENSG00000176998.3	ENSG00000177191.2	ENSG00000177757.1
	ENSG00000178081.8	ENSG00000178386.8	ENSG00000178741.7	ENSG00000179361.13
	ENSG00000179477.5	ENSG00000179673.3	ENSG00000180104.11	ENSG00000180185.7
	ENSG00000180257.8	ENSG00000180481.6	ENSG00000180881.15	ENSG00000180953.7
	ENSG00000181007.7	ENSG00000181315.6	ENSG00000182568.12	ENSG00000182771.13
	ENSG00000182986.8	ENSG00000184007.13	ENSG00000184389.8	ENSG00000184507.11
	ENSG00000185019.12	ENSG00000185038.10	ENSG00000185298.8	ENSG00000185344.9
	ENSG00000185608.4	ENSG00000186026.6	ENSG00000186283.9	ENSG00000186446.7
	ENSG00000186470.9	ENSG00000188322.4	ENSG00000188388.9	ENSG00000189280.3
	ENSG00000196189.8	ENSG00000196247.7	ENSG00000196458.6	ENSG00000196743.4
	ENSG00000197146.2	ENSG00000197498.8	ENSG00000197646.6	ENSG00000198040.6
	ENSG00000198336.5	ENSG00000198885.5	ENSG00000198945.3	ENSG00000204237.4
	ENSG00000204301.5	ENSG00000204308.6	ENSG00000204560.5	ENSG00000204856.7
	ENSG00000204920.6	ENSG00000204959.3	ENSG00000205312.4	ENSG00000205822.6
	ENSG00000205838.8	ENSG00000213494.5	ENSG00000213626.7	ENSG00000215221.2
	ENSG00000219545.5	ENSG00000223496.1	ENSG00000224389.4	ENSG00000224961.1
	ENSG00000225489.2	ENSG00000227676.2	ENSG00000228789.2	ENSG00000229028.2
	ENSG00000229609.1	ENSG00000230869.1	ENSG00000231360.2	ENSG00000232224.1
	ENSG00000233232.2	ENSG00000235209.1	ENSG00000236624.4	ENSG00000237510.3
	ENSG00000238198.1	ENSG00000239388.4	ENSG00000239670.1	ENSG00000239926.1
	ENSG00000241635.3	ENSG00000242441.3	ENSG00000242611.1	ENSG00000243414.4
	ENSG00000244414.2	ENSG00000248126.1	ENSG00000248489.1	ENSG00000248508.2
	ENSG00000248771.1	ENSG00000250571.2	ENSG00000250786.1	ENSG00000251432.2
	ENSG00000251580.1	ENSG00000253558.1	ENSG00000253853.1	ENSG00000254531.1
	ENSG00000254872.2	ENSG00000255455.2	ENSG00000255983.1	ENSG00000256223.1
	ENSG00000256268.1	ENSG00000256433.1	ENSG00000260103.2	ENSG00000260105.2
	ENSG00000260657.1	ENSG00000261556.4	ENSG00000263818.1	ENSG00000265121.1
	ENSG00000265485.1	ENSG00000267475.1	ENSG00000267623.2	ENSG00000267939.1
	ENSG00000268442.1	ENSG00000269190.1	ENSG00000271109.1	ENSG00000271550.1
	ENSG00000271581.1	ENSG00000272129.1	ENSG00000272455.1	
**Esophagus Gastroesophageal**	ENSG00000001460.13	ENSG00000005955.8	ENSG00000006747.10	ENSG00000007129.13
	ENSG00000013288.4	ENSG00000028839.5	ENSG00000047056.10	ENSG00000054219.9
	ENSG00000055955.11	ENSG00000064115.6	ENSG00000068024.12	ENSG00000083099.6
	ENSG00000083828.11	ENSG00000091262.10	ENSG00000092036.12	ENSG00000092847.6

ENSG00000099949.14	ENSG00000099956.13	ENSG00000100461.13	ENSG00000101190.8
ENSG00000101474.7	ENSG00000101639.14	ENSG00000103460.12	ENSG00000104361.5
ENSG00000104852.10	ENSG00000105819.9	ENSG00000106133.13	ENSG00000107159.8
ENSG00000107854.5	ENSG00000108010.7	ENSG00000108272.9	ENSG00000109452.8
ENSG00000110076.14	ENSG00000111752.6	ENSG00000111816.6	ENSG00000113318.9
ENSG00000115705.16	ENSG00000116106.7	ENSG00000116785.9	ENSG00000117477.8
ENSG00000117834.8	ENSG00000119147.5	ENSG00000120129.5	ENSG00000120451.6
ENSG00000120539.10	ENSG00000121067.13	ENSG00000122375.7	ENSG00000122692.7
ENSG00000122870.7	ENSG00000123374.6	ENSG00000124143.6	ENSG00000124275.10
ENSG00000124535.11	ENSG00000124549.10	ENSG00000124614.9	ENSG00000126246.5
ENSG00000126602.6	ENSG00000127564.12	ENSG00000128218.7	ENSG00000129646.9
ENSG00000130159.9	ENSG00000133731.5	ENSG00000134115.8	ENSG00000135314.8
ENSG00000136315.3	ENSG00000137992.10	ENSG00000137996.8	ENSG00000138297.9
ENSG00000138400.8	ENSG00000138792.5	ENSG00000138801.4	ENSG00000139624.8
ENSG00000141140.12	ENSG00000142082.10	ENSG00000142973.8	ENSG00000143185.3
ENSG00000143443.9	ENSG00000143575.10	ENSG00000143776.14	ENSG00000143951.11
ENSG00000144021.2	ENSG00000144026.7	ENSG00000144118.9	ENSG00000144283.17
ENSG00000144362.7	ENSG00000145506.9	ENSG00000147364.12	ENSG00000147679.7
ENSG00000151176.3	ENSG00000151458.7	ENSG00000152253.4	ENSG00000153446.11
ENSG00000155066.11	ENSG00000155324.5	ENSG00000155846.12	ENSG00000157322.12
ENSG00000157578.9	ENSG00000157837.11	ENSG00000160207.4	ENSG00000163155.7
ENSG00000164880.11	ENSG00000164941.9	ENSG00000165650.7	ENSG00000166333.9
ENSG00000166341.6	ENSG00000166402.4	ENSG00000166669.9	ENSG00000166938.8
ENSG00000167333.8	ENSG00000167670.11	ENSG00000167740.5	ENSG00000167785.4
ENSG00000167842.11	ENSG00000168116.9	ENSG00000168411.9	ENSG00000168778.7
ENSG00000169413.2	ENSG00000169609.9	ENSG00000169981.6	ENSG00000169994.14
ENSG00000170175.6	ENSG00000170876.7	ENSG00000170906.11	ENSG00000171130.13
ENSG00000171863.8	ENSG00000171867.12	ENSG00000172007.5	ENSG00000172661.13
ENSG00000173200.8	ENSG00000173226.12	ENSG00000173915.8	ENSG00000174125.3
ENSG00000174194.11	ENSG00000174514.8	ENSG00000174529.6	ENSG00000174652.13
ENSG00000175182.9	ENSG00000175899.10	ENSG00000176155.14	ENSG00000176386.4
ENSG00000176998.3	ENSG00000177673.2	ENSG00000178201.3	ENSG00000178381.7
ENSG00000178386.8	ENSG00000180185.7	ENSG00000180481.6	ENSG00000180574.3
ENSG00000183049.8	ENSG00000183801.3	ENSG00000184389.8	ENSG00000184517.7
ENSG00000184602.5	ENSG00000184619.3	ENSG00000185621.7	ENSG00000186283.9
ENSG00000186960.6	ENSG00000188130.9	ENSG00000188321.9	ENSG00000188388.9
ENSG00000196167.5	ENSG00000196189.8	ENSG00000196313.7	ENSG00000196458.6
ENSG00000196743.4	ENSG00000197165.6	ENSG00000197375.8	ENSG00000197646.6
ENSG00000197885.6	ENSG00000198040.6	ENSG00000198336.5	ENSG00000203843.3
ENSG00000204520.8	ENSG00000204839.4	ENSG00000205784.2	ENSG00000205822.6
ENSG00000213762.6	ENSG00000215790.2	ENSG00000215861.4	ENSG00000216775.2
ENSG00000218891.2	ENSG00000221990.2	ENSG00000223496.1	ENSG00000223685.1
ENSG00000223804.1	ENSG00000224631.3	ENSG00000224690.2	ENSG00000225241.3
ENSG00000225851.1	ENSG00000227082.1	ENSG00000227388.2	ENSG00000227558.4
ENSG00000227725.2	ENSG00000228216.1	ENSG00000228307.1	ENSG00000231943.3
ENSG00000232063.1	ENSG00000232285.1	ENSG00000234618.1	ENSG00000234630.1
ENSG00000235098.4	ENSG00000235117.2	ENSG00000236624.4	ENSG00000236905.2
ENSG00000237510.3	ENSG00000241127.3	ENSG00000242441.3	ENSG00000243244.1
ENSG00000245937.3	ENSG00000247077.2	ENSG00000248408.1	ENSG00000249493.1

	ENSG00000250334.1	ENSG00000250427.1	ENSG00000250786.1	ENSG00000251580.1
	ENSG00000254319.1	ENSG00000255455.2	ENSG00000256223.1	ENSG00000258171.1
	ENSG00000259243.1	ENSG00000259539.1	ENSG00000260804.2	ENSG00000261556.4
	ENSG00000267623.2	ENSG00000267939.1	ENSG00000269086.2	ENSG00000269439.1
	ENSG00000269974.1	ENSG00000271361.1	ENSG00000271623.1	ENSG00000272459.1
	ENSG00000272462.2	ENSG00000272659.1	ENSG00000272824.1	ENSG00000273033.1
	ENSG00000273372.1	ENSG00000273477.1		
	ENSG00000001561.6	ENSG00000002834.13	ENSG00000005955.8	ENSG00000006007.7
	ENSG00000006114.11	ENSG00000013288.4	ENSG00000029725.12	ENSG00000047579.15
	ENSG00000054118.9	ENSG00000054179.7	ENSG00000054277.8	ENSG00000054611.9
	ENSG00000062650.13	ENSG00000063438.12	ENSG00000069188.12	ENSG00000073331.13
	ENSG00000075391.12	ENSG00000083099.6	ENSG00000084110.6	ENSG00000085982.9
	ENSG00000086598.6	ENSG00000087206.12	ENSG00000088881.16	ENSG00000089127.8
	ENSG00000089818.12	ENSG00000092036.12	ENSG00000095139.9	ENSG00000095906.12
	ENSG00000096093.10	ENSG00000096717.7	ENSG00000099256.14	ENSG00000099800.3
	ENSG00000099940.7	ENSG00000099956.13	ENSG00000100263.9	ENSG00000100290.2
	ENSG00000100359.16	ENSG00000100612.9	ENSG00000101190.8	ENSG00000101639.14
	ENSG00000103599.15	ENSG00000104852.10	ENSG00000107679.10	ENSG00000107854.5
	ENSG00000108264.12	ENSG00000108272.9	ENSG00000108278.7	ENSG00000108433.11
	ENSG00000108559.7	ENSG00000108590.6	ENSG00000108785.7	ENSG00000110777.7
	ENSG00000111679.12	ENSG00000111752.6	ENSG00000112200.12	ENSG00000112667.8
	ENSG00000113583.6	ENSG00000114107.4	ENSG00000114200.5	ENSG00000114503.6
	ENSG00000115947.9	ENSG00000116120.8	ENSG00000116641.11	ENSG00000116785.9
	ENSG00000116922.10	ENSG00000117174.6	ENSG00000117226.7	ENSG00000117834.8
	ENSG00000118557.11	ENSG00000118596.7	ENSG00000119227.3	ENSG00000119986.6
	ENSG00000120008.11	ENSG00000120451.6	ENSG00000120669.11	ENSG00000121579.8
	ENSG00000122375.7	ENSG00000122376.7	ENSG00000122870.7	ENSG00000125046.10
	ENSG00000125633.6	ENSG00000125901.5	ENSG00000125977.6	ENSG00000126231.9
	ENSG00000126602.6	ENSG00000126749.10	ENSG00000126773.8	ENSG00000126804.9
	ENSG00000127884.4	ENSG00000129353.10	ENSG00000129467.9	ENSG00000130244.8
	ENSG00000130713.11	ENSG00000131015.4	ENSG00000131188.7	ENSG00000131242.11
**Esophagus Mucosa**	ENSG00000132170.15	ENSG00000132274.11	ENSG00000132275.6	ENSG00000132517.10
	ENSG00000133477.12	ENSG00000133731.5	ENSG00000133983.10	ENSG00000134283.13
	ENSG00000134308.9	ENSG00000134716.5	ENSG00000134802.13	ENSG00000134905.12
	ENSG00000135002.7	ENSG00000135912.6	ENSG00000135929.4	ENSG00000136059.10
	ENSG00000136068.10	ENSG00000136235.11	ENSG00000136237.14	ENSG00000136717.10
	ENSG00000137171.10	ENSG00000137288.5	ENSG00000137411.12	ENSG00000138074.10
	ENSG00000138119.12	ENSG00000138138.9	ENSG00000138152.7	ENSG00000138400.8
	ENSG00000138600.5	ENSG00000138801.4	ENSG00000138814.12	ENSG00000139343.6
	ENSG00000139372.10	ENSG00000141140.12	ENSG00000141404.11	ENSG00000141505.7
	ENSG00000141639.7	ENSG00000141644.13	ENSG00000142065.9	ENSG00000142208.11
	ENSG00000142230.7	ENSG00000142330.15	ENSG00000142599.13	ENSG00000142856.12
	ENSG00000142973.8	ENSG00000143398.15	ENSG00000143556.4	ENSG00000143612.14
	ENSG00000143742.8	ENSG00000143799.8	ENSG00000144021.2	ENSG00000144026.7
	ENSG00000144366.11	ENSG00000144451.14	ENSG00000144566.6	ENSG00000144567.6
	ENSG00000145244.7	ENSG00000145287.6	ENSG00000145335.11	ENSG00000145388.10
	ENSG00000145390.7	ENSG00000145949.8	ENSG00000146540.10	ENSG00000146576.8
	ENSG00000146729.5	ENSG00000147676.9	ENSG00000148335.10	ENSG00000148672.7
	ENSG00000149043.12	ENSG00000149532.11	ENSG00000149571.6	ENSG00000149798.3

ENSG00000149968.7	ENSG00000150753.7	ENSG00000151176.3	ENSG00000151715.3
ENSG00000152240.8	ENSG00000152931.7	ENSG00000153246.7	ENSG00000155254.8
ENSG00000156642.12	ENSG00000156869.8	ENSG00000157837.11	ENSG00000158220.9
ENSG00000158234.8	ENSG00000158517.9	ENSG00000159110.15	ENSG00000159212.8
ENSG00000159322.13	ENSG00000160972.5	ENSG00000161904.7	ENSG00000162040.5
ENSG00000162576.12	ENSG00000162600.7	ENSG00000162994.11	ENSG00000163072.10
ENSG00000163214.16	ENSG00000163568.9	ENSG00000164111.10	ENSG00000164338.5
ENSG00000164347.13	ENSG00000164574.11	ENSG00000164855.11	ENSG00000164880.11
ENSG00000165181.12	ENSG00000165406.11	ENSG00000165646.7	ENSG00000165650.7
ENSG00000165899.6	ENSG00000166341.6	ENSG00000166452.7	ENSG00000167046.4
ENSG00000167384.6	ENSG00000167535.3	ENSG00000167674.10	ENSG00000167842.11
ENSG00000168703.5	ENSG00000169282.13	ENSG00000169609.9	ENSG00000169994.14
ENSG00000170191.4	ENSG00000171729.9	ENSG00000171943.7	ENSG00000171984.10
ENSG00000172262.7	ENSG00000172572.6	ENSG00000172661.13	ENSG00000173039.14
ENSG00000173175.10	ENSG00000173226.12	ENSG00000173578.6	ENSG00000174125.3
ENSG00000174194.11	ENSG00000174282.7	ENSG00000174652.13	ENSG00000175048.12
ENSG00000175634.10	ENSG00000175764.10	ENSG00000175899.10	ENSG00000176124.7
ENSG00000176155.14	ENSG00000176390.10	ENSG00000176998.3	ENSG00000177098.4
ENSG00000177875.3	ENSG00000178115.10	ENSG00000178297.8	ENSG00000178363.3
ENSG00000178386.8	ENSG00000178430.4	ENSG00000179397.13	ENSG00000179673.3
ENSG00000180185.7	ENSG00000180481.6	ENSG00000180530.5	ENSG00000180881.15
ENSG00000180953.7	ENSG00000181007.7	ENSG00000181027.6	ENSG00000182704.6
ENSG00000183020.9	ENSG00000184389.8	ENSG00000184428.8	ENSG00000184517.7
ENSG00000185269.7	ENSG00000185298.8	ENSG00000185344.9	ENSG00000185565.7
ENSG00000185627.13	ENSG00000185630.14	ENSG00000186026.6	ENSG00000186417.9
ENSG00000186448.10	ENSG00000186470.9	ENSG00000186652.5	ENSG00000186834.2
ENSG00000186889.5	ENSG00000187094.7	ENSG00000187608.5	ENSG00000188086.8
ENSG00000188460.4	ENSG00000188659.5	ENSG00000188811.8	ENSG00000188997.3
ENSG00000196109.6	ENSG00000196139.7	ENSG00000196172.8	ENSG00000196247.7
ENSG00000196663.11	ENSG00000196739.10	ENSG00000197291.4	ENSG00000197302.6
ENSG00000197746.9	ENSG00000197935.6	ENSG00000198089.10	ENSG00000198336.5
ENSG00000198648.6	ENSG00000198885.5	ENSG00000198929.8	ENSG00000198945.3
ENSG00000203697.7	ENSG00000203786.5	ENSG00000204520.8	ENSG00000204959.3
ENSG00000204977.5	ENSG00000205022.5	ENSG00000205572.5	ENSG00000205822.6
ENSG00000206527.5	ENSG00000212734.4	ENSG00000213366.8	ENSG00000213612.3
ENSG00000213626.7	ENSG00000213760.6	ENSG00000213900.2	ENSG00000214290.3
ENSG00000215440.7	ENSG00000215450.2	ENSG00000219545.5	ENSG00000221955.6
ENSG00000223345.3	ENSG00000223496.1	ENSG00000223558.1	ENSG00000224961.1
ENSG00000225784.5	ENSG00000225851.1	ENSG00000226237.1	ENSG00000227057.3
ENSG00000227131.1	ENSG00000227621.1	ENSG00000228106.1	ENSG00000228960.4
ENSG00000229660.1	ENSG00000229912.1	ENSG00000231205.7	ENSG00000231360.2
ENSG00000231793.4	ENSG00000231861.1	ENSG00000231925.7	ENSG00000232677.2
ENSG00000233217.1	ENSG00000233961.1	ENSG00000235076.2	ENSG00000235097.1
ENSG00000235098.4	ENSG00000236624.4	ENSG00000237510.3	ENSG00000237560.1
ENSG00000237927.1	ENSG00000239388.4	ENSG00000239887.3	ENSG00000240038.2
ENSG00000240654.2	ENSG00000240875.1	ENSG00000241316.2	ENSG00000242220.2
ENSG00000242441.3	ENSG00000242611.1	ENSG00000242689.1	ENSG00000243244.1
ENSG00000243414.4	ENSG00000243896.3	ENSG00000244723.3	ENSG00000245937.3
ENSG00000247596.4	ENSG00000250334.1	ENSG00000250571.2	ENSG00000250786.1

	ENSG00000251580.1	ENSG00000253330.1	ENSG00000253768.1	ENSG00000254272.1
	ENSG00000254531.1	ENSG00000254636.1	ENSG00000254761.1	ENSG00000255103.1
	ENSG00000256050.2	ENSG00000256223.1	ENSG00000256660.1	ENSG00000258768.2
	ENSG00000258967.1	ENSG00000259218.1	ENSG00000259539.1	ENSG00000259959.1
	ENSG00000260027.3	ENSG00000260077.1	ENSG00000261272.1	ENSG00000261556.4
	ENSG00000261754.2	ENSG00000262165.1	ENSG00000262973.1	ENSG00000263508.1
	ENSG00000263874.1	ENSG00000264538.2	ENSG00000266970.1	ENSG00000267069.1
	ENSG00000267325.1	ENSG00000270097.1	ENSG00000271550.1	ENSG00000271868.1
	ENSG00000272129.1	ENSG00000272361.1	ENSG00000272455.1	ENSG00000272462.2
**Esophagus Muscularis**	ENSG00000004777.14	ENSG00000005955.8	ENSG00000006283.13	ENSG00000013016.10
	ENSG00000013288.4	ENSG00000027847.9	ENSG00000029725.12	ENSG00000042062.7
	ENSG00000051108.10	ENSG00000051596.5	ENSG00000051620.6	ENSG00000054219.9
	ENSG00000055955.11	ENSG00000057663.8	ENSG00000064115.6	ENSG00000066855.11
	ENSG00000067221.9	ENSG00000070961.10	ENSG00000073921.13	ENSG00000074370.13
	ENSG00000075131.5	ENSG00000079950.9	ENSG00000083099.6	ENSG00000083814.8
	ENSG00000084444.9	ENSG00000087206.12	ENSG00000088387.13	ENSG00000092036.12
	ENSG00000099769.5	ENSG00000099954.14	ENSG00000099956.13	ENSG00000100079.5
	ENSG00000100888.8	ENSG00000100968.9	ENSG00000101193.6	ENSG00000101350.6
	ENSG00000101460.8	ENSG00000101639.14	ENSG00000102743.10	ENSG00000102763.11
	ENSG00000103429.6	ENSG00000104361.5	ENSG00000104723.16	ENSG00000104852.10
	ENSG00000104972.10	ENSG00000105341.14	ENSG00000105609.12	ENSG00000105639.14
	ENSG00000105655.14	ENSG00000105852.6	ENSG00000106605.6	ENSG00000107341.4
	ENSG00000107854.5	ENSG00000108010.7	ENSG00000108272.9	ENSG00000108352.7
	ENSG00000108785.7	ENSG00000108786.6	ENSG00000109576.9	ENSG00000109771.11
	ENSG00000110455.9	ENSG00000111237.14	ENSG00000111275.8	ENSG00000111664.6
	ENSG00000111665.7	ENSG00000111676.10	ENSG00000111752.6	ENSG00000111816.6
	ENSG00000111832.8	ENSG00000111907.16	ENSG00000111913.11	ENSG00000112394.12
	ENSG00000112773.11	ENSG00000112902.7	ENSG00000113361.8	ENSG00000113739.6
	ENSG00000114735.5	ENSG00000114742.9	ENSG00000115808.7	ENSG00000116785.9
	ENSG00000116957.8	ENSG00000117226.7	ENSG00000117477.8	ENSG00000118004.13
	ENSG00000118402.5	ENSG00000118705.12	ENSG00000118997.9	ENSG00000120008.11
	ENSG00000120669.11	ENSG00000120910.10	ENSG00000121236.15	ENSG00000121289.13
	ENSG00000121879.3	ENSG00000122335.9	ENSG00000122376.7	ENSG00000122870.7
	ENSG00000124275.10	ENSG00000125733.13	ENSG00000125885.9	ENSG00000126226.17
	ENSG00000126231.9	ENSG00000126822.11	ENSG00000127903.12	ENSG00000128218.7
	ENSG00000128609.10	ENSG00000129566.8	ENSG00000130024.10	ENSG00000130377.9
	ENSG00000131697.13	ENSG00000132321.12	ENSG00000132781.13	ENSG00000133731.5
	ENSG00000133740.6	ENSG00000133983.10	ENSG00000134905.12	ENSG00000135709.8
	ENSG00000135914.5	ENSG00000136014.7	ENSG00000137288.5	ENSG00000137414.5
	ENSG00000137992.10	ENSG00000137996.8	ENSG00000138029.9	ENSG00000138152.7
	ENSG00000138172.6	ENSG00000138792.5	ENSG00000138801.4	ENSG00000139178.6
	ENSG00000139278.5	ENSG00000140543.9	ENSG00000140545.10	ENSG00000140943.12
	ENSG00000141140.12	ENSG00000141404.11	ENSG00000142973.8	ENSG00000143155.8
	ENSG00000143514.12	ENSG00000143924.14	ENSG00000144026.7	ENSG00000145244.7
	ENSG00000145569.5	ENSG00000146904.4	ENSG00000147669.6	ENSG00000148843.9
	ENSG00000149474.9	ENSG00000150753.7	ENSG00000151176.3	ENSG00000151239.9
	ENSG00000151348.9	ENSG00000152253.4	ENSG00000152455.11	ENSG00000152465.13
	ENSG00000153048.6	ENSG00000153246.7	ENSG00000153404.9	ENSG00000153446.11
	ENSG00000155254.8	ENSG00000156269.4	ENSG00000156502.9	ENSG00000156886.11

ENSG00000157578.9	ENSG00000157837.11	ENSG00000158106.8	ENSG00000158234.8
ENSG00000158669.7	ENSG00000159082.13	ENSG00000159592.6	ENSG00000159596.6
ENSG00000160072.15	ENSG00000160172.6	ENSG00000160404.13	ENSG00000162365.7
ENSG00000162664.12	ENSG00000162687.12	ENSG00000163394.5	ENSG00000163629.8
ENSG00000163959.5	ENSG00000164128.2	ENSG00000164176.8	ENSG00000164828.13
ENSG00000164880.11	ENSG00000164941.9	ENSG00000165646.7	ENSG00000165650.7
ENSG00000166333.9	ENSG00000166762.12	ENSG00000166913.8	ENSG00000167333.8
ENSG00000167377.13	ENSG00000167468.12	ENSG00000167740.5	ENSG00000167842.11
ENSG00000168291.8	ENSG00000169228.9	ENSG00000169609.9	ENSG00000169710.6
ENSG00000169758.8	ENSG00000169994.14	ENSG00000170035.11	ENSG00000170412.12
ENSG00000170906.11	ENSG00000171124.8	ENSG00000171862.5	ENSG00000172071.7
ENSG00000172661.13	ENSG00000172671.15	ENSG00000173253.10	ENSG00000174125.3
ENSG00000174194.11	ENSG00000174407.7	ENSG00000174652.13	ENSG00000175985.8
ENSG00000176046.7	ENSG00000176155.14	ENSG00000177030.12	ENSG00000178381.7
ENSG00000178386.8	ENSG00000179673.3	ENSG00000179978.10	ENSG00000180071.14
ENSG00000180185.7	ENSG00000180211.5	ENSG00000180481.6	ENSG00000180773.10
ENSG00000180881.15	ENSG00000180953.7	ENSG00000182551.9	ENSG00000182568.12
ENSG00000182771.13	ENSG00000183111.7	ENSG00000183260.5	ENSG00000183423.7
ENSG00000183735.5	ENSG00000183748.4	ENSG00000184007.13	ENSG00000184389.8
ENSG00000184517.7	ENSG00000184602.5	ENSG00000185340.11	ENSG00000185710.5
ENSG00000185722.12	ENSG00000185963.9	ENSG00000186160.4	ENSG00000186283.9
ENSG00000186470.9	ENSG00000187954.8	ENSG00000188130.9	ENSG00000188385.7
ENSG00000188659.5	ENSG00000189280.3	ENSG00000196458.6	ENSG00000196705.4
ENSG00000196743.4	ENSG00000196967.6	ENSG00000197124.7	ENSG00000197646.6
ENSG00000197747.4	ENSG00000197885.6	ENSG00000198171.8	ENSG00000198336.5
ENSG00000198805.7	ENSG00000198885.5	ENSG00000198931.6	ENSG00000198945.3
ENSG00000203843.3	ENSG00000204136.6	ENSG00000204344.10	ENSG00000204381.7
ENSG00000204421.2	ENSG00000204520.8	ENSG00000204856.7	ENSG00000204959.3
ENSG00000205578.4	ENSG00000205581.6	ENSG00000205822.6	ENSG00000213512.1
ENSG00000213533.7	ENSG00000213626.7	ENSG00000213753.6	ENSG00000213930.7
ENSG00000214243.3	ENSG00000214562.9	ENSG00000214944.5	ENSG00000215712.6
ENSG00000215861.4	ENSG00000217128.7	ENSG00000218891.2	ENSG00000219102.3
ENSG00000219355.2	ENSG00000219545.5	ENSG00000222020.2	ENSG00000223431.1
ENSG00000223496.1	ENSG00000224389.4	ENSG00000225241.3	ENSG00000225492.2
ENSG00000225851.1	ENSG00000226133.1	ENSG00000227160.2	ENSG00000227176.1
ENSG00000227345.4	ENSG00000227359.1	ENSG00000227388.2	ENSG00000227755.1
ENSG00000228561.2	ENSG00000229274.1	ENSG00000231360.2	ENSG00000231861.1
ENSG00000232063.1	ENSG00000232224.1	ENSG00000232587.1	ENSG00000232677.2
ENSG00000233590.1	ENSG00000233961.1	ENSG00000235098.4	ENSG00000235374.1
ENSG00000236104.2	ENSG00000236233.3	ENSG00000236297.1	ENSG00000236444.2
ENSG00000236624.4	ENSG00000236801.1	ENSG00000237176.3	ENSG00000237510.3
ENSG00000240038.2	ENSG00000241043.1	ENSG00000242441.3	ENSG00000242611.1
ENSG00000243244.1	ENSG00000244414.2	ENSG00000244754.4	ENSG00000245937.3
ENSG00000248408.1	ENSG00000248487.4	ENSG00000249646.2	ENSG00000250075.1
ENSG00000250334.1	ENSG00000250645.1	ENSG00000250786.1	ENSG00000250848.1
ENSG00000253598.1	ENSG00000253704.1	ENSG00000255000.1	ENSG00000255418.1
ENSG00000259539.1	ENSG00000259959.1	ENSG00000260105.2	ENSG00000260274.1
ENSG00000260558.1	ENSG00000260645.1	ENSG00000261076.1	ENSG00000261098.1
ENSG00000261193.1	ENSG00000261556.4	ENSG00000265478.1	ENSG00000266912.1

	ENSG00000267575.2	ENSG00000267868.1	ENSG00000267939.1	ENSG00000268442.1
	ENSG00000268927.1	ENSG00000269514.1	ENSG00000270614.1	ENSG00000271396.1
	ENSG00000271550.1	ENSG00000271623.1	ENSG00000272129.1	ENSG00000272201.1
	ENSG00000272462.2	ENSG00000272810.1	ENSG00000273356.1	
**Heart Atrial**	ENSG00000013288.4	ENSG00000025039.10	ENSG00000028203.13	ENSG00000037897.12
	ENSG00000040487.8	ENSG00000050327.10	ENSG00000063438.12	ENSG00000064651.9
	ENSG00000066248.10	ENSG00000066855.11	ENSG00000067221.9	ENSG00000073008.10
	ENSG00000075239.9	ENSG00000078124.7	ENSG00000079785.10	ENSG00000081791.4
	ENSG00000083099.6	ENSG00000086200.12	ENSG00000088538.12	ENSG00000090273.9
	ENSG00000091262.10	ENSG00000091879.9	ENSG00000092036.12	ENSG00000099800.3
	ENSG00000099956.13	ENSG00000099957.12	ENSG00000100181.17	ENSG00000100266.13
	ENSG00000101255.6	ENSG00000101310.10	ENSG00000101460.8	ENSG00000101608.8
	ENSG00000102743.10	ENSG00000103168.12	ENSG00000103472.5	ENSG00000104361.5
	ENSG00000105341.14	ENSG00000105738.6	ENSG00000106330.7	ENSG00000106511.5
	ENSG00000106603.13	ENSG00000107262.12	ENSG00000107404.13	ENSG00000107854.5
	ENSG00000107862.4	ENSG00000107874.6	ENSG00000108264.12	ENSG00000108799.8
	ENSG00000109163.6	ENSG00000109576.9	ENSG00000110934.6	ENSG00000111224.9
	ENSG00000111237.14	ENSG00000111254.3	ENSG00000111275.8	ENSG00000111364.11
	ENSG00000111752.6	ENSG00000112137.12	ENSG00000112164.5	ENSG00000112210.7
	ENSG00000112787.8	ENSG00000116120.8	ENSG00000116785.9	ENSG00000116957.8
	ENSG00000117226.7	ENSG00000118369.8	ENSG00000118596.7	ENSG00000118729.10
	ENSG00000119147.5	ENSG00000120334.11	ENSG00000120451.6	ENSG00000120539.10
	ENSG00000120833.9	ENSG00000123178.10	ENSG00000123191.9	ENSG00000123219.8
	ENSG00000123836.10	ENSG00000124406.12	ENSG00000124508.12	ENSG00000125885.9
	ENSG00000127249.10	ENSG00000127948.9	ENSG00000128218.7	ENSG00000130649.5
	ENSG00000131386.13	ENSG00000131401.7	ENSG00000131732.7	ENSG00000132002.3
	ENSG00000132681.12	ENSG00000133641.13	ENSG00000133983.10	ENSG00000134905.12
	ENSG00000135272.5	ENSG00000135956.4	ENSG00000136003.11	ENSG00000136235.11
	ENSG00000136827.11	ENSG00000136875.8	ENSG00000136877.10	ENSG00000137880.4
	ENSG00000137992.10	ENSG00000137996.8	ENSG00000138172.6	ENSG00000138760.4
	ENSG00000139200.9	ENSG00000139278.5	ENSG00000139343.6	ENSG00000139624.8
	ENSG00000140406.2	ENSG00000141140.12	ENSG00000142065.9	ENSG00000142937.7
	ENSG00000143155.8	ENSG00000143575.10	ENSG00000143924.14	ENSG00000143951.11
	ENSG00000144026.7	ENSG00000146904.4	ENSG00000148288.7	ENSG00000148429.10
	ENSG00000151348.9	ENSG00000151413.12	ENSG00000152049.5	ENSG00000153140.4
	ENSG00000153246.7	ENSG00000154589.2	ENSG00000155256.13	ENSG00000155324.5
	ENSG00000155368.12	ENSG00000156475.14	ENSG00000157911.5	ENSG00000158669.7
	ENSG00000159346.8	ENSG00000159445.8	ENSG00000159455.7	ENSG00000159596.6
	ENSG00000160271.10	ENSG00000160294.6	ENSG00000161203.9	ENSG00000162384.9
	ENSG00000162512.11	ENSG00000162722.8	ENSG00000163636.6	ENSG00000163931.11
	ENSG00000164880.11	ENSG00000165406.11	ENSG00000165646.7	ENSG00000165650.7
	ENSG00000165905.12	ENSG00000165996.9	ENSG00000166171.8	ENSG00000166426.7
	ENSG00000166913.8	ENSG00000166938.8	ENSG00000167081.12	ENSG00000167565.8
	ENSG00000168143.8	ENSG00000168918.9	ENSG00000169189.12	ENSG00000170604.3
	ENSG00000170906.11	ENSG00000170915.8	ENSG00000171533.7	ENSG00000171649.7
	ENSG00000171943.7	ENSG00000172671.15	ENSG00000173226.12	ENSG00000173915.8
	ENSG00000174007.7	ENSG00000174194.11	ENSG00000174529.6	ENSG00000174652.13
	ENSG00000175352.6	ENSG00000175764.10	ENSG00000176155.14	ENSG00000176595.3
	ENSG00000176903.3	ENSG00000176998.3	ENSG00000177590.6	ENSG00000178386.8

	ENSG00000178397.8	ENSG00000178796.8	ENSG00000179954.10	ENSG00000180113.11
	ENSG00000180185.7	ENSG00000180481.6	ENSG00000180773.10	ENSG00000182732.12
	ENSG00000182771.13	ENSG00000183444.10	ENSG00000184110.10	ENSG00000184389.8
	ENSG00000184557.3	ENSG00000185669.5	ENSG00000185739.9	ENSG00000185946.11
	ENSG00000186470.9	ENSG00000186532.7	ENSG00000186907.3	ENSG00000187193.8
	ENSG00000187676.7	ENSG00000188257.6	ENSG00000188659.5	ENSG00000188662.5
	ENSG00000189362.7	ENSG00000196611.4	ENSG00000196743.4	ENSG00000197375.8
	ENSG00000197646.6	ENSG00000198768.6	ENSG00000198885.5	ENSG00000203724.6
	ENSG00000204237.4	ENSG00000204287.9	ENSG00000204348.5	ENSG00000204520.8
	ENSG00000204652.5	ENSG00000204977.5	ENSG00000205281.6	ENSG00000206527.5
	ENSG00000213588.4	ENSG00000214293.4	ENSG00000215861.4	ENSG00000216775.2
	ENSG00000218336.3	ENSG00000219481.6	ENSG00000223496.1	ENSG00000224914.2
	ENSG00000225241.3	ENSG00000226005.3	ENSG00000226133.1	ENSG00000227176.1
	ENSG00000227345.4	ENSG00000228031.2	ENSG00000228421.2	ENSG00000229298.1
	ENSG00000231205.7	ENSG00000231360.2	ENSG00000231793.4	ENSG00000231861.1
	ENSG00000231925.7	ENSG00000232527.3	ENSG00000232677.2	ENSG00000232888.3
	ENSG00000235098.4	ENSG00000235271.1	ENSG00000236417.2	ENSG00000236624.4
	ENSG00000237161.3	ENSG00000237510.3	ENSG00000240038.2	ENSG00000241015.2
	ENSG00000241043.1	ENSG00000241278.1	ENSG00000242441.3	ENSG00000243414.4
	ENSG00000243444.3	ENSG00000243819.3	ENSG00000245937.3	ENSG00000248079.2
	ENSG00000248487.4	ENSG00000249129.1	ENSG00000249159.2	ENSG00000249915.3
	ENSG00000250075.1	ENSG00000250334.1	ENSG00000250571.2	ENSG00000251580.1
	ENSG00000253250.2	ENSG00000253797.2	ENSG00000255318.1	ENSG00000255374.1
	ENSG00000256433.1	ENSG00000256540.1	ENSG00000259344.1	ENSG00000259417.2
	ENSG00000259616.1	ENSG00000259982.1	ENSG00000260077.1	ENSG00000261079.1
	ENSG00000261455.1	ENSG00000261549.2	ENSG00000261556.4	ENSG00000262165.1
	ENSG00000266912.1	ENSG00000267623.2	ENSG00000267885.1	ENSG00000267939.1
	ENSG00000268442.1	ENSG00000270661.1	ENSG00000271040.1	ENSG00000271550.1
	ENSG00000272462.2	ENSG00000272567.1	ENSG00000272810.1	
**Heart Left Ventricle**	ENSG00000001561.6	ENSG00000003249.9	ENSG00000013288.4	ENSG00000019505.3
	ENSG00000026950.12	ENSG00000043514.11	ENSG00000049449.4	ENSG00000068781.16
	ENSG00000069493.10	ENSG00000076685.14	ENSG00000077348.4	ENSG00000079785.10
	ENSG00000083857.9	ENSG00000085063.10	ENSG00000086015.16	ENSG00000088538.12
	ENSG00000088992.13	ENSG00000091542.8	ENSG00000091986.11	ENSG00000092036.12
	ENSG00000099338.18	ENSG00000099953.5	ENSG00000099956.13	ENSG00000100461.13
	ENSG00000101222.8	ENSG00000101255.6	ENSG00000101493.6	ENSG00000101608.8
	ENSG00000102743.10	ENSG00000103313.7	ENSG00000103335.15	ENSG00000105227.10
	ENSG00000105341.14	ENSG00000105738.6	ENSG00000107854.5	ENSG00000107859.5
	ENSG00000108176.10	ENSG00000108272.9	ENSG00000108963.13	ENSG00000110395.4
	ENSG00000111300.5	ENSG00000112164.5	ENSG00000112294.8	ENSG00000113851.9
	ENSG00000114098.13	ENSG00000114779.15	ENSG00000115902.6	ENSG00000116120.8
	ENSG00000116273.5	ENSG00000116574.4	ENSG00000116690.7	ENSG00000117174.6
	ENSG00000117226.7	ENSG00000118369.8	ENSG00000119715.10	ENSG00000120907.13
	ENSG00000123453.12	ENSG00000123836.10	ENSG00000124172.5	ENSG00000125945.10
	ENSG00000126088.8	ENSG00000126231.9	ENSG00000126773.8	ENSG00000126804.9
	ENSG00000127990.11	ENSG00000129467.9	ENSG00000130177.10	ENSG00000130684.9
	ENSG00000131401.7	ENSG00000131732.7	ENSG00000133983.10	ENSG00000134077.11
	ENSG00000135914.5	ENSG00000136003.11	ENSG00000136169.12	ENSG00000136235.11
	ENSG00000136717.10	ENSG00000136875.8	ENSG00000137714.2	ENSG00000137760.10

	ENSG00000137819.9	ENSG00000137942.12	ENSG00000137996.8	ENSG00000138152.7
	ENSG00000138172.6	ENSG00000138400.8	ENSG00000138448.7	ENSG00000138472.6
	ENSG00000138760.4	ENSG00000138801.4	ENSG00000139182.9	ENSG00000140406.2
	ENSG00000140937.9	ENSG00000141012.8	ENSG00000141127.10	ENSG00000141140.12
	ENSG00000142973.8	ENSG00000143155.8	ENSG00000143486.11	ENSG00000143924.14
	ENSG00000144021.2	ENSG00000144362.7	ENSG00000146904.4	ENSG00000147364.12
	ENSG00000147679.7	ENSG00000149289.6	ENSG00000151176.3	ENSG00000151348.9
	ENSG00000151474.15	ENSG00000152253.4	ENSG00000152382.5	ENSG00000152465.13
	ENSG00000153786.8	ENSG00000157259.6	ENSG00000157322.12	ENSG00000157837.11
	ENSG00000157911.5	ENSG00000158987.15	ENSG00000159899.10	ENSG00000160226.11
	ENSG00000162643.8	ENSG00000162994.11	ENSG00000163354.10	ENSG00000163683.7
	ENSG00000164451.9	ENSG00000164849.7	ENSG00000164880.11	ENSG00000165406.11
	ENSG00000165646.7	ENSG00000165650.7	ENSG00000166268.6	ENSG00000166405.10
	ENSG00000166546.9	ENSG00000166548.11	ENSG00000167094.11	ENSG00000167680.11
	ENSG00000167840.9	ENSG00000167842.11	ENSG00000169609.9	ENSG00000169994.14
	ENSG00000170175.6	ENSG00000170846.11	ENSG00000170906.11	ENSG00000171724.2
	ENSG00000171729.9	ENSG00000171943.7	ENSG00000172215.5	ENSG00000172399.5
	ENSG00000172661.13	ENSG00000172671.15	ENSG00000173175.10	ENSG00000173226.12
	ENSG00000173253.10	ENSG00000173517.6	ENSG00000173846.8	ENSG00000174007.7
	ENSG00000174652.13	ENSG00000175426.6	ENSG00000176155.14	ENSG00000176998.3
	ENSG00000177590.6	ENSG00000178386.8	ENSG00000180104.11	ENSG00000180113.11
	ENSG00000180185.7	ENSG00000180481.6	ENSG00000182568.12	ENSG00000182771.13
	ENSG00000183208.8	ENSG00000183444.10	ENSG00000184014.3	ENSG00000184389.8
	ENSG00000184602.5	ENSG00000185483.7	ENSG00000186026.6	ENSG00000186448.10
	ENSG00000186470.9	ENSG00000187554.7	ENSG00000188243.8	ENSG00000188659.5
	ENSG00000188687.11	ENSG00000188735.8	ENSG00000189091.8	ENSG00000196611.4
	ENSG00000196743.4	ENSG00000197256.6	ENSG00000197646.6	ENSG00000197860.5
	ENSG00000198171.8	ENSG00000198624.8	ENSG00000198885.5	ENSG00000204020.5
	ENSG00000204161.9	ENSG00000204301.5	ENSG00000204520.8	ENSG00000204574.8
	ENSG00000204764.8	ENSG00000204959.3	ENSG00000204978.2	ENSG00000205362.6
	ENSG00000205822.6	ENSG00000213626.7	ENSG00000214135.4	ENSG00000215861.4
	ENSG00000215914.3	ENSG00000216775.2	ENSG00000217644.4	ENSG00000223496.1
	ENSG00000223865.6	ENSG00000224843.2	ENSG00000224914.2	ENSG00000225241.3
	ENSG00000225851.1	ENSG00000226816.2	ENSG00000227160.2	ENSG00000227176.1
	ENSG00000227388.2	ENSG00000229246.1	ENSG00000231925.7	ENSG00000232063.1
	ENSG00000232224.1	ENSG00000235387.1	ENSG00000236624.4	ENSG00000237176.3
	ENSG00000237510.3	ENSG00000237988.2	ENSG00000241015.2	ENSG00000242267.2
	ENSG00000244050.2	ENSG00000245937.3	ENSG00000248487.4	ENSG00000249915.3
	ENSG00000250056.1	ENSG00000250075.1	ENSG00000253540.1	ENSG00000255479.1
	ENSG00000255513.1	ENSG00000256223.1	ENSG00000256433.1	ENSG00000256594.3
	ENSG00000260105.2	ENSG00000261556.4	ENSG00000261672.1	ENSG00000265096.1
	ENSG00000266912.1	ENSG00000270115.1	ENSG00000270972.1	ENSG00000271499.1
	ENSG00000271550.1	ENSG00000272958.1		
	ENSG00000004777.14	ENSG00000005059.11	ENSG00000008513.10	ENSG00000013288.4
	ENSG00000016402.8	ENSG00000026950.12	ENSG00000064115.6	ENSG00000064651.9
	ENSG00000072832.10	ENSG00000076344.11	ENSG00000079387.9	ENSG00000079974.13
	ENSG00000082212.7	ENSG00000083896.8	ENSG00000085733.11	ENSG00000087250.4
	ENSG00000088826.13	ENSG00000090006.13	ENSG00000092036.12	ENSG00000099800.3
**Liver**	ENSG00000100612.9	ENSG00000101204.11	ENSG00000101460.8	ENSG00000103351.8

	ENSG00000105136.15	ENSG00000106133.13	ENSG00000107854.5	ENSG00000108264.12
	ENSG00000108433.11	ENSG00000111328.2	ENSG00000112175.6	ENSG00000117533.10
	ENSG00000117676.9	ENSG00000120451.6	ENSG00000121454.4	ENSG00000122376.7
	ENSG00000122678.10	ENSG00000125753.9	ENSG00000128928.4	ENSG00000129204.12
	ENSG00000131795.8	ENSG00000133106.10	ENSG00000133983.10	ENSG00000134884.9
	ENSG00000135164.14	ENSG00000135341.13	ENSG00000135750.10	ENSG00000135913.6
	ENSG00000136250.7	ENSG00000136717.10	ENSG00000136783.9	ENSG00000137404.10
	ENSG00000137411.12	ENSG00000139178.6	ENSG00000139370.6	ENSG00000139644.8
	ENSG00000141140.12	ENSG00000142856.12	ENSG00000143195.8	ENSG00000144021.2
	ENSG00000146205.9	ENSG00000151948.7	ENSG00000152382.5	ENSG00000153933.5
	ENSG00000154309.7	ENSG00000156172.5	ENSG00000156234.7	ENSG00000157322.12
	ENSG00000158669.7	ENSG00000160226.11	ENSG00000160710.11	ENSG00000161326.8
	ENSG00000162994.11	ENSG00000164880.11	ENSG00000165389.6	ENSG00000165568.13
	ENSG00000166006.8	ENSG00000166140.13	ENSG00000167165.14	ENSG00000167674.10
	ENSG00000167797.3	ENSG00000167800.9	ENSG00000167977.4	ENSG00000168065.11
	ENSG00000168268.6	ENSG00000168411.9	ENSG00000169733.7	ENSG00000170846.11
	ENSG00000170906.11	ENSG00000172586.7	ENSG00000173040.8	ENSG00000174007.7
	ENSG00000174358.11	ENSG00000174652.13	ENSG00000175356.8	ENSG00000176182.5
	ENSG00000176386.4	ENSG00000178297.8	ENSG00000178449.4	ENSG00000179562.2
	ENSG00000180185.7	ENSG00000180481.6	ENSG00000182134.11	ENSG00000183260.5
	ENSG00000183765.16	ENSG00000186470.9	ENSG00000187105.4	ENSG00000188659.5
	ENSG00000189366.5	ENSG00000196418.8	ENSG00000196653.7	ENSG00000197272.2
	ENSG00000197498.8	ENSG00000197646.6	ENSG00000197980.7	ENSG00000198060.5
	ENSG00000198108.3	ENSG00000198467.9	ENSG00000204264.4	ENSG00000204308.6
	ENSG00000204520.8	ENSG00000204959.3	ENSG00000211452.6	ENSG00000213315.4
	ENSG00000213626.7	ENSG00000215187.5	ENSG00000215267.4	ENSG00000221887.4
	ENSG00000223496.1	ENSG00000225241.3	ENSG00000225489.2	ENSG00000225492.2
	ENSG00000225851.1	ENSG00000226091.3	ENSG00000227107.1	ENSG00000227621.1
	ENSG00000230897.1	ENSG00000231412.2	ENSG00000232629.4	ENSG00000233215.1
	ENSG00000236624.4	ENSG00000237541.3	ENSG00000240065.3	ENSG00000242193.5
	ENSG00000244414.2	ENSG00000244752.2	ENSG00000245937.3	ENSG00000250305.4
	ENSG00000250786.1	ENSG00000251323.2	ENSG00000251417.1	ENSG00000254685.2
	ENSG00000254761.1	ENSG00000255455.2	ENSG00000255642.1	ENSG00000256528.1
	ENSG00000256720.1	ENSG00000259758.1	ENSG00000259967.1	ENSG00000259982.1
	ENSG00000260805.1	ENSG00000263082.1	ENSG00000266920.1	ENSG00000267623.2
	ENSG00000267659.1	ENSG00000269837.1	ENSG00000269996.1	ENSG00000272274.1
**Lung**	ENSG00000001561.6	ENSG00000003056.3	ENSG00000004777.14	ENSG00000005955.8
	ENSG00000007520.3	ENSG00000007541.10	ENSG00000008516.12	ENSG00000013016.10
	ENSG00000013288.4	ENSG00000027847.9	ENSG00000029725.12	ENSG00000032389.8
	ENSG00000051620.6	ENSG00000054219.9	ENSG00000063127.11	ENSG00000068781.16
	ENSG00000069431.6	ENSG00000069998.8	ENSG00000070759.12	ENSG00000070961.10
	ENSG00000075239.9	ENSG00000077348.4	ENSG00000083099.6	ENSG00000085415.11
	ENSG00000085719.7	ENSG00000086015.16	ENSG00000086288.7	ENSG00000086730.12
	ENSG00000090097.16	ENSG00000091262.10	ENSG00000092036.12	ENSG00000099956.13
	ENSG00000099957.12	ENSG00000100201.14	ENSG00000100206.5	ENSG00000100266.13
	ENSG00000100416.8	ENSG00000100490.5	ENSG00000100941.4	ENSG00000100983.5
	ENSG00000101161.6	ENSG00000101194.13	ENSG00000101224.13	ENSG00000101608.8
	ENSG00000102531.12	ENSG00000102996.4	ENSG00000103313.7	ENSG00000103599.15
	ENSG00000104361.5	ENSG00000104415.9	ENSG00000105290.7	ENSG00000107854.5

ENSG00000107874.6	ENSG00000108064.6	ENSG00000108094.10	ENSG00000108264.12
ENSG00000108272.9	ENSG00000108278.7	ENSG00000108352.7	ENSG00000108375.8
ENSG00000108829.9	ENSG00000111530.8	ENSG00000112294.8	ENSG00000112308.8
ENSG00000112667.8	ENSG00000115419.8	ENSG00000115568.11	ENSG00000115947.9
ENSG00000116785.9	ENSG00000116957.8	ENSG00000117523.11	ENSG00000117533.10
ENSG00000119537.11	ENSG00000120057.4	ENSG00000120451.6	ENSG00000120539.10
ENSG00000120669.11	ENSG00000121236.15	ENSG00000121594.7	ENSG00000121690.5
ENSG00000122025.10	ENSG00000122376.7	ENSG00000122692.7	ENSG00000122694.11
ENSG00000123352.13	ENSG00000123838.6	ENSG00000124788.13	ENSG00000125633.6
ENSG00000125743.6	ENSG00000127990.11	ENSG00000130684.9	ENSG00000131389.12
ENSG00000132623.11	ENSG00000133115.7	ENSG00000134256.8	ENSG00000134884.9
ENSG00000135333.9	ENSG00000135905.14	ENSG00000136371.5	ENSG00000136717.10
ENSG00000136868.9	ENSG00000136877.10	ENSG00000137070.13	ENSG00000137996.8
ENSG00000138029.9	ENSG00000138119.12	ENSG00000138382.9	ENSG00000138792.5
ENSG00000139144.5	ENSG00000139278.5	ENSG00000140406.2	ENSG00000140853.11
ENSG00000141140.12	ENSG00000142208.11	ENSG00000142599.13	ENSG00000142973.8
ENSG00000143155.8	ENSG00000143303.7	ENSG00000143919.10	ENSG00000143924.14
ENSG00000143951.11	ENSG00000144026.7	ENSG00000144366.11	ENSG00000144671.6
ENSG00000145390.7	ENSG00000148110.11	ENSG00000148634.11	ENSG00000148843.9
ENSG00000150526.7	ENSG00000152766.5	ENSG00000153048.6	ENSG00000153246.7
ENSG00000154198.10	ENSG00000155254.8	ENSG00000155256.13	ENSG00000155324.5
ENSG00000155849.11	ENSG00000156261.8	ENSG00000157224.11	ENSG00000157322.12
ENSG00000157379.9	ENSG00000159352.11	ENSG00000159445.8	ENSG00000159840.11
ENSG00000160710.11	ENSG00000161896.6	ENSG00000162039.10	ENSG00000162643.8
ENSG00000163106.6	ENSG00000163608.10	ENSG00000163762.2	ENSG00000164035.5
ENSG00000164294.9	ENSG00000164404.4	ENSG00000164880.11	ENSG00000164951.11
ENSG00000165084.11	ENSG00000165406.11	ENSG00000165650.7	ENSG00000165752.12
ENSG00000166145.10	ENSG00000166333.9	ENSG00000166589.8	ENSG00000166669.9
ENSG00000166938.8	ENSG00000167566.12	ENSG00000167641.6	ENSG00000167670.11
ENSG00000167842.11	ENSG00000168283.9	ENSG00000168334.8	ENSG00000168397.12
ENSG00000168411.9	ENSG00000169609.9	ENSG00000169710.6	ENSG00000170175.6
ENSG00000170191.4	ENSG00000170509.7	ENSG00000171234.9	ENSG00000171246.5
ENSG00000171533.7	ENSG00000171649.7	ENSG00000172340.10	ENSG00000173064.6
ENSG00000173852.9	ENSG00000174194.11	ENSG00000174353.13	ENSG00000174514.8
ENSG00000174652.13	ENSG00000174886.8	ENSG00000175264.3	ENSG00000175497.12
ENSG00000176155.14	ENSG00000176386.4	ENSG00000176834.9	ENSG00000176953.6
ENSG00000176998.3	ENSG00000177191.2	ENSG00000177551.5	ENSG00000177706.8
ENSG00000178386.8	ENSG00000178802.13	ENSG00000178952.4	ENSG00000179058.5
ENSG00000179988.9	ENSG00000180185.7	ENSG00000180353.6	ENSG00000180481.6
ENSG00000180914.6	ENSG00000181027.6	ENSG00000182185.14	ENSG00000182568.12
ENSG00000182851.2	ENSG00000183248.7	ENSG00000183506.12	ENSG00000183748.4
ENSG00000184313.15	ENSG00000184374.2	ENSG00000184389.8	ENSG00000184517.7
ENSG00000185344.9	ENSG00000185507.15	ENSG00000185522.4	ENSG00000185641.5
ENSG00000185689.11	ENSG00000185742.6	ENSG00000186160.4	ENSG00000186842.4
ENSG00000186998.11	ENSG00000187068.2	ENSG00000187091.9	ENSG00000187116.9
ENSG00000187193.8	ENSG00000188312.9	ENSG00000188659.5	ENSG00000188807.8
ENSG00000188888.7	ENSG00000189091.8	ENSG00000189127.3	ENSG00000189136.4
ENSG00000189241.6	ENSG00000189280.3	ENSG00000196109.6	ENSG00000196743.4
ENSG00000196793.9	ENSG00000196878.8	ENSG00000197146.2	ENSG00000197646.6

	ENSG00000198336.5	ENSG00000198399.10	ENSG00000198569.5	ENSG00000198829.5
	ENSG00000198842.5	ENSG00000198873.10	ENSG00000198885.5	ENSG00000198959.7
	ENSG00000203963.7	ENSG00000204020.5	ENSG00000204209.6	ENSG00000204237.4
	ENSG00000204520.8	ENSG00000204544.5	ENSG00000204789.3	ENSG00000204856.7
	ENSG00000204920.6	ENSG00000204977.5	ENSG00000205020.7	ENSG00000205085.4
	ENSG00000205822.6	ENSG00000211455.3	ENSG00000212125.2	ENSG00000213203.2
	ENSG00000213366.8	ENSG00000213626.7	ENSG00000213965.3	ENSG00000214439.3
	ENSG00000215861.4	ENSG00000218358.2	ENSG00000219545.5	ENSG00000223345.3
	ENSG00000223496.1	ENSG00000223561.2	ENSG00000224531.4	ENSG00000224914.2
	ENSG00000225518.2	ENSG00000225603.3	ENSG00000225683.1	ENSG00000225720.2
	ENSG00000225784.5	ENSG00000225851.1	ENSG00000226816.2	ENSG00000227160.2
	ENSG00000227242.2	ENSG00000228702.1	ENSG00000228997.1	ENSG00000230358.2
	ENSG00000231574.1	ENSG00000231925.7	ENSG00000232224.1	ENSG00000232527.3
	ENSG00000233077.1	ENSG00000233232.2	ENSG00000233295.3	ENSG00000233916.1
	ENSG00000235117.2	ENSG00000235907.2	ENSG00000236624.4	ENSG00000236714.1
	ENSG00000237253.1	ENSG00000237372.1	ENSG00000237510.3	ENSG00000237840.2
	ENSG00000239839.1	ENSG00000240038.2	ENSG00000240050.1	ENSG00000240403.1
	ENSG00000241015.2	ENSG00000241043.1	ENSG00000242441.3	ENSG00000243244.1
	ENSG00000243989.3	ENSG00000245466.1	ENSG00000245937.3	ENSG00000248098.6
	ENSG00000248487.4	ENSG00000250678.1	ENSG00000250786.1	ENSG00000251169.2
	ENSG00000251417.1	ENSG00000251580.1	ENSG00000251606.1	ENSG00000253853.1
	ENSG00000253959.1	ENSG00000254319.1	ENSG00000254761.1	ENSG00000255455.2
	ENSG00000255642.1	ENSG00000256223.1	ENSG00000256433.1	ENSG00000256937.1
	ENSG00000257877.1	ENSG00000258354.1	ENSG00000258408.1	ENSG00000258768.2
	ENSG00000259539.1	ENSG00000261556.4	ENSG00000261804.1	ENSG00000262714.1
	ENSG00000263105.1	ENSG00000266490.1	ENSG00000266912.1	ENSG00000267056.2
	ENSG00000267405.1	ENSG00000267575.2	ENSG00000267623.2	ENSG00000268442.1
	ENSG00000268849.1	ENSG00000271581.1	ENSG00000272102.1	ENSG00000272462.2
**Minor Salivary**	ENSG00000005955.8	ENSG00000014164.6	ENSG00000063854.8	ENSG00000088682.9
	ENSG00000090861.11	ENSG00000092036.12	ENSG00000100577.14	ENSG00000102595.14
	ENSG00000103811.11	ENSG00000105063.14	ENSG00000107854.5	ENSG00000108264.12
	ENSG00000108797.7	ENSG00000110203.4	ENSG00000110768.7	ENSG00000114790.8
	ENSG00000115041.8	ENSG00000115107.15	ENSG00000115310.13	ENSG00000116785.9
	ENSG00000120071.8	ENSG00000120451.6	ENSG00000123453.12	ENSG00000124275.10
	ENSG00000125618.12	ENSG00000125885.9	ENSG00000126822.11	ENSG00000127951.5
	ENSG00000130177.10	ENSG00000133731.5	ENSG00000134716.5	ENSG00000136856.13
	ENSG00000137941.12	ENSG00000140521.7	ENSG00000140835.8	ENSG00000142856.12
	ENSG00000144026.7	ENSG00000146373.12	ENSG00000146414.11	ENSG00000148057.11
	ENSG00000151006.7	ENSG00000152253.4	ENSG00000152784.11	ENSG00000155254.8
	ENSG00000157429.11	ENSG00000157578.9	ENSG00000159110.15	ENSG00000162512.11
	ENSG00000162994.11	ENSG00000164088.13	ENSG00000164855.11	ENSG00000165028.7
	ENSG00000165055.11	ENSG00000169609.9	ENSG00000171234.9	ENSG00000171943.7
	ENSG00000173226.12	ENSG00000174059.12	ENSG00000174652.13	ENSG00000175707.7
	ENSG00000176155.14	ENSG00000178386.8	ENSG00000178896.6	ENSG00000179057.9
	ENSG00000179912.15	ENSG00000179933.4	ENSG00000180481.6	ENSG00000182054.5
	ENSG00000185716.7	ENSG00000186470.9	ENSG00000186976.10	ENSG00000188629.7
	ENSG00000189011.9	ENSG00000197467.9	ENSG00000197568.9	ENSG00000204959.3
	ENSG00000215154.2	ENSG00000223496.1	ENSG00000228307.1	ENSG00000229391.3
	ENSG00000236624.4	ENSG00000238061.2	ENSG00000238078.1	ENSG00000248763.2

	ENSG00000248771.1	ENSG00000250091.2	ENSG00000255455.2	ENSG00000256223.1
	ENSG00000261556.4	ENSG00000261824.2	ENSG00000266733.1	ENSG00000267106.1
	ENSG00000267352.1	ENSG00000267939.1	ENSG00000271550.1	ENSG00000273270.1
**Muscle Skeletal**	ENSG00000005100.8	ENSG00000005243.5	ENSG00000005882.7	ENSG00000005955.8
	ENSG00000006042.7	ENSG00000006453.9	ENSG00000006530.11	ENSG00000008382.11
	ENSG00000008516.12	ENSG00000010017.9	ENSG00000011105.7	ENSG00000013288.4
	ENSG00000049245.8	ENSG00000050767.11	ENSG00000051108.10	ENSG00000051620.6
	ENSG00000054219.9	ENSG00000064687.8	ENSG00000065882.11	ENSG00000066855.11
	ENSG00000067798.9	ENSG00000075239.9	ENSG00000076201.10	ENSG00000076650.2
	ENSG00000077348.4	ENSG00000077782.15	ENSG00000079277.15	ENSG00000082212.7
	ENSG00000085719.7	ENSG00000086200.12	ENSG00000086232.8	ENSG00000087448.5
	ENSG00000088205.8	ENSG00000089022.9	ENSG00000089094.12	ENSG00000089775.7
	ENSG00000090372.10	ENSG00000090861.11	ENSG00000091137.7	ENSG00000092036.12
	ENSG00000095587.8	ENSG00000099956.13	ENSG00000100101.13	ENSG00000100243.16
	ENSG00000100330.11	ENSG00000100612.9	ENSG00000100983.5	ENSG00000101144.8
	ENSG00000101608.8	ENSG00000102683.6	ENSG00000102743.10	ENSG00000102908.16
	ENSG00000103351.8	ENSG00000104852.10	ENSG00000104921.10	ENSG00000104980.3
	ENSG00000105341.14	ENSG00000105610.4	ENSG00000105612.4	ENSG00000105696.4
	ENSG00000105738.6	ENSG00000105880.4	ENSG00000106305.5	ENSG00000107854.5
	ENSG00000108176.10	ENSG00000108272.9	ENSG00000108559.7	ENSG00000108963.13
	ENSG00000109084.9	ENSG00000110076.14	ENSG00000110881.7	ENSG00000110987.4
	ENSG00000111237.14	ENSG00000111361.8	ENSG00000111684.6	ENSG00000111907.16
	ENSG00000112293.10	ENSG00000112294.8	ENSG00000112378.11	ENSG00000112419.10
	ENSG00000112667.8	ENSG00000113758.9	ENSG00000114107.4	ENSG00000114738.6
	ENSG00000114902.9	ENSG00000115350.7	ENSG00000115902.6	ENSG00000116791.9
	ENSG00000116957.8	ENSG00000117226.7	ENSG00000117245.8	ENSG00000117592.8
	ENSG00000118369.8	ENSG00000119147.5	ENSG00000119537.11	ENSG00000120129.5
	ENSG00000120586.4	ENSG00000121864.5	ENSG00000122376.7	ENSG00000122783.12
	ENSG00000123836.10	ENSG00000124098.9	ENSG00000124212.5	ENSG00000124508.12
	ENSG00000124570.13	ENSG00000125775.10	ENSG00000125885.9	ENSG00000126214.16
	ENSG00000126773.8	ENSG00000126804.9	ENSG00000127022.10	ENSG00000127124.9
	ENSG00000127241.12	ENSG00000128335.9	ENSG00000129277.9	ENSG00000129292.16
	ENSG00000129467.9	ENSG00000130193.7	ENSG00000130433.3	ENSG00000130653.11
	ENSG00000130699.12	ENSG00000131435.8	ENSG00000131558.10	ENSG00000131732.7
	ENSG00000132122.7	ENSG00000132842.9	ENSG00000133392.12	ENSG00000133983.10
	ENSG00000134905.12	ENSG00000135426.10	ENSG00000135913.6	ENSG00000135974.5
	ENSG00000136003.11	ENSG00000136011.10	ENSG00000136014.7	ENSG00000136068.10
	ENSG00000136875.8	ENSG00000137274.8	ENSG00000137288.5	ENSG00000137573.9
	ENSG00000137992.10	ENSG00000137996.8	ENSG00000138172.6	ENSG00000138376.6
	ENSG00000138400.8	ENSG00000138801.4	ENSG00000138821.8	ENSG00000139370.6
	ENSG00000139508.10	ENSG00000139835.9	ENSG00000140548.5	ENSG00000140905.5
	ENSG00000141127.10	ENSG00000141140.12	ENSG00000141150.3	ENSG00000141255.8
	ENSG00000141279.11	ENSG00000141384.7	ENSG00000141526.10	ENSG00000142089.11
	ENSG00000142173.10	ENSG00000142973.8	ENSG00000143322.15	ENSG00000143514.12
	ENSG00000143774.12	ENSG00000143776.14	ENSG00000143924.14	ENSG00000143951.11
	ENSG00000144021.2	ENSG00000144134.14	ENSG00000144306.9	ENSG00000144644.10
	ENSG00000145365.10	ENSG00000149308.12	ENSG00000151164.14	ENSG00000151692.10
	ENSG00000152253.4	ENSG00000152291.9	ENSG00000152465.13	ENSG00000153113.19
	ENSG00000153132.8	ENSG00000153246.7	ENSG00000153291.11	ENSG00000153558.9

ENSG00000153786.8	ENSG00000153879.4	ENSG00000153936.12	ENSG00000155066.11
ENSG00000155256.13	ENSG00000155324.5	ENSG00000156269.4	ENSG00000157837.11
ENSG00000157911.5	ENSG00000157927.12	ENSG00000158220.9	ENSG00000158234.8
ENSG00000159267.10	ENSG00000159650.4	ENSG00000160072.15	ENSG00000160959.3
ENSG00000161326.8	ENSG00000161958.6	ENSG00000162076.8	ENSG00000162415.6
ENSG00000162909.13	ENSG00000163257.6	ENSG00000163820.10	ENSG00000164754.8
ENSG00000164904.11	ENSG00000165646.7	ENSG00000165650.7	ENSG00000165995.14
ENSG00000166266.9	ENSG00000166669.9	ENSG00000166949.11	ENSG00000167377.13
ENSG00000167842.11	ENSG00000168411.9	ENSG00000168806.6	ENSG00000169258.6
ENSG00000169710.6	ENSG00000169738.3	ENSG00000169750.4	ENSG00000169764.10
ENSG00000169994.14	ENSG00000170266.11	ENSG00000170390.10	ENSG00000170561.8
ENSG00000170745.7	ENSG00000170906.11	ENSG00000171067.6	ENSG00000171311.8
ENSG00000171467.11	ENSG00000171861.6	ENSG00000171943.7	ENSG00000172215.5
ENSG00000172318.4	ENSG00000172399.5	ENSG00000172409.5	ENSG00000173064.6
ENSG00000173141.4	ENSG00000173226.12	ENSG00000173915.8	ENSG00000174173.6
ENSG00000174194.11	ENSG00000174446.8	ENSG00000175264.3	ENSG00000175348.6
ENSG00000176155.14	ENSG00000176386.4	ENSG00000176463.9	ENSG00000176595.3
ENSG00000176915.10	ENSG00000177674.11	ENSG00000178297.8	ENSG00000178386.8
ENSG00000179562.2	ENSG00000179978.10	ENSG00000180113.11	ENSG00000180185.7
ENSG00000180353.6	ENSG00000180481.6	ENSG00000180771.10	ENSG00000180773.10
ENSG00000180776.11	ENSG00000180881.15	ENSG00000181284.2	ENSG00000181458.6
ENSG00000181938.9	ENSG00000182676.4	ENSG00000182759.3	ENSG00000182836.5
ENSG00000182903.11	ENSG00000183111.7	ENSG00000183751.10	ENSG00000184014.3
ENSG00000184226.10	ENSG00000184389.8	ENSG00000184517.7	ENSG00000184551.4
ENSG00000184886.3	ENSG00000185274.7	ENSG00000185989.9	ENSG00000186026.6
ENSG00000186377.6	ENSG00000186446.7	ENSG00000186470.9	ENSG00000187109.9
ENSG00000187446.7	ENSG00000187676.7	ENSG00000187954.8	ENSG00000188388.9
ENSG00000188629.7	ENSG00000188659.5	ENSG00000189280.3	ENSG00000196358.6
ENSG00000196704.7	ENSG00000196743.4	ENSG00000197208.5	ENSG00000197646.6
ENSG00000197956.5	ENSG00000198105.7	ENSG00000198171.8	ENSG00000198399.10
ENSG00000198576.2	ENSG00000198885.5	ENSG00000198931.6	ENSG00000204237.4
ENSG00000204287.9	ENSG00000204301.5	ENSG00000204381.7	ENSG00000204460.3
ENSG00000204482.6	ENSG00000204520.8	ENSG00000204978.2	ENSG00000204991.6
ENSG00000205678.3	ENSG00000205822.6	ENSG00000213626.7	ENSG00000213906.5
ENSG00000214029.3	ENSG00000214046.4	ENSG00000214871.3	ENSG00000215712.6
ENSG00000215861.4	ENSG00000217644.4	ENSG00000218483.1	ENSG00000221916.2
ENSG00000221990.2	ENSG00000223496.1	ENSG00000224051.2	ENSG00000224097.5
ENSG00000224389.4	ENSG00000224982.2	ENSG00000225241.3	ENSG00000225398.2
ENSG00000225784.5	ENSG00000226094.1	ENSG00000227388.2	ENSG00000227558.4
ENSG00000227621.1	ENSG00000229221.1	ENSG00000229321.1	ENSG00000229659.1
ENSG00000229920.2	ENSG00000231793.4	ENSG00000232237.2	ENSG00000233560.2
ENSG00000233578.1	ENSG00000233961.1	ENSG00000234584.1	ENSG00000235100.3
ENSG00000236624.4	ENSG00000237232.3	ENSG00000237440.4	ENSG00000237510.3
ENSG00000237560.1	ENSG00000240045.1	ENSG00000240204.2	ENSG00000241043.1
ENSG00000241316.2	ENSG00000241837.2	ENSG00000242611.1	ENSG00000243156.3
ENSG00000243244.1	ENSG00000243709.1	ENSG00000245937.3	ENSG00000248098.6
ENSG00000248489.1	ENSG00000248636.2	ENSG00000250075.1	ENSG00000250334.1
ENSG00000250892.1	ENSG00000251226.1	ENSG00000251369.4	ENSG00000251417.1
ENSG00000251580.1	ENSG00000254343.2	ENSG00000254615.2	ENSG00000255748.1

	ENSG00000259224.1	ENSG00000259728.1	ENSG00000260128.2	ENSG00000260743.1
	ENSG00000260979.1	ENSG00000261556.4	ENSG00000261824.2	ENSG00000262165.1
	ENSG00000265750.1	ENSG00000267053.2	ENSG00000267264.1	ENSG00000267939.1
	ENSG00000268442.1	ENSG00000268852.1	ENSG00000269190.1	ENSG00000269282.1
	ENSG00000269554.1	ENSG00000272325.1	ENSG00000272455.1	ENSG00000272462.2
	ENSG00000273356.1			
**Nerve Tibial**	ENSG00000003249.9	ENSG00000005243.5	ENSG00000005448.12	ENSG00000005955.8
	ENSG00000005981.8	ENSG00000006016.6	ENSG00000006042.7	ENSG00000006453.9
	ENSG00000011021.17	ENSG00000013288.4	ENSG00000014641.13	ENSG00000015413.5
	ENSG00000029725.12	ENSG00000030110.8	ENSG00000035687.9	ENSG00000039537.9
	ENSG00000043143.16	ENSG00000043514.11	ENSG00000051620.6	ENSG00000053372.4
	ENSG00000054219.9	ENSG00000055732.8	ENSG00000060982.10	ENSG00000066032.14
	ENSG00000066117.10	ENSG00000066455.8	ENSG00000066855.11	ENSG00000074755.10
	ENSG00000074966.6	ENSG00000075131.5	ENSG00000075461.5	ENSG00000077420.11
	ENSG00000078124.7	ENSG00000079785.10	ENSG00000080815.14	ENSG00000080947.10
	ENSG00000082996.15	ENSG00000083099.6	ENSG00000085719.7	ENSG00000087206.12
	ENSG00000088543.10	ENSG00000088808.12	ENSG00000088836.8	ENSG00000089775.7
	ENSG00000090581.5	ENSG00000091137.7	ENSG00000091262.10	ENSG00000092036.12
	ENSG00000095970.12	ENSG00000096093.10	ENSG00000097046.8	ENSG00000099949.14
	ENSG00000099956.13	ENSG00000099957.12	ENSG00000099968.13	ENSG00000100060.13
	ENSG00000100221.6	ENSG00000100280.12	ENSG00000100330.11	ENSG00000100345.16
	ENSG00000100461.13	ENSG00000100906.6	ENSG00000101460.8	ENSG00000101639.14
	ENSG00000101680.9	ENSG00000101752.7	ENSG00000103942.8	ENSG00000104714.9
	ENSG00000104852.10	ENSG00000105088.4	ENSG00000105290.7	ENSG00000105341.14
	ENSG00000105568.13	ENSG00000105793.11	ENSG00000105821.10	ENSG00000106723.12
	ENSG00000107036.7	ENSG00000107854.5	ENSG00000107864.10	ENSG00000108231.7
	ENSG00000108264.12	ENSG00000108272.9	ENSG00000108799.8	ENSG00000109576.9
	ENSG00000109944.6	ENSG00000110700.2	ENSG00000111275.8	ENSG00000111664.6
	ENSG00000111665.7	ENSG00000111752.6	ENSG00000112175.6	ENSG00000112592.8
	ENSG00000112667.8	ENSG00000113119.8	ENSG00000113318.9	ENSG00000114200.5
	ENSG00000114738.6	ENSG00000115041.8	ENSG00000115266.7	ENSG00000115902.6
	ENSG00000115947.9	ENSG00000116741.6	ENSG00000116785.9	ENSG00000116833.9
	ENSG00000116922.10	ENSG00000116957.8	ENSG00000117461.10	ENSG00000118873.11
	ENSG00000119227.3	ENSG00000119537.11	ENSG00000119977.16	ENSG00000120451.6
	ENSG00000120896.9	ENSG00000121236.15	ENSG00000122376.7	ENSG00000123643.8
	ENSG00000124508.12	ENSG00000125454.7	ENSG00000126215.9	ENSG00000126602.6
	ENSG00000126773.8	ENSG00000126804.9	ENSG00000128713.11	ENSG00000129474.11
	ENSG00000130173.9	ENSG00000130299.12	ENSG00000130592.9	ENSG00000130684.9
	ENSG00000130957.4	ENSG00000131142.9	ENSG00000131969.10	ENSG00000132849.14
	ENSG00000132964.7	ENSG00000133048.8	ENSG00000133107.10	ENSG00000133731.5
	ENSG00000134343.8	ENSG00000134802.13	ENSG00000135333.9	ENSG00000135912.6
	ENSG00000135956.4	ENSG00000136068.10	ENSG00000136235.11	ENSG00000136319.7
	ENSG00000136877.10	ENSG00000136897.6	ENSG00000137312.10	ENSG00000137414.5
	ENSG00000137812.15	ENSG00000137996.8	ENSG00000138152.7	ENSG00000138172.6
	ENSG00000138382.9	ENSG00000139168.3	ENSG00000139200.9	ENSG00000139624.8
	ENSG00000139793.14	ENSG00000140905.5	ENSG00000140990.10	ENSG00000141012.8
	ENSG00000141140.12	ENSG00000141968.3	ENSG00000142544.6	ENSG00000142856.12
	ENSG00000142973.8	ENSG00000143842.10	ENSG00000143951.11	ENSG00000144021.2
	ENSG00000144026.7	ENSG00000145431.6	ENSG00000145569.5	ENSG00000146842.12

ENSG00000148672.7	ENSG00000148843.9	ENSG00000149308.12	ENSG00000150457.7
ENSG00000150656.10	ENSG00000151348.9	ENSG00000152253.4	ENSG00000152382.5
ENSG00000153246.7	ENSG00000153446.11	ENSG00000153786.8	ENSG00000154229.7
ENSG00000154415.3	ENSG00000154645.9	ENSG00000154743.13	ENSG00000154760.9
ENSG00000155324.5	ENSG00000156269.4	ENSG00000157193.10	ENSG00000157335.15
ENSG00000157578.9	ENSG00000157837.11	ENSG00000158122.7	ENSG00000158445.7
ENSG00000159840.11	ENSG00000161888.7	ENSG00000161911.7	ENSG00000161929.10
ENSG00000162366.3	ENSG00000162511.7	ENSG00000162576.12	ENSG00000162779.16
ENSG00000162946.16	ENSG00000162994.11	ENSG00000163017.9	ENSG00000163430.5
ENSG00000163508.8	ENSG00000163618.13	ENSG00000163681.10	ENSG00000164023.10
ENSG00000164338.5	ENSG00000164556.7	ENSG00000164855.11	ENSG00000164880.11
ENSG00000165646.7	ENSG00000165650.7	ENSG00000166333.9	ENSG00000166451.9
ENSG00000166471.6	ENSG00000166669.9	ENSG00000167670.11	ENSG00000167674.10
ENSG00000167755.9	ENSG00000167785.4	ENSG00000167842.11	ENSG00000168079.12
ENSG00000168394.9	ENSG00000168778.7	ENSG00000169612.3	ENSG00000169905.8
ENSG00000169994.14	ENSG00000170144.14	ENSG00000170689.8	ENSG00000170745.7
ENSG00000170899.6	ENSG00000171189.12	ENSG00000171596.6	ENSG00000171649.7
ENSG00000171943.7	ENSG00000171984.10	ENSG00000172901.15	ENSG00000173226.12
ENSG00000174226.4	ENSG00000174652.13	ENSG00000175764.10	ENSG00000175832.8
ENSG00000176155.14	ENSG00000176399.3	ENSG00000177613.7	ENSG00000178201.3
ENSG00000178297.8	ENSG00000178381.7	ENSG00000178386.8	ENSG00000178445.8
ENSG00000178773.10	ENSG00000179399.9	ENSG00000179562.2	ENSG00000179673.3
ENSG00000179958.4	ENSG00000180016.2	ENSG00000180185.7	ENSG00000180481.6
ENSG00000180881.15	ENSG00000180953.7	ENSG00000181007.7	ENSG00000181396.8
ENSG00000182218.5	ENSG00000182557.3	ENSG00000182586.3	ENSG00000182667.10
ENSG00000182853.7	ENSG00000183020.9	ENSG00000183423.7	ENSG00000183831.6
ENSG00000184389.8	ENSG00000184517.7	ENSG00000184672.7	ENSG00000184857.7
ENSG00000185088.8	ENSG00000185800.7	ENSG00000186283.9	ENSG00000186448.10
ENSG00000186470.9	ENSG00000186583.7	ENSG00000186807.9	ENSG00000186889.5
ENSG00000186907.3	ENSG00000187140.4	ENSG00000187239.12	ENSG00000187607.11
ENSG00000188042.5	ENSG00000188610.8	ENSG00000188803.10	ENSG00000188910.7
ENSG00000188938.11	ENSG00000189362.7	ENSG00000196296.9	ENSG00000196313.7
ENSG00000196503.2	ENSG00000196504.11	ENSG00000196743.4	ENSG00000196839.8
ENSG00000196967.6	ENSG00000197146.2	ENSG00000197165.6	ENSG00000197183.8
ENSG00000197254.3	ENSG00000197256.6	ENSG00000197375.8	ENSG00000197646.6
ENSG00000197747.4	ENSG00000197991.10	ENSG00000198060.5	ENSG00000198399.10
ENSG00000198780.7	ENSG00000198931.6	ENSG00000203663.2	ENSG00000203843.3
ENSG00000204256.8	ENSG00000204314.6	ENSG00000204520.8	ENSG00000204599.10
ENSG00000204657.2	ENSG00000204681.6	ENSG00000204977.5	ENSG00000204978.2
ENSG00000205302.2	ENSG00000205683.7	ENSG00000205822.6	ENSG00000213409.4
ENSG00000213471.4	ENSG00000213533.7	ENSG00000213626.7	ENSG00000213983.7
ENSG00000214274.5	ENSG00000214562.9	ENSG00000215440.7	ENSG00000215492.5
ENSG00000215861.4	ENSG00000216775.2	ENSG00000221990.2	ENSG00000222020.2
ENSG00000223496.1	ENSG00000224051.2	ENSG00000224389.4	ENSG00000224728.1
ENSG00000224843.2	ENSG00000225159.1	ENSG00000225194.2	ENSG00000225241.3
ENSG00000225398.2	ENSG00000225784.5	ENSG00000226133.1	ENSG00000227082.1
ENSG00000227388.2	ENSG00000228886.1	ENSG00000228903.2	ENSG00000229373.4
ENSG00000230294.4	ENSG00000231205.7	ENSG00000231360.2	ENSG00000231793.4
ENSG00000231861.1	ENSG00000231925.7	ENSG00000232063.1	ENSG00000232224.1

	ENSG00000233822.3	ENSG00000234231.2	ENSG00000235173.2	ENSG00000236474.1
	ENSG00000236624.4	ENSG00000237238.2	ENSG00000237433.1	ENSG00000237510.3
	ENSG00000238121.1	ENSG00000238181.2	ENSG00000240007.1	ENSG00000240303.3
	ENSG00000240602.3	ENSG00000241399.2	ENSG00000242441.3	ENSG00000242611.1
	ENSG00000242992.2	ENSG00000243156.3	ENSG00000244414.2	ENSG00000244627.1
	ENSG00000244691.1	ENSG00000245937.3	ENSG00000248098.6	ENSG00000248487.4
	ENSG00000248489.1	ENSG00000248515.1	ENSG00000250334.1	ENSG00000250571.2
	ENSG00000250786.1	ENSG00000251254.1	ENSG00000251521.2	ENSG00000251580.1
	ENSG00000253164.1	ENSG00000253433.1	ENSG00000253558.1	ENSG00000253797.2
	ENSG00000254319.1	ENSG00000254339.1	ENSG00000254615.2	ENSG00000254636.1
	ENSG00000254761.1	ENSG00000254810.1	ENSG00000255455.2	ENSG00000256223.1
	ENSG00000256424.1	ENSG00000256433.1	ENSG00000256484.1	ENSG00000256913.1
	ENSG00000257335.4	ENSG00000257511.1	ENSG00000259959.1	ENSG00000259982.1
	ENSG00000260645.1	ENSG00000260910.1	ENSG00000261556.4	ENSG00000263321.1
	ENSG00000263874.1	ENSG00000266490.1	ENSG00000267325.1	ENSG00000267472.1
	ENSG00000268751.1	ENSG00000268927.1	ENSG00000271500.1	ENSG00000271550.1
	ENSG00000271581.1	ENSG00000272129.1	ENSG00000272221.1	ENSG00000272462.2
	ENSG00000272861.1	ENSG00000272899.1	ENSG00000273019.1	ENSG00000273399.1
	ENSG00000273481.1			
	ENSG00000005421.4	ENSG00000005955.8	ENSG00000013288.4	ENSG00000014641.13
	ENSG00000020181.13	ENSG00000051620.6	ENSG00000055732.8	ENSG00000060558.3
	ENSG00000060709.9	ENSG00000064393.11	ENSG00000066032.14	ENSG00000066427.17
	ENSG00000071054.11	ENSG00000075131.5	ENSG00000075336.7	ENSG00000079785.10
**Pancreas**	ENSG00000085644.9	ENSG00000085982.9	ENSG00000088538.12	ENSG00000090863.7
	ENSG00000092036.12	ENSG00000092094.6	ENSG00000096093.10	ENSG00000099800.3
	ENSG00000099956.13	ENSG00000100219.12	ENSG00000100330.11	ENSG00000100362.8
	ENSG00000100461.13	ENSG00000100612.9	ENSG00000101639.14	ENSG00000102996.4
	ENSG00000103351.8	ENSG00000104824.12	ENSG00000105287.8	ENSG00000105483.12
	ENSG00000106351.8	ENSG00000107679.10	ENSG00000107854.5	ENSG00000108055.9
	ENSG00000108272.9	ENSG00000108839.7	ENSG00000109576.9	ENSG00000109790.12
	ENSG00000111331.8	ENSG00000111816.6	ENSG00000111906.13	ENSG00000112294.8
	ENSG00000112796.5	ENSG00000113119.8	ENSG00000113231.9	ENSG00000113318.9
	ENSG00000113328.14	ENSG00000114503.6	ENSG00000115507.5	ENSG00000115523.12
	ENSG00000117122.9	ENSG00000117226.7	ENSG00000117450.9	ENSG00000117477.8
	ENSG00000117569.14	ENSG00000117697.10	ENSG00000119771.10	ENSG00000120662.11
	ENSG00000121236.15	ENSG00000121351.3	ENSG00000121454.4	ENSG00000121753.8
	ENSG00000122025.10	ENSG00000122376.7	ENSG00000122547.6	ENSG00000122641.9
	ENSG00000123843.8	ENSG00000124508.12	ENSG00000124831.14	ENSG00000125378.11
	ENSG00000125848.9	ENSG00000126804.9	ENSG00000127054.14	ENSG00000127507.13
	ENSG00000129993.10	ENSG00000130338.8	ENSG00000131795.8	ENSG00000133110.10
	ENSG00000133313.10	ENSG00000133835.10	ENSG00000133983.10	ENSG00000136682.10
	ENSG00000136717.10	ENSG00000136807.7	ENSG00000136875.8	ENSG00000137261.9
	ENSG00000137996.8	ENSG00000138119.12	ENSG00000138172.6	ENSG00000138347.11
	ENSG00000138777.15	ENSG00000139263.7	ENSG00000139278.5	ENSG00000139508.10
	ENSG00000139998.10	ENSG00000140323.4	ENSG00000140416.15	ENSG00000141127.10
	ENSG00000142599.13	ENSG00000142789.15	ENSG00000142973.8	ENSG00000143153.8
	ENSG00000144021.2	ENSG00000144134.14	ENSG00000144566.6	ENSG00000144730.12
	ENSG00000144827.4	ENSG00000145495.10	ENSG00000146039.6	ENSG00000146205.9
	ENSG00000146457.10	ENSG00000148143.8	ENSG00000148672.7	ENSG00000150526.7

	ENSG00000151131.5	ENSG00000152253.4	ENSG00000153214.5	ENSG00000153246.7
	ENSG00000153495.6	ENSG00000153786.8	ENSG00000154451.10	ENSG00000155254.8
	ENSG00000155324.5	ENSG00000157259.6	ENSG00000157322.12	ENSG00000157379.9
	ENSG00000157837.11	ENSG00000158006.9	ENSG00000158156.7	ENSG00000158234.8
	ENSG00000158825.5	ENSG00000159063.8	ENSG00000159648.7	ENSG00000160172.6
	ENSG00000160200.13	ENSG00000160293.12	ENSG00000161298.12	ENSG00000161326.8
	ENSG00000161904.7	ENSG00000162994.11	ENSG00000163072.10	ENSG00000163541.7
	ENSG00000164171.6	ENSG00000164338.5	ENSG00000164855.11	ENSG00000164880.11
	ENSG00000165282.9	ENSG00000166938.8	ENSG00000166974.8	ENSG00000167257.6
	ENSG00000167674.10	ENSG00000168026.12	ENSG00000169609.9	ENSG00000169710.6
	ENSG00000170634.8	ENSG00000170846.11	ENSG00000171105.9	ENSG00000171914.10
	ENSG00000171943.7	ENSG00000172661.13	ENSG00000173226.12	ENSG00000173567.10
	ENSG00000174194.11	ENSG00000174576.4	ENSG00000174652.13	ENSG00000175166.12
	ENSG00000175283.7	ENSG00000176155.14	ENSG00000176998.3	ENSG00000177628.11
	ENSG00000177674.11	ENSG00000177830.13	ENSG00000178386.8	ENSG00000180071.14
	ENSG00000180185.7	ENSG00000180257.8	ENSG00000180481.6	ENSG00000180730.4
	ENSG00000181007.7	ENSG00000181126.9	ENSG00000182095.10	ENSG00000183161.3
	ENSG00000183166.6	ENSG00000183248.7	ENSG00000183748.4	ENSG00000184040.7
	ENSG00000184110.10	ENSG00000184313.15	ENSG00000184389.8	ENSG00000184428.8
	ENSG00000184956.11	ENSG00000185344.9	ENSG00000185627.13	ENSG00000186448.10
	ENSG00000186907.3	ENSG00000186998.11	ENSG00000187486.5	ENSG00000188388.9
	ENSG00000188396.2	ENSG00000188659.5	ENSG00000188660.3	ENSG00000196458.6
	ENSG00000196859.3	ENSG00000197136.4	ENSG00000197646.6	ENSG00000198624.8
	ENSG00000198832.6	ENSG00000198885.5	ENSG00000204301.5	ENSG00000204520.8
	ENSG00000204525.10	ENSG00000204959.3	ENSG00000205578.4	ENSG00000205822.6
	ENSG00000213096.5	ENSG00000213612.3	ENSG00000213759.4	ENSG00000213761.4
	ENSG00000215861.4	ENSG00000217442.3	ENSG00000218226.1	ENSG00000219073.3
	ENSG00000223496.1	ENSG00000224261.2	ENSG00000224956.5	ENSG00000225217.1
	ENSG00000225241.3	ENSG00000225489.2	ENSG00000228038.1	ENSG00000229836.1
	ENSG00000230982.1	ENSG00000231360.2	ENSG00000232882.1	ENSG00000236417.2
	ENSG00000236624.4	ENSG00000237510.3	ENSG00000240241.1	ENSG00000240356.2
	ENSG00000241015.2	ENSG00000241043.1	ENSG00000241839.5	ENSG00000241926.1
	ENSG00000243244.1	ENSG00000243896.3	ENSG00000245937.3	ENSG00000246922.4
	ENSG00000248771.1	ENSG00000249464.1	ENSG00000249915.3	ENSG00000250786.1
	ENSG00000251491.2	ENSG00000251580.1	ENSG00000254872.2	ENSG00000256166.1
	ENSG00000256223.1	ENSG00000256433.1	ENSG00000257207.4	ENSG00000258884.1
	ENSG00000259683.1	ENSG00000259727.1	ENSG00000259959.1	ENSG00000260077.1
	ENSG00000261135.1	ENSG00000261455.1	ENSG00000261556.4	ENSG00000262074.3
	ENSG00000262686.1	ENSG00000262814.2	ENSG00000266289.1	ENSG00000266995.1
	ENSG00000267053.2	ENSG00000267651.1	ENSG00000271550.1	ENSG00000271821.1
**Pituitary**	ENSG00000000460.12	ENSG00000004838.9	ENSG00000005421.4	ENSG00000042813.3
	ENSG00000051620.6	ENSG00000054219.9	ENSG00000075239.9	ENSG00000079785.10
	ENSG00000092036.12	ENSG00000099800.3	ENSG00000099956.13	ENSG00000100271.12
	ENSG00000100330.11	ENSG00000101460.8	ENSG00000102924.7	ENSG00000103852.8
	ENSG00000103855.13	ENSG00000105854.8	ENSG00000106785.10	ENSG00000107854.5
	ENSG00000108264.12	ENSG00000108278.7	ENSG00000109381.15	ENSG00000109576.9
	ENSG00000110455.9	ENSG00000110906.8	ENSG00000111846.11	ENSG00000115255.6
	ENSG00000116785.9	ENSG00000117226.7	ENSG00000117477.8	ENSG00000120451.6
	ENSG00000120539.10	ENSG00000120688.7	ENSG00000120910.10	ENSG00000121542.7

	ENSG00000122376.7	ENSG00000124508.12	ENSG00000126602.6	ENSG00000130147.11
	ENSG00000132963.7	ENSG00000133983.10	ENSG00000134152.6	ENSG00000134183.7
	ENSG00000134452.15	ENSG00000134905.12	ENSG00000136378.10	ENSG00000136717.10
	ENSG00000138629.11	ENSG00000138814.12	ENSG00000140400.10	ENSG00000141294.5
	ENSG00000142798.12	ENSG00000144747.10	ENSG00000145949.8	ENSG00000146457.10
	ENSG00000147535.12	ENSG00000148672.7	ENSG00000148834.8	ENSG00000152359.10
	ENSG00000153162.8	ENSG00000155066.11	ENSG00000155229.16	ENSG00000155368.12
	ENSG00000155714.9	ENSG00000158411.6	ENSG00000158805.7	ENSG00000158987.15
	ENSG00000160404.13	ENSG00000161149.7	ENSG00000161618.5	ENSG00000162994.11
	ENSG00000163331.6	ENSG00000163635.13	ENSG00000164880.11	ENSG00000165178.8
	ENSG00000165623.5	ENSG00000165646.7	ENSG00000166033.7	ENSG00000166171.8
	ENSG00000166206.9	ENSG00000166266.9	ENSG00000166323.8	ENSG00000166938.8
	ENSG00000167081.12	ENSG00000167136.6	ENSG00000167613.11	ENSG00000168411.9
	ENSG00000169031.14	ENSG00000169085.7	ENSG00000170074.15	ENSG00000170175.6
	ENSG00000171124.8	ENSG00000172803.13	ENSG00000173226.12	ENSG00000173486.8
	ENSG00000173531.11	ENSG00000174194.11	ENSG00000174521.7	ENSG00000174652.13
	ENSG00000174749.5	ENSG00000175764.10	ENSG00000176155.14	ENSG00000176476.4
	ENSG00000176998.3	ENSG00000177494.5	ENSG00000178021.9	ENSG00000178028.9
	ENSG00000180185.7	ENSG00000180481.6	ENSG00000180987.3	ENSG00000182223.7
	ENSG00000182704.6	ENSG00000183072.9	ENSG00000183111.7	ENSG00000183291.11
	ENSG00000183662.6	ENSG00000185946.11	ENSG00000186026.6	ENSG00000186051.4
	ENSG00000186470.9	ENSG00000186522.10	ENSG00000188659.5	ENSG00000197279.3
	ENSG00000197321.10	ENSG00000197617.6	ENSG00000198040.6	ENSG00000198721.8
	ENSG00000198945.3	ENSG00000203546.3	ENSG00000203843.3	ENSG00000204525.10
	ENSG00000204574.8	ENSG00000204789.3	ENSG00000204889.6	ENSG00000204970.5
	ENSG00000204991.6	ENSG00000211450.5	ENSG00000215187.5	ENSG00000215548.2
	ENSG00000215861.4	ENSG00000216775.2	ENSG00000217442.3	ENSG00000219545.5
	ENSG00000223496.1	ENSG00000224333.1	ENSG00000224520.2	ENSG00000225241.3
	ENSG00000225648.1	ENSG00000228784.3	ENSG00000230366.5	ENSG00000232063.1
	ENSG00000232411.1	ENSG00000232560.2	ENSG00000233148.2	ENSG00000233469.1
	ENSG00000234031.1	ENSG00000234040.3	ENSG00000234371.5	ENSG00000235264.1
	ENSG00000235821.1	ENSG00000235933.1	ENSG00000236255.1	ENSG00000236624.4
	ENSG00000236940.1	ENSG00000238021.2	ENSG00000240563.1	ENSG00000242114.1
	ENSG00000242193.5	ENSG00000248487.4	ENSG00000250130.1	ENSG00000250903.4
	ENSG00000251188.1	ENSG00000251576.1	ENSG00000251580.1	ENSG00000253558.1
	ENSG00000258896.1	ENSG00000260077.1	ENSG00000261068.1	ENSG00000261556.4
	ENSG00000266490.1	ENSG00000267053.2	ENSG00000267541.1	ENSG00000267623.2
	ENSG00000267939.1	ENSG00000268442.1	ENSG00000269554.1	ENSG00000271172.1
	ENSG00000271550.1	ENSG00000271623.1	ENSG00000272537.1	ENSG00000273081.1
	ENSG00000273270.1			
	ENSG00000005955.8	ENSG00000005981.8	ENSG00000011332.15	ENSG00000013288.4
	ENSG00000014641.13	ENSG00000026025.9	ENSG00000035687.9	ENSG00000048707.9
	ENSG00000050165.13	ENSG00000050628.16	ENSG00000054654.11	ENSG00000063177.8
	ENSG00000064218.4	ENSG00000066855.11	ENSG00000070770.4	ENSG00000071794.11
**Skin Not Sun**	ENSG00000073008.10	ENSG00000073067.9	ENSG00000075142.9	ENSG00000083099.6
	ENSG00000085719.7	ENSG00000086288.7	ENSG00000088538.12	ENSG00000090013.5
	ENSG00000091262.10	ENSG00000092036.12	ENSG00000092964.12	ENSG00000096093.10
	ENSG00000099800.3	ENSG00000099949.14	ENSG00000099956.13	ENSG00000100191.4
	ENSG00000100345.16	ENSG00000100823.7	ENSG00000101639.14	ENSG00000103043.10

ENSG00000103599.15	ENSG00000104852.10	ENSG00000105290.7	ENSG00000105793.11
ENSG00000106853.12	ENSG00000107679.10	ENSG00000107854.5	ENSG00000108264.12
ENSG00000108278.7	ENSG00000108509.16	ENSG00000108559.7	ENSG00000108590.6
ENSG00000108799.8	ENSG00000109534.12	ENSG00000109576.9	ENSG00000110660.10
ENSG00000110700.2	ENSG00000111237.14	ENSG00000111530.8	ENSG00000112146.12
ENSG00000112378.11	ENSG00000113318.9	ENSG00000115053.11	ENSG00000115257.11
ENSG00000115523.12	ENSG00000115568.11	ENSG00000116337.11	ENSG00000116785.9
ENSG00000117226.7	ENSG00000117682.12	ENSG00000119938.8	ENSG00000120008.11
ENSG00000120451.6	ENSG00000120669.11	ENSG00000121236.15	ENSG00000121454.4
ENSG00000122025.10	ENSG00000122376.7	ENSG00000122565.14	ENSG00000123191.9
ENSG00000123219.8	ENSG00000123989.9	ENSG00000124207.12	ENSG00000124508.12
ENSG00000125851.5	ENSG00000126749.10	ENSG00000126773.8	ENSG00000126804.9
ENSG00000127249.10	ENSG00000127364.2	ENSG00000127954.8	ENSG00000129277.9
ENSG00000129467.9	ENSG00000130529.11	ENSG00000130559.14	ENSG00000130669.13
ENSG00000130684.9	ENSG00000132821.7	ENSG00000132879.9	ENSG00000133313.10
ENSG00000133731.5	ENSG00000133983.10	ENSG00000134283.13	ENSG00000134291.7
ENSG00000135069.9	ENSG00000135213.8	ENSG00000135333.9	ENSG00000135341.13
ENSG00000136247.10	ENSG00000136682.10	ENSG00000136717.10	ENSG00000136807.7
ENSG00000138119.12	ENSG00000138172.6	ENSG00000138801.4	ENSG00000139178.6
ENSG00000139278.5	ENSG00000139343.6	ENSG00000139714.8	ENSG00000140474.8
ENSG00000140623.9	ENSG00000141140.12	ENSG00000142046.10	ENSG00000142208.11
ENSG00000142599.13	ENSG00000142973.8	ENSG00000143514.12	ENSG00000143742.8
ENSG00000143847.11	ENSG00000143952.15	ENSG00000144021.2	ENSG00000144026.7
ENSG00000144134.14	ENSG00000144306.9	ENSG00000144827.4	ENSG00000145391.9
ENSG00000146215.9	ENSG00000146457.10	ENSG00000148843.9	ENSG00000148908.10
ENSG00000149179.9	ENSG00000150540.9	ENSG00000150753.7	ENSG00000150961.10
ENSG00000151348.9	ENSG00000151612.11	ENSG00000151948.7	ENSG00000152253.4
ENSG00000153786.8	ENSG00000153930.6	ENSG00000154240.12	ENSG00000155254.8
ENSG00000155256.13	ENSG00000156103.11	ENSG00000156414.14	ENSG00000156639.7
ENSG00000157259.6	ENSG00000157322.12	ENSG00000157379.9	ENSG00000157911.5
ENSG00000158106.8	ENSG00000158220.9	ENSG00000158786.4	ENSG00000159256.8
ENSG00000159873.5	ENSG00000159899.10	ENSG00000160172.6	ENSG00000160392.9
ENSG00000160867.10	ENSG00000161381.9	ENSG00000161904.7	ENSG00000162040.5
ENSG00000162994.11	ENSG00000163032.7	ENSG00000163072.10	ENSG00000163743.9
ENSG00000163781.8	ENSG00000163872.11	ENSG00000163938.12	ENSG00000164331.5
ENSG00000164338.5	ENSG00000164556.7	ENSG00000164619.4	ENSG00000164855.11
ENSG00000164880.11	ENSG00000165188.9	ENSG00000165280.11	ENSG00000165406.11
ENSG00000165695.5	ENSG00000165731.13	ENSG00000165899.6	ENSG00000166118.3
ENSG00000166289.5	ENSG00000166333.9	ENSG00000166396.8	ENSG00000166451.9
ENSG00000166455.9	ENSG00000166510.9	ENSG00000166913.8	ENSG00000166949.11
ENSG00000167377.13	ENSG00000167562.7	ENSG00000167674.10	ENSG00000169410.5
ENSG00000170175.6	ENSG00000170906.11	ENSG00000170915.8	ENSG00000171102.10
ENSG00000171219.8	ENSG00000171234.9	ENSG00000171953.11	ENSG00000171984.10
ENSG00000172197.9	ENSG00000172987.8	ENSG00000173040.8	ENSG00000173226.12
ENSG00000173267.9	ENSG00000173451.2	ENSG00000173578.6	ENSG00000174194.11
ENSG00000174238.10	ENSG00000174652.13	ENSG00000175634.10	ENSG00000175707.7
ENSG00000175764.10	ENSG00000176155.14	ENSG00000176998.3	ENSG00000177674.11
ENSG00000178297.8	ENSG00000178381.7	ENSG00000178386.8	ENSG00000179673.3
ENSG00000179978.10	ENSG00000180185.7	ENSG00000180353.6	ENSG00000180481.6

	ENSG00000180667.6	ENSG00000180953.7	ENSG00000181007.7	ENSG00000182319.5
	ENSG00000182704.6	ENSG00000182747.4	ENSG00000182771.13	ENSG00000183161.3
	ENSG00000184428.8	ENSG00000184517.7	ENSG00000185019.12	ENSG00000185298.8
	ENSG00000186026.6	ENSG00000186377.6	ENSG00000186446.7	ENSG00000186448.10
	ENSG00000186470.9	ENSG00000186652.5	ENSG00000186977.2	ENSG00000187193.8
	ENSG00000187239.12	ENSG00000187527.6	ENSG00000188295.10	ENSG00000188659.5
	ENSG00000188997.3	ENSG00000196172.8	ENSG00000196247.7	ENSG00000196260.3
	ENSG00000196422.6	ENSG00000196458.6	ENSG00000196653.7	ENSG00000196660.6
	ENSG00000196712.12	ENSG00000196917.4	ENSG00000197146.2	ENSG00000197646.6
	ENSG00000197935.6	ENSG00000198146.4	ENSG00000198171.8	ENSG00000198520.6
	ENSG00000198553.4	ENSG00000198885.5	ENSG00000198945.3	ENSG00000203780.6
	ENSG00000203805.6	ENSG00000204228.3	ENSG00000204520.8	ENSG00000204529.3
	ENSG00000204713.6	ENSG00000204959.3	ENSG00000205746.5	ENSG00000205822.6
	ENSG00000212734.4	ENSG00000213626.7	ENSG00000214659.4	ENSG00000215712.6
	ENSG00000215861.4	ENSG00000223345.3	ENSG00000223496.1	ENSG00000224228.2
	ENSG00000224914.2	ENSG00000225506.2	ENSG00000225556.1	ENSG00000225784.5
	ENSG00000226237.1	ENSG00000227676.2	ENSG00000227718.1	ENSG00000229912.1
	ENSG00000230006.3	ENSG00000230291.4	ENSG00000230869.1	ENSG00000230882.1
	ENSG00000231324.1	ENSG00000231861.1	ENSG00000232931.1	ENSG00000233217.1
	ENSG00000233942.1	ENSG00000235098.4	ENSG00000235117.2	ENSG00000235602.4
	ENSG00000235821.1	ENSG00000236474.1	ENSG00000236624.4	ENSG00000237440.4
	ENSG00000237510.3	ENSG00000237927.1	ENSG00000238266.1	ENSG00000239473.1
	ENSG00000239524.2	ENSG00000240204.2	ENSG00000240370.2	ENSG00000240891.2
	ENSG00000241316.2	ENSG00000242247.6	ENSG00000243802.2	ENSG00000243896.3
	ENSG00000244041.3	ENSG00000248323.1	ENSG00000250251.2	ENSG00000250334.1
	ENSG00000250571.2	ENSG00000250786.1	ENSG00000250853.1	ENSG00000250918.2
	ENSG00000251580.1	ENSG00000251655.2	ENSG00000254715.2	ENSG00000254761.1
	ENSG00000255374.1	ENSG00000255455.2	ENSG00000256223.1	ENSG00000258768.2
	ENSG00000258879.1	ENSG00000259539.1	ENSG00000259683.1	ENSG00000259959.1
	ENSG00000260077.1	ENSG00000260186.1	ENSG00000260645.1	ENSG00000261455.1
	ENSG00000261556.4	ENSG00000261701.2	ENSG00000261754.2	ENSG00000261770.1
	ENSG00000262165.1	ENSG00000263164.1	ENSG00000264868.1	ENSG00000266753.2
	ENSG00000267575.2	ENSG00000267767.2	ENSG00000267795.1	ENSG00000267939.1
	ENSG00000268442.1	ENSG00000269976.1	ENSG00000271161.1	ENSG00000271550.1
	ENSG00000271623.1	ENSG00000272221.1	ENSG00000272462.2	ENSG00000272810.1
	ENSG00000273173.1	ENSG00000273281.1	ENSG00000273356.1	
	ENSG00000003249.9	ENSG00000005471.11	ENSG00000005812.6	ENSG00000005955.8
	ENSG00000005981.8	ENSG00000011332.15	ENSG00000013288.4	ENSG00000035687.9
	ENSG00000050327.10	ENSG00000050628.16	ENSG00000060982.10	ENSG00000063438.12
	ENSG00000065809.9	ENSG00000065911.7	ENSG00000066855.11	ENSG00000069345.7
	ENSG00000071794.11	ENSG00000073067.9	ENSG00000074211.9	ENSG00000074527.7
	ENSG00000075275.12	ENSG00000081665.9	ENSG00000085998.9	ENSG00000087157.14
	ENSG00000088538.12	ENSG00000088876.7	ENSG00000089775.7	ENSG00000090512.7
	ENSG00000090612.16	ENSG00000092036.12	ENSG00000092098.12	ENSG00000092330.11
	ENSG00000095906.12	ENSG00000096093.10	ENSG00000099250.13	ENSG00000099800.3
**Skin Sun**	ENSG00000099956.13	ENSG00000100422.9	ENSG00000100612.9	ENSG00000100982.7
**Exposed**	ENSG00000100991.7	ENSG00000101017.9	ENSG00000101144.8	ENSG00000101190.8
	ENSG00000101608.8	ENSG00000101638.9	ENSG00000101639.14	ENSG00000103018.12
	ENSG00000103034.10	ENSG00000104728.11	ENSG00000104852.10	ENSG00000105792.15

ENSG00000105793.11	ENSG00000106571.8	ENSG00000106771.8	ENSG00000107338.8
ENSG00000107679.10	ENSG00000107854.5	ENSG00000108278.7	ENSG00000108559.7
ENSG00000108785.7	ENSG00000108799.8	ENSG00000108960.3	ENSG00000109265.8
ENSG00000109390.7	ENSG00000109534.12	ENSG00000109576.9	ENSG00000110777.7
ENSG00000111237.14	ENSG00000111664.6	ENSG00000111843.9	ENSG00000112062.6
ENSG00000112146.12	ENSG00000112651.7	ENSG00000112763.11	ENSG00000113141.11
ENSG00000113318.9	ENSG00000114107.4	ENSG00000114200.5	ENSG00000114904.8
ENSG00000115484.10	ENSG00000116750.9	ENSG00000116922.10	ENSG00000116957.8
ENSG00000116977.14	ENSG00000117448.9	ENSG00000117592.8	ENSG00000117682.12
ENSG00000117877.6	ENSG00000117899.6	ENSG00000120158.7	ENSG00000120451.6
ENSG00000121236.15	ENSG00000121335.10	ENSG00000122025.10	ENSG00000122376.7
ENSG00000123989.9	ENSG00000124019.9	ENSG00000124145.5	ENSG00000124508.12
ENSG00000126773.8	ENSG00000126804.9	ENSG00000127054.14	ENSG00000127249.10
ENSG00000128000.11	ENSG00000129214.10	ENSG00000130669.13	ENSG00000130684.9
ENSG00000131142.9	ENSG00000132481.2	ENSG00000132879.9	ENSG00000133731.5
ENSG00000133983.10	ENSG00000134160.9	ENSG00000134283.13	ENSG00000134369.11
ENSG00000134905.12	ENSG00000135164.14	ENSG00000135801.5	ENSG00000136014.7
ENSG00000136059.10	ENSG00000136205.12	ENSG00000136235.11	ENSG00000136379.7
ENSG00000136492.4	ENSG00000136682.10	ENSG00000136717.10	ENSG00000136807.7
ENSG00000137133.6	ENSG00000137177.14	ENSG00000137185.7	ENSG00000137414.5
ENSG00000137710.10	ENSG00000137764.15	ENSG00000137960.5	ENSG00000138119.12
ENSG00000138172.6	ENSG00000138835.18	ENSG00000139178.6	ENSG00000139200.9
ENSG00000139343.6	ENSG00000139433.5	ENSG00000140254.8	ENSG00000140545.10
ENSG00000140623.9	ENSG00000140807.4	ENSG00000140937.9	ENSG00000141140.12
ENSG00000141404.11	ENSG00000141522.7	ENSG00000142459.4	ENSG00000142599.13
ENSG00000142973.8	ENSG00000143552.5	ENSG00000143952.15	ENSG00000144021.2
ENSG00000144134.14	ENSG00000144306.9	ENSG00000144320.9	ENSG00000144566.6
ENSG00000145002.8	ENSG00000147364.12	ENSG00000148057.11	ENSG00000148335.10
ENSG00000148362.6	ENSG00000148843.9	ENSG00000148908.10	ENSG00000149289.6
ENSG00000149503.8	ENSG00000150750.6	ENSG00000150753.7	ENSG00000151090.13
ENSG00000151131.5	ENSG00000151176.3	ENSG00000151348.9	ENSG00000151704.11
ENSG00000151914.13	ENSG00000152137.2	ENSG00000153786.8	ENSG00000153789.8
ENSG00000154133.10	ENSG00000154222.10	ENSG00000155254.8	ENSG00000157322.12
ENSG00000157911.5	ENSG00000158122.7	ENSG00000158220.9	ENSG00000159110.15
ENSG00000159596.6	ENSG00000160007.13	ENSG00000160013.4	ENSG00000160199.10
ENSG00000160867.10	ENSG00000161040.12	ENSG00000161326.8	ENSG00000162040.5
ENSG00000162520.10	ENSG00000162779.16	ENSG00000162994.11	ENSG00000163378.9
ENSG00000163814.3	ENSG00000163935.9	ENSG00000163938.12	ENSG00000163959.5
ENSG00000164393.4	ENSG00000164574.11	ENSG00000164855.11	ENSG00000164880.11
ENSG00000165125.13	ENSG00000165406.11	ENSG00000165476.8	ENSG00000165650.7
ENSG00000165899.6	ENSG00000165912.11	ENSG00000166289.5	ENSG00000166323.8
ENSG00000166669.9	ENSG00000166949.11	ENSG00000167081.12	ENSG00000167232.9
ENSG00000167535.3	ENSG00000167562.7	ENSG00000167699.9	ENSG00000167842.11
ENSG00000168288.8	ENSG00000168671.5	ENSG00000168743.8	ENSG00000168904.10
ENSG00000169413.2	ENSG00000169550.8	ENSG00000170175.6	ENSG00000170549.3
ENSG00000170915.8	ENSG00000171102.10	ENSG00000171132.9	ENSG00000171219.8
ENSG00000171234.9	ENSG00000171467.11	ENSG00000171984.10	ENSG00000172244.4
ENSG00000172270.14	ENSG00000173200.8	ENSG00000173226.12	ENSG00000173267.9
ENSG00000173436.9	ENSG00000173705.4	ENSG00000174194.11	ENSG00000174196.5

	ENSG00000174652.13	ENSG00000175764.10	ENSG00000176142.8	ENSG00000176155.14
	ENSG00000176386.4	ENSG00000176731.7	ENSG00000177590.6	ENSG00000178297.8
	ENSG00000178386.8	ENSG00000178935.5	ENSG00000179240.4	ENSG00000179774.7
	ENSG00000179978.10	ENSG00000180185.7	ENSG00000180336.13	ENSG00000180481.6
	ENSG00000180667.6	ENSG00000180834.3	ENSG00000180875.4	ENSG00000180953.7
	ENSG00000182319.5	ENSG00000182704.6	ENSG00000182771.13	ENSG00000183598.3
	ENSG00000184350.8	ENSG00000184389.8	ENSG00000184428.8	ENSG00000185070.6
	ENSG00000185168.5	ENSG00000185298.8	ENSG00000185627.13	ENSG00000185666.10
	ENSG00000186160.4	ENSG00000186377.6	ENSG00000186470.9	ENSG00000186638.11
	ENSG00000186777.7	ENSG00000187189.9	ENSG00000187239.12	ENSG00000187944.2
	ENSG00000188086.8	ENSG00000188175.5	ENSG00000188388.9	ENSG00000188610.8
	ENSG00000188659.5	ENSG00000188783.5	ENSG00000188897.4	ENSG00000196247.7
	ENSG00000196335.8	ENSG00000196748.5	ENSG00000197008.5	ENSG00000197646.6
	ENSG00000197747.4	ENSG00000197991.10	ENSG00000198155.5	ENSG00000198171.8
	ENSG00000198300.8	ENSG00000198336.5	ENSG00000198346.6	ENSG00000198830.6
	ENSG00000198885.5	ENSG00000198901.9	ENSG00000198931.6	ENSG00000203697.7
	ENSG00000203780.6	ENSG00000204237.4	ENSG00000204267.9	ENSG00000204301.5
	ENSG00000204520.8	ENSG00000204713.6	ENSG00000204790.8	ENSG00000204889.6
	ENSG00000204959.3	ENSG00000205482.7	ENSG00000205562.1	ENSG00000205578.4
	ENSG00000206077.6	ENSG00000211451.7	ENSG00000212734.4	ENSG00000213626.7
	ENSG00000214185.3	ENSG00000214279.8	ENSG00000214290.3	ENSG00000215548.2
	ENSG00000215861.4	ENSG00000221887.4	ENSG00000221990.2	ENSG00000223496.1
	ENSG00000224172.1	ENSG00000224315.2	ENSG00000224389.4	ENSG00000224961.1
	ENSG00000225784.5	ENSG00000226221.1	ENSG00000226397.3	ENSG00000227558.4
	ENSG00000227676.2	ENSG00000229800.1	ENSG00000229912.1	ENSG00000230330.1
	ENSG00000230587.1	ENSG00000230869.1	ENSG00000231816.1	ENSG00000231861.1
	ENSG00000232001.1	ENSG00000232139.1	ENSG00000232629.4	ENSG00000232677.2
	ENSG00000233117.2	ENSG00000233217.1	ENSG00000233232.2	ENSG00000233280.2
	ENSG00000234928.1	ENSG00000235098.4	ENSG00000235117.2	ENSG00000235205.1
	ENSG00000235330.2	ENSG00000235370.5	ENSG00000235677.1	ENSG00000236417.2
	ENSG00000236624.4	ENSG00000237510.3	ENSG00000237560.1	ENSG00000237649.3
	ENSG00000239388.4	ENSG00000240204.2	ENSG00000240303.3	ENSG00000241635.3
	ENSG00000242247.6	ENSG00000242441.3	ENSG00000243444.3	ENSG00000243445.1
	ENSG00000243480.3	ENSG00000243646.4	ENSG00000247982.2	ENSG00000248487.4
	ENSG00000249363.1	ENSG00000249931.3	ENSG00000250334.1	ENSG00000250462.4
	ENSG00000250571.2	ENSG00000251399.1	ENSG00000251521.2	ENSG00000251576.1
	ENSG00000251580.1	ENSG00000254398.1	ENSG00000254480.1	ENSG00000254761.1
	ENSG00000256218.1	ENSG00000256223.1	ENSG00000257877.1	ENSG00000257927.1
	ENSG00000258768.2	ENSG00000258857.1	ENSG00000258879.1	ENSG00000258947.2
	ENSG00000259539.1	ENSG00000259683.1	ENSG00000259959.1	ENSG00000260454.1
	ENSG00000261455.1	ENSG00000261556.4	ENSG00000261754.2	ENSG00000263627.1
	ENSG00000267005.1	ENSG00000267053.2	ENSG00000267264.1	ENSG00000267339.1
	ENSG00000267575.2	ENSG00000267592.1	ENSG00000267648.1	ENSG00000267795.1
	ENSG00000267922.1	ENSG00000267939.1	ENSG00000268621.1	ENSG00000269892.1
	ENSG00000270178.1	ENSG00000270604.1	ENSG00000271040.1	ENSG00000271550.1
	ENSG00000271573.1	ENSG00000271623.1	ENSG00000272462.2	ENSG00000272520.1
	ENSG00000272690.1	ENSG00000272810.1	ENSG00000273143.1	
**Small Intestine**	ENSG00000005421.4	ENSG00000005469.7	ENSG00000052126.10	ENSG00000071909.14
	ENSG00000075131.5	ENSG00000077009.9	ENSG00000085552.12	ENSG00000085719.7

	ENSG00000089356.12	ENSG00000092036.12	ENSG00000101639.14	ENSG00000103226.13
	ENSG00000104231.6	ENSG00000105808.13	ENSG00000108264.12	ENSG00000109576.9
	ENSG00000109610.5	ENSG00000111536.4	ENSG00000111700.8	ENSG00000113119.8
	ENSG00000115107.15	ENSG00000115138.6	ENSG00000116641.11	ENSG00000116785.9
	ENSG00000117222.9	ENSG00000117226.7	ENSG00000117525.9	ENSG00000117758.9
	ENSG00000120451.6	ENSG00000121236.15	ENSG00000122376.7	ENSG00000123191.9
	ENSG00000124613.4	ENSG00000125347.9	ENSG00000125885.9	ENSG00000126254.7
	ENSG00000127249.10	ENSG00000128652.7	ENSG00000129810.10	ENSG00000131142.9
	ENSG00000131480.4	ENSG00000131849.10	ENSG00000132541.6	ENSG00000133731.5
	ENSG00000133983.10	ENSG00000134256.8	ENSG00000134905.12	ENSG00000134955.7
	ENSG00000136003.11	ENSG00000137411.12	ENSG00000137513.5	ENSG00000137869.9
	ENSG00000138756.13	ENSG00000138801.4	ENSG00000141002.14	ENSG00000141349.4
	ENSG00000145907.10	ENSG00000155254.8	ENSG00000155282.7	ENSG00000156049.6
	ENSG00000157259.6	ENSG00000157379.9	ENSG00000157837.11	ENSG00000159140.13
	ENSG00000159788.14	ENSG00000160094.10	ENSG00000163960.7	ENSG00000164338.5
	ENSG00000164398.8	ENSG00000165028.7	ENSG00000165264.6	ENSG00000165650.7
	ENSG00000170448.7	ENSG00000170906.11	ENSG00000171943.7	ENSG00000172020.8
	ENSG00000174194.11	ENSG00000174197.12	ENSG00000174652.13	ENSG00000176155.14
	ENSG00000176204.9	ENSG00000176386.4	ENSG00000177302.10	ENSG00000177830.13
	ENSG00000178297.8	ENSG00000178386.8	ENSG00000180113.11	ENSG00000180185.7
	ENSG00000180481.6	ENSG00000180953.7	ENSG00000184389.8	ENSG00000184445.7
	ENSG00000185339.4	ENSG00000186020.8	ENSG00000186446.7	ENSG00000186470.9
	ENSG00000186652.5	ENSG00000196301.3	ENSG00000196743.4	ENSG00000196967.6
	ENSG00000197008.5	ENSG00000198336.5	ENSG00000198604.6	ENSG00000198691.7
	ENSG00000204301.5	ENSG00000204544.5	ENSG00000204920.6	ENSG00000205822.6
	ENSG00000213386.3	ENSG00000215914.3	ENSG00000219545.5	ENSG00000221990.2
	ENSG00000223496.1	ENSG00000225241.3	ENSG00000225784.5	ENSG00000228421.2
	ENSG00000231360.2	ENSG00000231861.1	ENSG00000235370.5	ENSG00000235821.1
	ENSG00000236624.4	ENSG00000237176.3	ENSG00000237510.3	ENSG00000239857.2
	ENSG00000241015.2	ENSG00000244731.3	ENSG00000249685.1	ENSG00000249806.1
	ENSG00000250075.1	ENSG00000250081.1	ENSG00000250571.2	ENSG00000250658.1
	ENSG00000250786.1	ENSG00000251417.1	ENSG00000253967.1	ENSG00000254319.1
	ENSG00000255455.2	ENSG00000256383.1	ENSG00000259683.1	ENSG00000261556.4
	ENSG00000261600.1	ENSG00000261770.1	ENSG00000263164.1	ENSG00000268172.1
	ENSG00000268442.1	ENSG00000269979.1	ENSG00000271550.1	ENSG00000272462.2
	ENSG00000272823.1	ENSG00000273102.1		
**Spleen**	ENSG00000001084.6	ENSG00000001561.6	ENSG00000006432.11	ENSG00000013288.4
	ENSG00000026950.12	ENSG00000051620.6	ENSG00000051825.10	ENSG00000052749.9
	ENSG00000055813.5	ENSG00000060339.9	ENSG00000071909.14	ENSG00000072840.8
	ENSG00000083099.6	ENSG00000085644.9	ENSG00000086288.7	ENSG00000088882.7
	ENSG00000091140.8	ENSG00000091262.10	ENSG00000093010.7	ENSG00000099338.18
	ENSG00000099956.13	ENSG00000100330.11	ENSG00000101290.9	ENSG00000101639.14
	ENSG00000101680.9	ENSG00000103313.7	ENSG00000104524.9	ENSG00000105793.11
	ENSG00000106070.13	ENSG00000107854.5	ENSG00000107937.14	ENSG00000108264.12
	ENSG00000108590.6	ENSG00000109667.7	ENSG00000110076.14	ENSG00000110801.9
	ENSG00000112031.11	ENSG00000112245.6	ENSG00000113212.4	ENSG00000113318.9
	ENSG00000113356.6	ENSG00000114209.10	ENSG00000116785.9	ENSG00000117226.7
	ENSG00000117682.12	ENSG00000118762.3	ENSG00000119147.5	ENSG00000119242.4
	ENSG00000119915.4	ENSG00000120451.6	ENSG00000121454.4	ENSG00000122694.11

	ENSG00000123643.8	ENSG00000124181.10	ENSG00000124562.5	ENSG00000124588.15
	ENSG00000125812.11	ENSG00000125877.8	ENSG00000125885.9	ENSG00000125970.7
	ENSG00000126214.16	ENSG00000126804.9	ENSG00000127249.10	ENSG00000127507.13
	ENSG00000130684.9	ENSG00000131697.13	ENSG00000131969.10	ENSG00000133731.5
	ENSG00000134256.8	ENSG00000134569.5	ENSG00000134905.12	ENSG00000135956.4
	ENSG00000136197.8	ENSG00000136270.9	ENSG00000136371.5	ENSG00000136877.10
	ENSG00000138134.7	ENSG00000138386.12	ENSG00000140400.10	ENSG00000141012.8
	ENSG00000144021.2	ENSG00000144283.17	ENSG00000145244.7	ENSG00000147883.9
	ENSG00000148229.8	ENSG00000148339.8	ENSG00000149050.5	ENSG00000149474.9
	ENSG00000154719.9	ENSG00000157315.4	ENSG00000157322.12	ENSG00000157429.11
	ENSG00000157764.8	ENSG00000159648.7	ENSG00000160172.6	ENSG00000160226.11
	ENSG00000161714.7	ENSG00000161798.6	ENSG00000162976.8	ENSG00000162994.11
	ENSG00000163092.15	ENSG00000163349.17	ENSG00000164252.8	ENSG00000164880.11
	ENSG00000165406.11	ENSG00000165646.7	ENSG00000165650.7	ENSG00000165912.11
	ENSG00000166268.6	ENSG00000166762.12	ENSG00000166938.8	ENSG00000167785.4
	ENSG00000167792.7	ENSG00000168765.11	ENSG00000168826.11	ENSG00000168827.10
	ENSG00000169609.9	ENSG00000169710.6	ENSG00000170448.7	ENSG00000171649.7
	ENSG00000171840.7	ENSG00000171903.12	ENSG00000171943.7	ENSG00000172878.9
	ENSG00000173226.12	ENSG00000174007.7	ENSG00000174194.11	ENSG00000174652.13
	ENSG00000174775.12	ENSG00000175183.5	ENSG00000175764.10	ENSG00000176155.14
	ENSG00000176222.7	ENSG00000176386.4	ENSG00000177465.4	ENSG00000178386.8
	ENSG00000179673.3	ENSG00000180185.7	ENSG00000180481.6	ENSG00000180549.7
	ENSG00000184451.5	ENSG00000184492.5	ENSG00000184924.5	ENSG00000185499.12
	ENSG00000186020.8	ENSG00000186162.6	ENSG00000186448.10	ENSG00000186470.9
	ENSG00000186998.11	ENSG00000187741.10	ENSG00000187944.2	ENSG00000188388.9
	ENSG00000188610.8	ENSG00000188629.7	ENSG00000188659.5	ENSG00000189127.3
	ENSG00000196126.6	ENSG00000196189.8	ENSG00000196247.7	ENSG00000196458.6
	ENSG00000196743.4	ENSG00000197124.7	ENSG00000197191.3	ENSG00000197646.6
	ENSG00000197961.7	ENSG00000198128.3	ENSG00000198336.5	ENSG00000198483.8
	ENSG00000198746.8	ENSG00000198945.3	ENSG00000198959.7	ENSG00000204020.5
	ENSG00000204520.8	ENSG00000204977.5	ENSG00000212734.4	ENSG00000213453.3
	ENSG00000213626.7	ENSG00000213965.3	ENSG00000213995.7	ENSG00000220685.2
	ENSG00000221946.3	ENSG00000226816.2	ENSG00000228065.6	ENSG00000228146.2
	ENSG00000228412.2	ENSG00000228663.1	ENSG00000228778.1	ENSG00000228970.5
	ENSG00000229373.4	ENSG00000230869.1	ENSG00000231842.1	ENSG00000231861.1
	ENSG00000232063.1	ENSG00000232653.4	ENSG00000234127.4	ENSG00000234618.1
	ENSG00000236624.4	ENSG00000237510.3	ENSG00000237753.1	ENSG00000240563.1
	ENSG00000240754.1	ENSG00000241015.2	ENSG00000241170.2	ENSG00000242247.6
	ENSG00000242689.1	ENSG00000242960.1	ENSG00000244414.2	ENSG00000248884.1
	ENSG00000249465.1	ENSG00000249915.3	ENSG00000250334.1	ENSG00000251580.1
	ENSG00000253558.1	ENSG00000253853.1	ENSG00000254319.1	ENSG00000255455.2
	ENSG00000255513.1	ENSG00000258967.1	ENSG00000260274.1	ENSG00000260910.1
	ENSG00000261252.1	ENSG00000261353.1	ENSG00000261455.1	ENSG00000261556.4
	ENSG00000263305.1	ENSG00000267005.1	ENSG00000269086.2	ENSG00000270972.1
	ENSG00000271109.1	ENSG00000271550.1	ENSG00000271914.1	ENSG00000271943.1
	ENSG00000272129.1	ENSG00000272810.1	ENSG00000273019.1	
	ENSG00000005955.8	ENSG00000005981.8	ENSG00000009844.11	ENSG00000011376.5
	ENSG00000013288.4	ENSG00000014123.9	ENSG00000049246.10	ENSG00000055955.11
**Stomach**	ENSG00000059691.7	ENSG00000063854.8	ENSG00000065154.7	ENSG00000075142.9

ENSG00000085982.9	ENSG00000086065.9	ENSG00000090470.10	ENSG00000100987.10
ENSG00000101639.14	ENSG00000105605.3	ENSG00000105755.3	ENSG00000106069.16
ENSG00000106638.11	ENSG00000107854.5	ENSG00000108278.7	ENSG00000108433.11
ENSG00000108773.6	ENSG00000111752.6	ENSG00000112297.10	ENSG00000112851.10
ENSG00000113318.9	ENSG00000115339.9	ENSG00000115677.12	ENSG00000116704.6
ENSG00000116783.10	ENSG00000116785.9	ENSG00000116922.10	ENSG00000117226.7
ENSG00000117682.12	ENSG00000117899.6	ENSG00000119125.12	ENSG00000119227.3
ENSG00000119537.11	ENSG00000119705.5	ENSG00000120008.11	ENSG00000120451.6
ENSG00000120539.10	ENSG00000121236.15	ENSG00000121454.4	ENSG00000122376.7
ENSG00000124508.12	ENSG00000125409.8	ENSG00000125508.3	ENSG00000126231.9
ENSG00000126804.9	ENSG00000127533.3	ENSG00000130173.9	ENSG00000130684.9
ENSG00000132141.9	ENSG00000133454.11	ENSG00000133731.5	ENSG00000133997.7
ENSG00000134250.13	ENSG00000135063.13	ENSG00000135372.4	ENSG00000136111.8
ENSG00000136877.10	ENSG00000137078.4	ENSG00000137996.8	ENSG00000138119.12
ENSG00000138600.5	ENSG00000138759.13	ENSG00000139436.16	ENSG00000139970.12
ENSG00000139998.10	ENSG00000140400.10	ENSG00000141140.12	ENSG00000141404.11
ENSG00000141985.5	ENSG00000142856.12	ENSG00000144021.2	ENSG00000144026.7
ENSG00000144362.7	ENSG00000144401.10	ENSG00000146457.10	ENSG00000146477.4
ENSG00000148229.8	ENSG00000152253.4	ENSG00000152465.13	ENSG00000153446.11
ENSG00000153786.8	ENSG00000155254.8	ENSG00000156869.8	ENSG00000157211.10
ENSG00000157259.6	ENSG00000159618.11	ENSG00000160447.6	ENSG00000160791.12
ENSG00000161281.6	ENSG00000161381.9	ENSG00000161533.7	ENSG00000162994.11
ENSG00000163083.5	ENSG00000163449.6	ENSG00000163682.11	ENSG00000164880.11
ENSG00000165118.10	ENSG00000165650.7	ENSG00000167377.13	ENSG00000167842.11
ENSG00000168411.9	ENSG00000168564.5	ENSG00000169371.9	ENSG00000169599.8
ENSG00000170191.4	ENSG00000170448.7	ENSG00000170745.7	ENSG00000170802.11
ENSG00000170866.7	ENSG00000171928.9	ENSG00000171940.9	ENSG00000171943.7
ENSG00000172216.4	ENSG00000172340.10	ENSG00000172661.13	ENSG00000173391.4
ENSG00000174226.4	ENSG00000174652.13	ENSG00000176155.14	ENSG00000176222.7
ENSG00000176386.4	ENSG00000176998.3	ENSG00000177191.2	ENSG00000177943.9
ENSG00000178386.8	ENSG00000178952.4	ENSG00000179008.4	ENSG00000180104.11
ENSG00000180185.7	ENSG00000180481.6	ENSG00000180953.7	ENSG00000183506.12
ENSG00000183576.8	ENSG00000184389.8	ENSG00000185689.11	ENSG00000185818.7
ENSG00000185885.11	ENSG00000186446.7	ENSG00000186470.9	ENSG00000187045.12
ENSG00000187848.8	ENSG00000188388.9	ENSG00000188493.10	ENSG00000188629.7
ENSG00000188659.5	ENSG00000188931.3	ENSG00000196126.6	ENSG00000196275.9
ENSG00000196705.4	ENSG00000196993.4	ENSG00000197646.6	ENSG00000197935.6
ENSG00000197937.8	ENSG00000198336.5	ENSG00000198691.7	ENSG00000198756.6
ENSG00000198885.5	ENSG00000198898.8	ENSG00000204435.9	ENSG00000204520.8
ENSG00000204529.3	ENSG00000204616.6	ENSG00000204920.6	ENSG00000213901.6
ENSG00000215237.5	ENSG00000219545.5	ENSG00000223496.1	ENSG00000225217.1
ENSG00000225489.2	ENSG00000225784.5	ENSG00000225791.2	ENSG00000226816.2
ENSG00000227676.2	ENSG00000230358.2	ENSG00000231360.2	ENSG00000232019.1
ENSG00000232104.2	ENSG00000232237.2	ENSG00000233873.1	ENSG00000233913.6
ENSG00000234840.1	ENSG00000235098.4	ENSG00000236297.1	ENSG00000236624.4
ENSG00000236740.2	ENSG00000237176.3	ENSG00000237510.3	ENSG00000237753.1
ENSG00000239883.4	ENSG00000242247.6	ENSG00000242441.3	ENSG00000242611.1
ENSG00000243244.1	ENSG00000249685.1	ENSG00000250266.1	ENSG00000250334.1
ENSG00000250571.2	ENSG00000250673.1	ENSG00000250786.1	ENSG00000251580.1

	ENSG00000251665.1	ENSG00000253558.1	ENSG00000254761.1	ENSG00000255513.1
	ENSG00000255772.1	ENSG00000260645.1	ENSG00000261455.1	ENSG00000261556.4
	ENSG00000262165.1	ENSG00000263627.1	ENSG00000266490.1	ENSG00000267364.1
	ENSG00000267623.2	ENSG00000267939.1	ENSG00000268885.1	ENSG00000269893.2
	ENSG00000272462.2	ENSG00000273372.1		
**Thyroid**	ENSG00000001036.9	ENSG00000003249.9	ENSG00000005194.10	ENSG00000005955.8
	ENSG00000005981.8	ENSG00000006125.12	ENSG00000010379.11	ENSG00000013288.4
	ENSG00000014641.13	ENSG00000015475.14	ENSG00000031823.10	ENSG00000042813.3
	ENSG00000047579.15	ENSG00000051596.5	ENSG00000051620.6	ENSG00000054219.9
	ENSG00000055955.11	ENSG00000057704.6	ENSG00000058091.12	ENSG00000058799.9
	ENSG00000063438.12	ENSG00000065060.12	ENSG00000065328.12	ENSG00000066855.11
	ENSG00000067057.12	ENSG00000070882.8	ENSG00000071794.11	ENSG00000074660.11
	ENSG00000074755.10	ENSG00000075239.9	ENSG00000075303.8	ENSG00000077348.4
	ENSG00000079785.10	ENSG00000080839.7	ENSG00000083099.6	ENSG00000084110.6
	ENSG00000085662.9	ENSG00000085719.7	ENSG00000086189.5	ENSG00000087299.7
	ENSG00000088538.12	ENSG00000088881.16	ENSG00000088882.7	ENSG00000089327.10
	ENSG00000092036.12	ENSG00000095587.8	ENSG00000096433.6	ENSG00000099800.3
	ENSG00000099956.13	ENSG00000100154.10	ENSG00000100612.9	ENSG00000101460.8
	ENSG00000103591.8	ENSG00000103599.15	ENSG00000104112.4	ENSG00000104524.9
	ENSG00000104980.3	ENSG00000105219.4	ENSG00000106133.13	ENSG00000106524.4
	ENSG00000106714.13	ENSG00000106771.8	ENSG00000106992.13	ENSG00000107077.13
	ENSG00000107521.14	ENSG00000107745.12	ENSG00000107854.5	ENSG00000108264.12
	ENSG00000108278.7	ENSG00000108352.7	ENSG00000108381.6	ENSG00000108561.4
	ENSG00000108785.7	ENSG00000108786.6	ENSG00000108963.13	ENSG00000109084.9
	ENSG00000109576.9	ENSG00000110076.14	ENSG00000110851.7	ENSG00000111752.6
	ENSG00000111816.6	ENSG00000112159.7	ENSG00000112175.6	ENSG00000112667.8
	ENSG00000112902.7	ENSG00000112992.12	ENSG00000113318.9	ENSG00000113594.5
	ENSG00000113761.7	ENSG00000113851.9	ENSG00000114127.6	ENSG00000114503.6
	ENSG00000114738.6	ENSG00000115041.8	ENSG00000115042.5	ENSG00000115155.12
	ENSG00000115350.7	ENSG00000115946.3	ENSG00000116473.10	ENSG00000116785.9
	ENSG00000116922.10	ENSG00000116990.9	ENSG00000117245.8	ENSG00000117525.9
	ENSG00000117586.6	ENSG00000117592.8	ENSG00000117682.12	ENSG00000118046.10
	ENSG00000118777.6	ENSG00000118804.7	ENSG00000119537.11	ENSG00000120314.14
	ENSG00000120451.6	ENSG00000120694.15	ENSG00000121297.6	ENSG00000121454.4
	ENSG00000121594.7	ENSG00000121897.9	ENSG00000122376.7	ENSG00000122565.14
	ENSG00000123191.9	ENSG00000124098.9	ENSG00000124380.6	ENSG00000124507.6
	ENSG00000125870.6	ENSG00000126773.8	ENSG00000126804.9	ENSG00000126822.11
	ENSG00000127603.19	ENSG00000128218.7	ENSG00000128313.2	ENSG00000128536.11
	ENSG00000129159.6	ENSG00000129270.11	ENSG00000129993.10	ENSG00000130701.3
	ENSG00000131470.10	ENSG00000131584.14	ENSG00000131620.13	ENSG00000131669.5
	ENSG00000132911.4	ENSG00000132972.14	ENSG00000133256.8	ENSG00000133731.5
	ENSG00000133983.10	ENSG00000134802.13	ENSG00000134905.12	ENSG00000135164.14
	ENSG00000135914.5	ENSG00000136068.10	ENSG00000136715.13	ENSG00000136877.10
	ENSG00000137090.7	ENSG00000137288.5	ENSG00000137996.8	ENSG00000138029.9
	ENSG00000138172.6	ENSG00000138801.4	ENSG00000138942.11	ENSG00000139178.6
	ENSG00000139182.9	ENSG00000139428.7	ENSG00000139714.8	ENSG00000140416.15
	ENSG00000140750.12	ENSG00000141012.8	ENSG00000141140.12	ENSG00000141404.11
	ENSG00000141526.10	ENSG00000141664.5	ENSG00000142065.9	ENSG00000142185.12
	ENSG00000142319.14	ENSG00000142544.6	ENSG00000142599.13	ENSG00000142959.4

ENSG00000142973.8	ENSG00000143036.12	ENSG00000143185.3	ENSG00000143669.9
ENSG00000143924.14	ENSG00000143951.11	ENSG00000144021.2	ENSG00000144026.7
ENSG00000144161.8	ENSG00000144366.11	ENSG00000144580.9	ENSG00000145284.7
ENSG00000145390.7	ENSG00000145506.9	ENSG00000145687.11	ENSG00000145708.6
ENSG00000146007.6	ENSG00000146112.7	ENSG00000146221.8	ENSG00000146540.10
ENSG00000146700.8	ENSG00000147533.12	ENSG00000147573.12	ENSG00000148057.11
ENSG00000148204.7	ENSG00000148400.9	ENSG00000148843.9	ENSG00000149308.12
ENSG00000149311.13	ENSG00000149575.5	ENSG00000150764.9	ENSG00000151176.3
ENSG00000152253.4	ENSG00000152465.13	ENSG00000152492.9	ENSG00000152766.5
ENSG00000153066.8	ENSG00000153246.7	ENSG00000153786.8	ENSG00000154645.9
ENSG00000155254.8	ENSG00000155324.5	ENSG00000156049.6	ENSG00000156232.6
ENSG00000157578.9	ENSG00000158220.9	ENSG00000159840.11	ENSG00000159899.10
ENSG00000160233.6	ENSG00000160293.12	ENSG00000160404.13	ENSG00000160408.10
ENSG00000160593.13	ENSG00000160867.10	ENSG00000161249.16	ENSG00000161618.5
ENSG00000161798.6	ENSG00000161904.7	ENSG00000161960.10	ENSG00000162526.6
ENSG00000162869.11	ENSG00000162994.11	ENSG00000163082.9	ENSG00000163126.10
ENSG00000164294.9	ENSG00000164338.5	ENSG00000164880.11	ENSG00000164976.8
ENSG00000165113.8	ENSG00000165646.7	ENSG00000165650.7	ENSG00000165899.6
ENSG00000166012.10	ENSG00000166266.9	ENSG00000166311.5	ENSG00000166333.9
ENSG00000166938.8	ENSG00000166946.9	ENSG00000166949.11	ENSG00000167384.6
ENSG00000167670.11	ENSG00000167674.10	ENSG00000167747.9	ENSG00000167842.11
ENSG00000167889.8	ENSG00000168411.9	ENSG00000168538.11	ENSG00000168765.11
ENSG00000169375.11	ENSG00000169609.9	ENSG00000169641.9	ENSG00000169718.13
ENSG00000169727.8	ENSG00000169750.4	ENSG00000169894.13	ENSG00000170323.4
ENSG00000170385.9	ENSG00000170906.11	ENSG00000171234.9	ENSG00000171533.7
ENSG00000171596.6	ENSG00000171604.7	ENSG00000171943.7	ENSG00000171984.10
ENSG00000172071.7	ENSG00000172817.3	ENSG00000172965.10	ENSG00000173262.7
ENSG00000173267.9	ENSG00000174194.11	ENSG00000174652.13	ENSG00000175054.10
ENSG00000175550.3	ENSG00000175727.9	ENSG00000175764.10	ENSG00000175899.10
ENSG00000176022.3	ENSG00000176155.14	ENSG00000176358.11	ENSG00000176531.6
ENSG00000176998.3	ENSG00000177302.10	ENSG00000178386.8	ENSG00000179057.9
ENSG00000179397.13	ENSG00000179776.13	ENSG00000179978.10	ENSG00000180185.7
ENSG00000180189.9	ENSG00000180353.6	ENSG00000180481.6	ENSG00000180881.15
ENSG00000180953.7	ENSG00000181666.13	ENSG00000182107.5	ENSG00000182541.13
ENSG00000182704.6	ENSG00000182871.10	ENSG00000183208.8	ENSG00000183439.5
ENSG00000183675.10	ENSG00000183718.4	ENSG00000183831.6	ENSG00000184226.10
ENSG00000184602.5	ENSG00000184995.6	ENSG00000185372.2	ENSG00000186470.9
ENSG00000187091.9	ENSG00000187954.8	ENSG00000188211.4	ENSG00000188388.9
ENSG00000188610.8	ENSG00000188659.5	ENSG00000188906.9	ENSG00000196109.6
ENSG00000196323.7	ENSG00000196329.6	ENSG00000196418.8	ENSG00000196458.6
ENSG00000196611.4	ENSG00000196663.11	ENSG00000196743.4	ENSG00000197146.2
ENSG00000197183.8	ENSG00000197302.6	ENSG00000197375.8	ENSG00000197465.9
ENSG00000197646.6	ENSG00000197747.4	ENSG00000197991.10	ENSG00000198028.3
ENSG00000198128.3	ENSG00000198156.6	ENSG00000198171.8	ENSG00000198590.7
ENSG00000198885.5	ENSG00000198945.3	ENSG00000203965.8	ENSG00000204377.3
ENSG00000204529.3	ENSG00000204681.6	ENSG00000204954.5	ENSG00000204959.3
ENSG00000205020.7	ENSG00000205482.7	ENSG00000205822.6	ENSG00000205929.5
ENSG00000206028.1	ENSG00000211452.6	ENSG00000213123.6	ENSG00000213533.7
ENSG00000213626.7	ENSG00000214145.2	ENSG00000214295.4	ENSG00000214562.9

	ENSG00000214860.4	ENSG00000214982.6	ENSG00000215165.3	ENSG00000215187.5
	ENSG00000215595.1	ENSG00000215861.4	ENSG00000216775.2	ENSG00000217165.1
	ENSG00000218682.1	ENSG00000223496.1	ENSG00000223804.1	ENSG00000224081.3
	ENSG00000225135.1	ENSG00000225154.2	ENSG00000225383.2	ENSG00000225489.2
	ENSG00000225784.5	ENSG00000225851.1	ENSG00000226329.2	ENSG00000226989.1
	ENSG00000227160.2	ENSG00000227755.1	ENSG00000227777.1	ENSG00000227863.2
	ENSG00000228307.1	ENSG00000228418.2	ENSG00000228522.2	ENSG00000228882.1
	ENSG00000229391.3	ENSG00000230561.3	ENSG00000231574.1	ENSG00000231861.1
	ENSG00000231925.7	ENSG00000232212.1	ENSG00000232677.2	ENSG00000232956.4
	ENSG00000233232.2	ENSG00000233280.2	ENSG00000234840.1	ENSG00000235098.4
	ENSG00000235271.1	ENSG00000235619.1	ENSG00000235821.1	ENSG00000236474.1
	ENSG00000236624.4	ENSG00000236992.1	ENSG00000237510.3	ENSG00000238000.1
	ENSG00000238228.1	ENSG00000241015.2	ENSG00000242247.6	ENSG00000242262.1
	ENSG00000242689.1	ENSG00000243431.1	ENSG00000243480.3	ENSG00000244050.2
	ENSG00000245937.3	ENSG00000248487.4	ENSG00000248560.1	ENSG00000249242.3
	ENSG00000250334.1	ENSG00000250412.1	ENSG00000250571.2	ENSG00000250842.1
	ENSG00000251189.1	ENSG00000251221.1	ENSG00000251580.1	ENSG00000253433.1
	ENSG00000253558.1	ENSG00000253598.1	ENSG00000253853.1	ENSG00000254006.1
	ENSG00000254016.2	ENSG00000254319.1	ENSG00000254761.1	ENSG00000254810.1
	ENSG00000255318.1	ENSG00000255455.2	ENSG00000255875.2	ENSG00000256223.1
	ENSG00000257365.3	ENSG00000258647.1	ENSG00000258768.2	ENSG00000258818.2
	ENSG00000258846.1	ENSG00000259959.1	ENSG00000259967.1	ENSG00000260123.1
	ENSG00000261556.4	ENSG00000262118.1	ENSG00000262714.1	ENSG00000263627.1
	ENSG00000264301.1	ENSG00000266405.2	ENSG00000266912.1	ENSG00000267575.2
	ENSG00000267674.1	ENSG00000267819.1	ENSG00000267939.1	ENSG00000269526.1
	ENSG00000270143.1	ENSG00000270480.1	ENSG00000271550.1	ENSG00000271623.1
	ENSG00000272065.1	ENSG00000272221.1	ENSG00000272236.1	ENSG00000272438.1
	ENSG00000272461.1	ENSG00000272462.2	ENSG00000272666.1	ENSG00000272709.1
	ENSG00000272745.1	ENSG00000273230.1		
**Whole Blood**	ENSG00000001561.6	ENSG00000003056.3	ENSG00000005955.8	ENSG00000010219.9
	ENSG00000010361.9	ENSG00000010539.7	ENSG00000013288.4	ENSG00000026950.12
	ENSG00000040531.10	ENSG00000043514.11	ENSG00000049245.8	ENSG00000049541.6
	ENSG00000050767.11	ENSG00000051620.6	ENSG00000066427.17	ENSG00000067836.8
	ENSG00000070444.10	ENSG00000070759.12	ENSG00000071537.9	ENSG00000072401.10
	ENSG00000079691.15	ENSG00000079785.10	ENSG00000080815.14	ENSG00000086288.7
	ENSG00000086730.12	ENSG00000093144.14	ENSG00000096433.6	ENSG00000096717.7
	ENSG00000099949.14	ENSG00000099956.13	ENSG00000100147.9	ENSG00000100462.11
	ENSG00000100485.7	ENSG00000100601.5	ENSG00000101255.6	ENSG00000101327.4
	ENSG00000101639.14	ENSG00000102710.15	ENSG00000103313.7	ENSG00000103852.8
	ENSG00000103966.5	ENSG00000104219.8	ENSG00000104921.10	ENSG00000105085.6
	ENSG00000105223.14	ENSG00000105287.8	ENSG00000105793.11	ENSG00000105854.8
	ENSG00000105875.9	ENSG00000106069.16	ENSG00000106178.2	ENSG00000106341.6
	ENSG00000106804.6	ENSG00000107614.17	ENSG00000107679.10	ENSG00000108064.6
	ENSG00000108264.12	ENSG00000108272.9	ENSG00000108278.7	ENSG00000108479.7
	ENSG00000108523.11	ENSG00000108799.8	ENSG00000109854.9	ENSG00000109944.6
	ENSG00000110987.4	ENSG00000111144.5	ENSG00000111229.11	ENSG00000111913.11
	ENSG00000112293.10	ENSG00000112763.11	ENSG00000112992.12	ENSG00000113141.11
	ENSG00000113318.9	ENSG00000114735.5	ENSG00000114738.6	ENSG00000114779.15
	ENSG00000115808.7	ENSG00000116016.9	ENSG00000116120.8	ENSG00000116741.6

ENSG00000117450.9	ENSG00000117682.12	ENSG00000118246.9	ENSG00000118473.17
ENSG00000118518.11	ENSG00000118922.12	ENSG00000119321.4	ENSG00000119537.11
ENSG00000119844.10	ENSG00000120314.14	ENSG00000120451.6	ENSG00000121068.9
ENSG00000121807.5	ENSG00000121897.9	ENSG00000123143.8	ENSG00000124198.8
ENSG00000124209.3	ENSG00000124535.11	ENSG00000124570.13	ENSG00000125249.6
ENSG00000125734.9	ENSG00000126001.11	ENSG00000126749.10	ENSG00000127249.10
ENSG00000128274.11	ENSG00000128683.9	ENSG00000128923.6	ENSG00000129295.4
ENSG00000130299.12	ENSG00000130803.10	ENSG00000132199.14	ENSG00000132507.13
ENSG00000133056.9	ENSG00000133313.10	ENSG00000133731.5	ENSG00000133985.2
ENSG00000134873.5	ENSG00000134884.9	ENSG00000134905.12	ENSG00000135116.5
ENSG00000135447.12	ENSG00000135605.8	ENSG00000135905.14	ENSG00000135913.6
ENSG00000135929.4	ENSG00000135956.4	ENSG00000137103.12	ENSG00000137185.7
ENSG00000137507.7	ENSG00000137996.8	ENSG00000138821.8	ENSG00000139168.3
ENSG00000139178.6	ENSG00000139278.5	ENSG00000139572.3	ENSG00000140280.9
ENSG00000141013.10	ENSG00000141140.12	ENSG00000141526.10	ENSG00000141959.12
ENSG00000142252.6	ENSG00000142512.10	ENSG00000142599.13	ENSG00000142733.10
ENSG00000143811.12	ENSG00000144021.2	ENSG00000144674.12	ENSG00000145244.7
ENSG00000146859.6	ENSG00000147883.9	ENSG00000149311.13	ENSG00000150753.7
ENSG00000151150.16	ENSG00000151239.9	ENSG00000152061.17	ENSG00000153187.12
ENSG00000153774.4	ENSG00000153982.6	ENSG00000156502.9	ENSG00000158161.11
ENSG00000159445.8	ENSG00000160392.9	ENSG00000160710.11	ENSG00000160716.4
ENSG00000161326.8	ENSG00000161929.10	ENSG00000162511.7	ENSG00000162881.5
ENSG00000162927.9	ENSG00000162997.11	ENSG00000163166.9	ENSG00000163374.15
ENSG00000163754.13	ENSG00000164175.10	ENSG00000164849.7	ENSG00000165030.3
ENSG00000165092.8	ENSG00000166035.6	ENSG00000166669.9	ENSG00000166949.11
ENSG00000166986.8	ENSG00000167085.7	ENSG00000167094.11	ENSG00000167306.14
ENSG00000167460.10	ENSG00000167670.11	ENSG00000167699.9	ENSG00000168268.6
ENSG00000168394.9	ENSG00000168427.7	ENSG00000168765.11	ENSG00000169385.2
ENSG00000169710.6	ENSG00000170448.7	ENSG00000170802.11	ENSG00000171928.9
ENSG00000172244.4	ENSG00000172667.6	ENSG00000172830.8	ENSG00000172965.10
ENSG00000173559.8	ENSG00000173727.7	ENSG00000174123.6	ENSG00000174194.11
ENSG00000174652.13	ENSG00000174950.6	ENSG00000175509.8	ENSG00000175756.9
ENSG00000176155.14	ENSG00000176160.5	ENSG00000176358.11	ENSG00000176731.7
ENSG00000176974.13	ENSG00000178381.7	ENSG00000178977.3	ENSG00000179057.9
ENSG00000180185.7	ENSG00000180353.6	ENSG00000180481.6	ENSG00000180549.7
ENSG00000180881.15	ENSG00000181007.7	ENSG00000181315.6	ENSG00000181458.6
ENSG00000182463.11	ENSG00000183250.7	ENSG00000183309.7	ENSG00000183486.8
ENSG00000183748.4	ENSG00000184990.8	ENSG00000185344.9	ENSG00000185862.5
ENSG00000185963.9	ENSG00000186026.6	ENSG00000186187.7	ENSG00000186470.9
ENSG00000187147.13	ENSG00000187239.12	ENSG00000188026.7	ENSG00000188312.9
ENSG00000188582.4	ENSG00000188610.8	ENSG00000188659.5	ENSG00000189127.3
ENSG00000196126.6	ENSG00000196371.2	ENSG00000196653.7	ENSG00000196743.4
ENSG00000197272.2	ENSG00000197323.6	ENSG00000198089.10	ENSG00000198399.10
ENSG00000198478.6	ENSG00000198483.8	ENSG00000198515.9	ENSG00000198563.9
ENSG00000198624.8	ENSG00000198885.5	ENSG00000198945.3	ENSG00000204020.5
ENSG00000204209.6	ENSG00000204264.4	ENSG00000204388.5	ENSG00000204472.8
ENSG00000204520.8	ENSG00000204655.7	ENSG00000204657.2	ENSG00000204977.5
ENSG00000205020.7	ENSG00000213626.7	ENSG00000213719.4	ENSG00000214975.4
ENSG00000223496.1	ENSG00000224389.4	ENSG00000224831.2	ENSG00000225241.3

	ENSG00000225784.5	ENSG00000225851.1	ENSG00000225872.2	ENSG00000226816.2
	ENSG00000228716.2	ENSG00000229373.4	ENSG00000230869.1	ENSG00000232063.1
	ENSG00000232931.1	ENSG00000233236.1	ENSG00000233266.1	ENSG00000233961.1
	ENSG00000234498.2	ENSG00000235150.1	ENSG00000236624.4	ENSG00000236698.1
	ENSG00000237440.4	ENSG00000237510.3	ENSG00000237609.1	ENSG00000238042.1
	ENSG00000238201.1	ENSG00000240356.2	ENSG00000241484.5	ENSG00000242110.3
	ENSG00000242247.6	ENSG00000242550.1	ENSG00000243566.2	ENSG00000243627.4
	ENSG00000245937.3	ENSG00000248098.6	ENSG00000248196.1	ENSG00000248487.4
	ENSG00000248489.1	ENSG00000249476.1	ENSG00000250334.1	ENSG00000250571.2
	ENSG00000253364.1	ENSG00000255328.1	ENSG00000255455.2	ENSG00000256515.3
	ENSG00000257365.3	ENSG00000258768.2	ENSG00000259865.1	ENSG00000259959.1
	ENSG00000260077.1	ENSG00000260804.2	ENSG00000261824.2	ENSG00000263164.1
	ENSG00000267264.1	ENSG00000270164.1		
